# COVID-19 pleiotropy with brain imaging-derived phenotypes highlights novel insights into the biology shared between immune function and brain variation

**DOI:** 10.1093/braincomms/fcag372

**Published:** 2026-10-05

**Authors:** Qianyu Chen, Jun He, Brenda Cabrera-Mendoza, Ziang Xu, Dan Qiu, Renato Polimanti

**Affiliations:** Department of Psychiatry, Yale University School of Medicine, New Haven, CT 06510, USA; University of Connecticut, Storrs, CT 06269, USA; Department of Psychiatry, Yale University School of Medicine, New Haven, CT 06510, USA; Cooperative Studies Program Clinical Epidemiology Research Center (CSP-CERC), Veterans Affairs Connecticut Healthcare Center, West Haven, CT 06516, USA; Department of Psychiatry, Yale University School of Medicine, New Haven, CT 06510, USA; Cooperative Studies Program Clinical Epidemiology Research Center (CSP-CERC), Veterans Affairs Connecticut Healthcare Center, West Haven, CT 06516, USA; National Centre for Suicide Research and Prevention of Mental Ill-Health (NASP), Department of Learning, Informatics, Management and Ethics, Karolinska Institutet, Stockholm, S-171 77, Sweden; Department of Psychiatry, Yale University School of Medicine, New Haven, CT 06510, USA; Department of Psychiatry, Yale University School of Medicine, New Haven, CT 06510, USA; Cooperative Studies Program Clinical Epidemiology Research Center (CSP-CERC), Veterans Affairs Connecticut Healthcare Center, West Haven, CT 06516, USA; Department of Psychiatry, Yale University School of Medicine, New Haven, CT 06510, USA; Cooperative Studies Program Clinical Epidemiology Research Center (CSP-CERC), Veterans Affairs Connecticut Healthcare Center, West Haven, CT 06516, USA; Department of Biomedical Informatics and Data Science, Yale University School of Medicine, New Haven, CT 06510, USA; Department of Chronic Disease Epidemiology, Yale University School of Public Health, New Haven, CT 06510, USA; Wu Tsai Institute, Yale University, New Haven, CT 06511, USA

**Keywords:** immune system, neurobiology, magnetic resonance imaging, genetics, genome-wide association studies

## Abstract

This study investigated the pleiotropy and the underlying biology linking COVID-19 outcomes to brain variation, leveraging genome-wide data from the COVID-19 Host Genetics Initiative and multimodal brain-wide magnetic resonance imaging from the UK Biobank. In our analyses, COVID-19 outcomes showed evidence of polygenic overlap with respect to 11 imaging-derived phenotypes (IDPs) related to brain structure (e.g. parahippocampal and entorhinal cortical thickness) and connectivity (e.g. Broca’s area and dorsolateral prefrontal cortex). Among these, the genetic liability to COVID-19 hospitalization and SARS-CoV-2 infection was inversely related to the area of pars opercularis. We also identified local genetic correlations between COVID-19 outcomes and brain IDPs within five loci mapping genes involved in inflammatory response, immune regulation, brain development, and neuropsychiatric disorders (e.g. *LAMP3*, *SOX2*, and *TSHZ2*). Additionally, we uncovered colocalized variants linking COVID-19 outcomes to brain structural variation (rs13107325 with entorhinal mean thickness) and brain transcriptomic regulation of immune-related genes (e.g. *IL6R* in brain cortex and *HLA-C* in caudate basal ganglia). Our drug-repurposing analysis based on gene ontologies shared between COVID-19 outcomes and brain IDPs uncovered 41 compounds, including psychiatric medications that appear to also have antiviral and immunomodulatory activity. Overall, the present findings contributed to dissecting the biological mechanisms shared between COVID-19 and brain variation, highlighting the systemic nature of their relationship.

## Teaser

Chen *et al*. report that shared loci involved in immune function and neurodevelopmental processes contribute to the association between COVID-19 outcomes and brain variation. These findings highlight that the systemic nature of this relationship is likely driven by the interplay between central and peripheral systems.

## Graphical Abstract

**Graphical Abstract:**
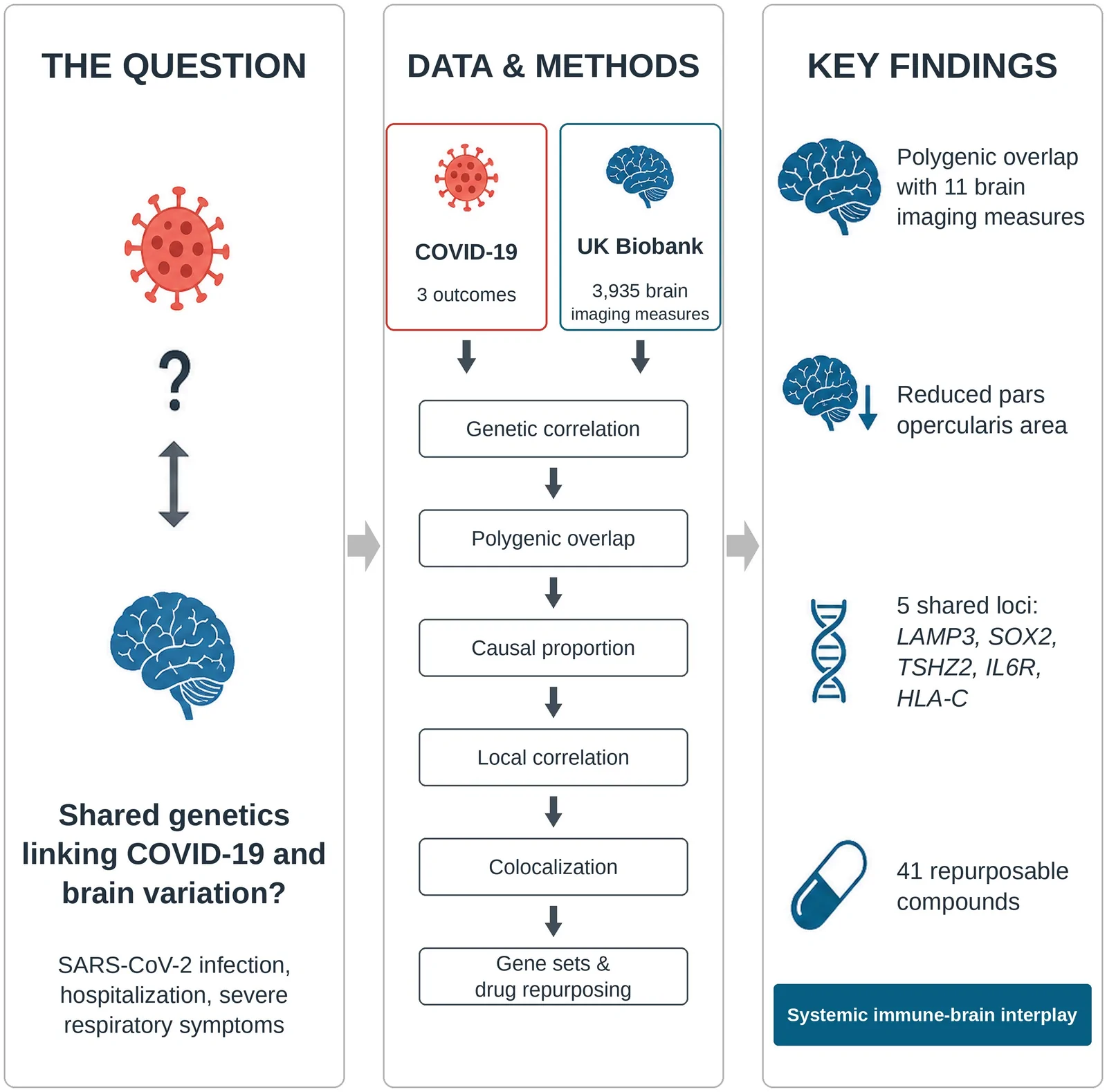

## Introduction

Growing evidence supports the impact of COVID-19 outcomes on brain health through multiple mechanisms, including inflammatory responses, vascular dysfunction, and immune activation.^1^ Neurological symptoms such as cognitive impairment, fatigue, and anosmia are frequently observed among individuals recovering from SARS-CoV-2 infection, and a recent study highlighted their potential relationship with structural and functional brain alterations.^2^ Multiple neuroimaging studies of COVID-19 outcomes reported changes across multiple brain regions, including reductions in global brain size and grey matter volume and thickness, alterations in neural activities and connectivity patterns, and disruptions in regions associated with sensory processing, cognition, memory, and attention.^3-6^ Genetically informed analyses also identified pleiotropic mechanisms linking COVID-19 outcomes to changes in brain structures.^7-9^ Additionally, Mendelian randomization studies reported an effect of severe COVID-19 on Alzheimer’s disease^10^ and cognitive function,^11^ while the genetic liability to major depression appears to affect SARS-CoV-2 infection.^12^

While previous studies contributed to disentangling the dynamics by which COVID-19 outcomes lead to neuropsychiatric symptoms, they have primarily focused on structural changes in cortical brain regions.^3-9^ Multimodal brain-wide phenotyping is expected to provide important insights into the neuroimmune processes contributing to the symptoms observed in certain COVID-19 patients.^13^ Specifically, a brain-wide analysis of COVID-19 outcomes can permit us to generate novel insights into the interplay between immune function and brain biology. Accordingly, assessing the pleiotropy between COVID-19 outcomes and brain volumetric changes can uncover biological pathways related to the impact of hypoxia or inflammation on brain function.^14^ Similarly, investigating the genetic mechanisms shared between COVID-19 outcomes and brain cortical variation can lead to the identification of biological mechanisms responsible for the immunological alterations observed in healthy and impaired individuals.^15^ Additionally, examining imaging-derived phenotypes (IDP) related to white matter, we can explore genetic mechanisms involved in the relationship among immune dysfunction, white matter alteration, and neuroinflammation.^16^ Finally, analyzing the pleiotropy linking COVID-19 outcomes with brain IDPs generated from functional magnetic resonance imaging (MRI) can permit us to increase the still-limited understanding of the interplay between immune system and brain connectivity.^17,18^ Accordingly, understanding the genetic mechanisms linking brain variation to COVID-19 outcomes can advance further the understanding of COVID-19 long-term consequences on brain biology^19^ and on neuropsychiatric and cognitive symptoms observed in certain patients.^20^

In the present study, we leveraged large-scale genomic datasets available from UK Biobank (UKB)-Oxford Brain Imaging Genetics Initiative^21^ and COVID-19 Host Genetics Initiative (HGI)^22,23^ to assess the pleiotropic mechanisms linking brain variation to SARS-CoV-2 infection, COVID-19 hospitalization, and COVID-19 severe respiratory symptoms. These three outcomes represent COVID-19 categories according to predefined and partially overlapping phenotypic criteria previously used by COVID-19 HGI^22^ and have been associated with distinct pleiotropy patterns.^24,25^ By examining their polygenic overlap, individual loci, shared molecular pathways, and potential drug targets in relation to brain IDPs, this study generated novel hypotheses regarding the genetic overlap between COVID-19 pathogenesis and brain variation and regarding the long-term consequences observed in certain patients after the acute phase of the infection.

## Subjects and methods

### Study design

The present study leveraged multiple analytic approaches to understand the pleiotropic mechanisms linking COVID-19 outcomes to brain variation. Because of the limited sample size of the brain IDP genome-wide association studies (GWAS), we initially conducted an exploratory global genetic correlation analysis to identify brain IDPs potentially linked to COVID-19 outcomes, applying a suggestive significance threshold. These were further investigated using multiple analytic approaches. MiXer bivariate models^26^ and the latent causal variable (LCV) analysis^27^ were used to assess polygenic overlap and genetically inferred effects, respectively. With the local analysis of (co)variant association (LAVA) method,^28^ we investigated which genomic regions present statistically significant correlations between genetic effects detected across multiple variants. We performed a colocalization analysis^29^ to detect variants with effects shared among COVID-19 outcomes, brain IDPs, and brain transcriptomic regulation. Finally, we performed a gene-set analysis^30^ to identify gene ontology enrichments shared between COVID-19 outcomes and brain IDPs and analyze their transcriptomic profiles in the context of existing molecular compounds to explore potential drug repurposing relevant for neuroimmune pathways.

The study was conducted leveraging genome-wide association statistics. Owing to the use of previously collected, deidentified data, the analyses conducted did not require institutional review board approval. Because no large-scale GWAS of brain IDPs is available for non-European descent populations, the analyses were limited to data generated from European-ancestry individuals.

### Data sources

COVID-19 genome-wide association statistics were obtained from the COVID-19 HGI.^22^ Specifically, we analyzed COVID-19 HGI Release 7,^23^ which investigated up to 219 692 cases and over 3 million controls (48% to 52% females) across 82 studies from 35 countries. In our study, we focused on three COVID-19 outcomes: (i) COVID-19 severe respiratory symptoms (i.e. COVID-19 cases requiring respiratory support or resulting in death; 13 769 cases and 1 072 442 controls of European ancestry); (ii) COVID-19 hospitalization (i.e. COVID-19 cases hospitalized due to infection-related symptoms; 32 519 cases and 2 062 805 controls of European ancestry); and (iii) SARS-CoV-2 infection (confirmed COVID-19 cases irrespective of symptom severity; 122 616 cases and 2 475 240 controls of European ancestry). HGI COVID-19 data are available at https://www.covid19hg.org/results/r7/.

Genome-wide association statistics of 3935 brain IDPs (Supplemental Table 1) were obtained from the Oxford Brain Imaging Genetics Server.^21,31^ These data were generated from 33 224 participants (52% females) of European descent enrolled in the UKB, a population-based cohort.^32^ UKB brain IDPs consist of measures of regional and tissue volumes, cortical areas, cortical thickness, regional and tissue intensities, cortical grey-white contrast, white matter hyperintensity volumes, regional T2*, white matter tracts, tfMRI activation, rfMRI node amplitude, and rfMRI connectivity, derived from six MRI modalities (T1-weighted and T2-weighted fluid-attenuated inversion recovery (T2-FLAIR) structural images; susceptibility-weighted MRI, swMRI; diffusion MRI, dMRI; task functional MRI, tfMRI; and resting-state functional MRI, rfMRI) and acquired using standardized protocols to ensure consistency and reliability across participants.^33^ Brain IDPs were derived by integrating multiple brain atlases. Details are available at https://biobank.ctsu.ox.ac.uk/crystal/crystal/docs/brain_mri.pdf. UKB brain IDP GWAS data are available at https://open.win.ox.ac.uk/ukbiobank/big40/.

These datasets were analyzed using methods that can account for sample overlap, because UKB is the cohort investigated in the brain IDP GWAS (100% of the sample) and it is also included in COVID-19 HGI GWAS meta-analyses (1–3% of the sample). Specifically, 680 cases and 419 851 controls from UKB were included in the GWAS of severe respiratory symptoms; 3748 cases and 416 783 controls from UKB were included in the GWAS of hospitalization; 14 539 cases and 405 992 controls from UKB were included in the GWAS of SARS-CoV-2 infection.

### Statistical analyses

#### Global genetic correlation

Linkage Disequilibrium Score Regression (LDSC) was used to estimate global genetic correlations (rg; i.e. shared genetic effects across the genome) among COVID-19 phenotypes and brain IDPs.^34^ LD reference panel was based on pre-computed data from European-descent populations available from 1000 Genomes Project Phase 3.^35^ In accordance with LDSC guidelines (available at https://github.com/bulik/ldsc), GWAS datasets were processed using munge_sumstats.py and the LDSC analyses were limited to HapMap3 common variants.^36^ To maximize our discovery power, we considered as significant brain IDPs those with rg *P* < 0.01 with at least two of the three COVID-19 outcomes (i.e. COVID-19 severe respiratory symptoms, COVID-19 hospitalization, and SARS-CoV-2 infection).

#### Local genetic correlation

LAVA was conducted to identify the genomic regions with shared genetic effects (local genetic correlation) between COVID-19 outcomes and brain IDPs.^28^ European-descent populations available from 1000 Genomes Project Phase 3 were used as LD reference panel. Following LAVA instructions (available at https://github.com/josefin-werme/LAVA), the analysis was conducted with respect to 2495 quasi-independent blocks, each around 1 Mb in size. Because of the genetic correlation among brain IDPs and among COVID-19 outcomes, false discovery rate correction (FDR *q* < 0.1) was employed to account for the number of genomic regions tested. With respect to each FDR-significant genomic region, we used the UCSC Genome Browser (Kent *et al*., 2002) to annotate the corresponding coding genes.

#### Latent causal variable analysis

To evaluate the genetic causal relationships underlying the genetic correlation between COVID-19 outcomes and brain IDPs, we conducted the LCV analysis.^27^ This was restricted to brain IDPs with rg *P* < 0.01 with at least two of the three COVID-19 outcomes. We selected the LCV approach, because it can account for GWAS sample overlap and does not require selecting individual loci as instrumental variables.^27^ In the LCV analysis, the genetic causality proportion (gcp) can range from zero (no partial genetic effect) to one (full genetic effect). Positive and negative gcp values reflect the direction of the effect. In the present study, gcp>0 reflected an effect of COVID-19 outcome on brain IDP, while gcp < 0 indicated an effect of brain IDP on COVID-19 outcome. Information regarding the sign of the LCV effect is provided by the LCV rho statistics: rho > 0 corresponds to a positive effect, while rho < 0 corresponds to a negative effect. To define statistically significant gcp estimates, we applied a Bonferroni multiple testing correction accounting for the tests performed (*N* = 23, *P* < 0.002).

#### Colocalization

We conducted colocalization analysis to identify shared genetic effects between COVID-19 outcomes and brain IDPs. Specifically, considering default parameters, we used HyPrColoc deterministic Bayesian algorithm to estimate the posterior probability of full colocalization (PPFC).^29^ Following developers’ recommendation,^29^ we used PPFC > 0.7 as the threshold for identifying colocalization signals. In addition, we prioritized candidate SNPs with the threshold of proportion of the PPFC explained by the candidate variant (P_SNP_ > 0.5). To further investigate the relationship of COVID-19 and brain IDPs with brain biology, we investigated their colocalization with tissue-specific transcriptomic regulation of 13 brain regions available from GTEx v8^37^ (i.e. amygdala, anterior cingulate cortex (BA24), caudate (basal ganglia), cerebellar hemisphere, cerebellum, cortex, frontal cortex (BA9), hippocampus, hypothalamus, nucleus accumbens (basal ganglia), putamen (basal ganglia), spinal cord (cervical c-1), and substantia nigra).

#### Mixture model analyses

We used bivariate mixture models available from MiXer approach^26^ to assess the polygenic overlap between COVID-19 outcomes and brain IDPs, estimating the number of shared variants and their effect concordance rate. Bivariate MixeR models with Akaike information criterion (AIC) for the best model versus the model with minimal/maximum possible polygenic overlap >0 were considered statistically meaningful. GSA-MiXeR approach^30^ was used to investigate mechanisms shared between COVID-19 phenotypes and brain IDPs. This analysis was restricted to gene ontologies (GO) related to biological processes, molecular functions, and cellular components. To account for multiple testing, Bonferroni correction was applied, considering the number of GOs tested (*P* < 6.31 × 10^−7^). To prioritize non-redundant GO terms based on their statistical strength and identify representative domains, we used REVIGO,^38^ considering its default settings (i.e. similarity threshold = 0.7 and UniProt as the reference database).

#### Drug-repurposing

We conducted a drug repurposing analysis to identify molecular compounds associated with transcriptomic profiles overlapping those associated with the GOs identified by GSA-MiXeR. Specifically, we applied the gene2drug approach^39^ to test GOs shared between COVID-19 outcomes and brain IDPs with respect to the connectivity map drug database (1.5 M gene expression profiles from ∼5000 small-molecule compounds, and ∼3000 genetic reagents, tested in multiple cell types).^40^ FDR correction (FDR *q* < 0.05) was applied to define statistically significant gene2drug results after accounting for the number of molecular compounds tested. The sign of the enrichment score derived from gene2drug reflects whether the drug downregulates or upregulates pathways associated with the outcome of interest.

## Results

### Polygenic overlap

Because of the limited sample size of the brain IDP GWAS (*N* = 33 224), we conducted an exploratory global genetic correlation analysis considering a suggestive significance threshold (*P* < 0.01). Eleven brain IDPs passed this exploratory screening (*P* < 0.01) with respect to at least two COVID-19 phenotypes (Supplemental Table 2). Figure 1 illustrates the trends observed in this initial screening analysis. We followed up this suggestive evidence of pleiotropy using MiXer bivariate models. IDP 2834 [rfMRI connectivity ICA100 edge 406: connectivity network among Broca’s area, dorsolateral prefrontal cortex (dlPFC), anterior prefrontal cortex, posterior cingulate cortex, caudate, and left dorsal anterior cingulate cortex; Fig. 2] showed suggestive genetic correlation with the three COVID-19 outcomes tested (rg = 0.26–0.31, *P* < 0.01), but bivariate MixeR models confirmed IDP 2834 polygenic overlap (i.e. shared variants) only with COVID-19 hospitalization and SARS-CoV-2 infection (56% and 43%, respectively; Supplemental Table 3). Additionally, while rfMRI amplitudes ICA100 node 32 (IDP 2195: brain activity related to the right supramarginal gyrus, primary somatosensory cortex, and motor cortex; Fig. 3) also showed comparable polygenic overlap with COVID-19 respiratory symptoms (31%) and SARS-CoV-2 infection (32%), rfMRI connectivity ICA100 edge 403 (IDP 2831: brain connectivity among anterior prefrontal cortex, caudate, Broca’s area, right dlPFC, and right dorsal anterior cingulate cortex; Fig. 4) showed a large difference in the fraction of shared variants between COVID-19 severe respiratory symptoms (14%) and COVID-19 hospitalization (41%; Supplemental Table 3). Regarding brain IDPs related to parahippocampal and entorhinal cortical thickness (IDP: 1069, 1115, 1160, and 1274), the fraction of shared causal variants was higher for COVID-19 hospitalization (>40%) than for COVID-19 severe respiratory symptoms (<15%; Supplemental Table 3). Also, with respect to the brain IDPs with suggestive global genetic correlation with COVID-19 outcomes (Fig. 1; Supplemental Table 2), the fraction of shared variants was larger between COVID-19 hospitalization and COVID-19 severe respiratory symptoms than between COVID-19 hospitalization and SARS-CoV-2 infection (Supplemental Table 3).

**Figure 1:**
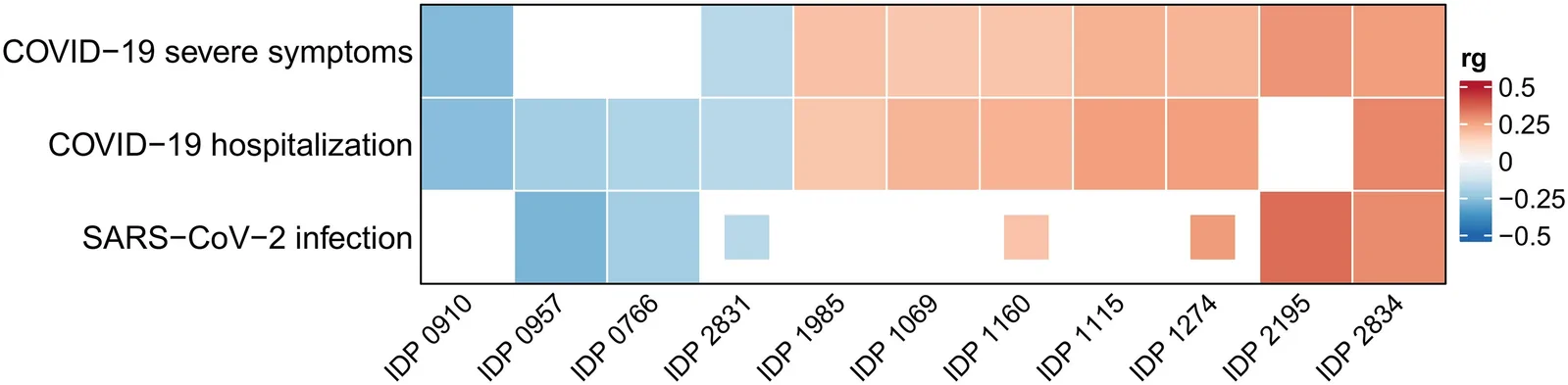
**Genetic correlations between COVID-19 outcomes (severe respiratory symptoms, 13 769 cases and 1 072 442 controls; hospitalization 32 519 cases and 2 062 805 controls; infection, 122 616 cases and 2 475 240 controls) and brain IDP (IDP, assessed in 33 224 participants).** Genetic correlations were estimated using linkage disequilibrium score regression, a method that uses genome-wide association statistics to quantify the genetic influence on one trait shared with another. The results reported are related to brain IDPs with suggestive evidence of genetic correlation at *P* < 0.01 with at least two COVID-19 outcomes (fully coloured cells). To show the full patterns with respect to the remaining COVID-19 outcomes, we also include partially coloured cells to represent nominally significant genetic correlations (0.01 ≤ *P* < 0.05) and blank cells for non-significant estimates (*P* ≥ 0.05). Statistical significance was estimated using a block jackknife approach to estimate standard errors, followed by a two-sided *Z*-test. Full statistics are available in Supplemental Table 2. IDP 766: Area of parsopercularis in the right hemisphere generated by parcellation of the pial surface using Desikan-Killiany parcellation; IDP 910: Area of Lat-Fis-ant-Horizont in the left hemisphere generated by parcellation of the white surface using Destrieux (a2009s) parcellation; IDP 957: Area of G-front-inf-Opercular in the right hemisphere generated by parcellation of the white surface using Destrieux (a2009s) parcellation; 1069: Mean thickness of parahippocampal in the right hemisphere generated by parcellation of the white surface using Desikan-Killiany parcellation; IDP 1115: Mean thickness of entorhinal in the right hemisphere generated by parcellation of the white surface using BA_exvivo parcellation; IDP 1160: Mean thickness of parahippocampal in the right hemisphere generated by parcellation of the white surface using DKT parcellation; IDP 1274: Mean thickness of G-oc-temp-med-Parahip in the right hemisphere generated by parcellation of the white surface using Destrieux (a2009s) parcellation; IDP 1985: Mean OD (orientation dispersion index) in medial lemniscus (right) on FA (fractional anisotropy) skeleton (from dMRI data); IDP 2195: rfMRI amplitudes (ICA100 node 32); IDP 2831: rfMRI connectivity (ICA100 edge 403); IDP 2834: rfMRI connectivity (ICA100 edge 406).

**Figure 2:**
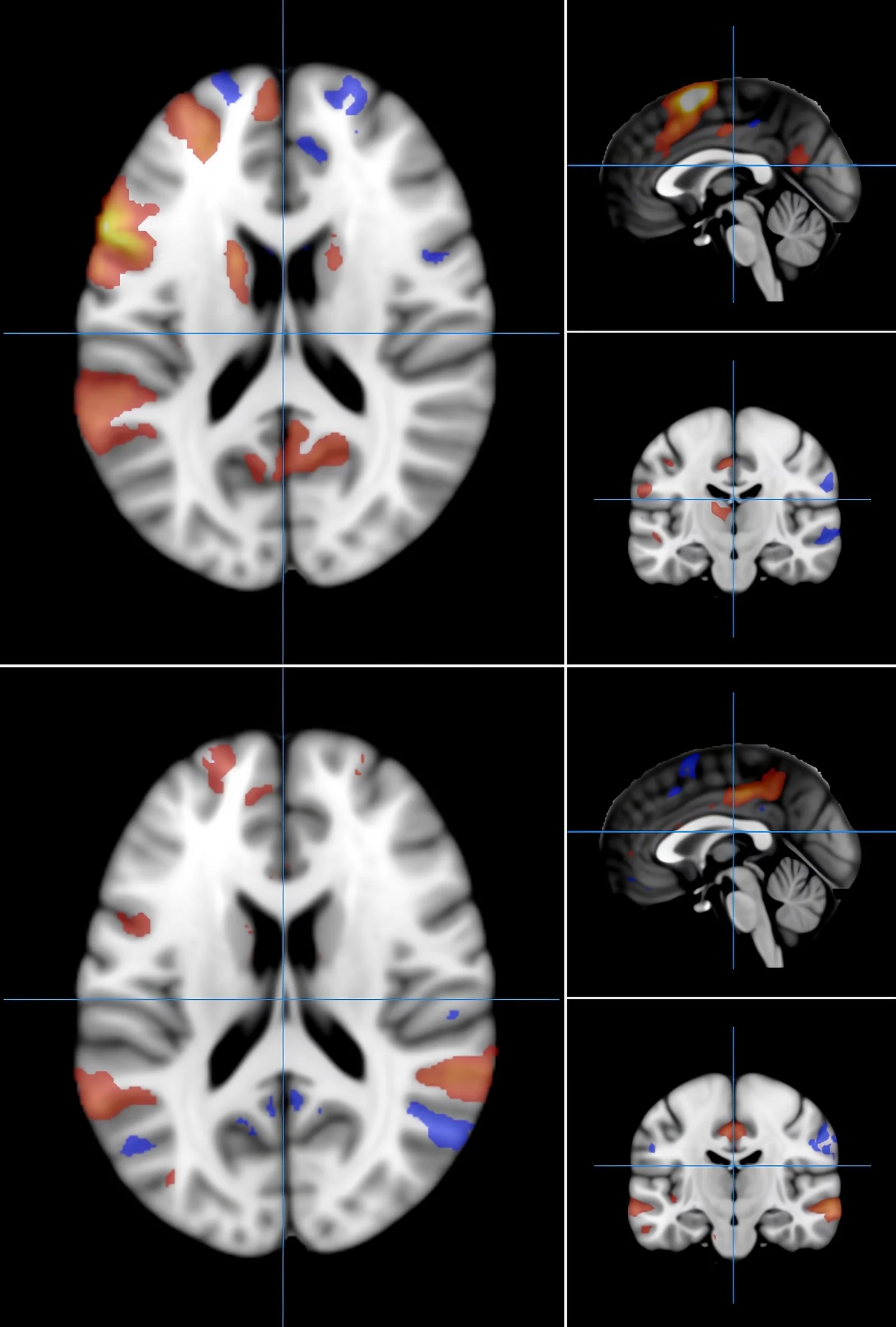
**Graphical representation of brain imaging-derived phenotype (IDP) 2834.** This reflects the edge 406 between nodes 28 (upper panel) and 29 (lower panel) of dimensionality 100 identified through spatial independent component analysis (ICA) of resting-state functional MRI across three anatomical planes: axial (left), sagittal (top right), and coronal (bottom right). Red overlay represents positive values, while negative values are indicated with blue overlay.

**Figure 3:**
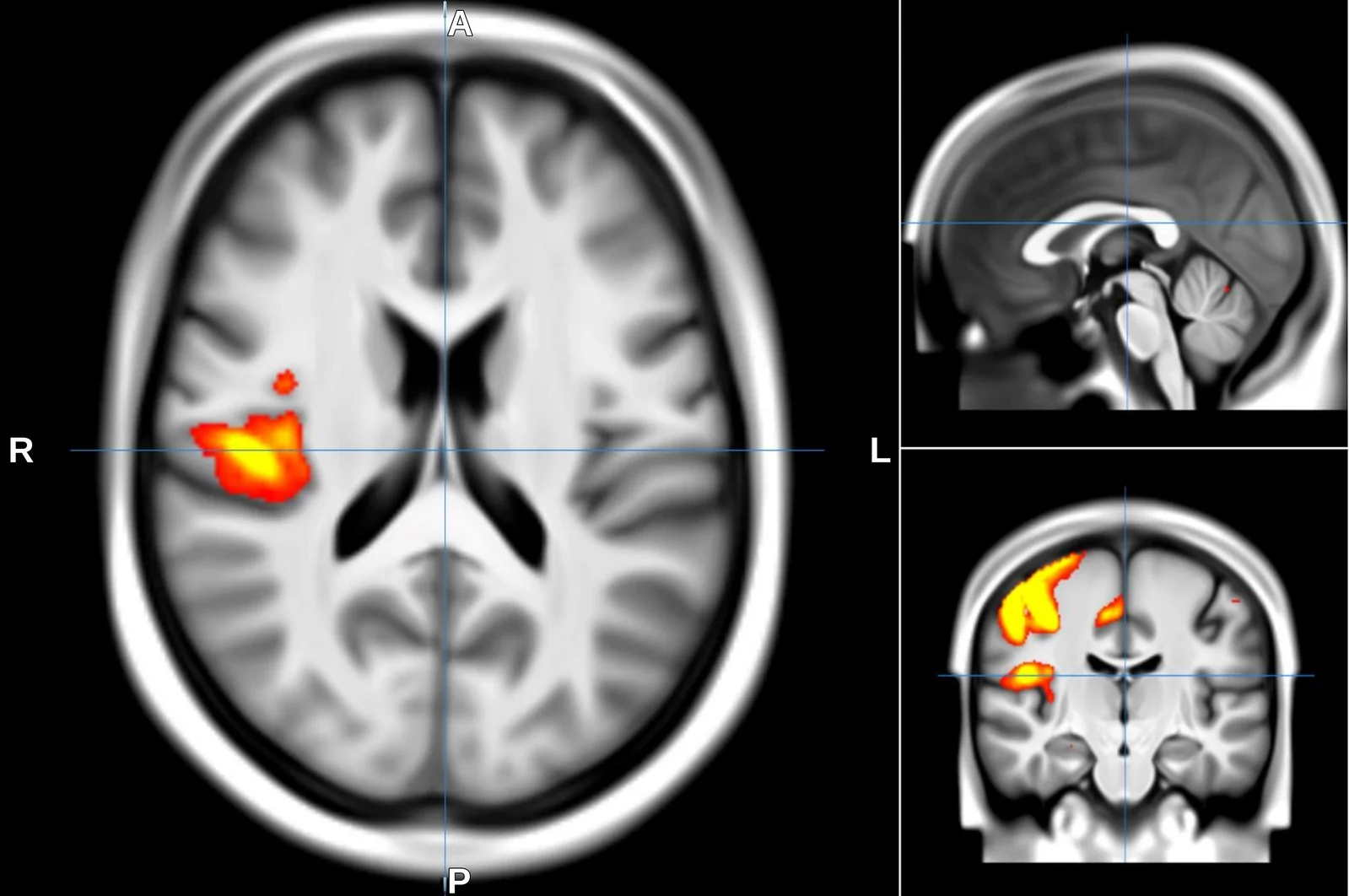
**Graphical representation of brain imaging-derived phenotype (IDP) 2195.** This reflects the amplitudes of node 32 of dimensionality 100 identified by spatial Independent Component Analysis (ICA) of resting-state functional MRI across three anatomical planes: axial (left), sagittal (top right), and coronal (bottom right). On the axial plane, anterior (A), posterior (P), right (R), and left (L) orientations are indicated.

**Figure 4:**
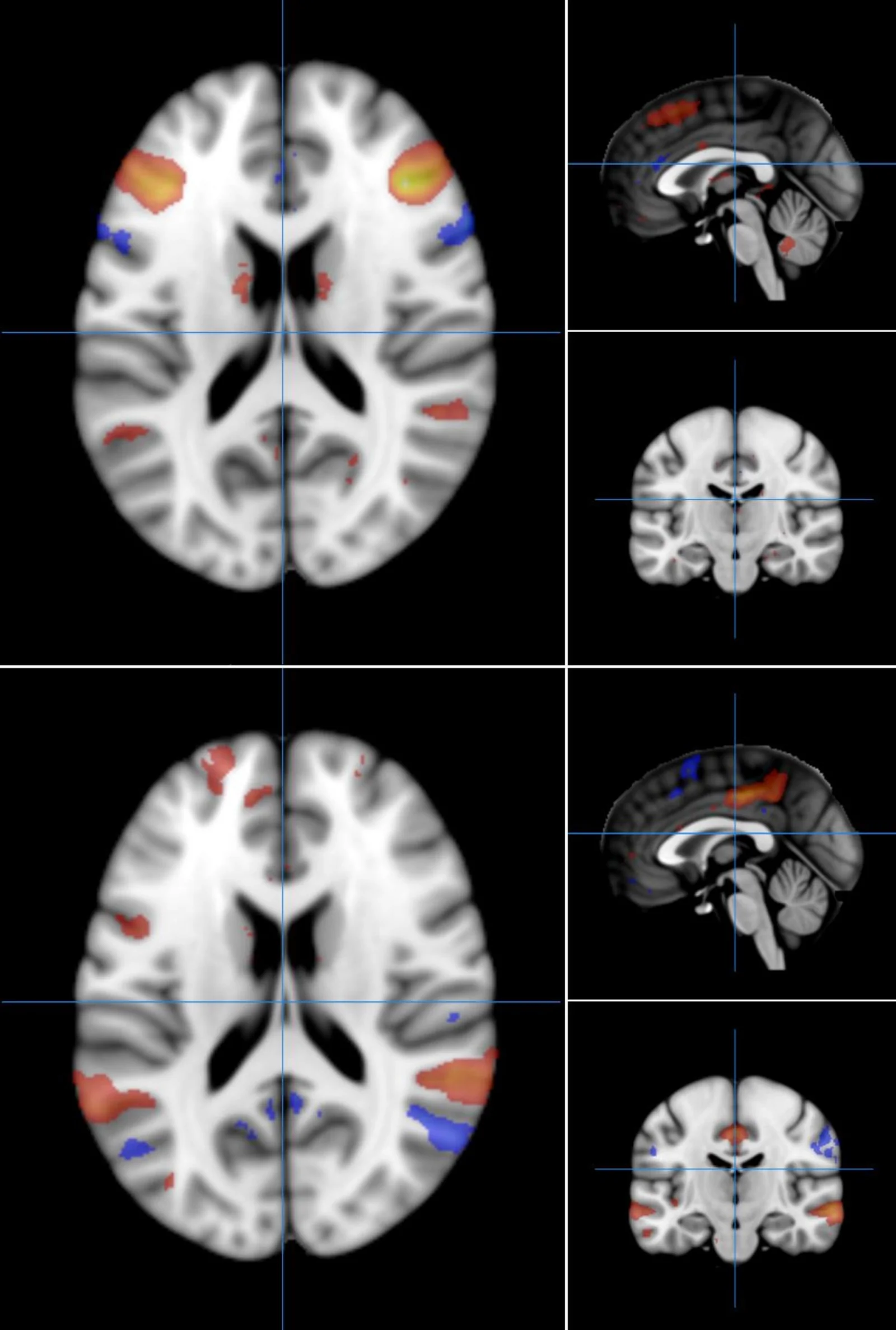
**Graphical representation of brain imaging-derived phenotype (IDP) 2831.** This reflects the edge 403 between nodes 25 (upper panel) and 29 (lower panel) of dimensionality 100 identified by spatial Independent Component Analysis (ICA) of resting-state functional MRI across three anatomical planes: axial (left), sagittal (top right of each panel), and coronal (bottom right of each panel). Red overlay represents positive values, while negative values are indicated with blue overlay.

### Latent causal variable analysis

After applying Bonferroni correction accounting for the number of tests performed (*N* = 23, *P* < 0.002), the LCV analysis uncovered statistically significant genetic causal proportions, highlighting a potential effect of the genetic liability to COVID-19 outcomes on brain IDPs (gcp > 0, Supplemental Table 4). Specifically, the area of pars opercularis in the right hemisphere (IDP 766) was inversely affected by the genetic liability to both COVID-19 hospitalization (gcp = 0.84, *P* = 2.89 × 10^−11^; rho = −0.19 ± 0.07) and SARS-CoV-2 infection (gcp = 0.38, *P* = 1.19 × 10^−11^; rho = −0.22 ± 0.09). Although the statistical strength was much more limited, SARS-CoV-2 infection also showed a significant genetic causal proportion with respect to reduced area of the opercular part of the inferior frontal gyrus in the right hemisphere (IDP 957 gcp = 0.66, *P* = 0.001; rho = −0.29 ± 0.09).

### Locus-specific pleiotropy

Considering the 11 brain IDPs identified by the global genetic correlation analysis, we uncovered five FDR-significant genomic regions (FDR *q* < 0.1) with locus-specific pleiotropy related to COVID-19 hospitalization and SARS-CoV-2 infection (Table 1). Of these, two survived a stricter Bonferroni correction accounting for the number of local genetic correlations tested (*N* = 22,482, *P* < 2.22 × 10^−6^). Specifically, locus ‘3:181 424 324–183 203 155’ showed Bonferroni-significant genetic correlation of COVID-19 hospitalization with two brain IDPs related to parahippocampal cortical thickness (IDP 1069, 1160; rho > 0.8, *P* < 2.4 × 10^−7^). These IDPs also showed FDR-significant local genetic correlation with COVID-19 hospitalization in locus ‘10:129 831 970–130 694 008’ (IDP 1069 rho = 0.88; IDP 1160 rho = 0.83). In locus ‘3:181 424 324–183 203 155’, IDP 1115 related to entorhinal cortical thickness also showed FDR-significant local genetic correlation with COVID-19 hospitalization (rho = 0.63). In locus ‘22:49 559 208–50 347 996’, COVID-19 hospitalization was genetically correlated with two IDPs related to the cortical areas of the pars opercularis (IDPs 766 and 957; rho = −0.97, FDR *q* < 0.1). The sign of these local genetic correlations was in line with the ones observed in the global genetic correlation analysis (positive rg/rho with respect to cortical thickness and negative rg/rho with respect to cortical areas). Conversely, while rfMRI connectivity ICA100 edge 406 (IDP 2834) showed a positive global genetic correlation with SARS-CoV-2 infection, we identified ‘2:84 395 900–85 992 794’ locus with local genetic correlation consistent with the one observed globally (rho = 1, FDR q < 0.1) and ‘20:51 533 751–52 411 532’ locus with inverse local genetic correlation (rho = −1, FDR *q* < 0.1). The local genetic correlations observed are supported by the adequate coverage of genetic variants (*N* > 400) and related principal components (*N* ≥ 140) and by the trends observed in the nominally significant findings within the same chromosomal regions (Supplemental Table 5).

**Table 1:** Local genetic correlations (rho) between COVID-19 outcomes and brain IDP surviving false discovery rate multiple testing correction

Locus	SNPs, *n*	PCs, *n*	COVID-19	Brain IDP	rho	*P*	Protein-Coding Genes
22:49 559 208–50 347 996	464	175	Hospitalization	766	−0.97	1.10E−05	*BRD1, CRELD2, ALG12, ZBED4*
				957	−0.97	1.01E−05	
3:181 424 324–183 203 155	519	176	Hospitalization	1069	0.85	1.46E−07	*SOX2, ATP11B, DCUN1D1, MCCC1, LAMP3, MCF2L2, B3GNT5*
				1115	0.63	1.91E−05	
				1160	0.83	2.38E−07	
10:129 831 970–130 694 008	482	186	Hospitalization	1160	0.83	1.77E−05	*PTPRE, MKI67*
				1069	0.88	2.19E−05	
20:51 533 751–52 411 532	424	176	Infection	2834	−1	1.97E−05	*TSHZ2, ZNF217*
2:84 395 900–85 992 794	545	140	Infection		1	2.12E−05	*SUCLG1, DNAH6, TRABD2A, TMSB10, KCMF1, TCF7L1, TGOLN2, RETSAT, ELMOD3, CAPG, SH2D6, MAT2A, GGCX, VAMP8, VAMP5, RNF181, TMEM150A, USP39, C2orf68, SFTPB, GNLY, ATOH8*

IDP 766: Area of parsopercularis in the right hemisphere generated by parcellation of the pial surface using Desikan-Killiany parcellation; IDP 910: Area of Lat-Fis-ant-Horizont in the left hemisphere generated by parcellation of the white surface using Destrieux (a2009s) parcellation; IDP 957: Area of G-front-inf-Opercular in the right hemisphere generated by parcellation of the white surface using Destrieux (a2009s) parcellation; IDP 1069: Mean thickness of parahippocampal in the right hemisphere generated by parcellation of the white surface using Desikan-Killiany parcellation; IDP 1115: Mean thickness of entorhinal in the right hemisphere generated by parcellation of the white surface using BA_exvivo parcellation; IDP 1160: Mean thickness of parahippocampal in the right hemisphere generated by parcellation of the white surface using DKT parcellation; IDP 1274: Mean thickness of G-oc-temp-med-Parahip in the right hemisphere generated by parcellation of the white surface using Destrieux (a2009s) parcellation; IDP 1985: Mean OD (orientation dispersion index) in medial lemniscus (right) on FA (fractional anisotropy) skeleton (from dMRI data); IDP 2195: rfMRI amplitudes (ICA100 node 32); IDP 2831: rfMRI connectivity (ICA100 edge 403); IDP 2834: rfMRI connectivity (ICA100 edge 406).

To further investigate locus-specific pleiotropy between COVID-19 outcomes and brain IDPs, we conducted a colocalization analysis, identifying three loci related to COVID-19 hospitalization (Supplemental Table 6). Specifically, rs79724577 (PPFC = 0.84, P_SNP_ = 1) and rs56328224 (PPFC = 0.83, P_SNP_ = 0.99) located on chromosome 17 (linkage disequilibrium r^2^ = 0.70) were related to both COVID-19 hospitalization and area of the opercular part of the inferior frontal gyrus in the right hemisphere (IDP 957). Rs13107325 (PPFC = 0.96, P_SNP_ = 0.66) showed shared effect on COVID-19 hospitalization and mean thickness of entorhinal in the right hemisphere (IDP 1115). We also observed colocalization between COVID-19 outcomes and brain-specific transcriptomic regulation (Supplemental Table 6). Specifically, COVID-19 hospitalization showed loci colocalized with transcriptomic regulation of *IL6R* (brain cortex, rs41264915 PPFC = 0.89, P_SNP_ = 0.72), *GBA1* (nucleus accumbens basal ganglia, rs41264915 PPFC = 0.83, P_SNP_ = 0.74), *HLA-J* (hypothalamus, rs9260078 PPFC = 0.86, P_SNP_ = 0.63; frontal cortex (BA9), rs2517723 PPFC = 0.86, P_SNP_ = 0.52; hippocampus, rs2517723 PPFC = 0.76, P_SNP_ = 0.510), *ACSM1* (cerebellar hemisphere, rs1250991 PPFC = 0.73, P_SNP_ = 0.54), *ENSG00000270055* (nucleus accumbens basal ganglia, rs72714666 PPFC = 0.86, P_SNP_ = 0.96; hypothalamus, rs72714666 PPFC = 0.84, P_SNP_ = 0.95), and *ACSF3* (cerebellar hemisphere, rs117169628 PPFC = 0.81, P_SNP_ = 0.965). Loci associated with SARS-CoV-2 infection colocalized with transcriptomic regulation of *ENSG00000272501* (caudate basal ganglia, rs1128175 PPFC = 0.76, P_SNP_ = 0.94), *HLA-C* (caudate basal ganglia, rs2245731 PPFC = 0.82, P_SNP_ = 0.96), *AZGP1* (caudate basal ganglia, rs13243708 PPFC = 0.72, P_SNP_ = 0.86), and *ZGLP1* (cerebellar hemisphere, rs142770866 PPFC = 0.86, P_SNP_ = 0.69).

### Gene-set and drug-repurposing analyses

After Bonferroni correction accounting for the number of GO terms tested (*N* = 79,239, *P* < 6.31 × 10^−7^) in the GSA-MiXer analysis, we identified 305 GOs that were statistically enriched across the three COVID-19 outcomes tested and the 11 brain IDPs identified (Supplemental Table 7). Comparing GSA-MiXeR statistics, COVID-19 hospitalization and SARS-CoV-2 infection showed a 10–15% increased enrichment for the shared GOs compared with COVID-19 severe respiratory symptoms and the 11 brain IDPs (Fig. 5; Supplemental Table 7). Through REVIGO approach, we identified 137 non-redundant Bonferroni-significant GOs related to biological processes (Supplemental Table 8). Of these, 109 were classified into six representative domains: cell projection organization (*N* = 13), cellular response to stress (*N* = 17), peptidyl-amino acid modification (*N* = 20), regulation of intracellular signal transduction (*N* = 30), tissue development (*N* = 18), and vesicle-mediated transport (*N* = 11). We observed neuroimmune convergence in both ‘tissue development’ and ‘regulation of intracellular signal transduction’ domains. Specifically, ‘tissue development’ included GOs such as immune system development (GO:0002520), leukocyte differentiation (GO:0002521), lymphocyte activation (GO:0046649), neurogenesis (GO:0022008), nervous system process (GO:0050877), and head development (GO:0060322). ‘Regulation of intracellular signal transduction’ domain included synaptic signalling (GO:0099536), regulation of immune system process (GO:0002682), and immune response (GO:0002764). Testing differences in non-redundant GO term enrichments among COVID-19 outcomes, we observed only nominally significant differences (*P* < 0.05) between COVID-19 hospitalization and COVID-19 respiratory symptoms (*N* = 23) and between SARS-CoV-2 infection and COVID-19 respiratory symptoms (*N* = 16), but not between COVID-19 hospitalization and SARS-CoV-2 infection (*P* > 0.05, Supplemental Table 8).

**Figure 5:**
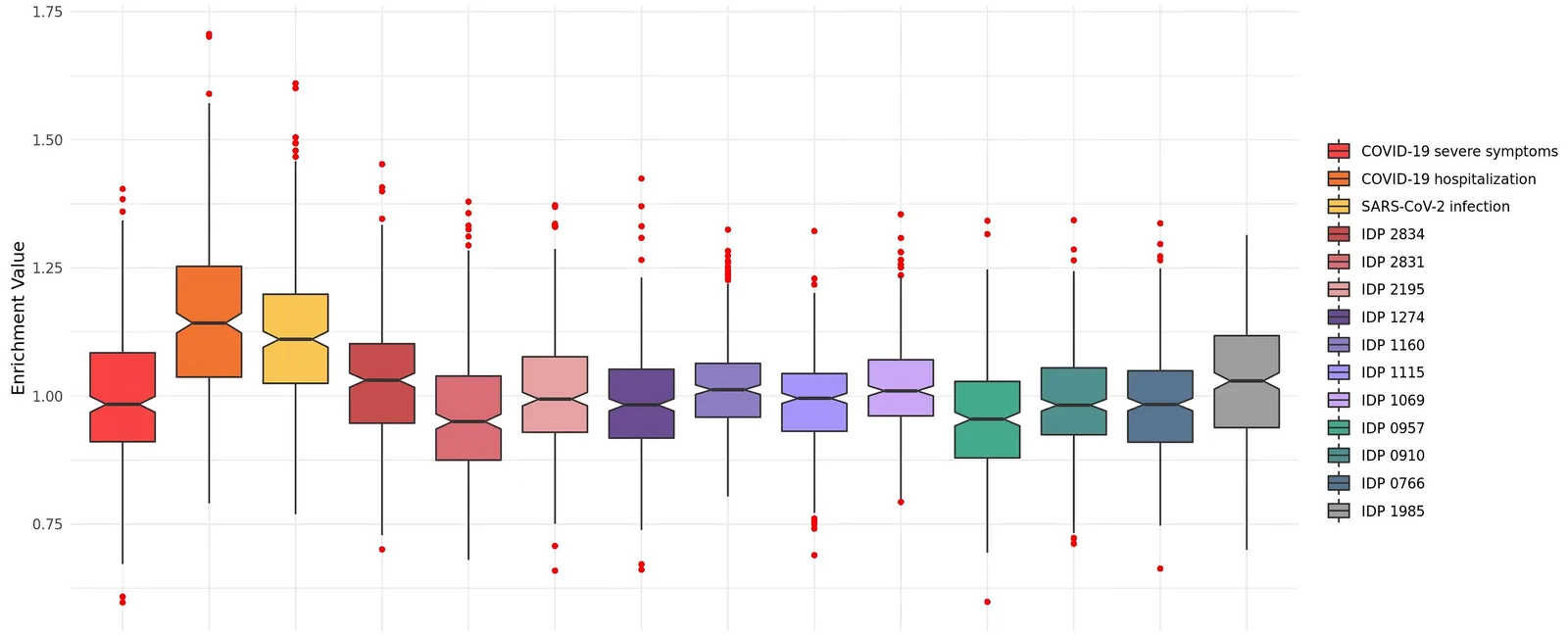
**Enrichment distribution of the 305 Bonferroni-significant GO shared among COVID-19 outcomes (severe respiratory symptoms, 13 769 cases and 1 072 442 controls; hospitalization 32 519 cases and 2 062 805 controls; infection, 122 616 cases and 2 475 240 controls) and the 11 brain IDP (IDP; assessed in 33 224 participants).** Statistical significance was estimated using a block jackknife-based test. Full results are available in Supplemental Table 7. Data points outside the box plots represent enrichment estimates for GO outside the distribution main range.

Considering FDR-significant GO enrichments shared across the three COVID-19 outcomes tested and the 11 brain IDPs identified, our drug-repurposing analysis identified 41 compounds (Supplemental Table 9). Fourteen of them were associated with downregulation of the pathways tested (enrichment score, ES < 0). These included antiviral drugs (e.g. ribavirin ES = −0.519, *P* = 4.15 × 10^−4^; top pathway: kinase activity), heart medications (e.g. canadine ES = −0.504, *P* = 6.68 × 10^−4^; top pathway: phosphoric ester hydrolase activity), chemotherapy medications (decitabine ES = −0.5, *P* = 7.67 × 10^−4^; top pathway: ion transmembrane transporter activity), antihypertensive agents (epivincamine ES = −0.468, *P* = 4.25 × 10^−4^; top pathway: nucleolus), antibiotics (e.g. ceforanide ES = −0.207, *P* = 2.4 × 10^−4^; top-pathway: immune response), and antihistaminic medications (levocabastine ES = −0.195, *P* = 6.47 × 10^−4^; top-pathway: mRNA metabolic process). While cardiovascular, anticancer, anti-infective, and antibacterial drugs were also among compounds associated with upregulated pathways (ES > 0), these also included antipsychotic medications (clozapine ES = 0.209, *P* = 2.02 × 10^−4^, top pathway: embryo development; chlorprothixene ES = 0.187, *P* = 0.001, top pathway: mRNA metabolic process) and antidepressants (nortriptyline ES = 0.196, *P* = 6.09 × 10^−4^, top pathway: intracellular protein transport).

## Discussion

Expanding on evidence generated by previous studies,^3-9^ our investigation highlighted that while the pleiotropy between COVID-19 outcomes and brain variation is limited due to the distinct nature of the related biological systems, these domains are connected through multiple shared genetic mechanisms. Indeed, while we found modest global genetic correlation, there was evidence of shared variants (polygenic overlap), possible directional effects (vertical pleiotropy), local genetic correlation (regional pleiotropy), and individual loci with shared effect (colocalization).

With respect to rfMRI-generated IDPs, we observed different patterns in line with previous studies that reported that COVID-19 outcomes can alter brain activity, increasing or decreasing functional connectivity depending on the brain regions assessed.^4,41,42^ In our study, the connectivity network among Broca’s area, dlPFC, anterior prefrontal cortex, posterior cingulate cortex, caudate, and left dorsal anterior cingulate cortex (IDP 2834, rfMRI connectivity ICA100 edge 406; Fig. 2) and the brain activity related to the right supramarginal gyrus, primary somatosensory cortex, and motor cortex (IDP 2195, rfMRI amplitudes ICA100 node 32; Fig. 3) showed positive genetic correlation with COVID-19 outcomes. Conversely, IDP 2831 (rfMRI connectivity ICA100 edge 403; Fig. 4) had an inverse genetic correlation with COVID-19 outcomes. Interestingly, the fraction of loci shared by these rfMRI-generated IDPs with COVID-19 hospitalization and SARS-CoV-2 infection was higher than the one observed with respect to COVID-19 respiratory symptoms (Supplemental Table 3). With respect to the brain regions underlying the rfMRI-generated IDPs identified in the present study, previous evidence supports their association with COVID-19 neurologic signs.^43,44^ For instance, dlPFC has been associated with cognitive deficits in young adults infected by SARS-CoV-2.^43^ In comparison with healthy controls, COVID-19 patients showed decreased regional homogeneity in the left angular gyrus, which was also negatively correlated with the severity of depression symptoms.^44^ Altered connectivity in the anterior cingulate gyrus and the left posterior cingulate gyrus has been reported in patients with post-COVID-19 syndrome.^41^ Additionally, tissue damage in the anterior cingulate cortex has been reported in mild COVID-19 cases,^45^ while the high expression of angiotensin-converting enzyme 2 (ACE2) in excitatory and inhibitory neurons and in non-neuronal cells in the posterior cingulate cortex suggests the vulnerability of this brain region to COVID-19 pathogenetic processes.^46^ With respect to Broca’s area, a reduction in the grey matter volume has been reported in COVID-19 patients.^47^ This may contribute to COVID-19 relationship with aphasia-like symptoms.^48^ These data show a potentially direct interplay between COVID-19 pathogenesis and altered neural connectivity that could have a key role in the neuropsychiatric symptoms observed in a subset of COVID-19 patients.

With respect to IDP 2834 (rfMRI connectivity ICA100 edge 406; Fig. 2), we also observed evidence of locus-specific pleiotropy, with two chromosome regions (i.e. ‘2:84 395 900–85 992 794’; ‘20:51 533 751–52 411 532’) showing local genetic correlation with SARS-CoV-2 infection. Importantly, both of these were complete local genetic correlations between IDP 2834 and SARS-CoV-2 infection, which showed opposite directions (rho = +1 in ‘2:84 395 900–85 992 794’; rho = −1 in ‘20:51 533 751–52 411 532’). The reliability of the estimate strength was supported by the coverage of these regions and the consistency of the trends observed among the nominally significant local genetic correlations observed within these regions (Supplemental Table 5). Accordingly, the different direction observed is likely due to the function of the genes underlying the local genetic correlation observed. In locus ‘20:51 533 751–52 411 532’, *TSHZ2* has been implicated in brain development, and it was one of the most significantly hypermethylated genes in COVID-19 patients six weeks after infection.^49^ In locus ‘2:84 395 900–85 992 794’, there are multiple genes involved in COVID-19 pathogenesis. *USP39* encodes an essential component for SARS-CoV-2 viral assembly and replication.^50^  *GGCX* is known to interact directly with viral proteins and has been suggested as a potential target for COVID-19 drug development.^51^

With respect to cortical thickness, our findings highlight a positive genetic correlation with COVID-19 outcomes in parahippocampal and entorhinal gyri, which also showed >40% of variants shared with COVID-19 hospitalization. Increased cortical thickness in the parahippocampal gyrus has also been reported (i) in healthy COVID-19 recovered patients compared with non-infected controls and (ii) in long-COVID-19 patients compared with healthy COVID-19 recovered patients.^15^ Further evidence of pleiotropy between COVID-19 outcomes and parahippocampal and entorhinal gyri has been highlighted by our locus-specific analyses. In region ‘3:181 424 324–183 203 155’, COVID-19 hospitalization was genetically correlated with the cortical thickness of parahippocampal and entorhinal gyri (IDPs 1069, 1115, and 1160). Among genes mapping within this region, *LAMP3* gene encodes an immune-related protein that was upregulated in hospitalized COVID-19 patients compared with non-hospitalized individuals.^52^ Another gene in this locus is *SOX2*, which plays an important role in brain development and cortical layer formation^53^ and has been used to assess COVID-19 infection on the olfactory cell lineage.^54^ Also mapping within locus ‘3:181 424 324–183 203 155’, *ATP11B* and *B3GNT5* showed altered regulation during SARS-CoV-2 infection, and variants in these genes have been associated with hippocampal synaptic plasticity impairment.^55^ We also observed locus-specific pleiotropy differentially linking COVID-19 outcomes to parahippocampal and entorhinal gyri. Specifically, locus ‘10: 129 831 970–130 694 008’ showed genetic correlation between COVID-19 hospitalization and parahippocampal cortical thickness (IDPs 1160 and 1069). In this region, *MKI67* encodes a protein involved in immune response to COVID-19,^56^ while the *PTPRE* was observed to be upregulated during SARS-CoV-2 infection.^57^ Conversely, our colocalization analysis highlights rs13107325 as a variant shared between COVID-19 hospitalization and entorhinal thickness in the right hemisphere (IDP 1115). This is a *SLC39A8* missense variant previously associated with many phenotypes, such as neuroimaging measures,^58^ blood-based biomarkers,^59^ cardiovascular conditions,^60^ and metabolic disorders.^61^ The role of *SLC39A8* protein product plays a key role in zinc and manganese cellular transport,^62^ which has been reported in the context of both brain development^63^ and COVID-19 pathogenesis.^64^ These results provide further evidence that genetic mechanisms influencing COVID-19 susceptibility disrupt brain regulation, specifically via altered developmental pathways.

Differently from cortical-thickness IDPs, COVID-19 outcomes showed an inverse genetic correlation with the cortical areas of the Lat-Fis-ant-Horizont area and the pars opercularis, which are involved in language production and syntax processing.^65^ Interestingly, our LCV analysis also highlighted a potential effect of COVID-19 hospitalization and SARS-CoV-2 infection on reducing the area of pars opercularis in the right hemisphere. Significant differences in the grey matter volume of the pars opercularis have been reported in acutely ill COVID-19 patients^47^ and in COVID-19 recovered patients at 1 year after discharge.^66^ Additionally, decreased structural metrics of the pars opercularis were also associated with sleep disturbances in COVID-19 patients.^67^ We also identified evidence of locus-specific pleiotropy between COVID-19 hospitalization and pars opercularis (IDPs 766 and 957). A local genetic correlation was observed in region ‘22:49 559 208–50 347 996’, where *BRD1* has been implicated in humoral immune response activation.^68^ Additionally, rs79724577 and rs56328224 showed colocalization between COVID-19 hospitalization and the area of the opercular part of the inferior frontal gyrus in the right hemisphere (IDP 957). These variants are located in the same region on chromosome 17 and have been associated with pulmonary function^69^ and cognitive measurements^70^ in addition to neuroimaging phenotypes^21,71^ and COVID-19 outcomes.^72^ Consistent with growing data on the immune-lung-brain axis,^73^ these findings suggest a pathophysiological mechanism driving the neurological manifestations of COVID-19 and other respiratory infections.

Further support for the pleiotropy between COVID-19 outcomes and brain function was observed in our colocalization analysis with the identification of pleiotropic variants linking COVID-19 hospitalization and SARS-CoV-2 infection to brain-specific transcriptomic regulation. In particular, COVID-19 hospitalization colocalized with variants regulating genes playing important roles in brain biology. For example, *IL6R* has been implicated in signalling pathways linking glial cells to neuroinflammatory and neurodegenerative processes.^74^  *GBA1* altered function has been linked to neuronal damage associated with neurodegenerative processes.^75^ SARS-CoV-2 infection colocalized variants regulating genes in caudate basal ganglia and cerebellar hemisphere. Among the colocalized loci, *AZGP1* appears to regulate energy homeostasis and glucose/lipid metabolism in pro-opiomelanocortin neurons,^76^ while *HLA-C* has been included among immune genes associated with neuroimaging traits as well as the development of neuropsychiatric disorders.^77^

Moving from individual loci to biological processes, cellular components, and molecular functions, we identified 305 GOs that reached Bonferroni significance with respect to the three COVID-19 outcomes investigated and the 11 brain IDPs identified. Based on their semantic similarities, we identified 137 non-redundant terms, and 109 of them were related to six domains reflecting broad biological dynamics. Among them, ‘tissue development’ and ‘regulation of intracellular signal transduction’ showed neuroimmune convergence. The GO term ‘tissue development’ (GO:0009888) was the most significant for COVID-19 hospitalization, IDP 2831, and IDP 2195, and other GO terms linked to this domain were related to the activation of immune system (i.e. immune system development, leukocyte differentiation, lymphocyte activation) and neurodevelopment (i.e. neurogenesis, nervous system process, head development). These findings appear to reflect the pleiotropy underlying crosstalk mechanisms between immune and central nervous systems that contribute to developmental processes.^78,79^ This convergence may also extend to ‘regulation of intracellular signal transduction’ domain, which included synaptic signalling (GO:0099536), regulation of immune system process (GO:0002682), and immune response (GO:0002764). An increasing number of studies reported various signalling mechanisms linking immune and central nervous systems, also highlighting their impact on health and disease.^80,81^ Our results suggest that these interconnected pathways can also contribute to COVID-19 neuropsychiatric comorbidities.

Our drug-repurposing analyses identified 41 compounds with transcriptomic profiles matching the genes related to the GOs shared between COVID-19 outcomes and brain IDPs. In line with the systemic nature of the pleiotropic mechanisms linking COVID-19 outcomes to brain function and structure, we identified drugs used with respect to a range of clinical domains (e.g. cardiovascular diseases, cancer, and painful conditions) in addition to immune and neuropsychiatric conditions. Drugs related to other domains were associated with upregulation and downregulation of the pathways identified. For instance, the downregulation of the kinase activity pathway in the context of ribavirin is in line with the antiviral activity of this compound.^82^ However, this downregulation may lead to altered regulation of cellular signalling and synaptic plasticity.^83^ With respect to the psychiatry-related compounds, gene2drug approach highlighted transcriptomic upregulation. These included antipsychotics (i.e. clozapine and chlorprothixene) and antidepressants (i.e. nortriptyline). Notably, clozapine and nortriptyline have been associated with worse COVID-19 outcomes because of their immunosuppressive effect.^84,85^ The top pathways related to these drugs were related to broad molecular functions (nortriptyline top pathway: intracellular protein transport; clozapine top pathway: embryo development), suggesting that their harmful effects may be driven by pathogenic processes acting through wide-ranging dynamics. Another antipsychotic medication, chlorpromazine, appears to exert antiviral and immunomodulatory activity, inhibiting the expression and secretion of IL-6 by monocytes activated by SARS-CoV-2.^86^ Additionally, in preclinical studies, chlorpromazine showed protection against SARS-CoV-2, possibly through antiviral activity.^87^ However, an observational study in adult inpatients hospitalized outside ICUs for COVID-19 showed that chlorpromazine was not associated with reduced mortality.^88^ Nevertheless, considering that mRNA metabolic process was the top pathway upregulated with respect to chlorpromazine, this compound may specifically act on the neurological forms of COVID-19^89^ through transcriptional remodelling.

Our study has several limitations that should be acknowledged. Due to the lack of large-scale GWAS in diverse populations, we investigated only data generated from populations of European descent. Therefore, our findings may not be generalizable to other population groups. In this context, because the brain IDP GWAS was based only on UKB cohort, the findings observed may reflect dynamics specific to the characteristics of this sample. Given the exploratory character of the present investigation, our initial assessment of COVID-19 genetic correlation did not account for the number of brain IDPs tested. Because of the limited sample size of the brain IDP GWAS, we only investigated GO terms and molecular compounds related to genetic mechanisms shared between brain IDPs and different COVID-19 outcomes. Future investigation based on more powerful datasets will be needed to assess molecular mechanisms underlying the pleiotropic patterns linking brain variation to different COVID-19 outcomes. Additionally, we identified shared genetic mechanisms considering the overlap of GSA-MiXer univariate model results due to the fact that the code of GSA-MiXer bivariate model is publicly available at this time.^90^ Finally, our study only assessed the genetic mechanisms shared between COVID-19 susceptibility and the predisposition to brain variation and did not take into account extrinsic factors such as exposure to the virus and to the temporal context of the pandemic. Longitudinal investigations will be needed to assess the interplay between genetic and environmental factors with respect to COVID-19 impact on brain variation.

In summary, this study provides novel insights into the genetic architecture shared between COVID-19 outcomes and brain variation. By leveraging large-scale genome-wide data and integrative analytical frameworks, we uncovered how neuroimmune pleiotropy affecting multiple biological processes could potentially contribute to COVID-19 impact on brain-related phenotypes. Overall, our findings strongly support the need to investigate COVID-19 outcomes and brain biology through studies assessing the interplay between central and peripheral systems.

## Supplementary Material

fcag372_Supplementary_Data

## Data Availability

COVID-19 genome-wide association statistics are available at https://www.covid19hg.org/results/r7/. Genome-wide association statistics of brain IDP are available at https://open.win.ox.ac.uk/ukbiobank/big40/. The data generated from the present study are included in the manuscript and its supplemental material. Analytic tools used in the present study are available through the following links: Linkage Disequilibrium Score Regression, https://github.com/bulik/ldsc; Latent Causal Variable Analysis, https://github.com/lukejoconnor/LCV; HyPrColoc, https://github.com/cnfoley/hyprcoloc; MiXer, https://github.com/precimed/mixer; GSA-MiXer, https://github.com/precimed/gsa-mixer; REVIGO, http://revigo.irb.hr/; gene2drug, https://github.com/franapoli/gep2pep.
