# Supplementary material for: COVID-19 pleiotropy with brain imaging-derived phenotypes highlights novel insights into the biology shared between immune function and brain variation
Source: Brain Commun. 2026 Oct 5;8(5):fcag372. doi: 10.1093/braincomms/fcag372 (PMC13669621; doi:10.1093/braincomms/fcag372)
Supplement: Supplementary file 1 [file fcag372_supplementary_data.pdf]

Supplemental Table 1: Description of the brain imaging-derived phenotypes (IDP) assessed in 33,224 UK Biobank participants.

IDP number	IDP short name	IDP description
0001	IDP T1 SIENAX peripheral grey normalised volume	Volume of peripheral cortical grey matter (from T1 brain image, normalised for head size)
0002	IDP T1 SIENAX peripheral grey unnormalised volume	Volume of peripheral cortical grey matter (from T1 brain image)
0003	IDP T1 SIENAX CSF normalised volume	Volume of ventricular cerebrospinal fluid (from T1 brain image, normalised for head size)
0004	IDP T1 SIENAX CSF unnormalised volume	Volume of ventricular cerebrospinal fluid (from T1 brain image)
0005	IDP T1 SIENAX grey normalised volume	Volume of grey matter (from T1 brain image, normalised for head size)
0006	IDP T1 SIENAX grey unnormalised volume	Volume of grey matter (from T1 brain image)
0007	IDP T1 SIENAX white normalised volume	Volume of white matter (from T1 brain image, normalised for head size)
0008	IDP T1 SIENAX white unnormalised volume	Volume of white matter (from T1 brain image)
0009	IDP T1 SIENAX brain-normalised volume	Volume of brain, grey+white, from T1 brain image, normalised for head size
0010	IDP T1 SIENAX brain-unnormalised volume	Volume of brain, grey+white, from T1 brain image
0011	IDP T1 FIRST left thalamus volume	Volume of left thalamus (from T1 brain image)
0012	IDP T1 FIRST right thalamus volume	Volume of right thalamus (from T1 brain image)
0013	IDP T1 FIRST left caudate volume	Volume of left caudate (from T1 brain image)
0014	IDP T1 FIRST right caudate volume	Volume of right caudate (from T1 brain image)
0015	IDP T1 FIRST left putamen volume	Volume of left putamen (from T1 brain image)
0016	IDP T1 FIRST right putamen volume	Volume of right putamen (from T1 brain image)
0017	IDP T1 FIRST left pallidum volume	Volume of left pallidum (from T1 brain image)
0018	IDP T1 FIRST right pallidum volume	Volume of right pallidum (from T1 brain image)
0019	IDP T1 FIRST left hippocampus volume	Volume of left hippocampus (from T1 brain image)
0020	IDP T1 FIRST right hippocampus volume	Volume of right hippocampus (from T1 brain image)
0021	IDP T1 FIRST left amygdala volume	Volume of left amygdala (from T1 brain image)
0022	IDP T1 FIRST right amygdala volume	Volume of right amygdala (from T1 brain image)
0023	IDP T1 FIRST left accumbens volume	Volume of left accumbens (from T1 brain image)
0024	IDP T1 FIRST right accumbens volume	Volume of right accumbens (from T1 brain image)
0025	IDP T1 FIRST brain stem+4th ventricle volume	Volume of brain stem + 4th ventricle (from T1 brain image)
0026	IDP T1 FAST ROIs L frontal pole	Volume of grey matter in Left Frontal Pole
0027	IDP T1 FAST ROIs R frontal pole	Volume of grey matter in Right Frontal Pole
0028	IDP T1 FAST ROIs L insular cortex	Volume of grey matter in Left Insular Cortex
0029	IDP T1 FAST ROIs R insular cortex	Volume of grey matter in Right Insular Cortex
0030	IDP T1 FAST ROIs L sup front gyrus	Volume of grey matter in Left Superior Frontal Gyrus
0031	IDP T1 FAST ROIs R sup front gyrus	Volume of grey matter in Right Superior Frontal Gyrus
0032	IDP T1 FAST ROIs L mid front gyrus	Volume of grey matter in Left Middle Frontal Gyrus
0033	IDP T1 FAST ROIs R mid front gyrus	Volume of grey matter in Right Middle Frontal Gyrus
0034	IDP T1 FAST ROIs L inf front gyrus parstri	Volume of grey matter in Left Inferior Frontal Gyrus, pars triangularis
0035	IDP T1 FAST ROIs R inf front gyrus parstri	Volume of grey matter in Right Inferior Frontal Gyrus, pars triangularis
0036	IDP T1 FAST ROIs L inf front gyrus parsop	Volume of grey matter in Left Inferior Frontal Gyrus, pars opercularis
0037	IDP T1 FAST ROIs R inf front gyrus parsop	Volume of grey matter in Right Inferior Frontal Gyrus, pars opercularis
0038	IDP T1 FAST ROIs L precentral gyrus	Volume of grey matter in Left Precentral Gyrus
0039	IDP T1 FAST ROIs R precentral gyrus	Volume of grey matter in Right Precentral Gyrus
0040	IDP T1 FAST ROIs L temporal pole	Volume of grey matter in Left Temporal Pole
0041	IDP T1 FAST ROIs R temporal pole	Volume of grey matter in Right Temporal Pole
0042	IDP T1 FAST ROIs L sup temp gyrus ant	Volume of grey matter in Left Superior Temporal Gyrus, anterior division
0043	IDP T1 FAST ROIs R sup temp gyrus ant	Volume of grey matter in Right Superior Temporal Gyrus, anterior division
0044	IDP T1 FAST ROIs L sup temp gyrus post	Volume of grey matter in Left Superior Temporal Gyrus, posterior division
0045	IDP T1 FAST ROIs R sup temp gyrus post	Volume of grey matter in Right Superior Temporal Gyrus, posterior division
0046	IDP T1 FAST ROIs L mid temp gyrus ant	Volume of grey matter in Left Middle Temporal Gyrus, anterior division
0047	IDP T1 FAST ROIs R mid temp gyrus ant	Volume of grey matter in Right Middle Temporal Gyrus, anterior division
0048	IDP T1 FAST ROIs L mid temp gyrus post	Volume of grey matter in Left Middle Temporal Gyrus, posterior division
0049	IDP T1 FAST ROIs R mid temp gyrus post	Volume of grey matter in Right Middle Temporal Gyrus, posterior division
0050	IDP T1 FAST ROIs L mid temp gyrus tempocc	Volume of grey matter in Left Middle Temporal Gyrus, temporooccipital part
0051	IDP T1 FAST ROIs R mid temp gyrus tempocc	Volume of grey matter in Right Middle Temporal Gyrus, temporooccipital part
0052	IDP T1 FAST ROIs L inf temp gyrus ant	Volume of grey matter in Left Inferior Temporal Gyrus, anterior division
0053	IDP T1 FAST ROIs R inf temp gyrus ant	Volume of grey matter in Right Inferior Temporal Gyrus, anterior division
0054	IDP T1 FAST ROIs L inf temp gyrus post	Volume of grey matter in Left Inferior Temporal Gyrus, posterior division
0055	IDP T1 FAST ROIs R inf temp gyrus post	Volume of grey matter in Right Inferior Temporal Gyrus, posterior division

IDP number	IDP short name	IDP description
0056	IDP T1 FAST ROIs L inf temp gyrus tempocc	Volume of grey matter in Left Inferior Temporal Gyrus, temporooccipital part
0057	IDP T1 FAST ROIs R inf temp gyrus tempocc	Volume of grey matter in Right Inferior Temporal Gyrus, temporooccipital part
0058	IDP T1 FAST ROIs L postcent gyrus	Volume of grey matter in Left Postcentral Gyrus
0059	IDP T1 FAST ROIs R postcent gyrus	Volume of grey matter in Right Postcentral Gyrus
0060	IDP T1 FAST ROIs L sup parietal lobule	Volume of grey matter in Left Superior Parietal Lobule
0061	IDP T1 FAST ROIs R sup parietal lobule	Volume of grey matter in Right Superior Parietal Lobule
0062	IDP T1 FAST ROIs L supramarg gyrus ant	Volume of grey matter in Left Supramarginal Gyrus, anterior division
0063	IDP T1 FAST ROIs R supramarg gyrus ant	Volume of grey matter in Right Supramarginal Gyrus, anterior division
0064	IDP T1 FAST ROIs L supramarg gyrus post	Volume of grey matter in Left Supramarginal Gyrus, posterior division
0065	IDP T1 FAST ROIs R supramarg gyrus post	Volume of grey matter in Right Supramarginal Gyrus, posterior division
0066	IDP T1 FAST ROIs L angular gyrus	Volume of grey matter in Left Angular Gyrus
0067	IDP T1 FAST ROIs R angular gyrus	Volume of grey matter in Right Angular Gyrus
0068	IDP T1 FAST ROIs L latocc cortex sup	Volume of grey matter in Left Lateral Occipital Cortex, superior division
0069	IDP T1 FAST ROIs R latocc cortex sup	Volume of grey matter in Right Lateral Occipital Cortex, superior division
0070	IDP T1 FAST ROIs L latocc cortex inf	Volume of grey matter in Left Lateral Occipital Cortex, inferior division
0071	IDP T1 FAST ROIs R latocc cortex inf	Volume of grey matter in Right Lateral Occipital Cortex, inferior division
0072	IDP T1 FAST ROIs L intracalc cortex	Volume of grey matter in Left Intracalcarine Cortex
0073	IDP T1 FAST ROIs R intracalc cortex	Volume of grey matter in Right Intracalcarine Cortex
0074	IDP T1 FAST ROIs L front med cortex	Volume of grey matter in Left Frontal Medial Cortex
0075	IDP T1 FAST ROIs R front med cortex	Volume of grey matter in Right Frontal Medial Cortex
0076	IDP T1 FAST ROIs L juxtapos lobule cortex	Volume of grey matter in Left Juxtapositional Lobule Cortex (formerly Supplementary Motor Cortex)
0077	IDP T1 FAST ROIs R juxtapos lobule cortex	Volume of grey matter in Right Juxtapositional Lobule Cortex (formerly Supplementary Motor Cortex)
0078	IDP T1 FAST ROIs L subcallosal cortex	Volume of grey matter in Left Subcallosal Cortex
0079	IDP T1 FAST ROIs R subcallosal cortex	Volume of grey matter in Right Subcallosal Cortex
0080	IDP T1 FAST ROIs L paracing gyrus	Volume of grey matter in Left Paracingulate Gyrus
0081	IDP T1 FAST ROIs R paracing gyrus	Volume of grey matter in Right Paracingulate Gyrus
0082	IDP T1 FAST ROIs L cing gyrus ant	Volume of grey matter in Left Cingulate Gyrus, anterior division
0083	IDP T1 FAST ROIs R cing gyrus ant	Volume of grey matter in Right Cingulate Gyrus, anterior division
0084	IDP T1 FAST ROIs L cing gyrus post	Volume of grey matter in Left Cingulate Gyrus, posterior division
0085	IDP T1 FAST ROIs R cing gyrus post	Volume of grey matter in Right Cingulate Gyrus, posterior division
0086	IDP T1 FAST ROIs L precun cortex	Volume of grey matter in Left Precuneous Cortex
0087	IDP T1 FAST ROIs R precun cortex	Volume of grey matter in Right Precuneous Cortex
0088	IDP T1 FAST ROIs L cuneal cortex	Volume of grey matter in Left Cuneal Cortex
0089	IDP T1 FAST ROIs R cuneal cortex	Volume of grey matter in Right Cuneal Cortex
0090	IDP T1 FAST ROIs L front orb cortex	Volume of grey matter in Left Frontal Orbital Cortex
0091	IDP T1 FAST ROIs R front orb cortex	Volume of grey matter in Right Frontal Orbital Cortex
0092	IDP T1 FAST ROIs L parahipp gyrus ant	Volume of grey matter in Left Parahippocampal Gyrus, anterior division
0093	IDP T1 FAST ROIs R parahipp gyrus ant	Volume of grey matter in Right Parahippocampal Gyrus, anterior division
0094	IDP T1 FAST ROIs L parahipp gyrus post	Volume of grey matter in Left Parahippocampal Gyrus, posterior division
0095	IDP T1 FAST ROIs R parahipp gyrus post	Volume of grey matter in Right Parahippocampal Gyrus, posterior division
0096	IDP T1 FAST ROIs L lingual gyrus	Volume of grey matter in Left Lingual Gyrus
0097	IDP T1 FAST ROIs R lingual gyrus	Volume of grey matter in Right Lingual Gyrus
0098	IDP T1 FAST ROIs L temp fusif cortex ant	Volume of grey matter in Left Temporal Fusiform Cortex, anterior division
0099	IDP T1 FAST ROIs R temp fusif cortex ant	Volume of grey matter in Right Temporal Fusiform Cortex, anterior division
0100	IDP T1 FAST ROIs L temp fusif cortex post	Volume of grey matter in Left Temporal Fusiform Cortex, posterior division
0101	IDP T1 FAST ROIs R temp fusif cortex post	Volume of grey matter in Right Temporal Fusiform Cortex, posterior division
0102	IDP T1 FAST ROIs L temp occ fusif cortex	Volume of grey matter in Left Temporal Occipital Fusiform Cortex
0103	IDP T1 FAST ROIs R temp occ fusif cortex	Volume of grey matter in Right Temporal Occipital Fusiform Cortex
0104	IDP T1 FAST ROIs L occ fusif gyrus	Volume of grey matter in Left Occipital Fusiform Gyrus
0105	IDP T1 FAST ROIs R occ fusif gyrus	Volume of grey matter in Right Occipital Fusiform Gyrus
0106	IDP T1 FAST ROIs L front operc cortex	Volume of grey matter in Left Frontal Operculum Cortex
0107	IDP T1 FAST ROIs R front operc cortex	Volume of grey matter in Right Frontal Operculum Cortex
0108	IDP T1 FAST ROIs L cent operc cortex	Volume of grey matter in Left Central Opercular Cortex
0109	IDP T1 FAST ROIs R cent operc cortex	Volume of grey matter in Right Central Opercular Cortex
0110	IDP T1 FAST ROIs L parietal operc cortex	Volume of grey matter in Left Parietal Operculum Cortex
0111	IDP T1 FAST ROIs R parietal operc cortex	Volume of grey matter in Right Parietal Operculum Cortex
0112	IDP T1 FAST ROIs L planum polare	Volume of grey matter in Left Planum Polare

IDP number	IDP short name	IDP description
0113	IDP T1 FAST ROIs R planum polare	Volume of grey matter in Right Planum Polare
0114	IDP T1 FAST ROIs L heschl gyrus	Volume of grey matter in Left Heschl's Gyrus (includes H1 and H2)
0115	IDP T1 FAST ROIs R heschl gyrus	Volume of grey matter in Right Heschl's Gyrus (includes H1 and H2)
0116	IDP T1 FAST ROIs L planum temporale	Volume of grey matter in Left Planum Temporale
0117	IDP T1 FAST ROIs R planum temporale	Volume of grey matter in Right Planum Temporale
0118	IDP T1 FAST ROIs L supracalc cortex	Volume of grey matter in Left Supracalcarine Cortex
0119	IDP T1 FAST ROIs R supracalc cortex	Volume of grey matter in Right Supracalcarine Cortex
0120	IDP T1 FAST ROIs L occ pole	Volume of grey matter in Left Occipital Pole
0121	IDP T1 FAST ROIs R occ pole	Volume of grey matter in Right Occipital Pole
0122	IDP T1 FAST ROIs L thalamus	Volume of grey matter in Left Thalamus
0123	IDP T1 FAST ROIs R thalamus	Volume of grey matter in Right Thalamus
0124	IDP T1 FAST ROIs L caudate	Volume of grey matter in Left Caudate
0125	IDP T1 FAST ROIs R caudate	Volume of grey matter in Right Caudate
0126	IDP T1 FAST ROIs L putamen	Volume of grey matter in Left Putamen
0127	IDP T1 FAST ROIs R putamen	Volume of grey matter in Right Putamen
0128	IDP T1 FAST ROIs L pallidum	Volume of grey matter in Left Pallidum
0129	IDP T1 FAST ROIs R pallidum	Volume of grey matter in Right Pallidum
0130	IDP T1 FAST ROIs L hippocampus	Volume of grey matter in Left Hippocampus
0131	IDP T1 FAST ROIs R hippocampus	Volume of grey matter in Right Hippocampus
0132	IDP T1 FAST ROIs L amygdala	Volume of grey matter in Left Amygdala
0133	IDP T1 FAST ROIs R amygdala	Volume of grey matter in Right Amygdala
0134	IDP T1 FAST ROIs L ventral striatum	Volume of grey matter in Left Ventral Striatum
0135	IDP T1 FAST ROIs R ventral striatum	Volume of grey matter in Right Ventral Striatum
0136	IDP T1 FAST ROIs brain stem	Volume of grey matter in Brain-Stem
0137	IDP T1 FAST ROIs L cerebellum I-IV	Volume of grey matter in Left I-IV Cerebellum
0138	IDP T1 FAST ROIs R cerebellum I-IV	Volume of grey matter in Right I-IV Cerebellum
0139	IDP T1 FAST ROIs L cerebellum V	Volume of grey matter in Left V Cerebellum
0140	IDP T1 FAST ROIs R cerebellum V	Volume of grey matter in Right V Cerebellum
0141	IDP T1 FAST ROIs L cerebellum VI	Volume of grey matter in Left VI Cerebellum
0142	IDP T1 FAST ROIs V cerebellum VI	Volume of grey matter in Vermis VI Cerebellum
0143	IDP T1 FAST ROIs R cerebellum VI	Volume of grey matter in Right VI Cerebellum
0144	IDP T1 FAST ROIs L cerebellum crus I	Volume of grey matter in Left Crus I Cerebellum
0145	IDP T1 FAST ROIs V cerebellum crus I	Volume of grey matter in Vermis Crus I Cerebellum
0146	IDP T1 FAST ROIs R cerebellum crus I	Volume of grey matter in Right Crus I Cerebellum
0147	IDP T1 FAST ROIs L cerebellum crus II	Volume of grey matter in Left Crus II Cerebellum
0148	IDP T1 FAST ROIs V cerebellum crus II	Volume of grey matter in Vermis Crus II Cerebellum
0149	IDP T1 FAST ROIs R cerebellum crus II	Volume of grey matter in Right Crus II Cerebellum
0150	IDP T1 FAST ROIs L cerebellum VIIb	Volume of grey matter in Left VIIb Cerebellum
0151	IDP T1 FAST ROIs V cerebellum VIIb	Volume of grey matter in Vermis VIIb Cerebellum
0152	IDP T1 FAST ROIs R cerebellum VIIb	Volume of grey matter in Right VIIb Cerebellum
0153	IDP T1 FAST ROIs L cerebellum VIIIa	Volume of grey matter in Left VIIIa Cerebellum
0154	IDP T1 FAST ROIs V cerebellum VIIIa	Volume of grey matter in Vermis VIIIa Cerebellum
0155	IDP T1 FAST ROIs R cerebellum VIIIa	Volume of grey matter in Right VIIIa Cerebellum
0156	IDP T1 FAST ROIs L cerebellum VIIIb	Volume of grey matter in Left VIIIb Cerebellum
0157	IDP T1 FAST ROIs V cerebellum VIIIb	Volume of grey matter in Vermis VIIIb Cerebellum
0158	IDP T1 FAST ROIs R cerebellum VIIIb	Volume of grey matter in Right VIIIb Cerebellum
0159	IDP T1 FAST ROIs L cerebellum IX	Volume of grey matter in Left IX Cerebellum
0160	IDP T1 FAST ROIs V cerebellum IX	Volume of grey matter in Vermis IX Cerebellum
0161	IDP T1 FAST ROIs R cerebellum IX	Volume of grey matter in Right IX Cerebellum
0162	IDP T1 FAST ROIs L cerebellum X	Volume of grey matter in Left X Cerebellum
0163	IDP T1 FAST ROIs V cerebellum X	Volume of grey matter in Vermis X Cerebellum
0164	IDP T1 FAST ROIs R cerebellum X	Volume of grey matter in Right X Cerebellum
0165	aseg global volume BrainSeg	Volume of BrainSeg in the whole brain generated by subcortical volumetric segmentation (aseg)
0166	aseg global volume BrainSegNotVent	Volume of BrainSegNotVent in the whole brain generated by subcortical volumetric segmentation (aseg)
0167	aseg global volume BrainSegNotVentSurf	Volume of BrainSegNotVentSurf in the whole brain generated by subcortical volumetric segmentation (aseg)
0168	aseg global volume SubCortGray	Volume of SubCortGray in the whole brain generated by subcortical volumetric segmentation (aseg)
0169	aseg global volume TotalGray	Volume of TotalGray in the whole brain generated by subcortical volumetric segmentation (aseg)

IDP number	IDP short name	IDP description
0170	aseg global volume SupraTentorial	Volume of SupraTentorial in the whole brain generated by subcortical volumetric segmentation (aseg)
0171	aseg global volume SupraTentorialNotVent	Volume of SupraTentorialNotVent in the whole brain generated by subcortical volumetric segmentation (aseg)
0172	aseg global volume EstimatedTotalIntraCranial	Volume of EstimatedTotalIntraCranial in the whole brain generated by subcortical volumetric segmentation (aseg)
0173	aseg global volume VentricleChoroid	Volume of VentricleChoroid in the whole brain generated by subcortical volumetric segmentation (aseg)
0174	aseg global volume 3rd-Ventricle	Volume of 3rd-Ventricle in the whole brain generated by subcortical volumetric segmentation (aseg)
0175	aseg global volume 4th-Ventricle	Volume of 4th-Ventricle in the whole brain generated by subcortical volumetric segmentation (aseg)
0176	aseg global volume 5th-Ventricle	Volume of 5th-Ventricle in the whole brain generated by subcortical volumetric segmentation (aseg)
0177	aseg global volume Brain-Stem	Volume of Brain-Stem in the whole brain generated by subcortical volumetric segmentation (aseg)
0178	aseg global volume CSF	Volume of CSF in the whole brain generated by subcortical volumetric segmentation (aseg)
0179	aseg global volume WM-hypointensities	Volume of WM-hypointensities in the whole brain generated by subcortical volumetric segmentation (aseg)
0180	aseg global volume non-WM-hypointensities	Volume of non-WM-hypointensities in the whole brain generated by subcortical volumetric segmentation (aseg)
0181	aseg global volume Optic-Chiasm	Volume of Optic-Chiasm in the whole brain generated by subcortical volumetric segmentation (aseg)
0182	aseg global volume CC-Posterior	Volume of CC-Posterior in the whole brain generated by subcortical volumetric segmentation (aseg)
0183	aseg global volume CC-Mid-Posterior	Volume of CC-Mid-Posterior in the whole brain generated by subcortical volumetric segmentation (aseg)
0184	aseg global volume CC-Central	Volume of CC-Central in the whole brain generated by subcortical volumetric segmentation (aseg)
0185	aseg global volume CC-Mid-Anterior	Volume of CC-Mid-Anterior in the whole brain generated by subcortical volumetric segmentation (aseg)
0186	aseg global volume CC-Anterior	Volume of CC-Anterior in the whole brain generated by subcortical volumetric segmentation (aseg)
0187	aseg global volume-ratio BrainSegVol-to-eTIV	Volume-ratio of BrainSegVol-to-eTIV in the whole brain generated by subcortical volumetric segmentation (aseg)
0188	aseg global volume-ratio MaskVol-to-eTIV	Volume-ratio of MaskVol-to-eTIV in the whole brain generated by subcortical volumetric segmentation (aseg)
0189	aseg lh volume Cortex	Volume of Cortex in the left hemisphere generated by subcortical volumetric segmentation (aseg)
0190	aseg lh volume CerebralWhiteMatter	Volume of CerebralWhiteMatter in the left hemisphere generated by subcortical volumetric segmentation (aseg)
0191	aseg lh volume Lateral-Ventricle	Volume of Lateral-Ventricle in the left hemisphere generated by subcortical volumetric segmentation (aseg)
0192	aseg lh volume Inf-Lat-Vent	Volume of Inf-Lat-Vent in the left hemisphere generated by subcortical volumetric segmentation (aseg)
0193	aseg lh volume Cerebellum-White-Matter	Volume of Cerebellum-White-Matter in the left hemisphere generated by subcortical volumetric segmentation (aseg)
0194	aseg lh volume Cerebellum-Cortex	Volume of Cerebellum-Cortex in the left hemisphere generated by subcortical volumetric segmentation (aseg)
0195	aseg lh volume Thalamus-Proper	Volume of Thalamus-Proper in the left hemisphere generated by subcortical volumetric segmentation (aseg)
0196	aseg lh volume Caudate	Volume of Caudate in the left hemisphere generated by subcortical volumetric segmentation (aseg)
0197	aseg lh volume Putamen	Volume of Putamen in the left hemisphere generated by subcortical volumetric segmentation (aseg)
0198	aseg lh volume Pallidum	Volume of Pallidum in the left hemisphere generated by subcortical volumetric segmentation (aseg)
0199	aseg lh volume Hippocampus	Volume of Hippocampus in the left hemisphere generated by subcortical volumetric segmentation (aseg)
0200	aseg lh volume Amygdala	Volume of Amygdala in the left hemisphere generated by subcortical volumetric segmentation (aseg)
0201	aseg lh volume Accumbens-area	Volume of Accumbens-area in the left hemisphere generated by subcortical volumetric segmentation (aseg)
0202	aseg lh volume VentralDC	Volume of VentralDC in the left hemisphere generated by subcortical volumetric segmentation (aseg)
0203	aseg lh volume vessel	Volume of vessel in the left hemisphere generated by subcortical volumetric segmentation (aseg)
0204	aseg lh volume choroid-plexus	Volume of choroid-plexus in the left hemisphere generated by subcortical volumetric segmentation (aseg)
0205	aseg lh number HolesBeforeFixing	Number of HolesBeforeFixing in the left hemisphere generated by subcortical volumetric segmentation (aseg)
0206	aseg rh volume Cortex	Volume of Cortex in the right hemisphere generated by subcortical volumetric segmentation (aseg)
0207	aseg rh volume CerebralWhiteMatter	Volume of CerebralWhiteMatter in the right hemisphere generated by subcortical volumetric segmentation (aseg)
0208	aseg rh volume Lateral-Ventricle	Volume of Lateral-Ventricle in the right hemisphere generated by subcortical volumetric segmentation (aseg)
0209	aseg rh volume Inf-Lat-Vent	Volume of Inf-Lat-Vent in the right hemisphere generated by subcortical volumetric segmentation (aseg)
0210	aseg rh volume Cerebellum-White-Matter	Volume of Cerebellum-White-Matter in the right hemisphere generated by subcortical volumetric segmentation (aseg)
0211	aseg rh volume Cerebellum-Cortex	Volume of Cerebellum-Cortex in the right hemisphere generated by subcortical volumetric segmentation (aseg)
0212	aseg rh volume Thalamus-Proper	Volume of Thalamus-Proper in the right hemisphere generated by subcortical volumetric segmentation (aseg)
0213	aseg rh volume Caudate	Volume of Caudate in the right hemisphere generated by subcortical volumetric segmentation (aseg)
0214	aseg rh volume Putamen	Volume of Putamen in the right hemisphere generated by subcortical volumetric segmentation (aseg)
0215	aseg rh volume Pallidum	Volume of Pallidum in the right hemisphere generated by subcortical volumetric segmentation (aseg)
0216	aseg rh volume Hippocampus	Volume of Hippocampus in the right hemisphere generated by subcortical volumetric segmentation (aseg)
0217	aseg rh volume Amygdala	Volume of Amygdala in the right hemisphere generated by subcortical volumetric segmentation (aseg)
0218	aseg rh volume Accumbens-area	Volume of Accumbens-area in the right hemisphere generated by subcortical volumetric segmentation (aseg)
0219	aseg rh volume VentralDC	Volume of VentralDC in the right hemisphere generated by subcortical volumetric segmentation (aseg)
0220	aseg rh volume vessel	Volume of vessel in the right hemisphere generated by subcortical volumetric segmentation (aseg)
0221	aseg rh volume choroid-plexus	Volume of choroid-plexus in the right hemisphere generated by subcortical volumetric segmentation (aseg)
0222	aseg rh number HolesBeforeFixing	Number of HolesBeforeFixing in the right hemisphere generated by subcortical volumetric segmentation (aseg)
0223	AmygNuclei lh volume Lateral-nucleus	Volume of Lateral-nucleus in the left hemisphere generated by subcortical volumetric sub-segmentation of the Amygdal
0224	AmygNuclei lh volume Basal-nucleus	Volume of Basal-nucleus in the left hemisphere generated by subcortical volumetric sub-segmentation of the Amygdala
0225	AmygNuclei lh volume Accessory-Basal-nucleus	Volume of Accessory-Basal-nucleus in the left hemisphere generated by subcortical volumetric sub-segmentation of the
0226	AmygNuclei lh volume Anterior-amygdaloid-area-AAA	Volume of Anterior-amygdaloid-area-AAA in the left hemisphere generated by subcortical volumetric sub-segmentation

IDP number	IDP short name	IDP description
0227	AmygNuclei lh volume Central-nucleus	Volume of Central-nucleus in the left hemisphere generated by subcortical volumetric sub-segmentation of the Amygdal
0228	AmygNuclei lh volume Medial-nucleus	Volume of Medial-nucleus in the left hemisphere generated by subcortical volumetric sub-segmentation of the Amygdala
0229	AmygNuclei lh volume Cortical-nucleus	Volume of Cortical-nucleus in the left hemisphere generated by subcortical volumetric sub-segmentation of the Amygda
0230	AmygNuclei lh volume Corticoamygdaloid-transitio	Volume of Corticoamygdaloid-transitio in the left hemisphere generated by subcortical volumetric sub-segmentation of t
0231	AmygNuclei lh volume Paralaminar-nucleus	Volume of Paralaminar-nucleus in the left hemisphere generated by subcortical volumetric sub-segmentation of the Am
0232	AmygNuclei lh volume Whole-amygdala	Volume of Whole-amygdala in the left hemisphere generated by subcortical volumetric sub-segmentation of the Amygda
0233	AmygNuclei rh volume Lateral-nucleus	Volume of Lateral-nucleus in the right hemisphere generated by subcortical volumetric sub-segmentation of the Amygda
0234	AmygNuclei rh volume Basal-nucleus	Volume of Basal-nucleus in the right hemisphere generated by subcortical volumetric sub-segmentation of the Amygdal
0235	AmygNuclei rh volume Accessory-Basal-nucleus	Volume of Accessory-Basal-nucleus in the right hemisphere generated by subcortical volumetric sub-segmentation of th
0236	AmygNuclei rh volume Anterior-amygdaloid-area-AAA	Volume of Anterior-amygdaloid-area-AAA in the right hemisphere generated by subcortical volumetric sub-segmentatio
0237	AmygNuclei rh volume Central-nucleus	Volume of Central-nucleus in the right hemisphere generated by subcortical volumetric sub-segmentation of the Amygd
0238	AmygNuclei rh volume Medial-nucleus	Volume of Medial-nucleus in the right hemisphere generated by subcortical volumetric sub-segmentation of the Amygda
0239	AmygNuclei rh volume Cortical-nucleus	Volume of Cortical-nucleus in the right hemisphere generated by subcortical volumetric sub-segmentation of the Amygd
0240	AmygNuclei rh volume Corticoamygdaloid-transitio	Volume of Corticoamygdaloid-transitio in the right hemisphere generated by subcortical volumetric sub-segmentation of
0241	AmygNuclei rh volume Paralaminar-nucleus	Volume of Paralaminar-nucleus in the right hemisphere generated by subcortical volumetric sub-segmentation of the Ar
0242	AmygNuclei rh volume Whole-amygdala	Volume of Whole-amygdala in the right hemisphere generated by subcortical volumetric sub-segmentation of the Amyg
0243	HippSubfield lh volume Hippocampal-tail	Volume of Hippocampal-tail in the left hemisphere generated by subcortical volumetric sub-segmentation of the Hippoca
0244	HippSubfield lh volume subiculum-body	Volume of subiculum-body in the left hemisphere generated by subcortical volumetric sub-segmentation of the Hippocal
0245	HippSubfield lh volume CA1-body	Volume of CA1-body in the left hemisphere generated by subcortical volumetric sub-segmentation of the Hippocampal S
0246	HippSubfield lh volume subiculum-head	Volume of subiculum-head in the left hemisphere generated by subcortical volumetric sub-segmentation of the Hippoca
0247	HippSubfield lh volume hippocampal-fissure	Volume of hippocampal-fissure in the left hemisphere generated by subcortical volumetric sub-segmentation of the Hipp
0248	HippSubfield lh volume presubiculum-head	Volume of presubiculum-head in the left hemisphere generated by subcortical volumetric sub-segmentation of the Hipp
0249	HippSubfield lh volume CA1-head	Volume of CA1-head in the left hemisphere generated by subcortical volumetric sub-segmentation of the Hippocampal S
0250	HippSubfield lh volume presubiculum-body	Volume of presubiculum-body in the left hemisphere generated by subcortical volumetric sub-segmentation of the Hipp
0251	HippSubfield lh volume parasubiculum	Volume of parasubiculum in the left hemisphere generated by subcortical volumetric sub-segmentation of the Hippocam
0252	HippSubfield lh volume molecular-layer-HP-head	Volume of molecular-layer-HP-head in the left hemisphere generated by subcortical volumetric sub-segmentation of the
0253	HippSubfield lh volume molecular-layer-HP-body	Volume of molecular-layer-HP-body in the left hemisphere generated by subcortical volumetric sub-segmentation of the
0254	HippSubfield lh volume GC-ML-DG-head	Volume of GC-ML-DG-head in the left hemisphere generated by subcortical volumetric sub-segmentation of the Hippoc
0255	HippSubfield lh volume CA3-body	Volume of CA3-body in the left hemisphere generated by subcortical volumetric sub-segmentation of the Hippocampal S
0256	HippSubfield lh volume GC-ML-DG-body	Volume of GC-ML-DG-body in the left hemisphere generated by subcortical volumetric sub-segmentation of the Hippoc
0257	HippSubfield lh volume CA4-head	Volume of CA4-head in the left hemisphere generated by subcortical volumetric sub-segmentation of the Hippocampal S
0258	HippSubfield lh volume CA4-body	Volume of CA4-body in the left hemisphere generated by subcortical volumetric sub-segmentation of the Hippocampal S
0259	HippSubfield lh volume fimbria	Volume of fimbria in the left hemisphere generated by subcortical volumetric sub-segmentation of the Hippocampal Sub
0260	HippSubfield lh volume CA3-head	Volume of CA3-head in the left hemisphere generated by subcortical volumetric sub-segmentation of the Hippocampal S
0261	HippSubfield lh volume HATA	Volume of HATA in the left hemisphere generated by subcortical volumetric sub-segmentation of the Hippocampal Subf
0262	HippSubfield lh volume Whole-hippocampal-body	Volume of Whole-hippocampal-body in the left hemisphere generated by subcortical volumetric sub-segmentation of the
0263	HippSubfield lh volume Whole-hippocampal-head	Volume of Whole-hippocampal-head in the left hemisphere generated by subcortical volumetric sub-segmentation of the
0264	HippSubfield lh volume Whole-hippocampus	Volume of Whole-hippocampus in the left hemisphere generated by subcortical volumetric sub-segmentation of the Hipp
0265	HippSubfield rh volume Hippocampal-tail	Volume of Hippocampal-tail in the right hemisphere generated by subcortical volumetric sub-segmentation of the Hippo
0266	HippSubfield rh volume subiculum-body	Volume of subiculum-body in the right hemisphere generated by subcortical volumetric sub-segmentation of the Hippoc
0267	HippSubfield rh volume CA1-body	Volume of CA1-body in the right hemisphere generated by subcortical volumetric sub-segmentation of the Hippocampal
0268	HippSubfield rh volume subiculum-head	Volume of subiculum-head in the right hemisphere generated by subcortical volumetric sub-segmentation of the Hippoc
0269	HippSubfield rh volume hippocampal-fissure	Volume of hippocampal-fissure in the right hemisphere generated by subcortical volumetric sub-segmentation of the Hip
0270	HippSubfield rh volume presubiculum-head	Volume of presubiculum-head in the right hemisphere generated by subcortical volumetric sub-segmentation of the Hip
0271	HippSubfield rh volume CA1-head	Volume of CA1-head in the right hemisphere generated by subcortical volumetric sub-segmentation of the Hippocampa
0272	HippSubfield rh volume presubiculum-body	Volume of presubiculum-body in the right hemisphere generated by subcortical volumetric sub-segmentation of the Hip
0273	HippSubfield rh volume parasubiculum	Volume of parasubiculum in the right hemisphere generated by subcortical volumetric sub-segmentation of the Hippoca
0274	HippSubfield rh volume molecular-layer-HP-head	Volume of molecular-layer-HP-head in the right hemisphere generated by subcortical volumetric sub-segmentation of th
0275	HippSubfield rh volume molecular-layer-HP-body	Volume of molecular-layer-HP-body in the right hemisphere generated by subcortical volumetric sub-segmentation of th
0276	HippSubfield rh volume GC-ML-DG-head	Volume of GC-ML-DG-head in the right hemisphere generated by subcortical volumetric sub-segmentation of the Hippo
0277	HippSubfield rh volume CA3-body	Volume of CA3-body in the right hemisphere generated by subcortical volumetric sub-segmentation of the Hippocampal
0278	HippSubfield rh volume GC-ML-DG-body	Volume of GC-ML-DG-body in the right hemisphere generated by subcortical volumetric sub-segmentation of the Hippo
0279	HippSubfield rh volume CA4-head	Volume of CA4-head in the right hemisphere generated by subcortical volumetric sub-segmentation of the Hippocampa
0280	HippSubfield rh volume CA4-body	Volume of CA4-body in the right hemisphere generated by subcortical volumetric sub-segmentation of the Hippocampal
0281	HippSubfield rh volume fimbria	Volume of fimbria in the right hemisphere generated by subcortical volumetric sub-segmentation of the Hippocampal Su
0282	HippSubfield rh volume CA3-head	Volume of CA3-head in the right hemisphere generated by subcortical volumetric sub-segmentation of the Hippocampa
0283	HippSubfield rh volume HATA	Volume of HATA in the right hemisphere generated by subcortical volumetric sub-segmentation of the Hippocampal Sul

IDP number	IDP short name	IDP description
0284	HippSubfield rh volume Whole-hippocampal-body	Volume of Whole-hippocampal-body in the right hemisphere generated by subcortical volumetric sub-segmentation of th
0285	HippSubfield rh volume Whole-hippocampal-head	Volume of Whole-hippocampal-head in the right hemisphere generated by subcortical volumetric sub-segmentation of tl
0286	HippSubfield rh volume Whole-hippocampus	Volume of Whole-hippocampus in the right hemisphere generated by subcortical volumetric sub-segmentation of the Hi
0287	ThalamNuclei lh volume MGN	Volume of MGN in the left hemisphere generated by subcortical volumetric sub-segmentation of the Thalamic Nuclei
0288	ThalamNuclei lh volume LGN	Volume of LGN in the left hemisphere generated by subcortical volumetric sub-segmentation of the Thalamic Nuclei
0289	ThalamNuclei lh volume PuI	Volume of PuI in the left hemisphere generated by subcortical volumetric sub-segmentation of the Thalamic Nuclei
0290	ThalamNuclei lh volume PuM	Volume of PuM in the left hemisphere generated by subcortical volumetric sub-segmentation of the Thalamic Nuclei
0291	ThalamNuclei lh volume L-Sg	Volume of L-Sg in the left hemisphere generated by subcortical volumetric sub-segmentation of the Thalamic Nuclei
0292	ThalamNuclei lh volume VPL	Volume of VPL in the left hemisphere generated by subcortical volumetric sub-segmentation of the Thalamic Nuclei
0293	ThalamNuclei lh volume CM	Volume of CM in the left hemisphere generated by subcortical volumetric sub-segmentation of the Thalamic Nuclei
0294	ThalamNuclei lh volume VLa	Volume of VLa in the left hemisphere generated by subcortical volumetric sub-segmentation of the Thalamic Nuclei
0295	ThalamNuclei lh volume PuA	Volume of PuA in the left hemisphere generated by subcortical volumetric sub-segmentation of the Thalamic Nuclei
0296	ThalamNuclei lh volume MDm	Volume of MDm in the left hemisphere generated by subcortical volumetric sub-segmentation of the Thalamic Nuclei
0297	ThalamNuclei lh volume Pf	Volume of Pf in the left hemisphere generated by subcortical volumetric sub-segmentation of the Thalamic Nuclei
0298	ThalamNuclei lh volume VAmc	Volume of VAmc in the left hemisphere generated by subcortical volumetric sub-segmentation of the Thalamic Nuclei
0299	ThalamNuclei lh volume MDl	Volume of MDl in the left hemisphere generated by subcortical volumetric sub-segmentation of the Thalamic Nuclei
0300	ThalamNuclei lh volume CeM	Volume of CeM in the left hemisphere generated by subcortical volumetric sub-segmentation of the Thalamic Nuclei
0301	ThalamNuclei lh volume VA	Volume of VA in the left hemisphere generated by subcortical volumetric sub-segmentation of the Thalamic Nuclei
0302	ThalamNuclei lh volume MV(Re)	Volume of MV(Re) in the left hemisphere generated by subcortical volumetric sub-segmentation of the Thalamic Nuclei
0303	ThalamNuclei lh volume VM	Volume of VM in the left hemisphere generated by subcortical volumetric sub-segmentation of the Thalamic Nuclei
0304	ThalamNuclei lh volume CL	Volume of CL in the left hemisphere generated by subcortical volumetric sub-segmentation of the Thalamic Nuclei
0305	ThalamNuclei lh volume PuL	Volume of PuL in the left hemisphere generated by subcortical volumetric sub-segmentation of the Thalamic Nuclei
0306	ThalamNuclei lh volume Pt	Volume of Pt in the left hemisphere generated by subcortical volumetric sub-segmentation of the Thalamic Nuclei
0307	ThalamNuclei lh volume AV	Volume of AV in the left hemisphere generated by subcortical volumetric sub-segmentation of the Thalamic Nuclei
0308	ThalamNuclei lh volume Pc	Volume of Pc in the left hemisphere generated by subcortical volumetric sub-segmentation of the Thalamic Nuclei
0309	ThalamNuclei lh volume VLp	Volume of VLp in the left hemisphere generated by subcortical volumetric sub-segmentation of the Thalamic Nuclei
0310	ThalamNuclei lh volume LP	Volume of LP in the left hemisphere generated by subcortical volumetric sub-segmentation of the Thalamic Nuclei
0311	ThalamNuclei rh volume LGN	Volume of LGN in the right hemisphere generated by subcortical volumetric sub-segmentation of the Thalamic Nuclei
0312	ThalamNuclei rh volume MGN	Volume of MGN in the right hemisphere generated by subcortical volumetric sub-segmentation of the Thalamic Nuclei
0313	ThalamNuclei rh volume PuI	Volume of PuI in the right hemisphere generated by subcortical volumetric sub-segmentation of the Thalamic Nuclei
0314	ThalamNuclei rh volume PuM	Volume of PuM in the right hemisphere generated by subcortical volumetric sub-segmentation of the Thalamic Nuclei
0315	ThalamNuclei rh volume L-Sg	Volume of L-Sg in the right hemisphere generated by subcortical volumetric sub-segmentation of the Thalamic Nuclei
0316	ThalamNuclei rh volume VPL	Volume of VPL in the right hemisphere generated by subcortical volumetric sub-segmentation of the Thalamic Nuclei
0317	ThalamNuclei rh volume CM	Volume of CM in the right hemisphere generated by subcortical volumetric sub-segmentation of the Thalamic Nuclei
0318	ThalamNuclei rh volume VLa	Volume of VLa in the right hemisphere generated by subcortical volumetric sub-segmentation of the Thalamic Nuclei
0319	ThalamNuclei rh volume PuA	Volume of PuA in the right hemisphere generated by subcortical volumetric sub-segmentation of the Thalamic Nuclei
0320	ThalamNuclei rh volume MDm	Volume of MDm in the right hemisphere generated by subcortical volumetric sub-segmentation of the Thalamic Nuclei
0321	ThalamNuclei rh volume Pf	Volume of Pf in the right hemisphere generated by subcortical volumetric sub-segmentation of the Thalamic Nuclei
0322	ThalamNuclei rh volume VAmc	Volume of VAmc in the right hemisphere generated by subcortical volumetric sub-segmentation of the Thalamic Nuclei
0323	ThalamNuclei rh volume MDl	Volume of MDl in the right hemisphere generated by subcortical volumetric sub-segmentation of the Thalamic Nuclei
0324	ThalamNuclei rh volume VA	Volume of VA in the right hemisphere generated by subcortical volumetric sub-segmentation of the Thalamic Nuclei
0325	ThalamNuclei rh volume MV(Re)	Volume of MV(Re) in the right hemisphere generated by subcortical volumetric sub-segmentation of the Thalamic Nucle
0326	ThalamNuclei rh volume CeM	Volume of CeM in the right hemisphere generated by subcortical volumetric sub-segmentation of the Thalamic Nuclei
0327	ThalamNuclei rh volume VM	Volume of VM in the right hemisphere generated by subcortical volumetric sub-segmentation of the Thalamic Nuclei
0328	ThalamNuclei rh volume PuL	Volume of PuL in the right hemisphere generated by subcortical volumetric sub-segmentation of the Thalamic Nuclei
0329	ThalamNuclei rh volume CL	Volume of CL in the right hemisphere generated by subcortical volumetric sub-segmentation of the Thalamic Nuclei
0330	ThalamNuclei rh volume VLp	Volume of VLp in the right hemisphere generated by subcortical volumetric sub-segmentation of the Thalamic Nuclei
0331	ThalamNuclei rh volume Pc	Volume of Pc in the right hemisphere generated by subcortical volumetric sub-segmentation of the Thalamic Nuclei
0332	ThalamNuclei rh volume Pt	Volume of Pt in the right hemisphere generated by subcortical volumetric sub-segmentation of the Thalamic Nuclei
0333	ThalamNuclei rh volume AV	Volume of AV in the right hemisphere generated by subcortical volumetric sub-segmentation of the Thalamic Nuclei
0334	ThalamNuclei rh volume LP	Volume of LP in the right hemisphere generated by subcortical volumetric sub-segmentation of the Thalamic Nuclei
0335	ThalamNuclei lh volume LD	Volume of LD in the left hemisphere generated by subcortical volumetric sub-segmentation of the Thalamic Nuclei
0336	ThalamNuclei rh volume LD	Volume of LD in the right hemisphere generated by subcortical volumetric sub-segmentation of the Thalamic Nuclei
0337	ThalamNuclei lh volume Whole-thalamus	Volume of Whole-thalamus in the left hemisphere generated by subcortical volumetric sub-segmentation of the Thalami
0338	ThalamNuclei rh volume Whole-thalamus	Volume of Whole-thalamus in the right hemisphere generated by subcortical volumetric sub-segmentation of the Thalan
0339	Brainstem global volume Medulla	Volume of Medulla in the whole brain generated by subcortical volumetric sub-segmentation of the Brainstem
0340	Brainstem global volume Pons	Volume of Pons in the whole brain generated by subcortical volumetric sub-segmentation of the Brainstem

IDP number	IDP short name	IDP description
0341	Brainstem global volume SCP	Volume of SCP in the whole brain generated by subcortical volumetric sub-segmentation of the Brainstem
0342	Brainstem global volume Midbrain	Volume of Midbrain in the whole brain generated by subcortical volumetric sub-segmentation of the Brainstem
0343	Brainstem global volume Whole-brainstem	Volume of Whole-brainstem in the whole brain generated by subcortical volumetric sub-segmentation of the Brainstem
0344	aparc-Desikan lh volume bankssts	Volume of bankssts in the left hemisphere generated by parcellation of the white surface using Desikan-Killiany parcella
0345	aparc-Desikan lh volume caudalanteriorcingulate	Volume of caudalanteriorcingulate in the left hemisphere generated by parcellation of the white surface using Desikan-K
0346	aparc-Desikan lh volume caudalmiddlefrontal	Volume of caudalmiddlefrontal in the left hemisphere generated by parcellation of the white surface using Desikan-Killia
0347	aparc-Desikan lh volume cuneus	Volume of cuneus in the left hemisphere generated by parcellation of the white surface using Desikan-Killiany parcellati
0348	aparc-Desikan lh volume entorhinal	Volume of entorhinal in the left hemisphere generated by parcellation of the white surface using Desikan-Killiany parcell
0349	aparc-Desikan lh volume fusiform	Volume of fusiform in the left hemisphere generated by parcellation of the white surface using Desikan-Killiany parcellat
0350	aparc-Desikan lh volume inferiorparietal	Volume of inferiorparietal in the left hemisphere generated by parcellation of the white surface using Desikan-Killiany pa
0351	aparc-Desikan lh volume inferiortemporal	Volume of inferiortemporal in the left hemisphere generated by parcellation of the white surface using Desikan-Killiany p
0352	aparc-Desikan lh volume isthmuscingulate	Volume of isthmuscingulate in the left hemisphere generated by parcellation of the white surface using Desikan-Killiany
0353	aparc-Desikan lh volume lateraloccipital	Volume of lateraloccipital in the left hemisphere generated by parcellation of the white surface using Desikan-Killiany pa
0354	aparc-Desikan lh volume lateralorbitofrontal	Volume of lateralorbitofrontal in the left hemisphere generated by parcellation of the white surface using Desikan-Killian
0355	aparc-Desikan lh volume lingual	Volume of lingual in the left hemisphere generated by parcellation of the white surface using Desikan-Killiany parcellatic
0356	aparc-Desikan lh volume medialorbitofrontal	Volume of medialorbitofrontal in the left hemisphere generated by parcellation of the white surface using Desikan-Killiar
0357	aparc-Desikan lh volume middletemporal	Volume of middletemporal in the left hemisphere generated by parcellation of the white surface using Desikan-Killiany p
0358	aparc-Desikan lh volume parahippocampal	Volume of parahippocampal in the left hemisphere generated by parcellation of the white surface using Desikan-Killiany
0359	aparc-Desikan lh volume paracentral	Volume of paracentral in the left hemisphere generated by parcellation of the white surface using Desikan-Killiany parce
0360	aparc-Desikan lh volume parsopercularis	Volume of parsopercularis in the left hemisphere generated by parcellation of the white surface using Desikan-Killiany p
0361	aparc-Desikan lh volume parsorbitalis	Volume of parsorbitalis in the left hemisphere generated by parcellation of the white surface using Desikan-Killiany parc
0362	aparc-Desikan lh volume parstriangularis	Volume of parstriangularis in the left hemisphere generated by parcellation of the white surface using Desikan-Killiany p
0363	aparc-Desikan lh volume pericalcarine	Volume of pericalcarine in the left hemisphere generated by parcellation of the white surface using Desikan-Killiany parc
0364	aparc-Desikan lh volume postcentral	Volume of postcentral in the left hemisphere generated by parcellation of the white surface using Desikan-Killiany parce
0365	aparc-Desikan lh volume posteriorcingulate	Volume of posteriorcingulate in the left hemisphere generated by parcellation of the white surface using Desikan-Killian
0366	aparc-Desikan lh volume precentral	Volume of precentral in the left hemisphere generated by parcellation of the white surface using Desikan-Killiany parcell
0367	aparc-Desikan lh volume precuneus	Volume of precuneus in the left hemisphere generated by parcellation of the white surface using Desikan-Killiany parcel
0368	aparc-Desikan lh volume rostralanteriorcingulate	Volume of rostralanteriorcingulate in the left hemisphere generated by parcellation of the white surface using Desikan-K
0369	aparc-Desikan lh volume rostralmiddlefrontal	Volume of rostralmiddlefrontal in the left hemisphere generated by parcellation of the white surface using Desikan-Killiar
0370	aparc-Desikan lh volume superiorfrontal	Volume of superiorfrontal in the left hemisphere generated by parcellation of the white surface using Desikan-Killiany pa
0371	aparc-Desikan lh volume superiorparietal	Volume of superiorparietal in the left hemisphere generated by parcellation of the white surface using Desikan-Killiany p
0372	aparc-Desikan lh volume superiortemporal	Volume of superiortemporal in the left hemisphere generated by parcellation of the white surface using Desikan-Killiany
0373	aparc-Desikan lh volume supramarginal	Volume of supramarginal in the left hemisphere generated by parcellation of the white surface using Desikan-Killiany pa
0374	aparc-Desikan lh volume frontalpole	Volume of frontalpole in the left hemisphere generated by parcellation of the white surface using Desikan-Killiany parcel
0375	aparc-Desikan lh volume transversetemporal	Volume of transversetemporal in the left hemisphere generated by parcellation of the white surface using Desikan-Killia
0376	aparc-Desikan lh volume insula	Volume of insula in the left hemisphere generated by parcellation of the white surface using Desikan-Killiany parcellatior
0377	aparc-Desikan rh volume bankssts	Volume of bankssts in the right hemisphere generated by parcellation of the white surface using Desikan-Killiany parcel
0378	aparc-Desikan rh volume caudalanteriorcingulate	Volume of caudalanteriorcingulate in the right hemisphere generated by parcellation of the white surface using Desikan-
0379	aparc-Desikan rh volume caudalmiddlefrontal	Volume of caudalmiddlefrontal in the right hemisphere generated by parcellation of the white surface using Desikan-Killi
0380	aparc-Desikan rh volume cuneus	Volume of cuneus in the right hemisphere generated by parcellation of the white surface using Desikan-Killiany parcella
0381	aparc-Desikan rh volume entorhinal	Volume of entorhinal in the right hemisphere generated by parcellation of the white surface using Desikan-Killiany parce
0382	aparc-Desikan rh volume fusiform	Volume of fusiform in the right hemisphere generated by parcellation of the white surface using Desikan-Killiany parcella
0383	aparc-Desikan rh volume inferiorparietal	Volume of inferiorparietal in the right hemisphere generated by parcellation of the white surface using Desikan-Killiany p
0384	aparc-Desikan rh volume inferiortemporal	Volume of inferiortemporal in the right hemisphere generated by parcellation of the white surface using Desikan-Killiany
0385	aparc-Desikan rh volume isthmuscingulate	Volume of isthmuscingulate in the right hemisphere generated by parcellation of the white surface using Desikan-Killian
0386	aparc-Desikan rh volume lateraloccipital	Volume of lateraloccipital in the right hemisphere generated by parcellation of the white surface using Desikan-Killiany p
0387	aparc-Desikan rh volume lateralorbitofrontal	Volume of lateralorbitofrontal in the right hemisphere generated by parcellation of the white surface using Desikan-Killia
0388	aparc-Desikan rh volume lingual	Volume of lingual in the right hemisphere generated by parcellation of the white surface using Desikan-Killiany parcellat
0389	aparc-Desikan rh volume medialorbitofrontal	Volume of medialorbitofrontal in the right hemisphere generated by parcellation of the white surface using Desikan-Killia
0390	aparc-Desikan rh volume middletemporal	Volume of middletemporal in the right hemisphere generated by parcellation of the white surface using Desikan-Killiany
0391	aparc-Desikan rh volume parahippocampal	Volume of parahippocampal in the right hemisphere generated by parcellation of the white surface using Desikan-Killiar
0392	aparc-Desikan rh volume paracentral	Volume of paracentral in the right hemisphere generated by parcellation of the white surface using Desikan-Killiany parc
0393	aparc-Desikan rh volume parsopercularis	Volume of parsopercularis in the right hemisphere generated by parcellation of the white surface using Desikan-Killiany
0394	aparc-Desikan rh volume parsorbitalis	Volume of parsorbitalis in the right hemisphere generated by parcellation of the white surface using Desikan-Killiany pai
0395	aparc-Desikan rh volume parstriangularis	Volume of parstriangularis in the right hemisphere generated by parcellation of the white surface using Desikan-Killiany
0396	aparc-Desikan rh volume pericalcarine	Volume of pericalcarine in the right hemisphere generated by parcellation of the white surface using Desikan-Killiany pa
0397	aparc-Desikan rh volume postcentral	Volume of postcentral in the right hemisphere generated by parcellation of the white surface using Desikan-Killiany parc

IDP number	IDP short name	IDP description
0398	aparc-Desikan rh volume posteriorcingulate	Volume of posteriorcingulate in the right hemisphere generated by parcellation of the white surface using Desikan-Killia
0399	aparc-Desikan rh volume precentral	Volume of precentral in the right hemisphere generated by parcellation of the white surface using Desikan-Killiany parce
0400	aparc-Desikan rh volume precuneus	Volume of precuneus in the right hemisphere generated by parcellation of the white surface using Desikan-Killiany parc
0401	aparc-Desikan rh volume rostralanteriorcingulate	Volume of rostralanteriorcingulate in the right hemisphere generated by parcellation of the white surface using Desikan-
0402	aparc-Desikan rh volume rostralmiddlefrontal	Volume of rostralmiddlefrontal in the right hemisphere generated by parcellation of the white surface using Desikan-Killi
0403	aparc-Desikan rh volume superiorfrontal	Volume of superiorfrontal in the right hemisphere generated by parcellation of the white surface using Desikan-Killiany p
0404	aparc-Desikan rh volume superiorparietal	Volume of superiorparietal in the right hemisphere generated by parcellation of the white surface using Desikan-Killiany
0405	aparc-Desikan rh volume superiortemporal	Volume of superiortemporal in the right hemisphere generated by parcellation of the white surface using Desikan-Killian
0406	aparc-Desikan rh volume supramarginal	Volume of supramarginal in the right hemisphere generated by parcellation of the white surface using Desikan-Killiany p
0407	aparc-Desikan rh volume frontalpole	Volume of frontalpole in the right hemisphere generated by parcellation of the white surface using Desikan-Killiany parc
0408	aparc-Desikan rh volume transversetemporal	Volume of transversetemporal in the right hemisphere generated by parcellation of the white surface using Desikan-Killi
0409	aparc-Desikan rh volume insula	Volume of insula in the right hemisphere generated by parcellation of the white surface using Desikan-Killiany parcellati
0410	BA-exvivo lh volume BA1	Volume of BA1 in the left hemisphere generated by parcellation of the white surface using BA_exvivo parcellation
0411	BA-exvivo lh volume BA2	Volume of BA2 in the left hemisphere generated by parcellation of the white surface using BA_exvivo parcellation
0412	BA-exvivo lh volume BA3a	Volume of BA3a in the left hemisphere generated by parcellation of the white surface using BA_exvivo parcellation
0413	BA-exvivo lh volume BA3b	Volume of BA3b in the left hemisphere generated by parcellation of the white surface using BA_exvivo parcellation
0414	BA-exvivo lh volume BA4a	Volume of BA4a in the left hemisphere generated by parcellation of the white surface using BA_exvivo parcellation
0415	BA-exvivo lh volume BA4p	Volume of BA4p in the left hemisphere generated by parcellation of the white surface using BA_exvivo parcellation
0416	BA-exvivo lh volume BA6	Volume of BA6 in the left hemisphere generated by parcellation of the white surface using BA_exvivo parcellation
0417	BA-exvivo lh volume BA44	Volume of BA44 in the left hemisphere generated by parcellation of the white surface using BA_exvivo parcellation
0418	BA-exvivo lh volume BA45	Volume of BA45 in the left hemisphere generated by parcellation of the white surface using BA_exvivo parcellation
0419	BA-exvivo lh volume V1	Volume of V1 in the left hemisphere generated by parcellation of the white surface using BA_exvivo parcellation
0420	BA-exvivo lh volume V2	Volume of V2 in the left hemisphere generated by parcellation of the white surface using BA_exvivo parcellation
0421	BA-exvivo lh volume MT	Volume of MT in the left hemisphere generated by parcellation of the white surface using BA_exvivo parcellation
0422	BA-exvivo lh volume perirhinal	Volume of perirhinal in the left hemisphere generated by parcellation of the white surface using BA_exvivo parcellation
0423	BA-exvivo lh volume entorhinal	Volume of entorhinal in the left hemisphere generated by parcellation of the white surface using BA_exvivo parcellation
0424	BA-exvivo rh volume BA1	Volume of BA1 in the right hemisphere generated by parcellation of the white surface using BA_exvivo parcellation
0425	BA-exvivo rh volume BA2	Volume of BA2 in the right hemisphere generated by parcellation of the white surface using BA_exvivo parcellation
0426	BA-exvivo rh volume BA3a	Volume of BA3a in the right hemisphere generated by parcellation of the white surface using BA_exvivo parcellation
0427	BA-exvivo rh volume BA3b	Volume of BA3b in the right hemisphere generated by parcellation of the white surface using BA_exvivo parcellation
0428	BA-exvivo rh volume BA4a	Volume of BA4a in the right hemisphere generated by parcellation of the white surface using BA_exvivo parcellation
0429	BA-exvivo rh volume BA4p	Volume of BA4p in the right hemisphere generated by parcellation of the white surface using BA_exvivo parcellation
0430	BA-exvivo rh volume BA6	Volume of BA6 in the right hemisphere generated by parcellation of the white surface using BA_exvivo parcellation
0431	BA-exvivo rh volume BA44	Volume of BA44 in the right hemisphere generated by parcellation of the white surface using BA_exvivo parcellation
0432	BA-exvivo rh volume BA45	Volume of BA45 in the right hemisphere generated by parcellation of the white surface using BA_exvivo parcellation
0433	BA-exvivo rh volume V1	Volume of V1 in the right hemisphere generated by parcellation of the white surface using BA_exvivo parcellation
0434	BA-exvivo rh volume V2	Volume of V2 in the right hemisphere generated by parcellation of the white surface using BA_exvivo parcellation
0435	BA-exvivo rh volume MT	Volume of MT in the right hemisphere generated by parcellation of the white surface using BA_exvivo parcellation
0436	BA-exvivo rh volume perirhinal	Volume of perirhinal in the right hemisphere generated by parcellation of the white surface using BA_exvivo parcellatior
0437	BA-exvivo rh volume entorhinal	Volume of entorhinal in the right hemisphere generated by parcellation of the white surface using BA_exvivo parcellatior
0438	aparc-DKTatlas lh volume caudalanteriorcingulate	Volume of caudalanteriorcingulate in the left hemisphere generated by parcellation of the white surface using DKT parc
0439	aparc-DKTatlas lh volume caudalmiddlefrontal	Volume of caudalmiddlefrontal in the left hemisphere generated by parcellation of the white surface using DKT parcellat
0440	aparc-DKTatlas lh volume cuneus	Volume of cuneus in the left hemisphere generated by parcellation of the white surface using DKT parcellation
0441	aparc-DKTatlas lh volume entorhinal	Volume of entorhinal in the left hemisphere generated by parcellation of the white surface using DKT parcellation
0442	aparc-DKTatlas lh volume fusiform	Volume of fusiform in the left hemisphere generated by parcellation of the white surface using DKT parcellation
0443	aparc-DKTatlas lh volume inferiorparietal	Volume of inferiorparietal in the left hemisphere generated by parcellation of the white surface using DKT parcellation
0444	aparc-DKTatlas lh volume inferiortemporal	Volume of inferiortemporal in the left hemisphere generated by parcellation of the white surface using DKT parcellation
0445	aparc-DKTatlas lh volume isthmuscingulate	Volume of isthmuscingulate in the left hemisphere generated by parcellation of the white surface using DKT parcellatior
0446	aparc-DKTatlas lh volume lateraloccipital	Volume of lateraloccipital in the left hemisphere generated by parcellation of the white surface using DKT parcellation
0447	aparc-DKTatlas lh volume lateralorbitofrontal	Volume of lateralorbitofrontal in the left hemisphere generated by parcellation of the white surface using DKT parcellatic
0448	aparc-DKTatlas lh volume lingual	Volume of lingual in the left hemisphere generated by parcellation of the white surface using DKT parcellation
0449	aparc-DKTatlas lh volume medialorbitofrontal	Volume of medialorbitofrontal in the left hemisphere generated by parcellation of the white surface using DKT parcellatic
0450	aparc-DKTatlas lh volume middletemporal	Volume of middletemporal in the left hemisphere generated by parcellation of the white surface using DKT parcellation
0451	aparc-DKTatlas lh volume parahippocampal	Volume of parahippocampal in the left hemisphere generated by parcellation of the white surface using DKT parcellatior
0452	aparc-DKTatlas lh volume paracentral	Volume of paracentral in the left hemisphere generated by parcellation of the white surface using DKT parcellation
0453	aparc-DKTatlas lh volume parsopercularis	Volume of parsopercularis in the left hemisphere generated by parcellation of the white surface using DKT parcellation
0454	aparc-DKTatlas lh volume parsorbitalis	Volume of parsorbitalis in the left hemisphere generated by parcellation of the white surface using DKT parcellation

IDP number	IDP short name	IDP description
0455	aparc-DKTatlas lh volume parstriangularis	Volume of parstriangularis in the left hemisphere generated by parcellation of the white surface using DKT parcellation
0456	aparc-DKTatlas lh volume pericalcarine	Volume of pericalcarine in the left hemisphere generated by parcellation of the white surface using DKT parcellation
0457	aparc-DKTatlas lh volume postcentral	Volume of postcentral in the left hemisphere generated by parcellation of the white surface using DKT parcellation
0458	aparc-DKTatlas lh volume posteriorcingulate	Volume of posteriorcingulate in the left hemisphere generated by parcellation of the white surface using DKT parcellatio
0459	aparc-DKTatlas lh volume precentral	Volume of precentral in the left hemisphere generated by parcellation of the white surface using DKT parcellation
0460	aparc-DKTatlas lh volume precuneus	Volume of precuneus in the left hemisphere generated by parcellation of the white surface using DKT parcellation
0461	aparc-DKTatlas lh volume rostralanteriorcingulate	Volume of rostralanteriorcingulate in the left hemisphere generated by parcellation of the white surface using DKT parce
0462	aparc-DKTatlas lh volume rostralmiddlefrontal	Volume of rostralmiddlefrontal in the left hemisphere generated by parcellation of the white surface using DKT parcellati
0463	aparc-DKTatlas lh volume superiorfrontal	Volume of superiorfrontal in the left hemisphere generated by parcellation of the white surface using DKT parcellation
0464	aparc-DKTatlas lh volume superiorparietal	Volume of superiorparietal in the left hemisphere generated by parcellation of the white surface using DKT parcellation
0465	aparc-DKTatlas lh volume superiortemporal	Volume of superiortemporal in the left hemisphere generated by parcellation of the white surface using DKT parcellation
0466	aparc-DKTatlas lh volume supramarginal	Volume of supramarginal in the left hemisphere generated by parcellation of the white surface using DKT parcellation
0467	aparc-DKTatlas lh volume transversetemporal	Volume of transversetemporal in the left hemisphere generated by parcellation of the white surface using DKT parcellat
0468	aparc-DKTatlas lh volume insula	Volume of insula in the left hemisphere generated by parcellation of the white surface using DKT parcellation
0469	aparc-DKTatlas rh volume caudalanteriorcingulate	Volume of caudalanteriorcingulate in the right hemisphere generated by parcellation of the white surface using DKT par
0470	aparc-DKTatlas rh volume caudalmiddlefrontal	Volume of caudalmiddlefrontal in the right hemisphere generated by parcellation of the white surface using DKT parcella
0471	aparc-DKTatlas rh volume cuneus	Volume of cuneus in the right hemisphere generated by parcellation of the white surface using DKT parcellation
0472	aparc-DKTatlas rh volume entorhinal	Volume of entorhinal in the right hemisphere generated by parcellation of the white surface using DKT parcellation
0473	aparc-DKTatlas rh volume fusiform	Volume of fusiform in the right hemisphere generated by parcellation of the white surface using DKT parcellation
0474	aparc-DKTatlas rh volume inferiorparietal	Volume of inferiorparietal in the right hemisphere generated by parcellation of the white surface using DKT parcellation
0475	aparc-DKTatlas rh volume inferiortemporal	Volume of inferiortemporal in the right hemisphere generated by parcellation of the white surface using DKT parcellation
0476	aparc-DKTatlas rh volume isthmuscingulate	Volume of isthmuscingulate in the right hemisphere generated by parcellation of the white surface using DKT parcellatic
0477	aparc-DKTatlas rh volume lateraloccipital	Volume of lateraloccipital in the right hemisphere generated by parcellation of the white surface using DKT parcellation
0478	aparc-DKTatlas rh volume lateralorbitofrontal	Volume of lateralorbitofrontal in the right hemisphere generated by parcellation of the white surface using DKT parcellat
0479	aparc-DKTatlas rh volume lingual	Volume of lingual in the right hemisphere generated by parcellation of the white surface using DKT parcellation
0480	aparc-DKTatlas rh volume medialorbitofrontal	Volume of medialorbitofrontal in the right hemisphere generated by parcellation of the white surface using DKT parcella
0481	aparc-DKTatlas rh volume middletemporal	Volume of middletemporal in the right hemisphere generated by parcellation of the white surface using DKT parcellation
0482	aparc-DKTatlas rh volume parahippocampal	Volume of parahippocampal in the right hemisphere generated by parcellation of the white surface using DKT parcellati
0483	aparc-DKTatlas rh volume paracentral	Volume of paracentral in the right hemisphere generated by parcellation of the white surface using DKT parcellation
0484	aparc-DKTatlas rh volume parsopercularis	Volume of parsopercularis in the right hemisphere generated by parcellation of the white surface using DKT parcellation
0485	aparc-DKTatlas rh volume parsorbitalis	Volume of parsorbitalis in the right hemisphere generated by parcellation of the white surface using DKT parcellation
0486	aparc-DKTatlas rh volume parstriangularis	Volume of parstriangularis in the right hemisphere generated by parcellation of the white surface using DKT parcellation
0487	aparc-DKTatlas rh volume pericalcarine	Volume of pericalcarine in the right hemisphere generated by parcellation of the white surface using DKT parcellation
0488	aparc-DKTatlas rh volume postcentral	Volume of postcentral in the right hemisphere generated by parcellation of the white surface using DKT parcellation
0489	aparc-DKTatlas rh volume posteriorcingulate	Volume of posteriorcingulate in the right hemisphere generated by parcellation of the white surface using DKT parcellati
0490	aparc-DKTatlas rh volume precentral	Volume of precentral in the right hemisphere generated by parcellation of the white surface using DKT parcellation
0491	aparc-DKTatlas rh volume precuneus	Volume of precuneus in the right hemisphere generated by parcellation of the white surface using DKT parcellation
0492	aparc-DKTatlas rh volume rostralanteriorcingulate	Volume of rostralanteriorcingulate in the right hemisphere generated by parcellation of the white surface using DKT parc
0493	aparc-DKTatlas rh volume rostralmiddlefrontal	Volume of rostralmiddlefrontal in the right hemisphere generated by parcellation of the white surface using DKT parcella
0494	aparc-DKTatlas rh volume superiorfrontal	Volume of superiorfrontal in the right hemisphere generated by parcellation of the white surface using DKT parcellation
0495	aparc-DKTatlas rh volume superiorparietal	Volume of superiorparietal in the right hemisphere generated by parcellation of the white surface using DKT parcellation
0496	aparc-DKTatlas rh volume superiortemporal	Volume of superiortemporal in the right hemisphere generated by parcellation of the white surface using DKT parcellatic
0497	aparc-DKTatlas rh volume supramarginal	Volume of supramarginal in the right hemisphere generated by parcellation of the white surface using DKT parcellation
0498	aparc-DKTatlas rh volume transversetemporal	Volume of transversetemporal in the right hemisphere generated by parcellation of the white surface using DKT parcella
0499	aparc-DKTatlas rh volume insula	Volume of insula in the right hemisphere generated by parcellation of the white surface using DKT parcellation
0500	aparc-a2009s lh volume G+S-frontomargin	Volume of G+S-frontomargin in the left hemisphere generated by parcellation of the white surface using Destrieux (a20(
0501	aparc-a2009s lh volume G+S-occipital-inf	Volume of G+S-occipital-inf in the left hemisphere generated by parcellation of the white surface using Destrieux (a2009
0502	aparc-a2009s lh volume G+S-paracentral	Volume of G+S-paracentral in the left hemisphere generated by parcellation of the white surface using Destrieux (a2009
0503	aparc-a2009s lh volume G+S-subcentral	Volume of G+S-subcentral in the left hemisphere generated by parcellation of the white surface using Destrieux (a2009
0504	aparc-a2009s lh volume G+S-transv-frontopol	Volume of G+S-transv-frontopol in the left hemisphere generated by parcellation of the white surface using Destrieux (a
0505	aparc-a2009s lh volume G+S-cingul-Ant	Volume of G+S-cingul-Ant in the left hemisphere generated by parcellation of the white surface using Destrieux (a2009s
0506	aparc-a2009s lh volume G+S-cingul-Mid-Ant	Volume of G+S-cingul-Mid-Ant in the left hemisphere generated by parcellation of the white surface using Destrieux (a2
0507	aparc-a2009s lh volume G+S-cingul-Mid-Post	Volume of G+S-cingul-Mid-Post in the left hemisphere generated by parcellation of the white surface using Destrieux (a
0508	aparc-a2009s lh volume G-cingul-Post-dorsal	Volume of G-cingul-Post-dorsal in the left hemisphere generated by parcellation of the white surface using Destrieux (a
0509	aparc-a2009s lh volume G-cingul-Post-ventral	Volume of G-cingul-Post-ventral in the left hemisphere generated by parcellation of the white surface using Destrieux (a
0510	aparc-a2009s lh volume G-cuneus	Volume of G-cuneus in the left hemisphere generated by parcellation of the white surface using Destrieux (a2009s) parc
0511	aparc-a2009s lh volume G-front-inf-Opercular	Volume of G-front-inf-Opercular in the left hemisphere generated by parcellation of the white surface using Destrieux (a

IDP number	IDP short name	IDP description
0512	aparc-a2009s lh volume G-front-inf-Orbital	Volume of G-front-inf-Orbital in the left hemisphere generated by parcellation of the white surface using Destrieux (a200
0513	aparc-a2009s lh volume G-front-inf-Triangul	Volume of G-front-inf-Triangul in the left hemisphere generated by parcellation of the white surface using Destrieux (a20
0514	aparc-a2009s lh volume G-front-middle	Volume of G-front-middle in the left hemisphere generated by parcellation of the white surface using Destrieux (a2009s)
0515	aparc-a2009s lh volume G-front-sup	Volume of G-front-sup in the left hemisphere generated by parcellation of the white surface using Destrieux (a2009s) pa
0516	aparc-a2009s lh volume G-Ins-lg+S-cent-ins	Volume of G-Ins-lg+S-cent-ins in the left hemisphere generated by parcellation of the white surface using Destrieux (a2
0517	aparc-a2009s lh volume G-insular-short	Volume of G-insular-short in the left hemisphere generated by parcellation of the white surface using Destrieux (a2009s
0518	aparc-a2009s lh volume G-occipital-middle	Volume of G-occipital-middle in the left hemisphere generated by parcellation of the white surface using Destrieux (a20
0519	aparc-a2009s lh volume G-occipital-sup	Volume of G-occipital-sup in the left hemisphere generated by parcellation of the white surface using Destrieux (a2009s
0520	aparc-a2009s lh volume G-oc-temp-lat-fusifor	Volume of G-oc-temp-lat-fusifor in the left hemisphere generated by parcellation of the white surface using Destrieux (a
0521	aparc-a2009s lh volume G-oc-temp-med-Lingual	Volume of G-oc-temp-med-Lingual in the left hemisphere generated by parcellation of the white surface using Destrieux
0522	aparc-a2009s lh volume G-oc-temp-med-Parahip	Volume of G-oc-temp-med-Parahip in the left hemisphere generated by parcellation of the white surface using Destrieux
0523	aparc-a2009s lh volume G-orbital	Volume of G-orbital in the left hemisphere generated by parcellation of the white surface using Destrieux (a2009s) parc
0524	aparc-a2009s lh volume G-pariet-inf-Angular	Volume of G-pariet-inf-Angular in the left hemisphere generated by parcellation of the white surface using Destrieux (a2
0525	aparc-a2009s lh volume G-pariet-inf-Supramar	Volume of G-pariet-inf-Supramar in the left hemisphere generated by parcellation of the white surface using Destrieux (
0526	aparc-a2009s lh volume G-parietal-sup	Volume of G-parietal-sup in the left hemisphere generated by parcellation of the white surface using Destrieux (a2009s)
0527	aparc-a2009s lh volume G-postcentral	Volume of G-postcentral in the left hemisphere generated by parcellation of the white surface using Destrieux (a2009s)
0528	aparc-a2009s lh volume G-precentral	Volume of G-precentral in the left hemisphere generated by parcellation of the white surface using Destrieux (a2009s) p
0529	aparc-a2009s lh volume G-precuneus	Volume of G-precuneus in the left hemisphere generated by parcellation of the white surface using Destrieux (a2009s)
0530	aparc-a2009s lh volume G-rectus	Volume of G-rectus in the left hemisphere generated by parcellation of the white surface using Destrieux (a2009s) parc
0531	aparc-a2009s lh volume G-subcallosal	Volume of G-subcallosal in the left hemisphere generated by parcellation of the white surface using Destrieux (a2009s)
0532	aparc-a2009s lh volume G-temp-sup-G-T-transv	Volume of G-temp-sup-G-T-transv in the left hemisphere generated by parcellation of the white surface using Destrieux
0533	aparc-a2009s lh volume G-temp-sup-Lateral	Volume of G-temp-sup-Lateral in the left hemisphere generated by parcellation of the white surface using Destrieux (a2
0534	aparc-a2009s lh volume G-temp-sup-Plan-polar	Volume of G-temp-sup-Plan-polar in the left hemisphere generated by parcellation of the white surface using Destrieux
0535	aparc-a2009s lh volume G-temp-sup-Plan-tempo	Volume of G-temp-sup-Plan-tempo in the left hemisphere generated by parcellation of the white surface using Destrieux
0536	aparc-a2009s lh volume G-temporal-inf	Volume of G-temporal-inf in the left hemisphere generated by parcellation of the white surface using Destrieux (a2009s)
0537	aparc-a2009s lh volume G-temporal-middle	Volume of G-temporal-middle in the left hemisphere generated by parcellation of the white surface using Destrieux (a20
0538	aparc-a2009s lh volume Lat-Fis-ant-Horizont	Volume of Lat-Fis-ant-Horizont in the left hemisphere generated by parcellation of the white surface using Destrieux (a2
0539	aparc-a2009s lh volume Lat-Fis-ant-Vertical	Volume of Lat-Fis-ant-Vertical in the left hemisphere generated by parcellation of the white surface using Destrieux (a20
0540	aparc-a2009s lh volume Lat-Fis-post	Volume of Lat-Fis-post in the left hemisphere generated by parcellation of the white surface using Destrieux (a2009s) pa
0541	aparc-a2009s lh volume Pole-occipital	Volume of Pole-occipital in the left hemisphere generated by parcellation of the white surface using Destrieux (a2009s)
0542	aparc-a2009s lh volume Pole-temporal	Volume of Pole-temporal in the left hemisphere generated by parcellation of the white surface using Destrieux (a2009s)
0543	aparc-a2009s lh volume S-calcarine	Volume of S-calcarine in the left hemisphere generated by parcellation of the white surface using Destrieux (a2009s) pa
0544	aparc-a2009s lh volume S-central	Volume of S-central in the left hemisphere generated by parcellation of the white surface using Destrieux (a2009s) parc
0545	aparc-a2009s lh volume S-cingul-Marginalis	Volume of S-cingul-Marginalis in the left hemisphere generated by parcellation of the white surface using Destrieux (a20
0546	aparc-a2009s lh volume S-circular-insula-ant	Volume of S-circular-insula-ant in the left hemisphere generated by parcellation of the white surface using Destrieux (a2
0547	aparc-a2009s lh volume S-circular-insula-inf	Volume of S-circular-insula-inf in the left hemisphere generated by parcellation of the white surface using Destrieux (a20
0548	aparc-a2009s lh volume S-circular-insula-sup	Volume of S-circular-insula-sup in the left hemisphere generated by parcellation of the white surface using Destrieux (a
0549	aparc-a2009s lh volume S-collat-transv-ant	Volume of S-collat-transv-ant in the left hemisphere generated by parcellation of the white surface using Destrieux (a20
0550	aparc-a2009s lh volume S-collat-transv-post	Volume of S-collat-transv-post in the left hemisphere generated by parcellation of the white surface using Destrieux (a2
0551	aparc-a2009s lh volume S-front-inf	Volume of S-front-inf in the left hemisphere generated by parcellation of the white surface using Destrieux (a2009s) parc
0552	aparc-a2009s lh volume S-front-middle	Volume of S-front-middle in the left hemisphere generated by parcellation of the white surface using Destrieux (a2009s)
0553	aparc-a2009s lh volume S-front-sup	Volume of S-front-sup in the left hemisphere generated by parcellation of the white surface using Destrieux (a2009s) pa
0554	aparc-a2009s lh volume S-interm-prim-Jensen	Volume of S-interm-prim-Jensen in the left hemisphere generated by parcellation of the white surface using Destrieux (
0555	aparc-a2009s lh volume S-intrapariet+P-trans	Volume of S-intrapariet+P-trans in the left hemisphere generated by parcellation of the white surface using Destrieux (a
0556	aparc-a2009s lh volume S-oc-middle+Lunatus	Volume of S-oc-middle+Lunatus in the left hemisphere generated by parcellation of the white surface using Destrieux (
0557	aparc-a2009s lh volume S-oc-sup+transversal	Volume of S-oc-sup+transversal in the left hemisphere generated by parcellation of the white surface using Destrieux (
0558	aparc-a2009s lh volume S-occipital-ant	Volume of S-occipital-ant in the left hemisphere generated by parcellation of the white surface using Destrieux (a2009s)
0559	aparc-a2009s lh volume S-oc-temp-lat	Volume of S-oc-temp-lat in the left hemisphere generated by parcellation of the white surface using Destrieux (a2009s)
0560	aparc-a2009s lh volume S-oc-temp-med+Lingual	Volume of S-oc-temp-med+Lingual in the left hemisphere generated by parcellation of the white surface using Destrieux
0561	aparc-a2009s lh volume S-orbital-lateral	Volume of S-orbital-lateral in the left hemisphere generated by parcellation of the white surface using Destrieux (a2009s
0562	aparc-a2009s lh volume S-orbital-med-olfact	Volume of S-orbital-med-olfact in the left hemisphere generated by parcellation of the white surface using Destrieux (a2
0563	aparc-a2009s lh volume S-orbital-H-Shaped	Volume of S-orbital-H-Shaped in the left hemisphere generated by parcellation of the white surface using Destrieux (a20
0564	aparc-a2009s lh volume S-parieto-occipital	Volume of S-parieto-occipital in the left hemisphere generated by parcellation of the white surface using Destrieux (a20
0565	aparc-a2009s lh volume S-pericallosal	Volume of S-pericallosal in the left hemisphere generated by parcellation of the white surface using Destrieux (a2009s)
0566	aparc-a2009s lh volume S-postcentral	Volume of S-postcentral in the left hemisphere generated by parcellation of the white surface using Destrieux (a2009s)
0567	aparc-a2009s lh volume S-precentral-inf-part	Volume of S-precentral-inf-part in the left hemisphere generated by parcellation of the white surface using Destrieux (a2
0568	aparc-a2009s lh volume S-precentral-sup-part	Volume of S-precentral-sup-part in the left hemisphere generated by parcellation of the white surface using Destrieux (

IDP number	IDP short name	IDP description
0569	aparc-a2009s lh volume S-suborbital	Volume of S-suborbital in the left hemisphere generated by parcellation of the white surface using Destrieux (a2009s) p
0570	aparc-a2009s lh volume S-subparietal	Volume of S-subparietal in the left hemisphere generated by parcellation of the white surface using Destrieux (a2009s)
0571	aparc-a2009s lh volume S-temporal-inf	Volume of S-temporal-inf in the left hemisphere generated by parcellation of the white surface using Destrieux (a2009s)
0572	aparc-a2009s lh volume S-temporal-sup	Volume of S-temporal-sup in the left hemisphere generated by parcellation of the white surface using Destrieux (a2009s
0573	aparc-a2009s lh volume S-temporal-transverse	Volume of S-temporal-transverse in the left hemisphere generated by parcellation of the white surface using Destrieux (
0574	aparc-a2009s rh volume G+S-frontomargin	Volume of G+S-frontomargin in the right hemisphere generated by parcellation of the white surface using Destrieux (a20
0575	aparc-a2009s rh volume G+S-occipital-inf	Volume of G+S-occipital-inf in the right hemisphere generated by parcellation of the white surface using Destrieux (a20
0576	aparc-a2009s rh volume G+S-paracentral	Volume of G+S-paracentral in the right hemisphere generated by parcellation of the white surface using Destrieux (a20
0577	aparc-a2009s rh volume G+S-subcentral	Volume of G+S-subcentral in the right hemisphere generated by parcellation of the white surface using Destrieux (a200
0578	aparc-a2009s rh volume G+S-transv-frontopol	Volume of G+S-transv-frontopol in the right hemisphere generated by parcellation of the white surface using Destrieux (
0579	aparc-a2009s rh volume G+S-cingul-Ant	Volume of G+S-cingul-Ant in the right hemisphere generated by parcellation of the white surface using Destrieux (a2009
0580	aparc-a2009s rh volume G+S-cingul-Mid-Ant	Volume of G+S-cingul-Mid-Ant in the right hemisphere generated by parcellation of the white surface using Destrieux (a
0581	aparc-a2009s rh volume G+S-cingul-Mid-Post	Volume of G+S-cingul-Mid-Post in the right hemisphere generated by parcellation of the white surface using Destrieux (
0582	aparc-a2009s rh volume G-cingul-Post-dorsal	Volume of G-cingul-Post-dorsal in the right hemisphere generated by parcellation of the white surface using Destrieux (
0583	aparc-a2009s rh volume G-cingul-Post-ventral	Volume of G-cingul-Post-ventral in the right hemisphere generated by parcellation of the white surface using Destrieux (
0584	aparc-a2009s rh volume G-cuneus	Volume of G-cuneus in the right hemisphere generated by parcellation of the white surface using Destrieux (a2009s) pa
0585	aparc-a2009s rh volume G-front-inf-Opercular	Volume of G-front-inf-Opercular in the right hemisphere generated by parcellation of the white surface using Destrieux (
0586	aparc-a2009s rh volume G-front-inf-Orbital	Volume of G-front-inf-Orbital in the right hemisphere generated by parcellation of the white surface using Destrieux (a20
0587	aparc-a2009s rh volume G-front-inf-Triangul	Volume of G-front-inf-Triangul in the right hemisphere generated by parcellation of the white surface using Destrieux (a2
0588	aparc-a2009s rh volume G-front-middle	Volume of G-front-middle in the right hemisphere generated by parcellation of the white surface using Destrieux (a2009
0589	aparc-a2009s rh volume G-front-sup	Volume of G-front-sup in the right hemisphere generated by parcellation of the white surface using Destrieux (a2009s) p
0590	aparc-a2009s rh volume G-Ins-lg+S-cent-ins	Volume of G-Ins-lg+S-cent-ins in the right hemisphere generated by parcellation of the white surface using Destrieux (a
0591	aparc-a2009s rh volume G-insular-short	Volume of G-insular-short in the right hemisphere generated by parcellation of the white surface using Destrieux (a2009
0592	aparc-a2009s rh volume G-occipital-middle	Volume of G-occipital-middle in the right hemisphere generated by parcellation of the white surface using Destrieux (a2
0593	aparc-a2009s rh volume G-occipital-sup	Volume of G-occipital-sup in the right hemisphere generated by parcellation of the white surface using Destrieux (a2009
0594	aparc-a2009s rh volume G-oc-temp-lat-fusifor	Volume of G-oc-temp-lat-fusifor in the right hemisphere generated by parcellation of the white surface using Destrieux (
0595	aparc-a2009s rh volume G-oc-temp-med-Lingual	Volume of G-oc-temp-med-Lingual in the right hemisphere generated by parcellation of the white surface using Destrieu
0596	aparc-a2009s rh volume G-oc-temp-med-Parahip	Volume of G-oc-temp-med-Parahip in the right hemisphere generated by parcellation of the white surface using Destrie
0597	aparc-a2009s rh volume G-orbital	Volume of G-orbital in the right hemisphere generated by parcellation of the white surface using Destrieux (a2009s) par
0598	aparc-a2009s rh volume G-pariet-inf-Angular	Volume of G-pariet-inf-Angular in the right hemisphere generated by parcellation of the white surface using Destrieux (a
0599	aparc-a2009s rh volume G-pariet-inf-Supramar	Volume of G-pariet-inf-Supramar in the right hemisphere generated by parcellation of the white surface using Destrieux
0600	aparc-a2009s rh volume G-parietal-sup	Volume of G-parietal-sup in the right hemisphere generated by parcellation of the white surface using Destrieux (a2009
0601	aparc-a2009s rh volume G-postcentral	Volume of G-postcentral in the right hemisphere generated by parcellation of the white surface using Destrieux (a2009s
0602	aparc-a2009s rh volume G-precentral	Volume of G-precentral in the right hemisphere generated by parcellation of the white surface using Destrieux (a2009s)
0603	aparc-a2009s rh volume G-precuneus	Volume of G-precuneus in the right hemisphere generated by parcellation of the white surface using Destrieux (a2009s)
0604	aparc-a2009s rh volume G-rectus	Volume of G-rectus in the right hemisphere generated by parcellation of the white surface using Destrieux (a2009s) par
0605	aparc-a2009s rh volume G-subcallosal	Volume of G-subcallosal in the right hemisphere generated by parcellation of the white surface using Destrieux (a2009s
0606	aparc-a2009s rh volume G-temp-sup-G-T-transv	Volume of G-temp-sup-G-T-transv in the right hemisphere generated by parcellation of the white surface using Destrieu
0607	aparc-a2009s rh volume G-temp-sup-Lateral	Volume of G-temp-sup-Lateral in the right hemisphere generated by parcellation of the white surface using Destrieux (a
0608	aparc-a2009s rh volume G-temp-sup-Plan-polar	Volume of G-temp-sup-Plan-polar in the right hemisphere generated by parcellation of the white surface using Destrieux
0609	aparc-a2009s rh volume G-temp-sup-Plan-tempo	Volume of G-temp-sup-Plan-tempo in the right hemisphere generated by parcellation of the white surface using Destrieu
0610	aparc-a2009s rh volume G-temporal-inf	Volume of G-temporal-inf in the right hemisphere generated by parcellation of the white surface using Destrieux (a2009
0611	aparc-a2009s rh volume G-temporal-middle	Volume of G-temporal-middle in the right hemisphere generated by parcellation of the white surface using Destrieux (a2
0612	aparc-a2009s rh volume Lat-Fis-ant-Horizont	Volume of Lat-Fis-ant-Horizont in the right hemisphere generated by parcellation of the white surface using Destrieux (a
0613	aparc-a2009s rh volume Lat-Fis-ant-Vertical	Volume of Lat-Fis-ant-Vertical in the right hemisphere generated by parcellation of the white surface using Destrieux (a2
0614	aparc-a2009s rh volume Lat-Fis-post	Volume of Lat-Fis-post in the right hemisphere generated by parcellation of the white surface using Destrieux (a2009s)
0615	aparc-a2009s rh volume Pole-occipital	Volume of Pole-occipital in the right hemisphere generated by parcellation of the white surface using Destrieux (a2009s
0616	aparc-a2009s rh volume Pole-temporal	Volume of Pole-temporal in the right hemisphere generated by parcellation of the white surface using Destrieux (a2009
0617	aparc-a2009s rh volume S-calcarine	Volume of S-calcarine in the right hemisphere generated by parcellation of the white surface using Destrieux (a2009s) p
0618	aparc-a2009s rh volume S-central	Volume of S-central in the right hemisphere generated by parcellation of the white surface using Destrieux (a2009s) par
0619	aparc-a2009s rh volume S-cingul-Marginalis	Volume of S-cingul-Marginalis in the right hemisphere generated by parcellation of the white surface using Destrieux (a2
0620	aparc-a2009s rh volume S-circular-insula-ant	Volume of S-circular-insula-ant in the right hemisphere generated by parcellation of the white surface using Destrieux (a
0621	aparc-a2009s rh volume S-circular-insula-inf	Volume of S-circular-insula-inf in the right hemisphere generated by parcellation of the white surface using Destrieux (a2
0622	aparc-a2009s rh volume S-circular-insula-sup	Volume of S-circular-insula-sup in the right hemisphere generated by parcellation of the white surface using Destrieux (
0623	aparc-a2009s rh volume S-collat-transv-ant	Volume of S-collat-transv-ant in the right hemisphere generated by parcellation of the white surface using Destrieux (a2
0624	aparc-a2009s rh volume S-collat-transv-post	Volume of S-collat-transv-post in the right hemisphere generated by parcellation of the white surface using Destrieux (a
0625	aparc-a2009s rh volume S-front-inf	Volume of S-front-inf in the right hemisphere generated by parcellation of the white surface using Destrieux (a2009s) pa

IDP number	IDP short name	IDP description
0626	aparc-a2009s rh volume S-front-middle	Volume of S-front-middle in the right hemisphere generated by parcellation of the white surface using Destrieux (a2009s
0627	aparc-a2009s rh volume S-front-sup	Volume of S-front-sup in the right hemisphere generated by parcellation of the white surface using Destrieux (a2009s) p
0628	aparc-a2009s rh volume S-interm-prim-Jensen	Volume of S-interm-prim-Jensen in the right hemisphere generated by parcellation of the white surface using Destrieux
0629	aparc-a2009s rh volume S-intrapariet+P-trans	Volume of S-intrapariet+P-trans in the right hemisphere generated by parcellation of the white surface using Destrieux (
0630	aparc-a2009s rh volume S-oc-middle+Lunatus	Volume of S-oc-middle+Lunatus in the right hemisphere generated by parcellation of the white surface using Destrieux (
0631	aparc-a2009s rh volume S-oc-sup+transversal	Volume of S-oc-sup+transversal in the right hemisphere generated by parcellation of the white surface using Destrieux (
0632	aparc-a2009s rh volume S-occipital-ant	Volume of S-occipital-ant in the right hemisphere generated by parcellation of the white surface using Destrieux (a2009s
0633	aparc-a2009s rh volume S-oc-temp-lat	Volume of S-oc-temp-lat in the right hemisphere generated by parcellation of the white surface using Destrieux (a2009s
0634	aparc-a2009s rh volume S-oc-temp-med+Lingual	Volume of S-oc-temp-med+Lingual in the right hemisphere generated by parcellation of the white surface using Destrieu
0635	aparc-a2009s rh volume S-orbital-lateral	Volume of S-orbital-lateral in the right hemisphere generated by parcellation of the white surface using Destrieux (a200
0636	aparc-a2009s rh volume S-orbital-med-olfact	Volume of S-orbital-med-olfact in the right hemisphere generated by parcellation of the white surface using Destrieux (a
0637	aparc-a2009s rh volume S-orbital-H-Shaped	Volume of S-orbital-H-Shaped in the right hemisphere generated by parcellation of the white surface using Destrieux (a:
0638	aparc-a2009s rh volume S-parieto-occipital	Volume of S-parieto-occipital in the right hemisphere generated by parcellation of the white surface using Destrieux (a2(
0639	aparc-a2009s rh volume S-pericallosal	Volume of S-pericallosal in the right hemisphere generated by parcellation of the white surface using Destrieux (a2009s
0640	aparc-a2009s rh volume S-postcentral	Volume of S-postcentral in the right hemisphere generated by parcellation of the white surface using Destrieux (a2009s)
0641	aparc-a2009s rh volume S-precentral-inf-part	Volume of S-precentral-inf-part in the right hemisphere generated by parcellation of the white surface using Destrieux (a
0642	aparc-a2009s rh volume S-precentral-sup-part	Volume of S-precentral-sup-part in the right hemisphere generated by parcellation of the white surface using Destrieux (
0643	aparc-a2009s rh volume S-suborbital	Volume of S-suborbital in the right hemisphere generated by parcellation of the white surface using Destrieux (a2009s)
0644	aparc-a2009s rh volume S-subparietal	Volume of S-subparietal in the right hemisphere generated by parcellation of the white surface using Destrieux (a2009s)
0645	aparc-a2009s rh volume S-temporal-inf	Volume of S-temporal-inf in the right hemisphere generated by parcellation of the white surface using Destrieux (a2009s
0646	aparc-a2009s rh volume S-temporal-sup	Volume of S-temporal-sup in the right hemisphere generated by parcellation of the white surface using Destrieux (a200
0647	aparc-a2009s rh volume S-temporal-transverse	Volume of S-temporal-transverse in the right hemisphere generated by parcellation of the white surface using Destrieux
0648	aparc-Desikan lh area TotalSurface	Area of TotalSurface in the left hemisphere generated by parcellation of the white surface using Desikan-Killiany parcell
0649	aparc-Desikan lh area bankssts	Area of bankssts in the left hemisphere generated by parcellation of the white surface using Desikan-Killiany parcellatio
0650	aparc-Desikan lh area caudalanteriorcingulate	Area of caudalanteriorcingulate in the left hemisphere generated by parcellation of the white surface using Desikan-Killi
0651	aparc-Desikan lh area caudalmiddlefrontal	Area of caudalmiddlefrontal in the left hemisphere generated by parcellation of the white surface using Desikan-Killiany
0652	aparc-Desikan lh area cuneus	Area of cuneus in the left hemisphere generated by parcellation of the white surface using Desikan-Killiany parcellation
0653	aparc-Desikan lh area entorhinal	Area of entorhinal in the left hemisphere generated by parcellation of the white surface using Desikan-Killiany parcellati
0654	aparc-Desikan lh area fusiform	Area of fusiform in the left hemisphere generated by parcellation of the white surface using Desikan-Killiany parcellation
0655	aparc-Desikan lh area inferiorparietal	Area of inferiorparietal in the left hemisphere generated by parcellation of the white surface using Desikan-Killiany parce
0656	aparc-Desikan lh area inferiortemporal	Area of inferiortemporal in the left hemisphere generated by parcellation of the white surface using Desikan-Killiany parc
0657	aparc-Desikan lh area isthmuscingulate	Area of isthmuscingulate in the left hemisphere generated by parcellation of the white surface using Desikan-Killiany pa
0658	aparc-Desikan lh area lateraloccipital	Area of lateraloccipital in the left hemisphere generated by parcellation of the white surface using Desikan-Killiany parce
0659	aparc-Desikan lh area lateralorbitofrontal	Area of lateralorbitofrontal in the left hemisphere generated by parcellation of the white surface using Desikan-Killiany p
0660	aparc-Desikan lh area lingual	Area of lingual in the left hemisphere generated by parcellation of the white surface using Desikan-Killiany parcellation
0661	aparc-Desikan lh area medialorbitofrontal	Area of medialorbitofrontal in the left hemisphere generated by parcellation of the white surface using Desikan-Killiany p
0662	aparc-Desikan lh area middletemporal	Area of middletemporal in the left hemisphere generated by parcellation of the white surface using Desikan-Killiany parc
0663	aparc-Desikan lh area parahippocampal	Area of parahippocampal in the left hemisphere generated by parcellation of the white surface using Desikan-Killiany pa
0664	aparc-Desikan lh area paracentral	Area of paracentral in the left hemisphere generated by parcellation of the white surface using Desikan-Killiany parcella
0665	aparc-Desikan lh area parsopercularis	Area of parsopercularis in the left hemisphere generated by parcellation of the white surface using Desikan-Killiany parc
0666	aparc-Desikan lh area parsorbitalis	Area of parsorbitalis in the left hemisphere generated by parcellation of the white surface using Desikan-Killiany parcella
0667	aparc-Desikan lh area parstriangularis	Area of parstriangularis in the left hemisphere generated by parcellation of the white surface using Desikan-Killiany parc
0668	aparc-Desikan lh area pericalcarine	Area of pericalcarine in the left hemisphere generated by parcellation of the white surface using Desikan-Killiany parcell
0669	aparc-Desikan lh area postcentral	Area of postcentral in the left hemisphere generated by parcellation of the white surface using Desikan-Killiany parcellat
0670	aparc-Desikan lh area posteriorcingulate	Area of posteriorcingulate in the left hemisphere generated by parcellation of the white surface using Desikan-Killiany pa
0671	aparc-Desikan lh area precentral	Area of precentral in the left hemisphere generated by parcellation of the white surface using Desikan-Killiany parcellati
0672	aparc-Desikan lh area precuneus	Area of precuneus in the left hemisphere generated by parcellation of the white surface using Desikan-Killiany parcellati
0673	aparc-Desikan lh area rostralanteriorcingulate	Area of rostralanteriorcingulate in the left hemisphere generated by parcellation of the white surface using Desikan-Killia
0674	aparc-Desikan lh area rostralmiddlefrontal	Area of rostralmiddlefrontal in the left hemisphere generated by parcellation of the white surface using Desikan-Killiany
0675	aparc-Desikan lh area superiorfrontal	Area of superiorfrontal in the left hemisphere generated by parcellation of the white surface using Desikan-Killiany parce
0676	aparc-Desikan lh area superiorparietal	Area of superiorparietal in the left hemisphere generated by parcellation of the white surface using Desikan-Killiany parc
0677	aparc-Desikan lh area superiortemporal	Area of superiortemporal in the left hemisphere generated by parcellation of the white surface using Desikan-Killiany pa
0678	aparc-Desikan lh area supramarginal	Area of supramarginal in the left hemisphere generated by parcellation of the white surface using Desikan-Killiany parce
0679	aparc-Desikan lh area frontalpole	Area of frontalpole in the left hemisphere generated by parcellation of the white surface using Desikan-Killiany parcellati
0680	aparc-Desikan lh area transversetemporal	Area of transversetemporal in the left hemisphere generated by parcellation of the white surface using Desikan-Killiany
0681	aparc-Desikan lh area insula	Area of insula in the left hemisphere generated by parcellation of the white surface using Desikan-Killiany parcellation
0682	aparc-Desikan rh area TotalSurface	Area of TotalSurface in the right hemisphere generated by parcellation of the white surface using Desikan-Killiany parce

IDP number	IDP short name	IDP description
0683	aparc-Desikan rh area bankssts	Area of bankssts in the right hemisphere generated by parcellation of the white surface using Desikan-Killiany parcellati
0684	aparc-Desikan rh area caudalanteriorcingulate	Area of caudalanteriorcingulate in the right hemisphere generated by parcellation of the white surface using Desikan-Kil
0685	aparc-Desikan rh area caudalmiddlefrontal	Area of caudalmiddlefrontal in the right hemisphere generated by parcellation of the white surface using Desikan-Killian
0686	aparc-Desikan rh area cuneus	Area of cuneus in the right hemisphere generated by parcellation of the white surface using Desikan-Killiany parcellatio
0687	aparc-Desikan rh area entorhinal	Area of entorhinal in the right hemisphere generated by parcellation of the white surface using Desikan-Killiany parcella
0688	aparc-Desikan rh area fusiform	Area of fusiform in the right hemisphere generated by parcellation of the white surface using Desikan-Killiany parcellatic
0689	aparc-Desikan rh area inferiorparietal	Area of inferiorparietal in the right hemisphere generated by parcellation of the white surface using Desikan-Killiany parc
0690	aparc-Desikan rh area inferiortemporal	Area of inferiortemporal in the right hemisphere generated by parcellation of the white surface using Desikan-Killiany pa
0691	aparc-Desikan rh area isthmuscingulate	Area of isthmuscingulate in the right hemisphere generated by parcellation of the white surface using Desikan-Killiany p
0692	aparc-Desikan rh area lateraloccipital	Area of lateraloccipital in the right hemisphere generated by parcellation of the white surface using Desikan-Killiany parc
0693	aparc-Desikan rh area lateralorbitofrontal	Area of lateralorbitofrontal in the right hemisphere generated by parcellation of the white surface using Desikan-Killiany
0694	aparc-Desikan rh area lingual	Area of lingual in the right hemisphere generated by parcellation of the white surface using Desikan-Killiany parcellation
0695	aparc-Desikan rh area medialorbitofrontal	Area of medialorbitofrontal in the right hemisphere generated by parcellation of the white surface using Desikan-Killiany
0696	aparc-Desikan rh area middletemporal	Area of middletemporal in the right hemisphere generated by parcellation of the white surface using Desikan-Killiany pa
0697	aparc-Desikan rh area parahippocampal	Area of parahippocampal in the right hemisphere generated by parcellation of the white surface using Desikan-Killiany p
0698	aparc-Desikan rh area paracentral	Area of paracentral in the right hemisphere generated by parcellation of the white surface using Desikan-Killiany parcell
0699	aparc-Desikan rh area parsopercularis	Area of parsopercularis in the right hemisphere generated by parcellation of the white surface using Desikan-Killiany pa
0700	aparc-Desikan rh area parsorbitalis	Area of parsorbitalis in the right hemisphere generated by parcellation of the white surface using Desikan-Killiany parce
0701	aparc-Desikan rh area parstriangularis	Area of parstriangularis in the right hemisphere generated by parcellation of the white surface using Desikan-Killiany pa
0702	aparc-Desikan rh area pericalcarine	Area of pericalcarine in the right hemisphere generated by parcellation of the white surface using Desikan-Killiany parce
0703	aparc-Desikan rh area postcentral	Area of postcentral in the right hemisphere generated by parcellation of the white surface using Desikan-Killiany parcell
0704	aparc-Desikan rh area posteriorcingulate	Area of posteriorcingulate in the right hemisphere generated by parcellation of the white surface using Desikan-Killiany
0705	aparc-Desikan rh area precentral	Area of precentral in the right hemisphere generated by parcellation of the white surface using Desikan-Killiany parcella
0706	aparc-Desikan rh area precuneus	Area of precuneus in the right hemisphere generated by parcellation of the white surface using Desikan-Killiany parcella
0707	aparc-Desikan rh area rostralanteriorcingulate	Area of rostralanteriorcingulate in the right hemisphere generated by parcellation of the white surface using Desikan-Kill
0708	aparc-Desikan rh area rostralmiddlefrontal	Area of rostralmiddlefrontal in the right hemisphere generated by parcellation of the white surface using Desikan-Killiany
0709	aparc-Desikan rh area superiorfrontal	Area of superiorfrontal in the right hemisphere generated by parcellation of the white surface using Desikan-Killiany parc
0710	aparc-Desikan rh area superiorparietal	Area of superiorparietal in the right hemisphere generated by parcellation of the white surface using Desikan-Killiany pa
0711	aparc-Desikan rh area superiortemporal	Area of superiortemporal in the right hemisphere generated by parcellation of the white surface using Desikan-Killiany p
0712	aparc-Desikan rh area supramarginal	Area of supramarginal in the right hemisphere generated by parcellation of the white surface using Desikan-Killiany parc
0713	aparc-Desikan rh area frontalpole	Area of frontalpole in the right hemisphere generated by parcellation of the white surface using Desikan-Killiany parcella
0714	aparc-Desikan rh area transversetemporal	Area of transversetemporal in the right hemisphere generated by parcellation of the white surface using Desikan-Killiany
0715	aparc-Desikan rh area insula	Area of insula in the right hemisphere generated by parcellation of the white surface using Desikan-Killiany parcellation
0716	aparc-pial lh area TotalSurface	Area of TotalSurface in the left hemisphere generated by parcellation of the pial surface using Desikan-Killiany parcellat
0717	aparc-pial lh area bankssts	Area of bankssts in the left hemisphere generated by parcellation of the pial surface using Desikan-Killiany parcellation
0718	aparc-pial lh area caudalanteriorcingulate	Area of caudalanteriorcingulate in the left hemisphere generated by parcellation of the pial surface using Desikan-Killiar
0719	aparc-pial lh area caudalmiddlefrontal	Area of caudalmiddlefrontal in the left hemisphere generated by parcellation of the pial surface using Desikan-Killiany pa
0720	aparc-pial lh area cuneus	Area of cuneus in the left hemisphere generated by parcellation of the pial surface using Desikan-Killiany parcellation
0721	aparc-pial lh area entorhinal	Area of entorhinal in the left hemisphere generated by parcellation of the pial surface using Desikan-Killiany parcellatior
0722	aparc-pial lh area fusiform	Area of fusiform in the left hemisphere generated by parcellation of the pial surface using Desikan-Killiany parcellation
0723	aparc-pial lh area inferiorparietal	Area of inferiorparietal in the left hemisphere generated by parcellation of the pial surface using Desikan-Killiany parcell
0724	aparc-pial lh area inferiortemporal	Area of inferiortemporal in the left hemisphere generated by parcellation of the pial surface using Desikan-Killiany parce
0725	aparc-pial lh area isthmuscingulate	Area of isthmuscingulate in the left hemisphere generated by parcellation of the pial surface using Desikan-Killiany parc
0726	aparc-pial lh area lateraloccipital	Area of lateraloccipital in the left hemisphere generated by parcellation of the pial surface using Desikan-Killiany parcell
0727	aparc-pial lh area lateralorbitofrontal	Area of lateralorbitofrontal in the left hemisphere generated by parcellation of the pial surface using Desikan-Killiany par
0728	aparc-pial lh area lingual	Area of lingual in the left hemisphere generated by parcellation of the pial surface using Desikan-Killiany parcellation
0729	aparc-pial lh area medialorbitofrontal	Area of medialorbitofrontal in the left hemisphere generated by parcellation of the pial surface using Desikan-Killiany pa
0730	aparc-pial lh area middletemporal	Area of middletemporal in the left hemisphere generated by parcellation of the pial surface using Desikan-Killiany parce
0731	aparc-pial lh area parahippocampal	Area of parahippocampal in the left hemisphere generated by parcellation of the pial surface using Desikan-Killiany parc
0732	aparc-pial lh area paracentral	Area of paracentral in the left hemisphere generated by parcellation of the pial surface using Desikan-Killiany parcellatic
0733	aparc-pial lh area parsopercularis	Area of parsopercularis in the left hemisphere generated by parcellation of the pial surface using Desikan-Killiany parce
0734	aparc-pial lh area parsorbitalis	Area of parsorbitalis in the left hemisphere generated by parcellation of the pial surface using Desikan-Killiany parcellati
0735	aparc-pial lh area parstriangularis	Area of parstriangularis in the left hemisphere generated by parcellation of the pial surface using Desikan-Killiany parce
0736	aparc-pial lh area pericalcarine	Area of pericalcarine in the left hemisphere generated by parcellation of the pial surface using Desikan-Killiany parcellat
0737	aparc-pial lh area postcentral	Area of postcentral in the left hemisphere generated by parcellation of the pial surface using Desikan-Killiany parcellatio
0738	aparc-pial lh area posteriorcingulate	Area of posteriorcingulate in the left hemisphere generated by parcellation of the pial surface using Desikan-Killiany par
0739	aparc-pial lh area precentral	Area of precentral in the left hemisphere generated by parcellation of the pial surface using Desikan-Killiany parcellatior

IDP number	IDP short name	IDP description
0740	aparc-pial lh area precuneus	Area of precuneus in the left hemisphere generated by parcellation of the pial surface using Desikan-Killiany parcellatio
0741	aparc-pial lh area rostralanteriorcingulate	Area of rostralanteriorcingulate in the left hemisphere generated by parcellation of the pial surface using Desikan-Killian
0742	aparc-pial lh area rostralmiddlefrontal	Area of rostralmiddlefrontal in the left hemisphere generated by parcellation of the pial surface using Desikan-Killiany pa
0743	aparc-pial lh area superiorfrontal	Area of superiorfrontal in the left hemisphere generated by parcellation of the pial surface using Desikan-Killiany parcell
0744	aparc-pial lh area superiorparietal	Area of superiorparietal in the left hemisphere generated by parcellation of the pial surface using Desikan-Killiany parce
0745	aparc-pial lh area superiortemporal	Area of superiortemporal in the left hemisphere generated by parcellation of the pial surface using Desikan-Killiany parc
0746	aparc-pial lh area supramarginal	Area of supramarginal in the left hemisphere generated by parcellation of the pial surface using Desikan-Killiany parcell
0747	aparc-pial lh area frontalpole	Area of frontalpole in the left hemisphere generated by parcellation of the pial surface using Desikan-Killiany parcellatio
0748	aparc-pial lh area transversetemporal	Area of transversetemporal in the left hemisphere generated by parcellation of the pial surface using Desikan-Killiany pa
0749	aparc-pial rh area TotalSurface	Area of TotalSurface in the right hemisphere generated by parcellation of the pial surface using Desikan-Killiany parcell
0750	aparc-pial rh area bankssts	Area of bankssts in the right hemisphere generated by parcellation of the pial surface using Desikan-Killiany parcellatio
0751	aparc-pial rh area caudalanteriorcingulate	Area of caudalanteriorcingulate in the right hemisphere generated by parcellation of the pial surface using Desikan-Killia
0752	aparc-pial rh area caudalmiddlefrontal	Area of caudalmiddlefrontal in the right hemisphere generated by parcellation of the pial surface using Desikan-Killiany
0753	aparc-pial rh area cuneus	Area of cuneus in the right hemisphere generated by parcellation of the pial surface using Desikan-Killiany parcellation
0754	aparc-pial rh area entorhinal	Area of entorhinal in the right hemisphere generated by parcellation of the pial surface using Desikan-Killiany parcellatic
0755	aparc-pial rh area fusiform	Area of fusiform in the right hemisphere generated by parcellation of the pial surface using Desikan-Killiany parcellation
0756	aparc-pial rh area inferiorparietal	Area of inferiorparietal in the right hemisphere generated by parcellation of the pial surface using Desikan-Killiany parce
0757	aparc-pial rh area inferiortemporal	Area of inferiortemporal in the right hemisphere generated by parcellation of the pial surface using Desikan-Killiany parc
0758	aparc-pial rh area isthmuscingulate	Area of isthmuscingulate in the right hemisphere generated by parcellation of the pial surface using Desikan-Killiany pa
0759	aparc-pial rh area lateraloccipital	Area of lateraloccipital in the right hemisphere generated by parcellation of the pial surface using Desikan-Killiany parce
0760	aparc-pial rh area lateralorbitofrontal	Area of lateralorbitofrontal in the right hemisphere generated by parcellation of the pial surface using Desikan-Killiany pa
0761	aparc-pial rh area lingual	Area of lingual in the right hemisphere generated by parcellation of the pial surface using Desikan-Killiany parcellation
0762	aparc-pial rh area medialorbitofrontal	Area of medialorbitofrontal in the right hemisphere generated by parcellation of the pial surface using Desikan-Killiany p
0763	aparc-pial rh area middletemporal	Area of middletemporal in the right hemisphere generated by parcellation of the pial surface using Desikan-Killiany parc
0764	aparc-pial rh area parahippocampal	Area of parahippocampal in the right hemisphere generated by parcellation of the pial surface using Desikan-Killiany pa
0765	aparc-pial rh area paracentral	Area of paracentral in the right hemisphere generated by parcellation of the pial surface using Desikan-Killiany parcellat
0766	aparc-pial rh area parsopercularis	Area of parsopercularis in the right hemisphere generated by parcellation of the pial surface using Desikan-Killiany parc
0767	aparc-pial rh area parsorbitalis	Area of parsorbitalis in the right hemisphere generated by parcellation of the pial surface using Desikan-Killiany parcella
0768	aparc-pial rh area parstriangularis	Area of parstriangularis in the right hemisphere generated by parcellation of the pial surface using Desikan-Killiany parc
0769	aparc-pial rh area pericalcarine	Area of pericalcarine in the right hemisphere generated by parcellation of the pial surface using Desikan-Killiany parcell
0770	aparc-pial rh area postcentral	Area of postcentral in the right hemisphere generated by parcellation of the pial surface using Desikan-Killiany parcellat
0771	aparc-pial rh area posteriorcingulate	Area of posteriorcingulate in the right hemisphere generated by parcellation of the pial surface using Desikan-Killiany pa
0772	aparc-pial rh area precentral	Area of precentral in the right hemisphere generated by parcellation of the pial surface using Desikan-Killiany parcellatic
0773	aparc-pial rh area precuneus	Area of precuneus in the right hemisphere generated by parcellation of the pial surface using Desikan-Killiany parcellati
0774	aparc-pial rh area rostralanteriorcingulate	Area of rostralanteriorcingulate in the right hemisphere generated by parcellation of the pial surface using Desikan-Killia
0775	aparc-pial rh area rostralmiddlefrontal	Area of rostralmiddlefrontal in the right hemisphere generated by parcellation of the pial surface using Desikan-Killiany p
0776	aparc-pial rh area superiorfrontal	Area of superiorfrontal in the right hemisphere generated by parcellation of the pial surface using Desikan-Killiany parce
0777	aparc-pial rh area superiorparietal	Area of superiorparietal in the right hemisphere generated by parcellation of the pial surface using Desikan-Killiany parc
0778	aparc-pial rh area superiortemporal	Area of superiortemporal in the right hemisphere generated by parcellation of the pial surface using Desikan-Killiany pa
0779	aparc-pial rh area supramarginal	Area of supramarginal in the right hemisphere generated by parcellation of the pial surface using Desikan-Killiany parce
0780	aparc-pial rh area frontalpole	Area of frontalpole in the right hemisphere generated by parcellation of the pial surface using Desikan-Killiany parcellati
0781	aparc-pial rh area transversetemporal	Area of transversetemporal in the right hemisphere generated by parcellation of the pial surface using Desikan-Killiany p
0782	BA-exvivo lh area BA1	Area of BA1 in the left hemisphere generated by parcellation of the white surface using BA_exvivo parcellation
0783	BA-exvivo lh area BA2	Area of BA2 in the left hemisphere generated by parcellation of the white surface using BA_exvivo parcellation
0784	BA-exvivo lh area BA3a	Area of BA3a in the left hemisphere generated by parcellation of the white surface using BA_exvivo parcellation
0785	BA-exvivo lh area BA3b	Area of BA3b in the left hemisphere generated by parcellation of the white surface using BA_exvivo parcellation
0786	BA-exvivo lh area BA4a	Area of BA4a in the left hemisphere generated by parcellation of the white surface using BA_exvivo parcellation
0787	BA-exvivo lh area BA4p	Area of BA4p in the left hemisphere generated by parcellation of the white surface using BA_exvivo parcellation
0788	BA-exvivo lh area BA6	Area of BA6 in the left hemisphere generated by parcellation of the white surface using BA_exvivo parcellation
0789	BA-exvivo lh area BA44	Area of BA44 in the left hemisphere generated by parcellation of the white surface using BA_exvivo parcellation
0790	BA-exvivo lh area BA45	Area of BA45 in the left hemisphere generated by parcellation of the white surface using BA_exvivo parcellation
0791	BA-exvivo lh area V1	Area of V1 in the left hemisphere generated by parcellation of the white surface using BA_exvivo parcellation
0792	BA-exvivo lh area V2	Area of V2 in the left hemisphere generated by parcellation of the white surface using BA_exvivo parcellation
0793	BA-exvivo lh area MT	Area of MT in the left hemisphere generated by parcellation of the white surface using BA_exvivo parcellation
0794	BA-exvivo lh area perirhinal	Area of perirhinal in the left hemisphere generated by parcellation of the white surface using BA_exvivo parcellation
0795	BA-exvivo lh area entorhinal	Area of entorhinal in the left hemisphere generated by parcellation of the white surface using BA_exvivo parcellation
0796	BA-exvivo rh area BA1	Area of BA1 in the right hemisphere generated by parcellation of the white surface using BA_exvivo parcellation

IDP number	IDP short name	IDP description
0797	BA-exvivo rh area BA2	Area of BA2 in the right hemisphere generated by parcellation of the white surface using BA_exvivo parcellation
0798	BA-exvivo rh area BA3a	Area of BA3a in the right hemisphere generated by parcellation of the white surface using BA_exvivo parcellation
0799	BA-exvivo rh area BA3b	Area of BA3b in the right hemisphere generated by parcellation of the white surface using BA_exvivo parcellation
0800	BA-exvivo rh area BA4a	Area of BA4a in the right hemisphere generated by parcellation of the white surface using BA_exvivo parcellation
0801	BA-exvivo rh area BA4p	Area of BA4p in the right hemisphere generated by parcellation of the white surface using BA_exvivo parcellation
0802	BA-exvivo rh area BA6	Area of BA6 in the right hemisphere generated by parcellation of the white surface using BA_exvivo parcellation
0803	BA-exvivo rh area BA44	Area of BA44 in the right hemisphere generated by parcellation of the white surface using BA_exvivo parcellation
0804	BA-exvivo rh area BA45	Area of BA45 in the right hemisphere generated by parcellation of the white surface using BA_exvivo parcellation
0805	BA-exvivo rh area V1	Area of V1 in the right hemisphere generated by parcellation of the white surface using BA_exvivo parcellation
0806	BA-exvivo rh area V2	Area of V2 in the right hemisphere generated by parcellation of the white surface using BA_exvivo parcellation
0807	BA-exvivo rh area MT	Area of MT in the right hemisphere generated by parcellation of the white surface using BA_exvivo parcellation
0808	BA-exvivo rh area perirhinal	Area of perirhinal in the right hemisphere generated by parcellation of the white surface using BA_exvivo parcellation
0809	BA-exvivo rh area entorhinal	Area of entorhinal in the right hemisphere generated by parcellation of the white surface using BA_exvivo parcellation
0810	aparc-DKTatlas lh area caudalanteriorcingulate	Area of caudalanteriorcingulate in the left hemisphere generated by parcellation of the white surface using DKT parcella
0811	aparc-DKTatlas lh area caudalmiddlefrontal	Area of caudalmiddlefrontal in the left hemisphere generated by parcellation of the white surface using DKT parcellation
0812	aparc-DKTatlas lh area cuneus	Area of cuneus in the left hemisphere generated by parcellation of the white surface using DKT parcellation
0813	aparc-DKTatlas lh area entorhinal	Area of entorhinal in the left hemisphere generated by parcellation of the white surface using DKT parcellation
0814	aparc-DKTatlas lh area fusiform	Area of fusiform in the left hemisphere generated by parcellation of the white surface using DKT parcellation
0815	aparc-DKTatlas lh area inferiorparietal	Area of inferiorparietal in the left hemisphere generated by parcellation of the white surface using DKT parcellation
0816	aparc-DKTatlas lh area inferiortemporal	Area of inferiortemporal in the left hemisphere generated by parcellation of the white surface using DKT parcellation
0817	aparc-DKTatlas lh area isthmuscingulate	Area of isthmuscingulate in the left hemisphere generated by parcellation of the white surface using DKT parcellation
0818	aparc-DKTatlas lh area lateraloccipital	Area of lateraloccipital in the left hemisphere generated by parcellation of the white surface using DKT parcellation
0819	aparc-DKTatlas lh area lateralorbitofrontal	Area of lateralorbitofrontal in the left hemisphere generated by parcellation of the white surface using DKT parcellation
0820	aparc-DKTatlas lh area lingual	Area of lingual in the left hemisphere generated by parcellation of the white surface using DKT parcellation
0821	aparc-DKTatlas lh area medialorbitofrontal	Area of medialorbitofrontal in the left hemisphere generated by parcellation of the white surface using DKT parcellation
0822	aparc-DKTatlas lh area middletemporal	Area of middletemporal in the left hemisphere generated by parcellation of the white surface using DKT parcellation
0823	aparc-DKTatlas lh area parahippocampal	Area of parahippocampal in the left hemisphere generated by parcellation of the white surface using DKT parcellation
0824	aparc-DKTatlas lh area paracentral	Area of paracentral in the left hemisphere generated by parcellation of the white surface using DKT parcellation
0825	aparc-DKTatlas lh area parsopercularis	Area of parsopercularis in the left hemisphere generated by parcellation of the white surface using DKT parcellation
0826	aparc-DKTatlas lh area parsorbitalis	Area of parsorbitalis in the left hemisphere generated by parcellation of the white surface using DKT parcellation
0827	aparc-DKTatlas lh area parstriangularis	Area of parstriangularis in the left hemisphere generated by parcellation of the white surface using DKT parcellation
0828	aparc-DKTatlas lh area pericalcarine	Area of pericalcarine in the left hemisphere generated by parcellation of the white surface using DKT parcellation
0829	aparc-DKTatlas lh area postcentral	Area of postcentral in the left hemisphere generated by parcellation of the white surface using DKT parcellation
0830	aparc-DKTatlas lh area posteriorcingulate	Area of posteriorcingulate in the left hemisphere generated by parcellation of the white surface using DKT parcellation
0831	aparc-DKTatlas lh area precentral	Area of precentral in the left hemisphere generated by parcellation of the white surface using DKT parcellation
0832	aparc-DKTatlas lh area precuneus	Area of precuneus in the left hemisphere generated by parcellation of the white surface using DKT parcellation
0833	aparc-DKTatlas lh area rostralanteriorcingulate	Area of rostralanteriorcingulate in the left hemisphere generated by parcellation of the white surface using DKT parcella
0834	aparc-DKTatlas lh area rostralmiddlefrontal	Area of rostralmiddlefrontal in the left hemisphere generated by parcellation of the white surface using DKT parcellation
0835	aparc-DKTatlas lh area superiorfrontal	Area of superiorfrontal in the left hemisphere generated by parcellation of the white surface using DKT parcellation
0836	aparc-DKTatlas lh area superiorparietal	Area of superiorparietal in the left hemisphere generated by parcellation of the white surface using DKT parcellation
0837	aparc-DKTatlas lh area superiortemporal	Area of superiortemporal in the left hemisphere generated by parcellation of the white surface using DKT parcellation
0838	aparc-DKTatlas lh area supramarginal	Area of supramarginal in the left hemisphere generated by parcellation of the white surface using DKT parcellation
0839	aparc-DKTatlas lh area transversetemporal	Area of transversetemporal in the left hemisphere generated by parcellation of the white surface using DKT parcellation
0840	aparc-DKTatlas lh area insula	Area of insula in the left hemisphere generated by parcellation of the white surface using DKT parcellation
0841	aparc-DKTatlas rh area caudalanteriorcingulate	Area of caudalanteriorcingulate in the right hemisphere generated by parcellation of the white surface using DKT parcel
0842	aparc-DKTatlas rh area caudalmiddlefrontal	Area of caudalmiddlefrontal in the right hemisphere generated by parcellation of the white surface using DKT parcellatic
0843	aparc-DKTatlas rh area cuneus	Area of cuneus in the right hemisphere generated by parcellation of the white surface using DKT parcellation
0844	aparc-DKTatlas rh area entorhinal	Area of entorhinal in the right hemisphere generated by parcellation of the white surface using DKT parcellation
0845	aparc-DKTatlas rh area fusiform	Area of fusiform in the right hemisphere generated by parcellation of the white surface using DKT parcellation
0846	aparc-DKTatlas rh area inferiorparietal	Area of inferiorparietal in the right hemisphere generated by parcellation of the white surface using DKT parcellation
0847	aparc-DKTatlas rh area inferiortemporal	Area of inferiortemporal in the right hemisphere generated by parcellation of the white surface using DKT parcellation
0848	aparc-DKTatlas rh area isthmuscingulate	Area of isthmuscingulate in the right hemisphere generated by parcellation of the white surface using DKT parcellation
0849	aparc-DKTatlas rh area lateraloccipital	Area of lateraloccipital in the right hemisphere generated by parcellation of the white surface using DKT parcellation
0850	aparc-DKTatlas rh area lateralorbitofrontal	Area of lateralorbitofrontal in the right hemisphere generated by parcellation of the white surface using DKT parcellation
0851	aparc-DKTatlas rh area lingual	Area of lingual in the right hemisphere generated by parcellation of the white surface using DKT parcellation
0852	aparc-DKTatlas rh area medialorbitofrontal	Area of medialorbitofrontal in the right hemisphere generated by parcellation of the white surface using DKT parcellatior
0853	aparc-DKTatlas rh area middletemporal	Area of middletemporal in the right hemisphere generated by parcellation of the white surface using DKT parcellation

IDP number	IDP short name	IDP description
0854	aparc-DKTatlas rh area parahippocampal	Area of parahippocampal in the right hemisphere generated by parcellation of the white surface using DKT parcellation
0855	aparc-DKTatlas rh area paracentral	Area of paracentral in the right hemisphere generated by parcellation of the white surface using DKT parcellation
0856	aparc-DKTatlas rh area parsopercularis	Area of parsopercularis in the right hemisphere generated by parcellation of the white surface using DKT parcellation
0857	aparc-DKTatlas rh area parsorbitalis	Area of parsorbitalis in the right hemisphere generated by parcellation of the white surface using DKT parcellation
0858	aparc-DKTatlas rh area parstriangularis	Area of parstriangularis in the right hemisphere generated by parcellation of the white surface using DKT parcellation
0859	aparc-DKTatlas rh area pericalcarine	Area of pericalcarine in the right hemisphere generated by parcellation of the white surface using DKT parcellation
0860	aparc-DKTatlas rh area postcentral	Area of postcentral in the right hemisphere generated by parcellation of the white surface using DKT parcellation
0861	aparc-DKTatlas rh area posteriorcingulate	Area of posteriorcingulate in the right hemisphere generated by parcellation of the white surface using DKT parcellation
0862	aparc-DKTatlas rh area precentral	Area of precentral in the right hemisphere generated by parcellation of the white surface using DKT parcellation
0863	aparc-DKTatlas rh area precuneus	Area of precuneus in the right hemisphere generated by parcellation of the white surface using DKT parcellation
0864	aparc-DKTatlas rh area rostralanteriorcingulate	Area of rostralanteriorcingulate in the right hemisphere generated by parcellation of the white surface using DKT parcell
0865	aparc-DKTatlas rh area rostralmiddlefrontal	Area of rostralmiddlefrontal in the right hemisphere generated by parcellation of the white surface using DKT parcellatio
0866	aparc-DKTatlas rh area superiorfrontal	Area of superiorfrontal in the right hemisphere generated by parcellation of the white surface using DKT parcellation
0867	aparc-DKTatlas rh area superiorparietal	Area of superiorparietal in the right hemisphere generated by parcellation of the white surface using DKT parcellation
0868	aparc-DKTatlas rh area superiortemporal	Area of superiortemporal in the right hemisphere generated by parcellation of the white surface using DKT parcellation
0869	aparc-DKTatlas rh area supramarginal	Area of supramarginal in the right hemisphere generated by parcellation of the white surface using DKT parcellation
0870	aparc-DKTatlas rh area transversetemporal	Area of transversetemporal in the right hemisphere generated by parcellation of the white surface using DKT parcellatio
0871	aparc-DKTatlas rh area insula	Area of insula in the right hemisphere generated by parcellation of the white surface using DKT parcellation
0872	aparc-a2009s lh area G+S-frontomargin	Area of G+S-frontomargin in the left hemisphere generated by parcellation of the white surface using Destrieux (a2009s
0873	aparc-a2009s lh area G+S-occipital-inf	Area of G+S-occipital-inf in the left hemisphere generated by parcellation of the white surface using Destrieux (a2009s)
0874	aparc-a2009s lh area G+S-paracentral	Area of G+S-paracentral in the left hemisphere generated by parcellation of the white surface using Destrieux (a2009s)
0875	aparc-a2009s lh area G+S-subcentral	Area of G+S-subcentral in the left hemisphere generated by parcellation of the white surface using Destrieux (a2009s) p
0876	aparc-a2009s lh area G+S-transv-frontopol	Area of G+S-transv-frontopol in the left hemisphere generated by parcellation of the white surface using Destrieux (a20
0877	aparc-a2009s lh area G+S-cingul-Ant	Area of G+S-cingul-Ant in the left hemisphere generated by parcellation of the white surface using Destrieux (a2009s) p
0878	aparc-a2009s lh area G+S-cingul-Mid-Ant	Area of G+S-cingul-Mid-Ant in the left hemisphere generated by parcellation of the white surface using Destrieux (a200
0879	aparc-a2009s lh area G+S-cingul-Mid-Post	Area of G+S-cingul-Mid-Post in the left hemisphere generated by parcellation of the white surface using Destrieux (a20
0880	aparc-a2009s lh area G-cingul-Post-dorsal	Area of G-cingul-Post-dorsal in the left hemisphere generated by parcellation of the white surface using Destrieux (a20
0881	aparc-a2009s lh area G-cingul-Post-ventral	Area of G-cingul-Post-ventral in the left hemisphere generated by parcellation of the white surface using Destrieux (a20
0882	aparc-a2009s lh area G-cuneus	Area of G-cuneus in the left hemisphere generated by parcellation of the white surface using Destrieux (a2009s) parcell
0883	aparc-a2009s lh area G-front-inf-Opercular	Area of G-front-inf-Opercular in the left hemisphere generated by parcellation of the white surface using Destrieux (a20
0884	aparc-a2009s lh area G-front-inf-Orbital	Area of G-front-inf-Orbital in the left hemisphere generated by parcellation of the white surface using Destrieux (a2009s
0885	aparc-a2009s lh area G-front-inf-Triangul	Area of G-front-inf-Triangul in the left hemisphere generated by parcellation of the white surface using Destrieux (a2009
0886	aparc-a2009s lh area G-front-middle	Area of G-front-middle in the left hemisphere generated by parcellation of the white surface using Destrieux (a2009s) pa
0887	aparc-a2009s lh area G-front-sup	Area of G-front-sup in the left hemisphere generated by parcellation of the white surface using Destrieux (a2009s) parce
0888	aparc-a2009s lh area G-Ins-lg+S-cent-ins	Area of G-Ins-lg+S-cent-ins in the left hemisphere generated by parcellation of the white surface using Destrieux (a200
0889	aparc-a2009s lh area G-insular-short	Area of G-insular-short in the left hemisphere generated by parcellation of the white surface using Destrieux (a2009s) p
0890	aparc-a2009s lh area G-occipital-middle	Area of G-occipital-middle in the left hemisphere generated by parcellation of the white surface using Destrieux (a2009s
0891	aparc-a2009s lh area G-occipital-sup	Area of G-occipital-sup in the left hemisphere generated by parcellation of the white surface using Destrieux (a2009s) p
0892	aparc-a2009s lh area G-oc-temp-lat-fusifor	Area of G-oc-temp-lat-fusifor in the left hemisphere generated by parcellation of the white surface using Destrieux (a20
0893	aparc-a2009s lh area G-oc-temp-med-Lingual	Area of G-oc-temp-med-Lingual in the left hemisphere generated by parcellation of the white surface using Destrieux (a
0894	aparc-a2009s lh area G-oc-temp-med-Parahip	Area of G-oc-temp-med-Parahip in the left hemisphere generated by parcellation of the white surface using Destrieux (
0895	aparc-a2009s lh area G-orbital	Area of G-orbital in the left hemisphere generated by parcellation of the white surface using Destrieux (a2009s) parcella
0896	aparc-a2009s lh area G-pariet-inf-Angular	Area of G-pariet-inf-Angular in the left hemisphere generated by parcellation of the white surface using Destrieux (a200
0897	aparc-a2009s lh area G-pariet-inf-Supramar	Area of G-pariet-inf-Supramar in the left hemisphere generated by parcellation of the white surface using Destrieux (a2
0898	aparc-a2009s lh area G-parietal-sup	Area of G-parietal-sup in the left hemisphere generated by parcellation of the white surface using Destrieux (a2009s) pa
0899	aparc-a2009s lh area G-postcentral	Area of G-postcentral in the left hemisphere generated by parcellation of the white surface using Destrieux (a2009s) pa
0900	aparc-a2009s lh area G-precentral	Area of G-precentral in the left hemisphere generated by parcellation of the white surface using Destrieux (a2009s) parc
0901	aparc-a2009s lh area G-precuneus	Area of G-precuneus in the left hemisphere generated by parcellation of the white surface using Destrieux (a2009s) par
0902	aparc-a2009s lh area G-rectus	Area of G-rectus in the left hemisphere generated by parcellation of the white surface using Destrieux (a2009s) parcella
0903	aparc-a2009s lh area G-subcallosal	Area of G-subcallosal in the left hemisphere generated by parcellation of the white surface using Destrieux (a2009s) pa
0904	aparc-a2009s lh area G-temp-sup-G-T-transv	Area of G-temp-sup-G-T-transv in the left hemisphere generated by parcellation of the white surface using Destrieux (a
0905	aparc-a2009s lh area G-temp-sup-Lateral	Area of G-temp-sup-Lateral in the left hemisphere generated by parcellation of the white surface using Destrieux (a200
0906	aparc-a2009s lh area G-temp-sup-Plan-polar	Area of G-temp-sup-Plan-polar in the left hemisphere generated by parcellation of the white surface using Destrieux (a2
0907	aparc-a2009s lh area G-temp-sup-Plan-tempo	Area of G-temp-sup-Plan-tempo in the left hemisphere generated by parcellation of the white surface using Destrieux (
0908	aparc-a2009s lh area G-temporal-inf	Area of G-temporal-inf in the left hemisphere generated by parcellation of the white surface using Destrieux (a2009s) pa
0909	aparc-a2009s lh area G-temporal-middle	Area of G-temporal-middle in the left hemisphere generated by parcellation of the white surface using Destrieux (a2009
0910	aparc-a2009s lh area Lat-Fis-ant-Horizont	Area of Lat-Fis-ant-Horizont in the left hemisphere generated by parcellation of the white surface using Destrieux (a200

IDP number	IDP short name	IDP description
0911	aparc-a2009s lh area Lat-Fis-ant-Vertical	Area of Lat-Fis-ant-Vertical in the left hemisphere generated by parcellation of the white surface using Destrieux (a2009
0912	aparc-a2009s lh area Lat-Fis-post	Area of Lat-Fis-post in the left hemisphere generated by parcellation of the white surface using Destrieux (a2009s) parc
0913	aparc-a2009s lh area Pole-occipital	Area of Pole-occipital in the left hemisphere generated by parcellation of the white surface using Destrieux (a2009s) pa
0914	aparc-a2009s lh area Pole-temporal	Area of Pole-temporal in the left hemisphere generated by parcellation of the white surface using Destrieux (a2009s) pa
0915	aparc-a2009s lh area S-calcarine	Area of S-calcarine in the left hemisphere generated by parcellation of the white surface using Destrieux (a2009s) parce
0916	aparc-a2009s lh area S-central	Area of S-central in the left hemisphere generated by parcellation of the white surface using Destrieux (a2009s) parcella
0917	aparc-a2009s lh area S-cingul-Marginalis	Area of S-cingul-Marginalis in the left hemisphere generated by parcellation of the white surface using Destrieux (a2009
0918	aparc-a2009s lh area S-circular-insula-ant	Area of S-circular-insula-ant in the left hemisphere generated by parcellation of the white surface using Destrieux (a200
0919	aparc-a2009s lh area S-circular-insula-inf	Area of S-circular-insula-inf in the left hemisphere generated by parcellation of the white surface using Destrieux (a2009
0920	aparc-a2009s lh area S-circular-insula-sup	Area of S-circular-insula-sup in the left hemisphere generated by parcellation of the white surface using Destrieux (a200
0921	aparc-a2009s lh area S-collat-transv-ant	Area of S-collat-transv-ant in the left hemisphere generated by parcellation of the white surface using Destrieux (a2009s
0922	aparc-a2009s lh area S-collat-transv-post	Area of S-collat-transv-post in the left hemisphere generated by parcellation of the white surface using Destrieux (a2009
0923	aparc-a2009s lh area S-front-inf	Area of S-front-inf in the left hemisphere generated by parcellation of the white surface using Destrieux (a2009s) parcell
0924	aparc-a2009s lh area S-front-middle	Area of S-front-middle in the left hemisphere generated by parcellation of the white surface using Destrieux (a2009s) pa
0925	aparc-a2009s lh area S-front-sup	Area of S-front-sup in the left hemisphere generated by parcellation of the white surface using Destrieux (a2009s) parce
0926	aparc-a2009s lh area S-interm-prim-Jensen	Area of S-interm-prim-Jensen in the left hemisphere generated by parcellation of the white surface using Destrieux (a20
0927	aparc-a2009s lh area S-intrapariet+P-trans	Area of S-intrapariet+P-trans in the left hemisphere generated by parcellation of the white surface using Destrieux (a20
0928	aparc-a2009s lh area S-oc-middle+Lunatus	Area of S-oc-middle+Lunatus in the left hemisphere generated by parcellation of the white surface using Destrieux (a20
0929	aparc-a2009s lh area S-oc-sup+transversal	Area of S-oc-sup+transversal in the left hemisphere generated by parcellation of the white surface using Destrieux (a20
0930	aparc-a2009s lh area S-occipital-ant	Area of S-occipital-ant in the left hemisphere generated by parcellation of the white surface using Destrieux (a2009s) pa
0931	aparc-a2009s lh area S-oc-temp-lat	Area of S-oc-temp-lat in the left hemisphere generated by parcellation of the white surface using Destrieux (a2009s) pa
0932	aparc-a2009s lh area S-oc-temp-med+Lingual	Area of S-oc-temp-med+Lingual in the left hemisphere generated by parcellation of the white surface using Destrieux (a
0933	aparc-a2009s lh area S-orbital-lateral	Area of S-orbital-lateral in the left hemisphere generated by parcellation of the white surface using Destrieux (a2009s) p
0934	aparc-a2009s lh area S-orbital-med-olfact	Area of S-orbital-med-olfact in the left hemisphere generated by parcellation of the white surface using Destrieux (a200
0935	aparc-a2009s lh area S-orbital-H-Shaped	Area of S-orbital-H-Shaped in the left hemisphere generated by parcellation of the white surface using Destrieux (a200
0936	aparc-a2009s lh area S-parieto-occipital	Area of S-parieto-occipital in the left hemisphere generated by parcellation of the white surface using Destrieux (a2009s
0937	aparc-a2009s lh area S-pericallosal	Area of S-pericallosal in the left hemisphere generated by parcellation of the white surface using Destrieux (a2009s) pa
0938	aparc-a2009s lh area S-postcentral	Area of S-postcentral in the left hemisphere generated by parcellation of the white surface using Destrieux (a2009s) pa
0939	aparc-a2009s lh area S-precentral-inf-part	Area of S-precentral-inf-part in the left hemisphere generated by parcellation of the white surface using Destrieux (a200
0940	aparc-a2009s lh area S-precentral-sup-part	Area of S-precentral-sup-part in the left hemisphere generated by parcellation of the white surface using Destrieux (a20
0941	aparc-a2009s lh area S-suborbital	Area of S-suborbital in the left hemisphere generated by parcellation of the white surface using Destrieux (a2009s) parc
0942	aparc-a2009s lh area S-subparietal	Area of S-subparietal in the left hemisphere generated by parcellation of the white surface using Destrieux (a2009s) pa
0943	aparc-a2009s lh area S-temporal-inf	Area of S-temporal-inf in the left hemisphere generated by parcellation of the white surface using Destrieux (a2009s) pa
0944	aparc-a2009s lh area S-temporal-sup	Area of S-temporal-sup in the left hemisphere generated by parcellation of the white surface using Destrieux (a2009s) p
0945	aparc-a2009s lh area S-temporal-transverse	Area of S-temporal-transverse in the left hemisphere generated by parcellation of the white surface using Destrieux (a2
0946	aparc-a2009s rh area G+S-frontomargin	Area of G+S-frontomargin in the right hemisphere generated by parcellation of the white surface using Destrieux (a200
0947	aparc-a2009s rh area G+S-occipital-inf	Area of G+S-occipital-inf in the right hemisphere generated by parcellation of the white surface using Destrieux (a2009s
0948	aparc-a2009s rh area G+S-paracentral	Area of G+S-paracentral in the right hemisphere generated by parcellation of the white surface using Destrieux (a2009s
0949	aparc-a2009s rh area G+S-subcentral	Area of G+S-subcentral in the right hemisphere generated by parcellation of the white surface using Destrieux (a2009s)
0950	aparc-a2009s rh area G+S-transv-frontopol	Area of G+S-transv-frontopol in the right hemisphere generated by parcellation of the white surface using Destrieux (a2
0951	aparc-a2009s rh area G+S-cingul-Ant	Area of G+S-cingul-Ant in the right hemisphere generated by parcellation of the white surface using Destrieux (a2009s)
0952	aparc-a2009s rh area G+S-cingul-Mid-Ant	Area of G+S-cingul-Mid-Ant in the right hemisphere generated by parcellation of the white surface using Destrieux (a20
0953	aparc-a2009s rh area G+S-cingul-Mid-Post	Area of G+S-cingul-Mid-Post in the right hemisphere generated by parcellation of the white surface using Destrieux (a2
0954	aparc-a2009s rh area G-cingul-Post-dorsal	Area of G-cingul-Post-dorsal in the right hemisphere generated by parcellation of the white surface using Destrieux (a20
0955	aparc-a2009s rh area G-cingul-Post-ventral	Area of G-cingul-Post-ventral in the right hemisphere generated by parcellation of the white surface using Destrieux (a2
0956	aparc-a2009s rh area G-cuneus	Area of G-cuneus in the right hemisphere generated by parcellation of the white surface using Destrieux (a2009s) parce
0957	aparc-a2009s rh area G-front-inf-Opercular	Area of G-front-inf-Opercular in the right hemisphere generated by parcellation of the white surface using Destrieux (a2
0958	aparc-a2009s rh area G-front-inf-Orbital	Area of G-front-inf-Orbital in the right hemisphere generated by parcellation of the white surface using Destrieux (a2009
0959	aparc-a2009s rh area G-front-inf-Triangul	Area of G-front-inf-Triangul in the right hemisphere generated by parcellation of the white surface using Destrieux (a200
0960	aparc-a2009s rh area G-front-middle	Area of G-front-middle in the right hemisphere generated by parcellation of the white surface using Destrieux (a2009s) p
0961	aparc-a2009s rh area G-front-sup	Area of G-front-sup in the right hemisphere generated by parcellation of the white surface using Destrieux (a2009s) par
0962	aparc-a2009s rh area G-Ins-lg+S-cent-ins	Area of G-Ins-lg+S-cent-ins in the right hemisphere generated by parcellation of the white surface using Destrieux (a20
0963	aparc-a2009s rh area G-insular-short	Area of G-insular-short in the right hemisphere generated by parcellation of the white surface using Destrieux (a2009s)
0964	aparc-a2009s rh area G-occipital-middle	Area of G-occipital-middle in the right hemisphere generated by parcellation of the white surface using Destrieux (a2009
0965	aparc-a2009s rh area G-occipital-sup	Area of G-occipital-sup in the right hemisphere generated by parcellation of the white surface using Destrieux (a2009s)
0966	aparc-a2009s rh area G-oc-temp-lat-fusifor	Area of G-oc-temp-lat-fusifor in the right hemisphere generated by parcellation of the white surface using Destrieux (a2
0967	aparc-a2009s rh area G-oc-temp-med-Lingual	Area of G-oc-temp-med-Lingual in the right hemisphere generated by parcellation of the white surface using Destrieux (

IDP number	IDP short name	IDP description
0968	aparc-a2009s rh area G-oc-temp-med-Parahip	Area of G-oc-temp-med-Parahip in the right hemisphere generated by parcellation of the white surface using Destrieux (
0969	aparc-a2009s rh area G-orbital	Area of G-orbital in the right hemisphere generated by parcellation of the white surface using Destrieux (a2009s) parcel
0970	aparc-a2009s rh area G-pariet-inf-Angular	Area of G-pariet-inf-Angular in the right hemisphere generated by parcellation of the white surface using Destrieux (a20
0971	aparc-a2009s rh area G-pariet-inf-Supramar	Area of G-pariet-inf-Supramar in the right hemisphere generated by parcellation of the white surface using Destrieux (a2
0972	aparc-a2009s rh area G-parietal-sup	Area of G-parietal-sup in the right hemisphere generated by parcellation of the white surface using Destrieux (a2009s) p
0973	aparc-a2009s rh area G-postcentral	Area of G-postcentral in the right hemisphere generated by parcellation of the white surface using Destrieux (a2009s) pa
0974	aparc-a2009s rh area G-precentral	Area of G-precentral in the right hemisphere generated by parcellation of the white surface using Destrieux (a2009s) pa
0975	aparc-a2009s rh area G-precuneus	Area of G-precuneus in the right hemisphere generated by parcellation of the white surface using Destrieux (a2009s) pa
0976	aparc-a2009s rh area G-rectus	Area of G-rectus in the right hemisphere generated by parcellation of the white surface using Destrieux (a2009s) parcel
0977	aparc-a2009s rh area G-subcallosal	Area of G-subcallosal in the right hemisphere generated by parcellation of the white surface using Destrieux (a2009s) p
0978	aparc-a2009s rh area G-temp-sup-G-T-transv	Area of G-temp-sup-G-T-transv in the right hemisphere generated by parcellation of the white surface using Destrieux (a
0979	aparc-a2009s rh area G-temp-sup-Lateral	Area of G-temp-sup-Lateral in the right hemisphere generated by parcellation of the white surface using Destrieux (a200
0980	aparc-a2009s rh area G-temp-sup-Plan-polar	Area of G-temp-sup-Plan-polar in the right hemisphere generated by parcellation of the white surface using Destrieux (a
0981	aparc-a2009s rh area G-temp-sup-Plan-tempo	Area of G-temp-sup-Plan-tempo in the right hemisphere generated by parcellation of the white surface using Destrieux (
0982	aparc-a2009s rh area G-temporal-inf	Area of G-temporal-inf in the right hemisphere generated by parcellation of the white surface using Destrieux (a2009s) p
0983	aparc-a2009s rh area G-temporal-middle	Area of G-temporal-middle in the right hemisphere generated by parcellation of the white surface using Destrieux (a200
0984	aparc-a2009s rh area Lat-Fis-ant-Horizont	Area of Lat-Fis-ant-Horizont in the right hemisphere generated by parcellation of the white surface using Destrieux (a20
0985	aparc-a2009s rh area Lat-Fis-ant-Vertical	Area of Lat-Fis-ant-Vertical in the right hemisphere generated by parcellation of the white surface using Destrieux (a200
0986	aparc-a2009s rh area Lat-Fis-post	Area of Lat-Fis-post in the right hemisphere generated by parcellation of the white surface using Destrieux (a2009s) par
0987	aparc-a2009s rh area Pole-occipital	Area of Pole-occipital in the right hemisphere generated by parcellation of the white surface using Destrieux (a2009s) pa
0988	aparc-a2009s rh area Pole-temporal	Area of Pole-temporal in the right hemisphere generated by parcellation of the white surface using Destrieux (a2009s) p
0989	aparc-a2009s rh area S-calcarine	Area of S-calcarine in the right hemisphere generated by parcellation of the white surface using Destrieux (a2009s) parc
0990	aparc-a2009s rh area S-central	Area of S-central in the right hemisphere generated by parcellation of the white surface using Destrieux (a2009s) parcel
0991	aparc-a2009s rh area S-cingul-Marginalis	Area of S-cingul-Marginalis in the right hemisphere generated by parcellation of the white surface using Destrieux (a200
0992	aparc-a2009s rh area S-circular-insula-ant	Area of S-circular-insula-ant in the right hemisphere generated by parcellation of the white surface using Destrieux (a20
0993	aparc-a2009s rh area S-circular-insula-inf	Area of S-circular-insula-inf in the right hemisphere generated by parcellation of the white surface using Destrieux (a200
0994	aparc-a2009s rh area S-circular-insula-sup	Area of S-circular-insula-sup in the right hemisphere generated by parcellation of the white surface using Destrieux (a20
0995	aparc-a2009s rh area S-collat-transv-ant	Area of S-collat-transv-ant in the right hemisphere generated by parcellation of the white surface using Destrieux (a2009
0996	aparc-a2009s rh area S-collat-transv-post	Area of S-collat-transv-post in the right hemisphere generated by parcellation of the white surface using Destrieux (a200
0997	aparc-a2009s rh area S-front-inf	Area of S-front-inf in the right hemisphere generated by parcellation of the white surface using Destrieux (a2009s) parce
0998	aparc-a2009s rh area S-front-middle	Area of S-front-middle in the right hemisphere generated by parcellation of the white surface using Destrieux (a2009s) p
0999	aparc-a2009s rh area S-front-sup	Area of S-front-sup in the right hemisphere generated by parcellation of the white surface using Destrieux (a2009s) parc
1000	aparc-a2009s rh area S-interm-prim-Jensen	Area of S-interm-prim-Jensen in the right hemisphere generated by parcellation of the white surface using Destrieux (a2
1001	aparc-a2009s rh area S-intrapariet+P-trans	Area of S-intrapariet+P-trans in the right hemisphere generated by parcellation of the white surface using Destrieux (a2
1002	aparc-a2009s rh area S-oc-middle+Lunatus	Area of S-oc-middle+Lunatus in the right hemisphere generated by parcellation of the white surface using Destrieux (a2
1003	aparc-a2009s rh area S-oc-sup+transversal	Area of S-oc-sup+transversal in the right hemisphere generated by parcellation of the white surface using Destrieux (a2
1004	aparc-a2009s rh area S-occipital-ant	Area of S-occipital-ant in the right hemisphere generated by parcellation of the white surface using Destrieux (a2009s) p
1005	aparc-a2009s rh area S-oc-temp-lat	Area of S-oc-temp-lat in the right hemisphere generated by parcellation of the white surface using Destrieux (a2009s) pa
1006	aparc-a2009s rh area S-oc-temp-med+Lingual	Area of S-oc-temp-med+Lingual in the right hemisphere generated by parcellation of the white surface using Destrieux (
1007	aparc-a2009s rh area S-orbital-lateral	Area of S-orbital-lateral in the right hemisphere generated by parcellation of the white surface using Destrieux (a2009s)
1008	aparc-a2009s rh area S-orbital-med-olfact	Area of S-orbital-med-olfact in the right hemisphere generated by parcellation of the white surface using Destrieux (a200
1009	aparc-a2009s rh area S-orbital-H-Shaped	Area of S-orbital-H-Shaped in the right hemisphere generated by parcellation of the white surface using Destrieux (a200
1010	aparc-a2009s rh area S-parieto-occipital	Area of S-parieto-occipital in the right hemisphere generated by parcellation of the white surface using Destrieux (a2009
1011	aparc-a2009s rh area S-pericallosal	Area of S-pericallosal in the right hemisphere generated by parcellation of the white surface using Destrieux (a2009s) p
1012	aparc-a2009s rh area S-postcentral	Area of S-postcentral in the right hemisphere generated by parcellation of the white surface using Destrieux (a2009s) pa
1013	aparc-a2009s rh area S-precentral-inf-part	Area of S-precentral-inf-part in the right hemisphere generated by parcellation of the white surface using Destrieux (a20
1014	aparc-a2009s rh area S-precentral-sup-part	Area of S-precentral-sup-part in the right hemisphere generated by parcellation of the white surface using Destrieux (a2
1015	aparc-a2009s rh area S-suborbital	Area of S-suborbital in the right hemisphere generated by parcellation of the white surface using Destrieux (a2009s) par
1016	aparc-a2009s rh area S-subparietal	Area of S-subparietal in the right hemisphere generated by parcellation of the white surface using Destrieux (a2009s) pa
1017	aparc-a2009s rh area S-temporal-inf	Area of S-temporal-inf in the right hemisphere generated by parcellation of the white surface using Destrieux (a2009s) p
1018	aparc-a2009s rh area S-temporal-sup	Area of S-temporal-sup in the right hemisphere generated by parcellation of the white surface using Destrieux (a2009s)
1019	aparc-a2009s rh area S-temporal-transverse	Area of S-temporal-transverse in the right hemisphere generated by parcellation of the white surface using Destrieux (a
1020	aparc-Desikan lh thickness GlobalMeanThickness	Mean thickness of GlobalMeanMean thickness in the left hemisphere generated by parcellation of the white surface usi
1021	aparc-Desikan lh thickness bankssts	Mean thickness of bankssts in the left hemisphere generated by parcellation of the white surface using Desikan-Killiany
1022	aparc-Desikan lh thickness caudalanteriorcingulate	Mean thickness of caudalanteriorcingulate in the left hemisphere generated by parcellation of the white surface using D
1023	aparc-Desikan lh thickness caudalmiddlefrontal	Mean thickness of caudalmiddlefrontal in the left hemisphere generated by parcellation of the white surface using Desik
1024	aparc-Desikan lh thickness cuneus	Mean thickness of cuneus in the left hemisphere generated by parcellation of the white surface using Desikan-Killiany p

IDP number	IDP short name	IDP description
1025	aparc-Desikan lh thickness entorhinal	Mean thickness of entorhinal in the left hemisphere generated by parcellation of the white surface using Desikan-Killiany
1026	aparc-Desikan lh thickness fusiform	Mean thickness of fusiform in the left hemisphere generated by parcellation of the white surface using Desikan-Killiany p
1027	aparc-Desikan lh thickness inferiorparietal	Mean thickness of inferiorparietal in the left hemisphere generated by parcellation of the white surface using Desikan-Ki
1028	aparc-Desikan lh thickness inferiortemporal	Mean thickness of inferiortemporal in the left hemisphere generated by parcellation of the white surface using Desikan-K
1029	aparc-Desikan lh thickness isthmuscingulate	Mean thickness of isthmuscingulate in the left hemisphere generated by parcellation of the white surface using Desikan
1030	aparc-Desikan lh thickness lateraloccipital	Mean thickness of lateraloccipital in the left hemisphere generated by parcellation of the white surface using Desikan-Ki
1031	aparc-Desikan lh thickness lateralorbitofrontal	Mean thickness of lateralorbitofrontal in the left hemisphere generated by parcellation of the white surface using Desika
1032	aparc-Desikan lh thickness lingual	Mean thickness of lingual in the left hemisphere generated by parcellation of the white surface using Desikan-Killiany pa
1033	aparc-Desikan lh thickness medialorbitofrontal	Mean thickness of medialorbitofrontal in the left hemisphere generated by parcellation of the white surface using Desika
1034	aparc-Desikan lh thickness middletemporal	Mean thickness of middletemporal in the left hemisphere generated by parcellation of the white surface using Desikan-K
1035	aparc-Desikan lh thickness parahippocampal	Mean thickness of parahippocampal in the left hemisphere generated by parcellation of the white surface using Desikan
1036	aparc-Desikan lh thickness paracentral	Mean thickness of paracentral in the left hemisphere generated by parcellation of the white surface using Desikan-Killia
1037	aparc-Desikan lh thickness parsopercularis	Mean thickness of parsopercularis in the left hemisphere generated by parcellation of the white surface using Desikan-K
1038	aparc-Desikan lh thickness parsorbitalis	Mean thickness of parsorbitalis in the left hemisphere generated by parcellation of the white surface using Desikan-Killia
1039	aparc-Desikan lh thickness parstriangularis	Mean thickness of parstriangularis in the left hemisphere generated by parcellation of the white surface using Desikan-K
1040	aparc-Desikan lh thickness pericalcarine	Mean thickness of pericalcarine in the left hemisphere generated by parcellation of the white surface using Desikan-Killi
1041	aparc-Desikan lh thickness postcentral	Mean thickness of postcentral in the left hemisphere generated by parcellation of the white surface using Desikan-Killian
1042	aparc-Desikan lh thickness posteriorcingulate	Mean thickness of posteriorcingulate in the left hemisphere generated by parcellation of the white surface using Desikan
1043	aparc-Desikan lh thickness precentral	Mean thickness of precentral in the left hemisphere generated by parcellation of the white surface using Desikan-Killiany
1044	aparc-Desikan lh thickness precuneus	Mean thickness of precuneus in the left hemisphere generated by parcellation of the white surface using Desikan-Killian
1045	aparc-Desikan lh thickness rostralanteriorcingulate	Mean thickness of rostralanteriorcingulate in the left hemisphere generated by parcellation of the white surface using De
1046	aparc-Desikan lh thickness rostralmiddlefrontal	Mean thickness of rostralmiddlefrontal in the left hemisphere generated by parcellation of the white surface using Desika
1047	aparc-Desikan lh thickness superiorfrontal	Mean thickness of superiorfrontal in the left hemisphere generated by parcellation of the white surface using Desikan-Ki
1048	aparc-Desikan lh thickness superiorparietal	Mean thickness of superiorparietal in the left hemisphere generated by parcellation of the white surface using Desikan-K
1049	aparc-Desikan lh thickness superiortemporal	Mean thickness of superiortemporal in the left hemisphere generated by parcellation of the white surface using Desikan
1050	aparc-Desikan lh thickness supramarginal	Mean thickness of supramarginal in the left hemisphere generated by parcellation of the white surface using Desikan-Ki
1051	aparc-Desikan lh thickness frontalpole	Mean thickness of frontalpole in the left hemisphere generated by parcellation of the white surface using Desikan-Killian
1052	aparc-Desikan lh thickness transversetemporal	Mean thickness of transversetemporal in the left hemisphere generated by parcellation of the white surface using Desik
1053	aparc-Desikan lh thickness insula	Mean thickness of insula in the left hemisphere generated by parcellation of the white surface using Desikan-Killiany pa
1054	aparc-Desikan rh thickness GlobalMeanThickness	Mean thickness of GlobalMeanMean thickness in the right hemisphere generated by parcellation of the white surface us
1055	aparc-Desikan rh thickness bankssts	Mean thickness of bankssts in the right hemisphere generated by parcellation of the white surface using Desikan-Killian
1056	aparc-Desikan rh thickness caudalanteriorcingulate	Mean thickness of caudalanteriorcingulate in the right hemisphere generated by parcellation of the white surface using I
1057	aparc-Desikan rh thickness caudalmiddlefrontal	Mean thickness of caudalmiddlefrontal in the right hemisphere generated by parcellation of the white surface using Des
1058	aparc-Desikan rh thickness cuneus	Mean thickness of cuneus in the right hemisphere generated by parcellation of the white surface using Desikan-Killiany
1059	aparc-Desikan rh thickness entorhinal	Mean thickness of entorhinal in the right hemisphere generated by parcellation of the white surface using Desikan-Killia
1060	aparc-Desikan rh thickness fusiform	Mean thickness of fusiform in the right hemisphere generated by parcellation of the white surface using Desikan-Killiany
1061	aparc-Desikan rh thickness inferiorparietal	Mean thickness of inferiorparietal in the right hemisphere generated by parcellation of the white surface using Desikan-K
1062	aparc-Desikan rh thickness inferiortemporal	Mean thickness of inferiortemporal in the right hemisphere generated by parcellation of the white surface using Desikan
1063	aparc-Desikan rh thickness isthmuscingulate	Mean thickness of isthmuscingulate in the right hemisphere generated by parcellation of the white surface using Desika
1064	aparc-Desikan rh thickness lateraloccipital	Mean thickness of lateraloccipital in the right hemisphere generated by parcellation of the white surface using Desikan-K
1065	aparc-Desikan rh thickness lateralorbitofrontal	Mean thickness of lateralorbitofrontal in the right hemisphere generated by parcellation of the white surface using Desik
1066	aparc-Desikan rh thickness lingual	Mean thickness of lingual in the right hemisphere generated by parcellation of the white surface using Desikan-Killiany p
1067	aparc-Desikan rh thickness medialorbitofrontal	Mean thickness of medialorbitofrontal in the right hemisphere generated by parcellation of the white surface using Desik
1068	aparc-Desikan rh thickness middletemporal	Mean thickness of middletemporal in the right hemisphere generated by parcellation of the white surface using Desikan
1069	aparc-Desikan rh thickness parahippocampal	Mean thickness of parahippocampal in the right hemisphere generated by parcellation of the white surface using Desika
1070	aparc-Desikan rh thickness paracentral	Mean thickness of paracentral in the right hemisphere generated by parcellation of the white surface using Desikan-Killi
1071	aparc-Desikan rh thickness parsopercularis	Mean thickness of parsopercularis in the right hemisphere generated by parcellation of the white surface using Desikan
1072	aparc-Desikan rh thickness parsorbitalis	Mean thickness of parsorbitalis in the right hemisphere generated by parcellation of the white surface using Desikan-Kil
1073	aparc-Desikan rh thickness parstriangularis	Mean thickness of parstriangularis in the right hemisphere generated by parcellation of the white surface using Desikan
1074	aparc-Desikan rh thickness pericalcarine	Mean thickness of pericalcarine in the right hemisphere generated by parcellation of the white surface using Desikan-Ki
1075	aparc-Desikan rh thickness postcentral	Mean thickness of postcentral in the right hemisphere generated by parcellation of the white surface using Desikan-Killi
1076	aparc-Desikan rh thickness posteriorcingulate	Mean thickness of posteriorcingulate in the right hemisphere generated by parcellation of the white surface using Desika
1077	aparc-Desikan rh thickness precentral	Mean thickness of precentral in the right hemisphere generated by parcellation of the white surface using Desikan-Killia
1078	aparc-Desikan rh thickness precuneus	Mean thickness of precuneus in the right hemisphere generated by parcellation of the white surface using Desikan-Killia
1079	aparc-Desikan rh thickness rostralanteriorcingulate	Mean thickness of rostralanteriorcingulate in the right hemisphere generated by parcellation of the white surface using D
1080	aparc-Desikan rh thickness rostralmiddlefrontal	Mean thickness of rostralmiddlefrontal in the right hemisphere generated by parcellation of the white surface using Desi
1081	aparc-Desikan rh thickness superiorfrontal	Mean thickness of superiorfrontal in the right hemisphere generated by parcellation of the white surface using Desikan-I

IDP number	IDP short name	IDP description
1082	aparc-Desikan rh thickness superiorparietal	Mean thickness of superiorparietal in the right hemisphere generated by parcellation of the white surface using Desikan
1083	aparc-Desikan rh thickness superiortemporal	Mean thickness of superiortemporal in the right hemisphere generated by parcellation of the white surface using Desika
1084	aparc-Desikan rh thickness supramarginal	Mean thickness of supramarginal in the right hemisphere generated by parcellation of the white surface using Desikan-I
1085	aparc-Desikan rh thickness frontalpole	Mean thickness of frontalpole in the right hemisphere generated by parcellation of the white surface using Desikan-Killia
1086	aparc-Desikan rh thickness transversetemporal	Mean thickness of transversetemporal in the right hemisphere generated by parcellation of the white surface using Desi
1087	aparc-Desikan rh thickness insula	Mean thickness of insula in the right hemisphere generated by parcellation of the white surface using Desikan-Killiany p
1088	BA-exvivo lh thickness BA1	Mean thickness of BA1 in the left hemisphere generated by parcellation of the white surface using BA_exvivo parcellatio
1089	BA-exvivo lh thickness BA2	Mean thickness of BA2 in the left hemisphere generated by parcellation of the white surface using BA_exvivo parcellatio
1090	BA-exvivo lh thickness BA3a	Mean thickness of BA3a in the left hemisphere generated by parcellation of the white surface using BA_exvivo parcellat
1091	BA-exvivo lh thickness BA3b	Mean thickness of BA3b in the left hemisphere generated by parcellation of the white surface using BA_exvivo parcellat
1092	BA-exvivo lh thickness BA4a	Mean thickness of BA4a in the left hemisphere generated by parcellation of the white surface using BA_exvivo parcellat
1093	BA-exvivo lh thickness BA4p	Mean thickness of BA4p in the left hemisphere generated by parcellation of the white surface using BA_exvivo parcellat
1094	BA-exvivo lh thickness BA6	Mean thickness of BA6 in the left hemisphere generated by parcellation of the white surface using BA_exvivo parcellatio
1095	BA-exvivo lh thickness BA44	Mean thickness of BA44 in the left hemisphere generated by parcellation of the white surface using BA_exvivo parcellat
1096	BA-exvivo lh thickness BA45	Mean thickness of BA45 in the left hemisphere generated by parcellation of the white surface using BA_exvivo parcellat
1097	BA-exvivo lh thickness V1	Mean thickness of V1 in the left hemisphere generated by parcellation of the white surface using BA_exvivo parcellation
1098	BA-exvivo lh thickness V2	Mean thickness of V2 in the left hemisphere generated by parcellation of the white surface using BA_exvivo parcellation
1099	BA-exvivo lh thickness MT	Mean thickness of MT in the left hemisphere generated by parcellation of the white surface using BA_exvivo parcellation
1100	BA-exvivo lh thickness perirhinal	Mean thickness of perirhinal in the left hemisphere generated by parcellation of the white surface using BA_exvivo parc
1101	BA-exvivo lh thickness entorhinal	Mean thickness of entorhinal in the left hemisphere generated by parcellation of the white surface using BA_exvivo parc
1102	BA-exvivo rh thickness BA1	Mean thickness of BA1 in the right hemisphere generated by parcellation of the white surface using BA_exvivo parcellat
1103	BA-exvivo rh thickness BA2	Mean thickness of BA2 in the right hemisphere generated by parcellation of the white surface using BA_exvivo parcellat
1104	BA-exvivo rh thickness BA3a	Mean thickness of BA3a in the right hemisphere generated by parcellation of the white surface using BA_exvivo parcell
1105	BA-exvivo rh thickness BA3b	Mean thickness of BA3b in the right hemisphere generated by parcellation of the white surface using BA_exvivo parcell
1106	BA-exvivo rh thickness BA4a	Mean thickness of BA4a in the right hemisphere generated by parcellation of the white surface using BA_exvivo parcell
1107	BA-exvivo rh thickness BA4p	Mean thickness of BA4p in the right hemisphere generated by parcellation of the white surface using BA_exvivo parcell
1108	BA-exvivo rh thickness BA6	Mean thickness of BA6 in the right hemisphere generated by parcellation of the white surface using BA_exvivo parcellat
1109	BA-exvivo rh thickness BA44	Mean thickness of BA44 in the right hemisphere generated by parcellation of the white surface using BA_exvivo parcell
1110	BA-exvivo rh thickness BA45	Mean thickness of BA45 in the right hemisphere generated by parcellation of the white surface using BA_exvivo parcell
1111	BA-exvivo rh thickness V1	Mean thickness of V1 in the right hemisphere generated by parcellation of the white surface using BA_exvivo parcellatio
1112	BA-exvivo rh thickness V2	Mean thickness of V2 in the right hemisphere generated by parcellation of the white surface using BA_exvivo parcellatio
1113	BA-exvivo rh thickness MT	Mean thickness of MT in the right hemisphere generated by parcellation of the white surface using BA_exvivo parcellati
1114	BA-exvivo rh thickness perirhinal	Mean thickness of perirhinal in the right hemisphere generated by parcellation of the white surface using BA_exvivo par
1115	BA-exvivo rh thickness entorhinal	Mean thickness of entorhinal in the right hemisphere generated by parcellation of the white surface using BA_exvivo pa
1116	aparc-DKTatlas lh thickness caudalanteriorcingulate	Mean thickness of caudalanteriorcingulate in the left hemisphere generated by parcellation of the white surface using D
1117	aparc-DKTatlas lh thickness caudalmiddlefrontal	Mean thickness of caudalmiddlefrontal in the left hemisphere generated by parcellation of the white surface using DKT p
1118	aparc-DKTatlas lh thickness cuneus	Mean thickness of cuneus in the left hemisphere generated by parcellation of the white surface using DKT parcellation
1119	aparc-DKTatlas lh thickness entorhinal	Mean thickness of entorhinal in the left hemisphere generated by parcellation of the white surface using DKT parcellatio
1120	aparc-DKTatlas lh thickness fusiform	Mean thickness of fusiform in the left hemisphere generated by parcellation of the white surface using DKT parcellation
1121	aparc-DKTatlas lh thickness inferiorparietal	Mean thickness of inferiorparietal in the left hemisphere generated by parcellation of the white surface using DKT parce
1122	aparc-DKTatlas lh thickness inferiortemporal	Mean thickness of inferiortemporal in the left hemisphere generated by parcellation of the white surface using DKT parc
1123	aparc-DKTatlas lh thickness isthmuscingulate	Mean thickness of isthmuscingulate in the left hemisphere generated by parcellation of the white surface using DKT par
1124	aparc-DKTatlas lh thickness lateraloccipital	Mean thickness of lateraloccipital in the left hemisphere generated by parcellation of the white surface using DKT parce
1125	aparc-DKTatlas lh thickness lateralorbitofrontal	Mean thickness of lateralorbitofrontal in the left hemisphere generated by parcellation of the white surface using DKT pa
1126	aparc-DKTatlas lh thickness lingual	Mean thickness of lingual in the left hemisphere generated by parcellation of the white surface using DKT parcellation
1127	aparc-DKTatlas lh thickness medialorbitofrontal	Mean thickness of medialorbitofrontal in the left hemisphere generated by parcellation of the white surface using DKT p
1128	aparc-DKTatlas lh thickness middletemporal	Mean thickness of middletemporal in the left hemisphere generated by parcellation of the white surface using DKT parc
1129	aparc-DKTatlas lh thickness parahippocampal	Mean thickness of parahippocampal in the left hemisphere generated by parcellation of the white surface using DKT pa
1130	aparc-DKTatlas lh thickness paracentral	Mean thickness of paracentral in the left hemisphere generated by parcellation of the white surface using DKT parcellat
1131	aparc-DKTatlas lh thickness parsopercularis	Mean thickness of parsopercularis in the left hemisphere generated by parcellation of the white surface using DKT parc
1132	aparc-DKTatlas lh thickness parsorbitalis	Mean thickness of parsorbitalis in the left hemisphere generated by parcellation of the white surface using DKT parcella
1133	aparc-DKTatlas lh thickness parstriangularis	Mean thickness of parstriangularis in the left hemisphere generated by parcellation of the white surface using DKT parc
1134	aparc-DKTatlas lh thickness pericalcarine	Mean thickness of pericalcarine in the left hemisphere generated by parcellation of the white surface using DKT parcella
1135	aparc-DKTatlas lh thickness postcentral	Mean thickness of postcentral in the left hemisphere generated by parcellation of the white surface using DKT parcellati
1136	aparc-DKTatlas lh thickness posteriorcingulate	Mean thickness of posteriorcingulate in the left hemisphere generated by parcellation of the white surface using DKT pa
1137	aparc-DKTatlas lh thickness precentral	Mean thickness of precentral in the left hemisphere generated by parcellation of the white surface using DKT parcellatio
1138	aparc-DKTatlas lh thickness precuneus	Mean thickness of precuneus in the left hemisphere generated by parcellation of the white surface using DKT parcellatio

IDP number	IDP short name	IDP description
1139	aparc-DKTatlas lh thickness rostralanteriorcingulate	Mean thickness of rostralanteriorcingulate in the left hemisphere generated by parcellation of the white surface using DI
1140	aparc-DKTatlas lh thickness rostralmiddlefrontal	Mean thickness of rostralmiddlefrontal in the left hemisphere generated by parcellation of the white surface using DKT p
1141	aparc-DKTatlas lh thickness superiorfrontal	Mean thickness of superiorfrontal in the left hemisphere generated by parcellation of the white surface using DKT parce
1142	aparc-DKTatlas lh thickness superiorparietal	Mean thickness of superiorparietal in the left hemisphere generated by parcellation of the white surface using DKT parc
1143	aparc-DKTatlas lh thickness superiortemporal	Mean thickness of superiortemporal in the left hemisphere generated by parcellation of the white surface using DKT par
1144	aparc-DKTatlas lh thickness supramarginal	Mean thickness of supramarginal in the left hemisphere generated by parcellation of the white surface using DKT parce
1145	aparc-DKTatlas lh thickness transversetemporal	Mean thickness of transversetemporal in the left hemisphere generated by parcellation of the white surface using DKT p
1146	aparc-DKTatlas lh thickness insula	Mean thickness of insula in the left hemisphere generated by parcellation of the white surface using DKT parcellation
1147	aparc-DKTatlas rh thickness caudalanteriorcingulate	Mean thickness of caudalanteriorcingulate in the right hemisphere generated by parcellation of the white surface using I
1148	aparc-DKTatlas rh thickness caudalmiddlefrontal	Mean thickness of caudalmiddlefrontal in the right hemisphere generated by parcellation of the white surface using DKT
1149	aparc-DKTatlas rh thickness cuneus	Mean thickness of cuneus in the right hemisphere generated by parcellation of the white surface using DKT parcellation
1150	aparc-DKTatlas rh thickness entorhinal	Mean thickness of entorhinal in the right hemisphere generated by parcellation of the white surface using DKT parcellat
1151	aparc-DKTatlas rh thickness fusiform	Mean thickness of fusiform in the right hemisphere generated by parcellation of the white surface using DKT parcellatio
1152	aparc-DKTatlas rh thickness inferiorparietal	Mean thickness of inferiorparietal in the right hemisphere generated by parcellation of the white surface using DKT parc
1153	aparc-DKTatlas rh thickness inferiortemporal	Mean thickness of inferiortemporal in the right hemisphere generated by parcellation of the white surface using DKT par
1154	aparc-DKTatlas rh thickness isthmuscingulate	Mean thickness of isthmuscingulate in the right hemisphere generated by parcellation of the white surface using DKT pa
1155	aparc-DKTatlas rh thickness lateraloccipital	Mean thickness of lateraloccipital in the right hemisphere generated by parcellation of the white surface using DKT parc
1156	aparc-DKTatlas rh thickness lateralorbitofrontal	Mean thickness of lateralorbitofrontal in the right hemisphere generated by parcellation of the white surface using DKT p
1157	aparc-DKTatlas rh thickness lingual	Mean thickness of lingual in the right hemisphere generated by parcellation of the white surface using DKT parcellation
1158	aparc-DKTatlas rh thickness medialorbitofrontal	Mean thickness of medialorbitofrontal in the right hemisphere generated by parcellation of the white surface using DKT
1159	aparc-DKTatlas rh thickness middletemporal	Mean thickness of middletemporal in the right hemisphere generated by parcellation of the white surface using DKT par
1160	aparc-DKTatlas rh thickness parahippocampal	Mean thickness of parahippocampal in the right hemisphere generated by parcellation of the white surface using DKT p
1161	aparc-DKTatlas rh thickness paracentral	Mean thickness of paracentral in the right hemisphere generated by parcellation of the white surface using DKT parcella
1162	aparc-DKTatlas rh thickness parsopercularis	Mean thickness of parsopercularis in the right hemisphere generated by parcellation of the white surface using DKT par
1163	aparc-DKTatlas rh thickness parsorbitalis	Mean thickness of parsorbitalis in the right hemisphere generated by parcellation of the white surface using DKT parcell
1164	aparc-DKTatlas rh thickness parstriangularis	Mean thickness of parstriangularis in the right hemisphere generated by parcellation of the white surface using DKT par
1165	aparc-DKTatlas rh thickness pericalcarine	Mean thickness of pericalcarine in the right hemisphere generated by parcellation of the white surface using DKT parce
1166	aparc-DKTatlas rh thickness postcentral	Mean thickness of postcentral in the right hemisphere generated by parcellation of the white surface using DKT parcella
1167	aparc-DKTatlas rh thickness posteriorcingulate	Mean thickness of posteriorcingulate in the right hemisphere generated by parcellation of the white surface using DKT p
1168	aparc-DKTatlas rh thickness precentral	Mean thickness of precentral in the right hemisphere generated by parcellation of the white surface using DKT parcellat
1169	aparc-DKTatlas rh thickness precuneus	Mean thickness of precuneus in the right hemisphere generated by parcellation of the white surface using DKT parcella
1170	aparc-DKTatlas rh thickness rostralanteriorcingulate	Mean thickness of rostralanteriorcingulate in the right hemisphere generated by parcellation of the white surface using D
1171	aparc-DKTatlas rh thickness rostralmiddlefrontal	Mean thickness of rostralmiddlefrontal in the right hemisphere generated by parcellation of the white surface using DKT
1172	aparc-DKTatlas rh thickness superiorfrontal	Mean thickness of superiorfrontal in the right hemisphere generated by parcellation of the white surface using DKT parc
1173	aparc-DKTatlas rh thickness superiorparietal	Mean thickness of superiorparietal in the right hemisphere generated by parcellation of the white surface using DKT par
1174	aparc-DKTatlas rh thickness superiortemporal	Mean thickness of superiortemporal in the right hemisphere generated by parcellation of the white surface using DKT pa
1175	aparc-DKTatlas rh thickness supramarginal	Mean thickness of supramarginal in the right hemisphere generated by parcellation of the white surface using DKT parc
1176	aparc-DKTatlas rh thickness transversetemporal	Mean thickness of transversetemporal in the right hemisphere generated by parcellation of the white surface using DKT
1177	aparc-DKTatlas rh thickness insula	Mean thickness of insula in the right hemisphere generated by parcellation of the white surface using DKT parcellation
1178	aparc-a2009s lh thickness G+S-frontomargin	Mean thickness of G+S-frontomargin in the left hemisphere generated by parcellation of the white surface using Destrie
1179	aparc-a2009s lh thickness G+S-occipital-inf	Mean thickness of G+S-occipital-inf in the left hemisphere generated by parcellation of the white surface using Destrieu
1180	aparc-a2009s lh thickness G+S-paracentral	Mean thickness of G+S-paracentral in the left hemisphere generated by parcellation of the white surface using Destrieu
1181	aparc-a2009s lh thickness G+S-subcentral	Mean thickness of G+S-subcentral in the left hemisphere generated by parcellation of the white surface using Destrieux
1182	aparc-a2009s lh thickness G+S-transv-frontopol	Mean thickness of G+S-transv-frontopol in the left hemisphere generated by parcellation of the white surface using Des
1183	aparc-a2009s lh thickness G+S-cingul-Ant	Mean thickness of G+S-cingul-Ant in the left hemisphere generated by parcellation of the white surface using Destrieux
1184	aparc-a2009s lh thickness G+S-cingul-Mid-Ant	Mean thickness of G+S-cingul-Mid-Ant in the left hemisphere generated by parcellation of the white surface using Destr
1185	aparc-a2009s lh thickness G+S-cingul-Mid-Post	Mean thickness of G+S-cingul-Mid-Post in the left hemisphere generated by parcellation of the white surface using Des
1186	aparc-a2009s lh thickness G-cingul-Post-dorsal	Mean thickness of G-cingul-Post-dorsal in the left hemisphere generated by parcellation of the white surface using Dest
1187	aparc-a2009s lh thickness G-cingul-Post-ventral	Mean thickness of G-cingul-Post-ventral in the left hemisphere generated by parcellation of the white surface using Des
1188	aparc-a2009s lh thickness G-cuneus	Mean thickness of G-cuneus in the left hemisphere generated by parcellation of the white surface using Destrieux (a200
1189	aparc-a2009s lh thickness G-front-inf-Opercular	Mean thickness of G-front-inf-Opercular in the left hemisphere generated by parcellation of the white surface using Dest
1190	aparc-a2009s lh thickness G-front-inf-Orbital	Mean thickness of G-front-inf-Orbital in the left hemisphere generated by parcellation of the white surface using Destrieu
1191	aparc-a2009s lh thickness G-front-inf-Triangul	Mean thickness of G-front-inf-Triangul in the left hemisphere generated by parcellation of the white surface using Destri
1192	aparc-a2009s lh thickness G-front-middle	Mean thickness of G-front-middle in the left hemisphere generated by parcellation of the white surface using Destrieux (
1193	aparc-a2009s lh thickness G-front-sup	Mean thickness of G-front-sup in the left hemisphere generated by parcellation of the white surface using Destrieux (a2
1194	aparc-a2009s lh thickness G-Ins-lg+S-cent-ins	Mean thickness of G-Ins-lg+S-cent-ins in the left hemisphere generated by parcellation of the white surface using Destri
1195	aparc-a2009s lh thickness G-insular-short	Mean thickness of G-insular-short in the left hemisphere generated by parcellation of the white surface using Destrieux

IDP number	IDP short name	IDP description
1196	aparc-a2009s lh thickness G-occipital-middle	Mean thickness of G-occipital-middle in the left hemisphere generated by parcellation of the white surface using Destrie
1197	aparc-a2009s lh thickness G-occipital-sup	Mean thickness of G-occipital-sup in the left hemisphere generated by parcellation of the white surface using Destrieux
1198	aparc-a2009s lh thickness G-oc-temp-lat-fusifor	Mean thickness of G-oc-temp-lat-fusifor in the left hemisphere generated by parcellation of the white surface using Dest
1199	aparc-a2009s lh thickness G-oc-temp-med-Lingual	Mean thickness of G-oc-temp-med-Lingual in the left hemisphere generated by parcellation of the white surface using D
1200	aparc-a2009s lh thickness G-oc-temp-med-Parahip	Mean thickness of G-oc-temp-med-Parahip in the left hemisphere generated by parcellation of the white surface using D
1201	aparc-a2009s lh thickness G-orbital	Mean thickness of G-orbital in the left hemisphere generated by parcellation of the white surface using Destrieux (a2009
1202	aparc-a2009s lh thickness G-pariet-inf-Angular	Mean thickness of G-pariet-inf-Angular in the left hemisphere generated by parcellation of the white surface using Destr
1203	aparc-a2009s lh thickness G-pariet-inf-Supramar	Mean thickness of G-pariet-inf-Supramar in the left hemisphere generated by parcellation of the white surface using De
1204	aparc-a2009s lh thickness G-parietal-sup	Mean thickness of G-parietal-sup in the left hemisphere generated by parcellation of the white surface using Destrieux (
1205	aparc-a2009s lh thickness G-postcentral	Mean thickness of G-postcentral in the left hemisphere generated by parcellation of the white surface using Destrieux (a
1206	aparc-a2009s lh thickness G-precentral	Mean thickness of G-precentral in the left hemisphere generated by parcellation of the white surface using Destrieux (a2
1207	aparc-a2009s lh thickness G-precuneus	Mean thickness of G-precuneus in the left hemisphere generated by parcellation of the white surface using Destrieux (a
1208	aparc-a2009s lh thickness G-rectus	Mean thickness of G-rectus in the left hemisphere generated by parcellation of the white surface using Destrieux (a2009
1209	aparc-a2009s lh thickness G-subcallosal	Mean thickness of G-subcallosal in the left hemisphere generated by parcellation of the white surface using Destrieux (a
1210	aparc-a2009s lh thickness G-temp-sup-G-T-transv	Mean thickness of G-temp-sup-G-T-transv in the left hemisphere generated by parcellation of the white surface using D
1211	aparc-a2009s lh thickness G-temp-sup-Lateral	Mean thickness of G-temp-sup-Lateral in the left hemisphere generated by parcellation of the white surface using Destr
1212	aparc-a2009s lh thickness G-temp-sup-Plan-polar	Mean thickness of G-temp-sup-Plan-polar in the left hemisphere generated by parcellation of the white surface using De
1213	aparc-a2009s lh thickness G-temp-sup-Plan-tempo	Mean thickness of G-temp-sup-Plan-tempo in the left hemisphere generated by parcellation of the white surface using D
1214	aparc-a2009s lh thickness G-temporal-inf	Mean thickness of G-temporal-inf in the left hemisphere generated by parcellation of the white surface using Destrieux (
1215	aparc-a2009s lh thickness G-temporal-middle	Mean thickness of G-temporal-middle in the left hemisphere generated by parcellation of the white surface using Destrie
1216	aparc-a2009s lh thickness Lat-Fis-ant-Horizont	Mean thickness of Lat-Fis-ant-Horizont in the left hemisphere generated by parcellation of the white surface using Destr
1217	aparc-a2009s lh thickness Lat-Fis-ant-Vertical	Mean thickness of Lat-Fis-ant-Vertical in the left hemisphere generated by parcellation of the white surface using Destri
1218	aparc-a2009s lh thickness Lat-Fis-post	Mean thickness of Lat-Fis-post in the left hemisphere generated by parcellation of the white surface using Destrieux (a2
1219	aparc-a2009s lh thickness Pole-occipital	Mean thickness of Pole-occipital in the left hemisphere generated by parcellation of the white surface using Destrieux (a
1220	aparc-a2009s lh thickness Pole-temporal	Mean thickness of Pole-temporal in the left hemisphere generated by parcellation of the white surface using Destrieux (
1221	aparc-a2009s lh thickness S-calcarine	Mean thickness of S-calcarine in the left hemisphere generated by parcellation of the white surface using Destrieux (a20
1222	aparc-a2009s lh thickness S-central	Mean thickness of S-central in the left hemisphere generated by parcellation of the white surface using Destrieux (a200
1223	aparc-a2009s lh thickness S-cingul-Marginalis	Mean thickness of S-cingul-Marginalis in the left hemisphere generated by parcellation of the white surface using Destri
1224	aparc-a2009s lh thickness S-circular-insula-ant	Mean thickness of S-circular-insula-ant in the left hemisphere generated by parcellation of the white surface using Destr
1225	aparc-a2009s lh thickness S-circular-insula-inf	Mean thickness of S-circular-insula-inf in the left hemisphere generated by parcellation of the white surface using Destri
1226	aparc-a2009s lh thickness S-circular-insula-sup	Mean thickness of S-circular-insula-sup in the left hemisphere generated by parcellation of the white surface using Dest
1227	aparc-a2009s lh thickness S-collat-transv-ant	Mean thickness of S-collat-transv-ant in the left hemisphere generated by parcellation of the white surface using Destrie
1228	aparc-a2009s lh thickness S-collat-transv-post	Mean thickness of S-collat-transv-post in the left hemisphere generated by parcellation of the white surface using Destri
1229	aparc-a2009s lh thickness S-front-inf	Mean thickness of S-front-inf in the left hemisphere generated by parcellation of the white surface using Destrieux (a200
1230	aparc-a2009s lh thickness S-front-middle	Mean thickness of S-front-middle in the left hemisphere generated by parcellation of the white surface using Destrieux (
1231	aparc-a2009s lh thickness S-front-sup	Mean thickness of S-front-sup in the left hemisphere generated by parcellation of the white surface using Destrieux (a20
1232	aparc-a2009s lh thickness S-interm-prim-Jensen	Mean thickness of S-interm-prim-Jensen in the left hemisphere generated by parcellation of the white surface using Des
1233	aparc-a2009s lh thickness S-intrapariet+P-trans	Mean thickness of S-intrapariet+P-trans in the left hemisphere generated by parcellation of the white surface using Des
1234	aparc-a2009s lh thickness S-oc-middle+Lunatus	Mean thickness of S-oc-middle+Lunatus in the left hemisphere generated by parcellation of the white surface using Des
1235	aparc-a2009s lh thickness S-oc-sup+transversal	Mean thickness of S-oc-sup+transversal in the left hemisphere generated by parcellation of the white surface using Des
1236	aparc-a2009s lh thickness S-occipital-ant	Mean thickness of S-occipital-ant in the left hemisphere generated by parcellation of the white surface using Destrieux (
1237	aparc-a2009s lh thickness S-oc-temp-lat	Mean thickness of S-oc-temp-lat in the left hemisphere generated by parcellation of the white surface using Destrieux (a
1238	aparc-a2009s lh thickness S-oc-temp-med+Lingual	Mean thickness of S-oc-temp-med+Lingual in the left hemisphere generated by parcellation of the white surface using D
1239	aparc-a2009s lh thickness S-orbital-lateral	Mean thickness of S-orbital-lateral in the left hemisphere generated by parcellation of the white surface using Destrieux
1240	aparc-a2009s lh thickness S-orbital-med-olfact	Mean thickness of S-orbital-med-olfact in the left hemisphere generated by parcellation of the white surface using Destr
1241	aparc-a2009s lh thickness S-orbital-H-Shaped	Mean thickness of S-orbital-H-Shaped in the left hemisphere generated by parcellation of the white surface using Destri
1242	aparc-a2009s lh thickness S-parieto-occipital	Mean thickness of S-parieto-occipital in the left hemisphere generated by parcellation of the white surface using Destrie
1243	aparc-a2009s lh thickness S-pericallosal	Mean thickness of S-pericallosal in the left hemisphere generated by parcellation of the white surface using Destrieux (a
1244	aparc-a2009s lh thickness S-postcentral	Mean thickness of S-postcentral in the left hemisphere generated by parcellation of the white surface using Destrieux (a
1245	aparc-a2009s lh thickness S-precentral-inf-part	Mean thickness of S-precentral-inf-part in the left hemisphere generated by parcellation of the white surface using Destr
1246	aparc-a2009s lh thickness S-precentral-sup-part	Mean thickness of S-precentral-sup-part in the left hemisphere generated by parcellation of the white surface using Des
1247	aparc-a2009s lh thickness S-suborbital	Mean thickness of S-suborbital in the left hemisphere generated by parcellation of the white surface using Destrieux (a2
1248	aparc-a2009s lh thickness S-subparietal	Mean thickness of S-subparietal in the left hemisphere generated by parcellation of the white surface using Destrieux (a
1249	aparc-a2009s lh thickness S-temporal-inf	Mean thickness of S-temporal-inf in the left hemisphere generated by parcellation of the white surface using Destrieux (
1250	aparc-a2009s lh thickness S-temporal-sup	Mean thickness of S-temporal-sup in the left hemisphere generated by parcellation of the white surface using Destrieux
1251	aparc-a2009s lh thickness S-temporal-transverse	Mean thickness of S-temporal-transverse in the left hemisphere generated by parcellation of the white surface using De
1252	aparc-a2009s rh thickness G+S-frontomargin	Mean thickness of G+S-frontomargin in the right hemisphere generated by parcellation of the white surface using Destri

IDP number	IDP short name	IDP description
1253	aparc-a2009s rh thickness G+S-occipital-inf	Mean thickness of G+S-occipital-inf in the right hemisphere generated by parcellation of the white surface using Destrie
1254	aparc-a2009s rh thickness G+S-paracentral	Mean thickness of G+S-paracentral in the right hemisphere generated by parcellation of the white surface using Destrie
1255	aparc-a2009s rh thickness G+S-subcentral	Mean thickness of G+S-subcentral in the right hemisphere generated by parcellation of the white surface using Destrieu
1256	aparc-a2009s rh thickness G+S-transv-frontopol	Mean thickness of G+S-transv-frontopol in the right hemisphere generated by parcellation of the white surface using De
1257	aparc-a2009s rh thickness G+S-cingul-Ant	Mean thickness of G+S-cingul-Ant in the right hemisphere generated by parcellation of the white surface using Destrieu
1258	aparc-a2009s rh thickness G+S-cingul-Mid-Ant	Mean thickness of G+S-cingul-Mid-Ant in the right hemisphere generated by parcellation of the white surface using Des
1259	aparc-a2009s rh thickness G+S-cingul-Mid-Post	Mean thickness of G+S-cingul-Mid-Post in the right hemisphere generated by parcellation of the white surface using De
1260	aparc-a2009s rh thickness G-cingul-Post-dorsal	Mean thickness of G-cingul-Post-dorsal in the right hemisphere generated by parcellation of the white surface using De
1261	aparc-a2009s rh thickness G-cingul-Post-ventral	Mean thickness of G-cingul-Post-ventral in the right hemisphere generated by parcellation of the white surface using De
1262	aparc-a2009s rh thickness G-cuneus	Mean thickness of G-cuneus in the right hemisphere generated by parcellation of the white surface using Destrieux (a2(
1263	aparc-a2009s rh thickness G-front-inf-Opercular	Mean thickness of G-front-inf-Opercular in the right hemisphere generated by parcellation of the white surface using De
1264	aparc-a2009s rh thickness G-front-inf-Orbital	Mean thickness of G-front-inf-Orbital in the right hemisphere generated by parcellation of the white surface using Destri
1265	aparc-a2009s rh thickness G-front-inf-Triangul	Mean thickness of G-front-inf-Triangul in the right hemisphere generated by parcellation of the white surface using Dest
1266	aparc-a2009s rh thickness G-front-middle	Mean thickness of G-front-middle in the right hemisphere generated by parcellation of the white surface using Destrieux
1267	aparc-a2009s rh thickness G-front-sup	Mean thickness of G-front-sup in the right hemisphere generated by parcellation of the white surface using Destrieux (a
1268	aparc-a2009s rh thickness G-Ins-lg+S-cent-ins	Mean thickness of G-Ins-lg+S-cent-ins in the right hemisphere generated by parcellation of the white surface using Dest
1269	aparc-a2009s rh thickness G-insular-short	Mean thickness of G-insular-short in the right hemisphere generated by parcellation of the white surface using Destrieux
1270	aparc-a2009s rh thickness G-occipital-middle	Mean thickness of G-occipital-middle in the right hemisphere generated by parcellation of the white surface using Destri
1271	aparc-a2009s rh thickness G-occipital-sup	Mean thickness of G-occipital-sup in the right hemisphere generated by parcellation of the white surface using Destrieu:
1272	aparc-a2009s rh thickness G-oc-temp-lat-fusifor	Mean thickness of G-oc-temp-lat-fusifor in the right hemisphere generated by parcellation of the white surface using De
1273	aparc-a2009s rh thickness G-oc-temp-med-Lingual	Mean thickness of G-oc-temp-med-Lingual in the right hemisphere generated by parcellation of the white surface using
1274	aparc-a2009s rh thickness G-oc-temp-med-Parahip	Mean thickness of G-oc-temp-med-Parahip in the right hemisphere generated by parcellation of the white surface using
1275	aparc-a2009s rh thickness G-orbital	Mean thickness of G-orbital in the right hemisphere generated by parcellation of the white surface using Destrieux (a20(
1276	aparc-a2009s rh thickness G-pariet-inf-Angular	Mean thickness of G-pariet-inf-Angular in the right hemisphere generated by parcellation of the white surface using Des
1277	aparc-a2009s rh thickness G-pariet-inf-Supramar	Mean thickness of G-pariet-inf-Supramar in the right hemisphere generated by parcellation of the white surface using D
1278	aparc-a2009s rh thickness G-parietal-sup	Mean thickness of G-parietal-sup in the right hemisphere generated by parcellation of the white surface using Destrieux
1279	aparc-a2009s rh thickness G-postcentral	Mean thickness of G-postcentral in the right hemisphere generated by parcellation of the white surface using Destrieux
1280	aparc-a2009s rh thickness G-precentral	Mean thickness of G-precentral in the right hemisphere generated by parcellation of the white surface using Destrieux (a
1281	aparc-a2009s rh thickness G-precuneus	Mean thickness of G-precuneus in the right hemisphere generated by parcellation of the white surface using Destrieux (
1282	aparc-a2009s rh thickness G-rectus	Mean thickness of G-rectus in the right hemisphere generated by parcellation of the white surface using Destrieux (a20(
1283	aparc-a2009s rh thickness G-subcallosal	Mean thickness of G-subcallosal in the right hemisphere generated by parcellation of the white surface using Destrieux
1284	aparc-a2009s rh thickness G-temp-sup-G-T-transv	Mean thickness of G-temp-sup-G-T-transv in the right hemisphere generated by parcellation of the white surface using I
1285	aparc-a2009s rh thickness G-temp-sup-Lateral	Mean thickness of G-temp-sup-Lateral in the right hemisphere generated by parcellation of the white surface using Dest
1286	aparc-a2009s rh thickness G-temp-sup-Plan-polar	Mean thickness of G-temp-sup-Plan-polar in the right hemisphere generated by parcellation of the white surface using D
1287	aparc-a2009s rh thickness G-temp-sup-Plan-tempo	Mean thickness of G-temp-sup-Plan-tempo in the right hemisphere generated by parcellation of the white surface using
1288	aparc-a2009s rh thickness G-temporal-inf	Mean thickness of G-temporal-inf in the right hemisphere generated by parcellation of the white surface using Destrieux
1289	aparc-a2009s rh thickness G-temporal-middle	Mean thickness of G-temporal-middle in the right hemisphere generated by parcellation of the white surface using Destr
1290	aparc-a2009s rh thickness Lat-Fis-ant-Horizont	Mean thickness of Lat-Fis-ant-Horizont in the right hemisphere generated by parcellation of the white surface using Des
1291	aparc-a2009s rh thickness Lat-Fis-ant-Vertical	Mean thickness of Lat-Fis-ant-Vertical in the right hemisphere generated by parcellation of the white surface using Dest
1292	aparc-a2009s rh thickness Lat-Fis-post	Mean thickness of Lat-Fis-post in the right hemisphere generated by parcellation of the white surface using Destrieux (a
1293	aparc-a2009s rh thickness Pole-occipital	Mean thickness of Pole-occipital in the right hemisphere generated by parcellation of the white surface using Destrieux
1294	aparc-a2009s rh thickness Pole-temporal	Mean thickness of Pole-temporal in the right hemisphere generated by parcellation of the white surface using Destrieux
1295	aparc-a2009s rh thickness S-calcarine	Mean thickness of S-calcarine in the right hemisphere generated by parcellation of the white surface using Destrieux (a:
1296	aparc-a2009s rh thickness S-central	Mean thickness of S-central in the right hemisphere generated by parcellation of the white surface using Destrieux (a20
1297	aparc-a2009s rh thickness S-cingul-Marginalis	Mean thickness of S-cingul-Marginalis in the right hemisphere generated by parcellation of the white surface using Dest
1298	aparc-a2009s rh thickness S-circular-insula-ant	Mean thickness of S-circular-insula-ant in the right hemisphere generated by parcellation of the white surface using Des
1299	aparc-a2009s rh thickness S-circular-insula-inf	Mean thickness of S-circular-insula-inf in the right hemisphere generated by parcellation of the white surface using Dest
1300	aparc-a2009s rh thickness S-circular-insula-sup	Mean thickness of S-circular-insula-sup in the right hemisphere generated by parcellation of the white surface using De
1301	aparc-a2009s rh thickness S-collat-transv-ant	Mean thickness of S-collat-transv-ant in the right hemisphere generated by parcellation of the white surface using Destr
1302	aparc-a2009s rh thickness S-collat-transv-post	Mean thickness of S-collat-transv-post in the right hemisphere generated by parcellation of the white surface using Dest
1303	aparc-a2009s rh thickness S-front-inf	Mean thickness of S-front-inf in the right hemisphere generated by parcellation of the white surface using Destrieux (a2(
1304	aparc-a2009s rh thickness S-front-middle	Mean thickness of S-front-middle in the right hemisphere generated by parcellation of the white surface using Destrieux
1305	aparc-a2009s rh thickness S-front-sup	Mean thickness of S-front-sup in the right hemisphere generated by parcellation of the white surface using Destrieux (a:
1306	aparc-a2009s rh thickness S-interm-prim-Jensen	Mean thickness of S-interm-prim-Jensen in the right hemisphere generated by parcellation of the white surface using De
1307	aparc-a2009s rh thickness S-intrapariet+P-trans	Mean thickness of S-intrapariet+P-trans in the right hemisphere generated by parcellation of the white surface using De
1308	aparc-a2009s rh thickness S-oc-middle+Lunatus	Mean thickness of S-oc-middle+Lunatus in the right hemisphere generated by parcellation of the white surface using De
1309	aparc-a2009s rh thickness S-oc-sup+transversal	Mean thickness of S-oc-sup+transversal in the right hemisphere generated by parcellation of the white surface using De

IDP number	IDP short name	IDP description
1310	aparc-a2009s rh thickness S-occipital-ant	Mean thickness of S-occipital-ant in the right hemisphere generated by parcellation of the white surface using Destrieux
1311	aparc-a2009s rh thickness S-oc-temp-lat	Mean thickness of S-oc-temp-lat in the right hemisphere generated by parcellation of the white surface using Destrieux
1312	aparc-a2009s rh thickness S-oc-temp-med+Lingual	Mean thickness of S-oc-temp-med+Lingual in the right hemisphere generated by parcellation of the white surface using
1313	aparc-a2009s rh thickness S-orbital-lateral	Mean thickness of S-orbital-lateral in the right hemisphere generated by parcellation of the white surface using Destrieu
1314	aparc-a2009s rh thickness S-orbital-med-olfact	Mean thickness of S-orbital-med-olfact in the right hemisphere generated by parcellation of the white surface using Des
1315	aparc-a2009s rh thickness S-orbital-H-Shaped	Mean thickness of S-orbital-H-Shaped in the right hemisphere generated by parcellation of the white surface using Dest
1316	aparc-a2009s rh thickness S-parieto-occipital	Mean thickness of S-parieto-occipital in the right hemisphere generated by parcellation of the white surface using Destri
1317	aparc-a2009s rh thickness S-pericallosal	Mean thickness of S-pericallosal in the right hemisphere generated by parcellation of the white surface using Destrieux
1318	aparc-a2009s rh thickness S-postcentral	Mean thickness of S-postcentral in the right hemisphere generated by parcellation of the white surface using Destrieux (
1319	aparc-a2009s rh thickness S-precentral-inf-part	Mean thickness of S-precentral-inf-part in the right hemisphere generated by parcellation of the white surface using Des
1320	aparc-a2009s rh thickness S-precentral-sup-part	Mean thickness of S-precentral-sup-part in the right hemisphere generated by parcellation of the white surface using De
1321	aparc-a2009s rh thickness S-suborbital	Mean thickness of S-suborbital in the right hemisphere generated by parcellation of the white surface using Destrieux (a
1322	aparc-a2009s rh thickness S-subparietal	Mean thickness of S-subparietal in the right hemisphere generated by parcellation of the white surface using Destrieux (
1323	aparc-a2009s rh thickness S-temporal-inf	Mean thickness of S-temporal-inf in the right hemisphere generated by parcellation of the white surface using Destrieux
1324	aparc-a2009s rh thickness S-temporal-sup	Mean thickness of S-temporal-sup in the right hemisphere generated by parcellation of the white surface using Destrieu
1325	aparc-a2009s rh thickness S-temporal-transverse	Mean thickness of S-temporal-transverse in the right hemisphere generated by parcellation of the white surface using D
1326	aseg global intensity 3rd-Ventricle	Mean intensity of 3rd-Ventricle in the whole brain generated by subcortical volumetric segmentation (aseg)
1327	aseg global intensity 4th-Ventricle	Mean intensity of 4th-Ventricle in the whole brain generated by subcortical volumetric segmentation (aseg)
1328	aseg global intensity 5th-Ventricle	Mean intensity of 5th-Ventricle in the whole brain generated by subcortical volumetric segmentation (aseg)
1329	aseg global intensity Brain-Stem	Mean intensity of Brain-Stem in the whole brain generated by subcortical volumetric segmentation (aseg)
1330	aseg global intensity CSF	Mean intensity of CSF in the whole brain generated by subcortical volumetric segmentation (aseg)
1331	aseg global intensity WM-hypointensities	Mean intensity of WM-hypointensities in the whole brain generated by subcortical volumetric segmentation (aseg)
1332	aseg global intensity non-WM-hypointensities	Mean intensity of non-WM-hypointensities in the whole brain generated by subcortical volumetric segmentation (aseg)
1333	aseg global intensity Optic-Chiasm	Mean intensity of Optic-Chiasm in the whole brain generated by subcortical volumetric segmentation (aseg)
1334	aseg global intensity CC-Posterior	Mean intensity of CC-Posterior in the whole brain generated by subcortical volumetric segmentation (aseg)
1335	aseg global intensity CC-Mid-Posterior	Mean intensity of CC-Mid-Posterior in the whole brain generated by subcortical volumetric segmentation (aseg)
1336	aseg global intensity CC-Central	Mean intensity of CC-Central in the whole brain generated by subcortical volumetric segmentation (aseg)
1337	aseg global intensity CC-Mid-Anterior	Mean intensity of CC-Mid-Anterior in the whole brain generated by subcortical volumetric segmentation (aseg)
1338	aseg global intensity CC-Anterior	Mean intensity of CC-Anterior in the whole brain generated by subcortical volumetric segmentation (aseg)
1339	aseg lh intensity Lateral-Ventricle	Mean intensity of Lateral-Ventricle in the left hemisphere generated by subcortical volumetric segmentation (aseg)
1340	aseg lh intensity Inf-Lat-Vent	Mean intensity of Inf-Lat-Vent in the left hemisphere generated by subcortical volumetric segmentation (aseg)
1341	aseg lh intensity Cerebellum-White-Matter	Mean intensity of Cerebellum-White-Matter in the left hemisphere generated by subcortical volumetric segmentation (as
1342	aseg lh intensity Cerebellum-Cortex	Mean intensity of Cerebellum-Cortex in the left hemisphere generated by subcortical volumetric segmentation (aseg)
1343	aseg lh intensity Thalamus-Proper	Mean intensity of Thalamus-Proper in the left hemisphere generated by subcortical volumetric segmentation (aseg)
1344	aseg lh intensity Caudate	Mean intensity of Caudate in the left hemisphere generated by subcortical volumetric segmentation (aseg)
1345	aseg lh intensity Putamen	Mean intensity of Putamen in the left hemisphere generated by subcortical volumetric segmentation (aseg)
1346	aseg lh intensity Pallidum	Mean intensity of Pallidum in the left hemisphere generated by subcortical volumetric segmentation (aseg)
1347	aseg lh intensity Hippocampus	Mean intensity of Hippocampus in the left hemisphere generated by subcortical volumetric segmentation (aseg)
1348	aseg lh intensity Amygdala	Mean intensity of Amygdala in the left hemisphere generated by subcortical volumetric segmentation (aseg)
1349	aseg lh intensity Accumbens-area	Mean intensity of Accumbens-area in the left hemisphere generated by subcortical volumetric segmentation (aseg)
1350	aseg lh intensity VentralDC	Mean intensity of VentralDC in the left hemisphere generated by subcortical volumetric segmentation (aseg)
1351	aseg lh intensity vessel	Mean intensity of vessel in the left hemisphere generated by subcortical volumetric segmentation (aseg)
1352	aseg lh intensity choroid-plexus	Mean intensity of choroid-plexus in the left hemisphere generated by subcortical volumetric segmentation (aseg)
1353	aseg rh intensity Lateral-Ventricle	Mean intensity of Lateral-Ventricle in the right hemisphere generated by subcortical volumetric segmentation (aseg)
1354	aseg rh intensity Inf-Lat-Vent	Mean intensity of Inf-Lat-Vent in the right hemisphere generated by subcortical volumetric segmentation (aseg)
1355	aseg rh intensity Cerebellum-White-Matter	Mean intensity of Cerebellum-White-Matter in the right hemisphere generated by subcortical volumetric segmentation (a
1356	aseg rh intensity Cerebellum-Cortex	Mean intensity of Cerebellum-Cortex in the right hemisphere generated by subcortical volumetric segmentation (aseg)
1357	aseg rh intensity Thalamus-Proper	Mean intensity of Thalamus-Proper in the right hemisphere generated by subcortical volumetric segmentation (aseg)
1358	aseg rh intensity Caudate	Mean intensity of Caudate in the right hemisphere generated by subcortical volumetric segmentation (aseg)
1359	aseg rh intensity Putamen	Mean intensity of Putamen in the right hemisphere generated by subcortical volumetric segmentation (aseg)
1360	aseg rh intensity Pallidum	Mean intensity of Pallidum in the right hemisphere generated by subcortical volumetric segmentation (aseg)
1361	aseg rh intensity Hippocampus	Mean intensity of Hippocampus in the right hemisphere generated by subcortical volumetric segmentation (aseg)
1362	aseg rh intensity Amygdala	Mean intensity of Amygdala in the right hemisphere generated by subcortical volumetric segmentation (aseg)
1363	aseg rh intensity Accumbens-area	Mean intensity of Accumbens-area in the right hemisphere generated by subcortical volumetric segmentation (aseg)
1364	aseg rh intensity VentralDC	Mean intensity of VentralDC in the right hemisphere generated by subcortical volumetric segmentation (aseg)
1365	aseg rh intensity vessel	Mean intensity of vessel in the right hemisphere generated by subcortical volumetric segmentation (aseg)
1366	aseg rh intensity choroid-plexus	Mean intensity of choroid-plexus in the right hemisphere generated by subcortical volumetric segmentation (aseg)

IDP number	IDP short name	IDP description
1367	wg lh intensity-contrast unknown	Grey-white contrast in unknown in the left hemisphere calculated as a percentage of the mean gray-white matter intensi
1368	wg lh intensity-contrast bankssts	Grey-white contrast in bankssts in the left hemisphere calculated as a percentage of the mean gray-white matter intensi
1369	wg lh intensity-contrast caudalanteriorcingulate	Grey-white contrast in caudalanteriorcingulate in the left hemisphere calculated as a percentage of the mean gray-white
1370	wg lh intensity-contrast caudalmiddlefrontal	Grey-white contrast in caudalmiddlefrontal in the left hemisphere calculated as a percentage of the mean gray-white ma
1371	wg lh intensity-contrast cuneus	Grey-white contrast in cuneus in the left hemisphere calculated as a percentage of the mean gray-white matter intensity
1372	wg lh intensity-contrast entorhinal	Grey-white contrast in entorhinal in the left hemisphere calculated as a percentage of the mean gray-white matter intens
1373	wg lh intensity-contrast fusiform	Grey-white contrast in fusiform in the left hemisphere calculated as a percentage of the mean gray-white matter intensit
1374	wg lh intensity-contrast inferiorparietal	Grey-white contrast in inferiorparietal in the left hemisphere calculated as a percentage of the mean gray-white matter ir
1375	wg lh intensity-contrast inferiortemporal	Grey-white contrast in inferiortemporal in the left hemisphere calculated as a percentage of the mean gray-white matter
1376	wg lh intensity-contrast isthmuscingulate	Grey-white contrast in isthmuscingulate in the left hemisphere calculated as a percentage of the mean gray-white matte
1377	wg lh intensity-contrast lateraloccipital	Grey-white contrast in lateraloccipital in the left hemisphere calculated as a percentage of the mean gray-white matter ir
1378	wg lh intensity-contrast lateralorbitofrontal	Grey-white contrast in lateralorbitofrontal in the left hemisphere calculated as a percentage of the mean gray-white matt
1379	wg lh intensity-contrast lingual	Grey-white contrast in lingual in the left hemisphere calculated as a percentage of the mean gray-white matter intensity
1380	wg lh intensity-contrast medialorbitofrontal	Grey-white contrast in medialorbitofrontal in the left hemisphere calculated as a percentage of the mean gray-white mat
1381	wg lh intensity-contrast middletemporal	Grey-white contrast in middletemporal in the left hemisphere calculated as a percentage of the mean gray-white matter
1382	wg lh intensity-contrast parahippocampal	Grey-white contrast in parahippocampal in the left hemisphere calculated as a percentage of the mean gray-white matte
1383	wg lh intensity-contrast paracentral	Grey-white contrast in paracentral in the left hemisphere calculated as a percentage of the mean gray-white matter inter
1384	wg lh intensity-contrast parsopercularis	Grey-white contrast in parsopercularis in the left hemisphere calculated as a percentage of the mean gray-white matter
1385	wg lh intensity-contrast parsorbitalis	Grey-white contrast in parsorbitalis in the left hemisphere calculated as a percentage of the mean gray-white matter inte
1386	wg lh intensity-contrast parstriangularis	Grey-white contrast in parstriangularis in the left hemisphere calculated as a percentage of the mean gray-white matter
1387	wg lh intensity-contrast pericalcarine	Grey-white contrast in pericalcarine in the left hemisphere calculated as a percentage of the mean gray-white matter int
1388	wg lh intensity-contrast postcentral	Grey-white contrast in postcentral in the left hemisphere calculated as a percentage of the mean gray-white matter inter
1389	wg lh intensity-contrast posteriorcingulate	Grey-white contrast in posteriorcingulate in the left hemisphere calculated as a percentage of the mean gray-white matt
1390	wg lh intensity-contrast precentral	Grey-white contrast in precentral in the left hemisphere calculated as a percentage of the mean gray-white matter intens
1391	wg lh intensity-contrast precuneus	Grey-white contrast in precuneus in the left hemisphere calculated as a percentage of the mean gray-white matter inten
1392	wg lh intensity-contrast rostralanteriorcingulate	Grey-white contrast in rostralanteriorcingulate in the left hemisphere calculated as a percentage of the mean gray-white
1393	wg lh intensity-contrast rostralmiddlefrontal	Grey-white contrast in rostralmiddlefrontal in the left hemisphere calculated as a percentage of the mean gray-white ma
1394	wg lh intensity-contrast superiorfrontal	Grey-white contrast in superiorfrontal in the left hemisphere calculated as a percentage of the mean gray-white matter ir
1395	wg lh intensity-contrast superiorparietal	Grey-white contrast in superiorparietal in the left hemisphere calculated as a percentage of the mean gray-white matter
1396	wg lh intensity-contrast superiortemporal	Grey-white contrast in superiortemporal in the left hemisphere calculated as a percentage of the mean gray-white matte
1397	wg lh intensity-contrast supramarginal	Grey-white contrast in supramarginal in the left hemisphere calculated as a percentage of the mean gray-white matter ir
1398	wg lh intensity-contrast frontalpole	Grey-white contrast in frontalpole in the left hemisphere calculated as a percentage of the mean gray-white matter inten
1399	wg lh intensity-contrast temporalpole	Grey-white contrast in temporalpole in the left hemisphere calculated as a percentage of the mean gray-white matter int
1400	wg lh intensity-contrast transversetemporal	Grey-white contrast in transversetemporal in the left hemisphere calculated as a percentage of the mean gray-white ma
1401	wg lh intensity-contrast insula	Grey-white contrast in insula in the left hemisphere calculated as a percentage of the mean gray-white matter intensity f
1402	wg rh intensity-contrast unknown	Grey-white contrast in unknown in the right hemisphere calculated as a percentage of the mean gray-white matter inten
1403	wg rh intensity-contrast bankssts	Grey-white contrast in bankssts in the right hemisphere calculated as a percentage of the mean gray-white matter inten
1404	wg rh intensity-contrast caudalanteriorcingulate	Grey-white contrast in caudalanteriorcingulate in the right hemisphere calculated as a percentage of the mean gray-whi
1405	wg rh intensity-contrast caudalmiddlefrontal	Grey-white contrast in caudalmiddlefrontal in the right hemisphere calculated as a percentage of the mean gray-white m
1406	wg rh intensity-contrast cuneus	Grey-white contrast in cuneus in the right hemisphere calculated as a percentage of the mean gray-white matter intensi
1407	wg rh intensity-contrast entorhinal	Grey-white contrast in entorhinal in the right hemisphere calculated as a percentage of the mean gray-white matter inter
1408	wg rh intensity-contrast fusiform	Grey-white contrast in fusiform in the right hemisphere calculated as a percentage of the mean gray-white matter intens
1409	wg rh intensity-contrast inferiorparietal	Grey-white contrast in inferiorparietal in the right hemisphere calculated as a percentage of the mean gray-white matter
1410	wg rh intensity-contrast inferiortemporal	Grey-white contrast in inferiortemporal in the right hemisphere calculated as a percentage of the mean gray-white matte
1411	wg rh intensity-contrast isthmuscingulate	Grey-white contrast in isthmuscingulate in the right hemisphere calculated as a percentage of the mean gray-white matt
1412	wg rh intensity-contrast lateraloccipital	Grey-white contrast in lateraloccipital in the right hemisphere calculated as a percentage of the mean gray-white matter
1413	wg rh intensity-contrast lateralorbitofrontal	Grey-white contrast in lateralorbitofrontal in the right hemisphere calculated as a percentage of the mean gray-white ma
1414	wg rh intensity-contrast lingual	Grey-white contrast in lingual in the right hemisphere calculated as a percentage of the mean gray-white matter intensity
1415	wg rh intensity-contrast medialorbitofrontal	Grey-white contrast in medialorbitofrontal in the right hemisphere calculated as a percentage of the mean gray-white ma
1416	wg rh intensity-contrast middletemporal	Grey-white contrast in middletemporal in the right hemisphere calculated as a percentage of the mean gray-white matte
1417	wg rh intensity-contrast parahippocampal	Grey-white contrast in parahippocampal in the right hemisphere calculated as a percentage of the mean gray-white mat
1418	wg rh intensity-contrast paracentral	Grey-white contrast in paracentral in the right hemisphere calculated as a percentage of the mean gray-white matter int
1419	wg rh intensity-contrast parsopercularis	Grey-white contrast in parsopercularis in the right hemisphere calculated as a percentage of the mean gray-white matte
1420	wg rh intensity-contrast parsorbitalis	Grey-white contrast in parsorbitalis in the right hemisphere calculated as a percentage of the mean gray-white matter in
1421	wg rh intensity-contrast parstriangularis	Grey-white contrast in parstriangularis in the right hemisphere calculated as a percentage of the mean gray-white matte
1422	wg rh intensity-contrast pericalcarine	Grey-white contrast in pericalcarine in the right hemisphere calculated as a percentage of the mean gray-white matter ir
1423	wg rh intensity-contrast postcentral	Grey-white contrast in postcentral in the right hemisphere calculated as a percentage of the mean gray-white matter inte

IDP number	IDP short name	IDP description
1424	wg rh intensity-contrast posteriorcingulate	Grey-white contrast in posteriorcingulate in the right hemisphere calculated as a percentage of the mean gray-white ma
1425	wg rh intensity-contrast precentral	Grey-white contrast in precentral in the right hemisphere calculated as a percentage of the mean gray-white matter inte
1426	wg rh intensity-contrast precuneus	Grey-white contrast in precuneus in the right hemisphere calculated as a percentage of the mean gray-white matter inte
1427	wg rh intensity-contrast rostralanteriorcingulate	Grey-white contrast in rostralanteriorcingulate in the right hemisphere calculated as a percentage of the mean gray-whit
1428	wg rh intensity-contrast rostralmiddlefrontal	Grey-white contrast in rostralmiddlefrontal in the right hemisphere calculated as a percentage of the mean gray-white m
1429	wg rh intensity-contrast superiorfrontal	Grey-white contrast in superiorfrontal in the right hemisphere calculated as a percentage of the mean gray-white matter
1430	wg rh intensity-contrast superiorparietal	Grey-white contrast in superiorparietal in the right hemisphere calculated as a percentage of the mean gray-white matte
1431	wg rh intensity-contrast superiortemporal	Grey-white contrast in superiortemporal in the right hemisphere calculated as a percentage of the mean gray-white mat
1432	wg rh intensity-contrast supramarginal	Grey-white contrast in supramarginal in the right hemisphere calculated as a percentage of the mean gray-white matter
1433	wg rh intensity-contrast frontalpole	Grey-white contrast in frontalpole in the right hemisphere calculated as a percentage of the mean gray-white matter inte
1434	wg rh intensity-contrast temporalpole	Grey-white contrast in temporalpole in the right hemisphere calculated as a percentage of the mean gray-white matter i
1435	wg rh intensity-contrast transversetemporal	Grey-white contrast in transversetemporal in the right hemisphere calculated as a percentage of the mean gray-white m
1436	wg rh intensity-contrast insula	Grey-white contrast in insula in the right hemisphere calculated as a percentage of the mean gray-white matter intensity
1437	IDP T2 FLAIR BIANCA WMH volume	Total volume of white matter hyperintensities (from T1 and T2_FLAIR images)
1438	IDP SWI T2star left thalamus	Median T2star in left thalamus (from SWI data)
1439	IDP SWI T2star right thalamus	Median T2star in right thalamus (from SWI data)
1440	IDP SWI T2star left caudate	Median T2star in left caudate (from SWI data)
1441	IDP SWI T2star right caudate	Median T2star in right caudate (from SWI data)
1442	IDP SWI T2star left putamen	Median T2star in left putamen (from SWI data)
1443	IDP SWI T2star right putamen	Median T2star in right putamen (from SWI data)
1444	IDP SWI T2star left pallidum	Median T2star in left pallidum (from SWI data)
1445	IDP SWI T2star right pallidum	Median T2star in right pallidum (from SWI data)
1446	IDP SWI T2star left hippocampus	Median T2star in left hippocampus (from SWI data)
1447	IDP SWI T2star right hippocampus	Median T2star in right hippocampus (from SWI data)
1448	IDP SWI T2star left amygdala	Median T2star in left amygdala (from SWI data)
1449	IDP SWI T2star right amygdala	Median T2star in right amygdala (from SWI data)
1450	IDP SWI T2star left accumbens	Median T2star in left accumbens (from SWI data)
1451	IDP SWI T2star right accumbens	Median T2star in right accumbens (from SWI data)
1452	IDP dMRI TBSS FA Middle cerebellar peduncle	Mean FA (fractional anisotropy) in middle cerebellar peduncle on FA skeleton (from dMRI data)
1453	IDP dMRI TBSS FA Pontine crossing tract	Mean FA (fractional anisotropy) in pontine crossing tract on FA skeleton (from dMRI data)
1454	IDP dMRI TBSS FA Genu of corpus callosum	Mean FA (fractional anisotropy) in genu of corpus callosum on FA skeleton (from dMRI data)
1455	IDP dMRI TBSS FA Body of corpus callosum	Mean FA (fractional anisotropy) in body of corpus callosum on FA skeleton (from dMRI data)
1456	IDP dMRI TBSS FA Splenium of corpus callosum	Mean FA (fractional anisotropy) in splenium of corpus callosum on FA skeleton (from dMRI data)
1457	IDP dMRI TBSS FA Fornix	Mean FA (fractional anisotropy) in fornix on FA skeleton (from dMRI data)
1458	IDP dMRI TBSS FA Corticospinal tract R	Mean FA (fractional anisotropy) in corticospinal tract (right) on FA skeleton (from dMRI data)
1459	IDP dMRI TBSS FA Corticospinal tract L	Mean FA (fractional anisotropy) in corticospinal tract (left) on FA skeleton (from dMRI data)
1460	IDP dMRI TBSS FA Medial lemniscus R	Mean FA (fractional anisotropy) in medial lemniscus (right) on FA skeleton (from dMRI data)
1461	IDP dMRI TBSS FA Medial lemniscus L	Mean FA (fractional anisotropy) in medial lemniscus (left) on FA skeleton (from dMRI data)
1462	IDP dMRI TBSS FA Inferior cerebellar peduncle R	Mean FA (fractional anisotropy) in inferior cerebellar peduncle (right) on FA skeleton (from dMRI data)
1463	IDP dMRI TBSS FA Inferior cerebellar peduncle L	Mean FA (fractional anisotropy) in inferior cerebellar peduncle (left) on FA skeleton (from dMRI data)
1464	IDP dMRI TBSS FA Superior cerebellar peduncle R	Mean FA (fractional anisotropy) in superior cerebellar peduncle (right) on FA skeleton (from dMRI data)
1465	IDP dMRI TBSS FA Superior cerebellar peduncle L	Mean FA (fractional anisotropy) in superior cerebellar peduncle (left) on FA skeleton (from dMRI data)
1466	IDP dMRI TBSS FA Cerebral peduncle R	Mean FA (fractional anisotropy) in cerebral peduncle (right) on FA skeleton (from dMRI data)
1467	IDP dMRI TBSS FA Cerebral peduncle L	Mean FA (fractional anisotropy) in cerebral peduncle (left) on FA skeleton (from dMRI data)
1468	IDP dMRI TBSS FA Anterior limb of internal capsule R	Mean FA (fractional anisotropy) in anterior limb of internal capsule (right) on FA skeleton (from dMRI data)
1469	IDP dMRI TBSS FA Anterior limb of internal capsule L	Mean FA (fractional anisotropy) in anterior limb of internal capsule (left) on FA skeleton (from dMRI data)
1470	IDP dMRI TBSS FA Posterior limb of internal capsule R	Mean FA (fractional anisotropy) in posterior limb of internal capsule (right) on FA skeleton (from dMRI data)
1471	IDP dMRI TBSS FA Posterior limb of internal capsule L	Mean FA (fractional anisotropy) in posterior limb of internal capsule (left) on FA skeleton (from dMRI data)
1472	IDP dMRI TBSS FA Retrolenticular part of internal capsule R	Mean FA (fractional anisotropy) in retrolenticular part of internal capsule (right) on FA skeleton (from dMRI data)
1473	IDP dMRI TBSS FA Retrolenticular part of internal capsule L	Mean FA (fractional anisotropy) in retrolenticular part of internal capsule (left) on FA skeleton (from dMRI data)
1474	IDP dMRI TBSS FA Anterior corona radiata R	Mean FA (fractional anisotropy) in anterior corona radiata (right) on FA skeleton (from dMRI data)
1475	IDP dMRI TBSS FA Anterior corona radiata L	Mean FA (fractional anisotropy) in anterior corona radiata (left) on FA skeleton (from dMRI data)
1476	IDP dMRI TBSS FA Superior corona radiata R	Mean FA (fractional anisotropy) in superior corona radiata (right) on FA skeleton (from dMRI data)
1477	IDP dMRI TBSS FA Superior corona radiata L	Mean FA (fractional anisotropy) in superior corona radiata (left) on FA skeleton (from dMRI data)
1478	IDP dMRI TBSS FA Posterior corona radiata R	Mean FA (fractional anisotropy) in posterior corona radiata (right) on FA skeleton (from dMRI data)
1479	IDP dMRI TBSS FA Posterior corona radiata L	Mean FA (fractional anisotropy) in posterior corona radiata (left) on FA skeleton (from dMRI data)
1480	IDP dMRI TBSS FA Posterior thalamic radiation R	Mean FA (fractional anisotropy) in posterior thalamic radiation (right) on FA skeleton (from dMRI data)

IDP number	IDP short name	IDP description
1481	IDP dMRI TBSS FA Posterior thalamic radiation L	Mean FA (fractional anisotropy) in posterior thalamic radiation (left) on FA skeleton (from dMRI data)
1482	IDP dMRI TBSS FA Sagittal stratum R	Mean FA (fractional anisotropy) in sagittal stratum (right) on FA skeleton (from dMRI data)
1483	IDP dMRI TBSS FA Sagittal stratum L	Mean FA (fractional anisotropy) in sagittal stratum (left) on FA skeleton (from dMRI data)
1484	IDP dMRI TBSS FA External capsule R	Mean FA (fractional anisotropy) in external capsule (right) on FA skeleton (from dMRI data)
1485	IDP dMRI TBSS FA External capsule L	Mean FA (fractional anisotropy) in external capsule (left) on FA skeleton (from dMRI data)
1486	IDP dMRI TBSS FA Cingulum cingulate gyrus R	Mean FA (fractional anisotropy) in cingulum cingulate gyrus (right) on FA skeleton (from dMRI data)
1487	IDP dMRI TBSS FA Cingulum cingulate gyrus L	Mean FA (fractional anisotropy) in cingulum cingulate gyrus (left) on FA skeleton (from dMRI data)
1488	IDP dMRI TBSS FA Cingulum hippocampus R	Mean FA (fractional anisotropy) in cingulum hippocampus (right) on FA skeleton (from dMRI data)
1489	IDP dMRI TBSS FA Cingulum hippocampus L	Mean FA (fractional anisotropy) in cingulum hippocampus (left) on FA skeleton (from dMRI data)
1490	IDP dMRI TBSS FA Fornix cres+Stria terminalis R	Mean FA (fractional anisotropy) in fornix cres+stria terminalis (right) on FA skeleton (from dMRI data)
1491	IDP dMRI TBSS FA Fornix cres+Stria terminalis L	Mean FA (fractional anisotropy) in fornix cres+stria terminalis (left) on FA skeleton (from dMRI data)
1492	IDP dMRI TBSS FA Superior longitudinal fasciculus R	Mean FA (fractional anisotropy) in superior longitudinal fasciculus (right) on FA skeleton (from dMRI data)
1493	IDP dMRI TBSS FA Superior longitudinal fasciculus L	Mean FA (fractional anisotropy) in superior longitudinal fasciculus (left) on FA skeleton (from dMRI data)
1494	IDP dMRI TBSS FA Superior fronto-occipital fasciculus R	Mean FA (fractional anisotropy) in superior fronto-occipital fasciculus (right) on FA skeleton (from dMRI data)
1495	IDP dMRI TBSS FA Superior fronto-occipital fasciculus L	Mean FA (fractional anisotropy) in superior fronto-occipital fasciculus (left) on FA skeleton (from dMRI data)
1496	IDP dMRI TBSS FA Uncinate fasciculus R	Mean FA (fractional anisotropy) in uncinate fasciculus (right) on FA skeleton (from dMRI data)
1497	IDP dMRI TBSS FA Uncinate fasciculus L	Mean FA (fractional anisotropy) in uncinate fasciculus (left) on FA skeleton (from dMRI data)
1498	IDP dMRI TBSS FA Tapetum R	Mean FA (fractional anisotropy) in tapetum (right) on FA skeleton (from dMRI data)
1499	IDP dMRI TBSS FA Tapetum L	Mean FA (fractional anisotropy) in tapetum (left) on FA skeleton (from dMRI data)
1500	IDP dMRI ProbtrackX FA ar l	Weighted-mean FA (fractional anisotropy) in tract left acoustic radiation (from dMRI data)
1501	IDP dMRI ProbtrackX FA ar r	Weighted-mean FA (fractional anisotropy) in tract right acoustic radiation (from dMRI data)
1502	IDP dMRI ProbtrackX FA atr l	Weighted-mean FA (fractional anisotropy) in tract left anterior thalamic radiation (from dMRI data)
1503	IDP dMRI ProbtrackX FA atr r	Weighted-mean FA (fractional anisotropy) in tract right anterior thalamic radiation (from dMRI data)
1504	IDP dMRI ProbtrackX FA cgc l	Weighted-mean FA (fractional anisotropy) in tract left cingulate gyrus part of cingulum (from dMRI data)
1505	IDP dMRI ProbtrackX FA cgc r	Weighted-mean FA (fractional anisotropy) in tract right cingulate gyrus part of cingulum (from dMRI data)
1506	IDP dMRI ProbtrackX FA cgh l	Weighted-mean FA (fractional anisotropy) in tract left parahippocampal part of cingulum (from dMRI data)
1507	IDP dMRI ProbtrackX FA cgh r	Weighted-mean FA (fractional anisotropy) in tract right parahippocampal part of cingulum (from dMRI data)
1508	IDP dMRI ProbtrackX FA cst l	Weighted-mean FA (fractional anisotropy) in tract left corticospinal tract (from dMRI data)
1509	IDP dMRI ProbtrackX FA cst r	Weighted-mean FA (fractional anisotropy) in tract right corticospinal tract (from dMRI data)
1510	IDP dMRI ProbtrackX FA fma	Weighted-mean FA (fractional anisotropy) in tract forceps major (from dMRI data)
1511	IDP dMRI ProbtrackX FA fmi	Weighted-mean FA (fractional anisotropy) in tract forceps minor (from dMRI data)
1512	IDP dMRI ProbtrackX FA ifo l	Weighted-mean FA (fractional anisotropy) in tract left inferior fronto-occipital fasciculus (from dMRI data)
1513	IDP dMRI ProbtrackX FA ifo r	Weighted-mean FA (fractional anisotropy) in tract right inferior fronto-occipital fasciculus (from dMRI data)
1514	IDP dMRI ProbtrackX FA ilf l	Weighted-mean FA (fractional anisotropy) in tract left inferior longitudinal fasciculus (from dMRI data)
1515	IDP dMRI ProbtrackX FA ilf r	Weighted-mean FA (fractional anisotropy) in tract right inferior longitudinal fasciculus (from dMRI data)
1516	IDP dMRI ProbtrackX FA mcp	Weighted-mean FA (fractional anisotropy) in tract middle cerebellar peduncle (from dMRI data)
1517	IDP dMRI ProbtrackX FA ml l	Weighted-mean FA (fractional anisotropy) in tract left medial lemniscus (from dMRI data)
1518	IDP dMRI ProbtrackX FA ml r	Weighted-mean FA (fractional anisotropy) in tract right medial lemniscus (from dMRI data)
1519	IDP dMRI ProbtrackX FA ptr l	Weighted-mean FA (fractional anisotropy) in tract left posterior thalamic radiation (from dMRI data)
1520	IDP dMRI ProbtrackX FA ptr r	Weighted-mean FA (fractional anisotropy) in tract right posterior thalamic radiation (from dMRI data)
1521	IDP dMRI ProbtrackX FA slf l	Weighted-mean FA (fractional anisotropy) in tract left superior longitudinal fasciculus (from dMRI data)
1522	IDP dMRI ProbtrackX FA slf r	Weighted-mean FA (fractional anisotropy) in tract right superior longitudinal fasciculus (from dMRI data)
1523	IDP dMRI ProbtrackX FA str l	Weighted-mean FA (fractional anisotropy) in tract left superior thalamic radiation (from dMRI data)
1524	IDP dMRI ProbtrackX FA str r	Weighted-mean FA (fractional anisotropy) in tract right superior thalamic radiation (from dMRI data)
1525	IDP dMRI ProbtrackX FA unc l	Weighted-mean FA (fractional anisotropy) in tract left uncinate fasciculus (from dMRI data)
1526	IDP dMRI ProbtrackX FA unc r	Weighted-mean FA (fractional anisotropy) in tract right uncinate fasciculus (from dMRI data)
1527	IDP dMRI TBSS MO Middle cerebellar peduncle	Mean MO (diffusion tensor mode) in middle cerebellar peduncle on FA (fractional anisotropy) skeleton (from dMRI data)
1528	IDP dMRI TBSS MO Pontine crossing tract	Mean MO (diffusion tensor mode) in pontine crossing tract on FA (fractional anisotropy) skeleton (from dMRI data)
1529	IDP dMRI TBSS MO Genu of corpus callosum	Mean MO (diffusion tensor mode) in genu of corpus callosum on FA (fractional anisotropy) skeleton (from dMRI data)
1530	IDP dMRI TBSS MO Body of corpus callosum	Mean MO (diffusion tensor mode) in body of corpus callosum on FA (fractional anisotropy) skeleton (from dMRI data)
1531	IDP dMRI TBSS MO Splenium of corpus callosum	Mean MO (diffusion tensor mode) in splenium of corpus callosum on FA (fractional anisotropy) skeleton (from dMRI dat
1532	IDP dMRI TBSS MO Fornix	Mean MO (diffusion tensor mode) in fornix on FA (fractional anisotropy) skeleton (from dMRI data)
1533	IDP dMRI TBSS MO Corticospinal tract R	Mean MO (diffusion tensor mode) in corticospinal tract (right) on FA (fractional anisotropy) skeleton (from dMRI data)
1534	IDP dMRI TBSS MO Corticospinal tract L	Mean MO (diffusion tensor mode) in corticospinal tract (left) on FA (fractional anisotropy) skeleton (from dMRI data)
1535	IDP dMRI TBSS MO Medial lemniscus R	Mean MO (diffusion tensor mode) in medial lemniscus (right) on FA (fractional anisotropy) skeleton (from dMRI data)
1536	IDP dMRI TBSS MO Medial lemniscus L	Mean MO (diffusion tensor mode) in medial lemniscus (left) on FA (fractional anisotropy) skeleton (from dMRI data)
1537	IDP dMRI TBSS MO Inferior cerebellar peduncle R	Mean MO (diffusion tensor mode) in inferior cerebellar peduncle (right) on FA (fractional anisotropy) skeleton (from dMF

IDP number	IDP short name	IDP description
1538	IDP dMRI TBSS MO Inferior cerebellar peduncle L	Mean MO (diffusion tensor mode) in inferior cerebellar peduncle (left) on FA (fractional anisotropy) skeleton (from dMRI
1539	IDP dMRI TBSS MO Superior cerebellar peduncle R	Mean MO (diffusion tensor mode) in superior cerebellar peduncle (right) on FA (fractional anisotropy) skeleton (from dM
1540	IDP dMRI TBSS MO Superior cerebellar peduncle L	Mean MO (diffusion tensor mode) in superior cerebellar peduncle (left) on FA (fractional anisotropy) skeleton (from dMR
1541	IDP dMRI TBSS MO Cerebral peduncle R	Mean MO (diffusion tensor mode) in cerebral peduncle (right) on FA (fractional anisotropy) skeleton (from dMRI data)
1542	IDP dMRI TBSS MO Cerebral peduncle L	Mean MO (diffusion tensor mode) in cerebral peduncle (left) on FA (fractional anisotropy) skeleton (from dMRI data)
1543	IDP dMRI TBSS MO Anterior limb of internal capsule R	Mean MO (diffusion tensor mode) in anterior limb of internal capsule (right) on FA (fractional anisotropy) skeleton (from
1544	IDP dMRI TBSS MO Anterior limb of internal capsule L	Mean MO (diffusion tensor mode) in anterior limb of internal capsule (left) on FA (fractional anisotropy) skeleton (from d
1545	IDP dMRI TBSS MO Posterior limb of internal capsule R	Mean MO (diffusion tensor mode) in posterior limb of internal capsule (right) on FA (fractional anisotropy) skeleton (from
1546	IDP dMRI TBSS MO Posterior limb of internal capsule L	Mean MO (diffusion tensor mode) in posterior limb of internal capsule (left) on FA (fractional anisotropy) skeleton (from
1547	IDP dMRI TBSS MO Retrolenticular part of internal capsule R	Mean MO (diffusion tensor mode) in retrolenticular part of internal capsule (right) on FA (fractional anisotropy) skeleton
1548	IDP dMRI TBSS MO Retrolenticular part of internal capsule L	Mean MO (diffusion tensor mode) in retrolenticular part of internal capsule (left) on FA (fractional anisotropy) skeleton (f
1549	IDP dMRI TBSS MO Anterior corona radiata R	Mean MO (diffusion tensor mode) in anterior corona radiata (right) on FA (fractional anisotropy) skeleton (from dMRI da
1550	IDP dMRI TBSS MO Anterior corona radiata L	Mean MO (diffusion tensor mode) in anterior corona radiata (left) on FA (fractional anisotropy) skeleton (from dMRI data
1551	IDP dMRI TBSS MO Superior corona radiata R	Mean MO (diffusion tensor mode) in superior corona radiata (right) on FA (fractional anisotropy) skeleton (from dMRI da
1552	IDP dMRI TBSS MO Superior corona radiata L	Mean MO (diffusion tensor mode) in superior corona radiata (left) on FA (fractional anisotropy) skeleton (from dMRI data
1553	IDP dMRI TBSS MO Posterior corona radiata R	Mean MO (diffusion tensor mode) in posterior corona radiata (right) on FA (fractional anisotropy) skeleton (from dMRI d
1554	IDP dMRI TBSS MO Posterior corona radiata L	Mean MO (diffusion tensor mode) in posterior corona radiata (left) on FA (fractional anisotropy) skeleton (from dMRI dat
1555	IDP dMRI TBSS MO Posterior thalamic radiation R	Mean MO (diffusion tensor mode) in posterior thalamic radiation (right) on FA (fractional anisotropy) skeleton (from dMR
1556	IDP dMRI TBSS MO Posterior thalamic radiation L	Mean MO (diffusion tensor mode) in posterior thalamic radiation (left) on FA (fractional anisotropy) skeleton (from dMRI
1557	IDP dMRI TBSS MO Sagittal stratum R	Mean MO (diffusion tensor mode) in sagittal stratum (right) on FA (fractional anisotropy) skeleton (from dMRI data)
1558	IDP dMRI TBSS MO Sagittal stratum L	Mean MO (diffusion tensor mode) in sagittal stratum (left) on FA (fractional anisotropy) skeleton (from dMRI data)
1559	IDP dMRI TBSS MO External capsule R	Mean MO (diffusion tensor mode) in external capsule (right) on FA (fractional anisotropy) skeleton (from dMRI data)
1560	IDP dMRI TBSS MO External capsule L	Mean MO (diffusion tensor mode) in external capsule (left) on FA (fractional anisotropy) skeleton (from dMRI data)
1561	IDP dMRI TBSS MO Cingulum cingulate gyrus R	Mean MO (diffusion tensor mode) in cingulum cingulate gyrus (right) on FA (fractional anisotropy) skeleton (from dMRI d
1562	IDP dMRI TBSS MO Cingulum cingulate gyrus L	Mean MO (diffusion tensor mode) in cingulum cingulate gyrus (left) on FA (fractional anisotropy) skeleton (from dMRI da
1563	IDP dMRI TBSS MO Cingulum hippocampus R	Mean MO (diffusion tensor mode) in cingulum hippocampus (right) on FA (fractional anisotropy) skeleton (from dMRI da
1564	IDP dMRI TBSS MO Cingulum hippocampus L	Mean MO (diffusion tensor mode) in cingulum hippocampus (left) on FA (fractional anisotropy) skeleton (from dMRI data
1565	IDP dMRI TBSS MO Fornix cres+Stria terminalis R	Mean MO (diffusion tensor mode) in fornix cres+stria terminalis (right) on FA (fractional anisotropy) skeleton (from dMRI
1566	IDP dMRI TBSS MO Fornix cres+Stria terminalis L	Mean MO (diffusion tensor mode) in fornix cres+stria terminalis (left) on FA (fractional anisotropy) skeleton (from dMRI d
1567	IDP dMRI TBSS MO Superior longitudinal fasciculus R	Mean MO (diffusion tensor mode) in superior longitudinal fasciculus (right) on FA (fractional anisotropy) skeleton (from d
1568	IDP dMRI TBSS MO Superior longitudinal fasciculus L	Mean MO (diffusion tensor mode) in superior longitudinal fasciculus (left) on FA (fractional anisotropy) skeleton (from dM
1569	IDP dMRI TBSS MO Superior fronto-occipital fasciculus R	Mean MO (diffusion tensor mode) in superior fronto-occipital fasciculus (right) on FA (fractional anisotropy) skeleton (fro
1570	IDP dMRI TBSS MO Superior fronto-occipital fasciculus L	Mean MO (diffusion tensor mode) in superior fronto-occipital fasciculus (left) on FA (fractional anisotropy) skeleton (from
1571	IDP dMRI TBSS MO Uncinate fasciculus R	Mean MO (diffusion tensor mode) in uncinate fasciculus (right) on FA (fractional anisotropy) skeleton (from dMRI data)
1572	IDP dMRI TBSS MO Uncinate fasciculus L	Mean MO (diffusion tensor mode) in uncinate fasciculus (left) on FA (fractional anisotropy) skeleton (from dMRI data)
1573	IDP dMRI TBSS MO Tapetum R	Mean MO (diffusion tensor mode) in tapetum (right) on FA (fractional anisotropy) skeleton (from dMRI data)
1574	IDP dMRI TBSS MO Tapetum L	Mean MO (diffusion tensor mode) in tapetum (left) on FA (fractional anisotropy) skeleton (from dMRI data)
1575	IDP dMRI ProbtrackX MO ar l	Weighted-mean MO (diffusion tensor mode) in tract left acoustic radiation (from dMRI data)
1576	IDP dMRI ProbtrackX MO ar r	Weighted-mean MO (diffusion tensor mode) in tract right acoustic radiation (from dMRI data)
1577	IDP dMRI ProbtrackX MO atr l	Weighted-mean MO (diffusion tensor mode) in tract left anterior thalamic radiation (from dMRI data)
1578	IDP dMRI ProbtrackX MO atr r	Weighted-mean MO (diffusion tensor mode) in tract right anterior thalamic radiation (from dMRI data)
1579	IDP dMRI ProbtrackX MO cgc l	Weighted-mean MO (diffusion tensor mode) in tract left cingulate gyrus part of cingulum (from dMRI data)
1580	IDP dMRI ProbtrackX MO cgc r	Weighted-mean MO (diffusion tensor mode) in tract right cingulate gyrus part of cingulum (from dMRI data)
1581	IDP dMRI ProbtrackX MO cgh l	Weighted-mean MO (diffusion tensor mode) in tract left parahippocampal part of cingulum (from dMRI data)
1582	IDP dMRI ProbtrackX MO cgh r	Weighted-mean MO (diffusion tensor mode) in tract right parahippocampal part of cingulum (from dMRI data)
1583	IDP dMRI ProbtrackX MO cst l	Weighted-mean MO (diffusion tensor mode) in tract left corticospinal tract (from dMRI data)
1584	IDP dMRI ProbtrackX MO cst r	Weighted-mean MO (diffusion tensor mode) in tract right corticospinal tract (from dMRI data)
1585	IDP dMRI ProbtrackX MO fma	Weighted-mean MO (diffusion tensor mode) in tract forceps major (from dMRI data)
1586	IDP dMRI ProbtrackX MO fmi	Weighted-mean MO (diffusion tensor mode) in tract forceps minor (from dMRI data)
1587	IDP dMRI ProbtrackX MO ifo l	Weighted-mean MO (diffusion tensor mode) in tract left inferior fronto-occipital fasciculus (from dMRI data)
1588	IDP dMRI ProbtrackX MO ifo r	Weighted-mean MO (diffusion tensor mode) in tract right inferior fronto-occipital fasciculus (from dMRI data)
1589	IDP dMRI ProbtrackX MO ilf l	Weighted-mean MO (diffusion tensor mode) in tract left inferior longitudinal fasciculus (from dMRI data)
1590	IDP dMRI ProbtrackX MO ilf r	Weighted-mean MO (diffusion tensor mode) in tract right inferior longitudinal fasciculus (from dMRI data)
1591	IDP dMRI ProbtrackX MO mcp	Weighted-mean MO (diffusion tensor mode) in tract middle cerebellar peduncle (from dMRI data)
1592	IDP dMRI ProbtrackX MO ml l	Weighted-mean MO (diffusion tensor mode) in tract left medial lemniscus (from dMRI data)
1593	IDP dMRI ProbtrackX MO ml r	Weighted-mean MO (diffusion tensor mode) in tract right medial lemniscus (from dMRI data)
1594	IDP dMRI ProbtrackX MO ptr l	Weighted-mean MO (diffusion tensor mode) in tract left posterior thalamic radiation (from dMRI data)

IDP number	IDP short name	IDP description
1595	IDP dMRI ProbtrackX MO ptr r	Weighted-mean MO (diffusion tensor mode) in tract right posterior thalamic radiation (from dMRI data)
1596	IDP dMRI ProbtrackX MO slf l	Weighted-mean MO (diffusion tensor mode) in tract left superior longitudinal fasciculus (from dMRI data)
1597	IDP dMRI ProbtrackX MO slf r	Weighted-mean MO (diffusion tensor mode) in tract right superior longitudinal fasciculus (from dMRI data)
1598	IDP dMRI ProbtrackX MO str l	Weighted-mean MO (diffusion tensor mode) in tract left superior thalamic radiation (from dMRI data)
1599	IDP dMRI ProbtrackX MO str r	Weighted-mean MO (diffusion tensor mode) in tract right superior thalamic radiation (from dMRI data)
1600	IDP dMRI ProbtrackX MO unc l	Weighted-mean MO (diffusion tensor mode) in tract left uncinate fasciculus (from dMRI data)
1601	IDP dMRI ProbtrackX MO unc r	Weighted-mean MO (diffusion tensor mode) in tract right uncinate fasciculus (from dMRI data)
1602	IDP dMRI TBSS MD Middle cerebellar peduncle	Mean MD (mean diffusivity) in middle cerebellar peduncle on FA (fractional anisotropy) skeleton (from dMRI data)
1603	IDP dMRI TBSS MD Pontine crossing tract	Mean MD (mean diffusivity) in pontine crossing tract on FA (fractional anisotropy) skeleton (from dMRI data)
1604	IDP dMRI TBSS MD Genu of corpus callosum	Mean MD (mean diffusivity) in genu of corpus callosum on FA (fractional anisotropy) skeleton (from dMRI data)
1605	IDP dMRI TBSS MD Body of corpus callosum	Mean MD (mean diffusivity) in body of corpus callosum on FA (fractional anisotropy) skeleton (from dMRI data)
1606	IDP dMRI TBSS MD Splenium of corpus callosum	Mean MD (mean diffusivity) in splenium of corpus callosum on FA (fractional anisotropy) skeleton (from dMRI data)
1607	IDP dMRI TBSS MD Fornix	Mean MD (mean diffusivity) in fornix on FA (fractional anisotropy) skeleton (from dMRI data)
1608	IDP dMRI TBSS MD Corticospinal tract R	Mean MD (mean diffusivity) in corticospinal tract (right) on FA (fractional anisotropy) skeleton (from dMRI data)
1609	IDP dMRI TBSS MD Corticospinal tract L	Mean MD (mean diffusivity) in corticospinal tract (left) on FA (fractional anisotropy) skeleton (from dMRI data)
1610	IDP dMRI TBSS MD Medial lemniscus R	Mean MD (mean diffusivity) in medial lemniscus (right) on FA (fractional anisotropy) skeleton (from dMRI data)
1611	IDP dMRI TBSS MD Medial lemniscus L	Mean MD (mean diffusivity) in medial lemniscus (left) on FA (fractional anisotropy) skeleton (from dMRI data)
1612	IDP dMRI TBSS MD Inferior cerebellar peduncle R	Mean MD (mean diffusivity) in inferior cerebellar peduncle (right) on FA (fractional anisotropy) skeleton (from dMRI data
1613	IDP dMRI TBSS MD Inferior cerebellar peduncle L	Mean MD (mean diffusivity) in inferior cerebellar peduncle (left) on FA (fractional anisotropy) skeleton (from dMRI data)
1614	IDP dMRI TBSS MD Superior cerebellar peduncle R	Mean MD (mean diffusivity) in superior cerebellar peduncle (right) on FA (fractional anisotropy) skeleton (from dMRI dat
1615	IDP dMRI TBSS MD Superior cerebellar peduncle L	Mean MD (mean diffusivity) in superior cerebellar peduncle (left) on FA (fractional anisotropy) skeleton (from dMRI data
1616	IDP dMRI TBSS MD Cerebral peduncle R	Mean MD (mean diffusivity) in cerebral peduncle (right) on FA (fractional anisotropy) skeleton (from dMRI data)
1617	IDP dMRI TBSS MD Cerebral peduncle L	Mean MD (mean diffusivity) in cerebral peduncle (left) on FA (fractional anisotropy) skeleton (from dMRI data)
1618	IDP dMRI TBSS MD Anterior limb of internal capsule R	Mean MD (mean diffusivity) in anterior limb of internal capsule (right) on FA (fractional anisotropy) skeleton (from dMRI
1619	IDP dMRI TBSS MD Anterior limb of internal capsule L	Mean MD (mean diffusivity) in anterior limb of internal capsule (left) on FA (fractional anisotropy) skeleton (from dMRI d
1620	IDP dMRI TBSS MD Posterior limb of internal capsule R	Mean MD (mean diffusivity) in posterior limb of internal capsule (right) on FA (fractional anisotropy) skeleton (from dMR
1621	IDP dMRI TBSS MD Posterior limb of internal capsule L	Mean MD (mean diffusivity) in posterior limb of internal capsule (left) on FA (fractional anisotropy) skeleton (from dMRI
1622	IDP dMRI TBSS MD Retrolenticular part of internal capsule R	Mean MD (mean diffusivity) in retrolenticular part of internal capsule (right) on FA (fractional anisotropy) skeleton (from
1623	IDP dMRI TBSS MD Retrolenticular part of internal capsule L	Mean MD (mean diffusivity) in retrolenticular part of internal capsule (left) on FA (fractional anisotropy) skeleton (from dI
1624	IDP dMRI TBSS MD Anterior corona radiata R	Mean MD (mean diffusivity) in anterior corona radiata (right) on FA (fractional anisotropy) skeleton (from dMRI data)
1625	IDP dMRI TBSS MD Anterior corona radiata L	Mean MD (mean diffusivity) in anterior corona radiata (left) on FA (fractional anisotropy) skeleton (from dMRI data)
1626	IDP dMRI TBSS MD Superior corona radiata R	Mean MD (mean diffusivity) in superior corona radiata (right) on FA (fractional anisotropy) skeleton (from dMRI data)
1627	IDP dMRI TBSS MD Superior corona radiata L	Mean MD (mean diffusivity) in superior corona radiata (left) on FA (fractional anisotropy) skeleton (from dMRI data)
1628	IDP dMRI TBSS MD Posterior corona radiata R	Mean MD (mean diffusivity) in posterior corona radiata (right) on FA (fractional anisotropy) skeleton (from dMRI data)
1629	IDP dMRI TBSS MD Posterior corona radiata L	Mean MD (mean diffusivity) in posterior corona radiata (left) on FA (fractional anisotropy) skeleton (from dMRI data)
1630	IDP dMRI TBSS MD Posterior thalamic radiation R	Mean MD (mean diffusivity) in posterior thalamic radiation (right) on FA (fractional anisotropy) skeleton (from dMRI data
1631	IDP dMRI TBSS MD Posterior thalamic radiation L	Mean MD (mean diffusivity) in posterior thalamic radiation (left) on FA (fractional anisotropy) skeleton (from dMRI data)
1632	IDP dMRI TBSS MD Sagittal stratum R	Mean MD (mean diffusivity) in sagittal stratum (right) on FA (fractional anisotropy) skeleton (from dMRI data)
1633	IDP dMRI TBSS MD Sagittal stratum L	Mean MD (mean diffusivity) in sagittal stratum (left) on FA (fractional anisotropy) skeleton (from dMRI data)
1634	IDP dMRI TBSS MD External capsule R	Mean MD (mean diffusivity) in external capsule (right) on FA (fractional anisotropy) skeleton (from dMRI data)
1635	IDP dMRI TBSS MD External capsule L	Mean MD (mean diffusivity) in external capsule (left) on FA (fractional anisotropy) skeleton (from dMRI data)
1636	IDP dMRI TBSS MD Cingulum cingulate gyrus R	Mean MD (mean diffusivity) in cingulum cingulate gyrus (right) on FA (fractional anisotropy) skeleton (from dMRI data)
1637	IDP dMRI TBSS MD Cingulum cingulate gyrus L	Mean MD (mean diffusivity) in cingulum cingulate gyrus (left) on FA (fractional anisotropy) skeleton (from dMRI data)
1638	IDP dMRI TBSS MD Cingulum hippocampus R	Mean MD (mean diffusivity) in cingulum hippocampus (right) on FA (fractional anisotropy) skeleton (from dMRI data)
1639	IDP dMRI TBSS MD Cingulum hippocampus L	Mean MD (mean diffusivity) in cingulum hippocampus (left) on FA (fractional anisotropy) skeleton (from dMRI data)
1640	IDP dMRI TBSS MD Fornix cres+Stria terminalis R	Mean MD (mean diffusivity) in fornix cres+stria terminalis (right) on FA (fractional anisotropy) skeleton (from dMRI data)
1641	IDP dMRI TBSS MD Fornix cres+Stria terminalis L	Mean MD (mean diffusivity) in fornix cres+stria terminalis (left) on FA (fractional anisotropy) skeleton (from dMRI data)
1642	IDP dMRI TBSS MD Superior longitudinal fasciculus R	Mean MD (mean diffusivity) in superior longitudinal fasciculus (right) on FA (fractional anisotropy) skeleton (from dMRI c
1643	IDP dMRI TBSS MD Superior longitudinal fasciculus L	Mean MD (mean diffusivity) in superior longitudinal fasciculus (left) on FA (fractional anisotropy) skeleton (from dMRI da
1644	IDP dMRI TBSS MD Superior fronto-occipital fasciculus R	Mean MD (mean diffusivity) in superior fronto-occipital fasciculus (right) on FA (fractional anisotropy) skeleton (from dMI
1645	IDP dMRI TBSS MD Superior fronto-occipital fasciculus L	Mean MD (mean diffusivity) in superior fronto-occipital fasciculus (left) on FA (fractional anisotropy) skeleton (from dMR
1646	IDP dMRI TBSS MD Uncinate fasciculus R	Mean MD (mean diffusivity) in uncinate fasciculus (right) on FA (fractional anisotropy) skeleton (from dMRI data)
1647	IDP dMRI TBSS MD Uncinate fasciculus L	Mean MD (mean diffusivity) in uncinate fasciculus (left) on FA (fractional anisotropy) skeleton (from dMRI data)
1648	IDP dMRI TBSS MD Tapetum R	Mean MD (mean diffusivity) in tapetum (right) on FA (fractional anisotropy) skeleton (from dMRI data)
1649	IDP dMRI TBSS MD Tapetum L	Mean MD (mean diffusivity) in tapetum (left) on FA (fractional anisotropy) skeleton (from dMRI data)
1650	IDP dMRI TBSS L1 Middle cerebellar peduncle	Mean L1 in middle cerebellar peduncle on FA (fractional anisotropy) skeleton (from dMRI data)
1651	IDP dMRI TBSS L1 Pontine crossing tract	Mean L1 in pontine crossing tract on FA (fractional anisotropy) skeleton (from dMRI data)

IDP number	IDP short name	IDP description
1652	IDP dMRI TBSS L1 Genu of corpus callosum	Mean L1 in genu of corpus callosum on FA (fractional anisotropy) skeleton (from dMRI data)
1653	IDP dMRI TBSS L1 Body of corpus callosum	Mean L1 in body of corpus callosum on FA (fractional anisotropy) skeleton (from dMRI data)
1654	IDP dMRI TBSS L1 Splenium of corpus callosum	Mean L1 in splenium of corpus callosum on FA (fractional anisotropy) skeleton (from dMRI data)
1655	IDP dMRI TBSS L1 Fornix	Mean L1 in fornix on FA (fractional anisotropy) skeleton (from dMRI data)
1656	IDP dMRI TBSS L1 Corticospinal tract R	Mean L1 in corticospinal tract (right) on FA (fractional anisotropy) skeleton (from dMRI data)
1657	IDP dMRI TBSS L1 Corticospinal tract L	Mean L1 in corticospinal tract (left) on FA (fractional anisotropy) skeleton (from dMRI data)
1658	IDP dMRI TBSS L1 Medial lemniscus R	Mean L1 in medial lemniscus (right) on FA (fractional anisotropy) skeleton (from dMRI data)
1659	IDP dMRI TBSS L1 Medial lemniscus L	Mean L1 in medial lemniscus (left) on FA (fractional anisotropy) skeleton (from dMRI data)
1660	IDP dMRI TBSS L1 Inferior cerebellar peduncle R	Mean L1 in inferior cerebellar peduncle (right) on FA (fractional anisotropy) skeleton (from dMRI data)
1661	IDP dMRI TBSS L1 Inferior cerebellar peduncle L	Mean L1 in inferior cerebellar peduncle (left) on FA (fractional anisotropy) skeleton (from dMRI data)
1662	IDP dMRI TBSS L1 Superior cerebellar peduncle R	Mean L1 in superior cerebellar peduncle (right) on FA (fractional anisotropy) skeleton (from dMRI data)
1663	IDP dMRI TBSS L1 Superior cerebellar peduncle L	Mean L1 in superior cerebellar peduncle (left) on FA (fractional anisotropy) skeleton (from dMRI data)
1664	IDP dMRI TBSS L1 Cerebral peduncle R	Mean L1 in cerebral peduncle (right) on FA (fractional anisotropy) skeleton (from dMRI data)
1665	IDP dMRI TBSS L1 Cerebral peduncle L	Mean L1 in cerebral peduncle (left) on FA (fractional anisotropy) skeleton (from dMRI data)
1666	IDP dMRI TBSS L1 Anterior limb of internal capsule R	Mean L1 in anterior limb of internal capsule (right) on FA (fractional anisotropy) skeleton (from dMRI data)
1667	IDP dMRI TBSS L1 Anterior limb of internal capsule L	Mean L1 in anterior limb of internal capsule (left) on FA (fractional anisotropy) skeleton (from dMRI data)
1668	IDP dMRI TBSS L1 Posterior limb of internal capsule R	Mean L1 in posterior limb of internal capsule (right) on FA (fractional anisotropy) skeleton (from dMRI data)
1669	IDP dMRI TBSS L1 Posterior limb of internal capsule L	Mean L1 in posterior limb of internal capsule (left) on FA (fractional anisotropy) skeleton (from dMRI data)
1670	IDP dMRI TBSS L1 Retrolenticular part of internal capsule R	Mean L1 in retrolenticular part of internal capsule (right) on FA (fractional anisotropy) skeleton (from dMRI data)
1671	IDP dMRI TBSS L1 Retrolenticular part of internal capsule L	Mean L1 in retrolenticular part of internal capsule (left) on FA (fractional anisotropy) skeleton (from dMRI data)
1672	IDP dMRI TBSS L1 Anterior corona radiata R	Mean L1 in anterior corona radiata (right) on FA (fractional anisotropy) skeleton (from dMRI data)
1673	IDP dMRI TBSS L1 Anterior corona radiata L	Mean L1 in anterior corona radiata (left) on FA (fractional anisotropy) skeleton (from dMRI data)
1674	IDP dMRI TBSS L1 Superior corona radiata R	Mean L1 in superior corona radiata (right) on FA (fractional anisotropy) skeleton (from dMRI data)
1675	IDP dMRI TBSS L1 Superior corona radiata L	Mean L1 in superior corona radiata (left) on FA (fractional anisotropy) skeleton (from dMRI data)
1676	IDP dMRI TBSS L1 Posterior corona radiata R	Mean L1 in posterior corona radiata (right) on FA (fractional anisotropy) skeleton (from dMRI data)
1677	IDP dMRI TBSS L1 Posterior corona radiata L	Mean L1 in posterior corona radiata (left) on FA (fractional anisotropy) skeleton (from dMRI data)
1678	IDP dMRI TBSS L1 Posterior thalamic radiation R	Mean L1 in posterior thalamic radiation (right) on FA (fractional anisotropy) skeleton (from dMRI data)
1679	IDP dMRI TBSS L1 Posterior thalamic radiation L	Mean L1 in posterior thalamic radiation (left) on FA (fractional anisotropy) skeleton (from dMRI data)
1680	IDP dMRI TBSS L1 Sagittal stratum R	Mean L1 in sagittal stratum (right) on FA (fractional anisotropy) skeleton (from dMRI data)
1681	IDP dMRI TBSS L1 Sagittal stratum L	Mean L1 in sagittal stratum (left) on FA (fractional anisotropy) skeleton (from dMRI data)
1682	IDP dMRI TBSS L1 External capsule R	Mean L1 in external capsule (right) on FA (fractional anisotropy) skeleton (from dMRI data)
1683	IDP dMRI TBSS L1 External capsule L	Mean L1 in external capsule (left) on FA (fractional anisotropy) skeleton (from dMRI data)
1684	IDP dMRI TBSS L1 Cingulum cingulate gyrus R	Mean L1 in cingulum cingulate gyrus (right) on FA (fractional anisotropy) skeleton (from dMRI data)
1685	IDP dMRI TBSS L1 Cingulum cingulate gyrus L	Mean L1 in cingulum cingulate gyrus (left) on FA (fractional anisotropy) skeleton (from dMRI data)
1686	IDP dMRI TBSS L1 Cingulum hippocampus R	Mean L1 in cingulum hippocampus (right) on FA (fractional anisotropy) skeleton (from dMRI data)
1687	IDP dMRI TBSS L1 Cingulum hippocampus L	Mean L1 in cingulum hippocampus (left) on FA (fractional anisotropy) skeleton (from dMRI data)
1688	IDP dMRI TBSS L1 Fornix cres+Stria terminalis R	Mean L1 in fornix cres+stria terminalis (right) on FA (fractional anisotropy) skeleton (from dMRI data)
1689	IDP dMRI TBSS L1 Fornix cres+Stria terminalis L	Mean L1 in fornix cres+stria terminalis (left) on FA (fractional anisotropy) skeleton (from dMRI data)
1690	IDP dMRI TBSS L1 Superior longitudinal fasciculus R	Mean L1 in superior longitudinal fasciculus (right) on FA (fractional anisotropy) skeleton (from dMRI data)
1691	IDP dMRI TBSS L1 Superior longitudinal fasciculus L	Mean L1 in superior longitudinal fasciculus (left) on FA (fractional anisotropy) skeleton (from dMRI data)
1692	IDP dMRI TBSS L1 Superior fronto-occipital fasciculus R	Mean L1 in superior fronto-occipital fasciculus (right) on FA (fractional anisotropy) skeleton (from dMRI data)
1693	IDP dMRI TBSS L1 Superior fronto-occipital fasciculus L	Mean L1 in superior fronto-occipital fasciculus (left) on FA (fractional anisotropy) skeleton (from dMRI data)
1694	IDP dMRI TBSS L1 Uncinate fasciculus R	Mean L1 in uncinate fasciculus (right) on FA (fractional anisotropy) skeleton (from dMRI data)
1695	IDP dMRI TBSS L1 Uncinate fasciculus L	Mean L1 in uncinate fasciculus (left) on FA (fractional anisotropy) skeleton (from dMRI data)
1696	IDP dMRI TBSS L1 Tapetum R	Mean L1 in tapetum (right) on FA (fractional anisotropy) skeleton (from dMRI data)
1697	IDP dMRI TBSS L1 Tapetum L	Mean L1 in tapetum (left) on FA (fractional anisotropy) skeleton (from dMRI data)
1698	IDP dMRI TBSS L2 Middle cerebellar peduncle	Mean L2 in middle cerebellar peduncle on FA (fractional anisotropy) skeleton (from dMRI data)
1699	IDP dMRI TBSS L2 Pontine crossing tract	Mean L2 in pontine crossing tract on FA (fractional anisotropy) skeleton (from dMRI data)
1700	IDP dMRI TBSS L2 Genu of corpus callosum	Mean L2 in genu of corpus callosum on FA (fractional anisotropy) skeleton (from dMRI data)
1701	IDP dMRI TBSS L2 Body of corpus callosum	Mean L2 in body of corpus callosum on FA (fractional anisotropy) skeleton (from dMRI data)
1702	IDP dMRI TBSS L2 Splenium of corpus callosum	Mean L2 in splenium of corpus callosum on FA (fractional anisotropy) skeleton (from dMRI data)
1703	IDP dMRI TBSS L2 Fornix	Mean L2 in fornix on FA (fractional anisotropy) skeleton (from dMRI data)
1704	IDP dMRI TBSS L2 Corticospinal tract R	Mean L2 in corticospinal tract (right) on FA (fractional anisotropy) skeleton (from dMRI data)
1705	IDP dMRI TBSS L2 Corticospinal tract L	Mean L2 in corticospinal tract (left) on FA (fractional anisotropy) skeleton (from dMRI data)
1706	IDP dMRI TBSS L2 Medial lemniscus R	Mean L2 in medial lemniscus (right) on FA (fractional anisotropy) skeleton (from dMRI data)
1707	IDP dMRI TBSS L2 Medial lemniscus L	Mean L2 in medial lemniscus (left) on FA (fractional anisotropy) skeleton (from dMRI data)
1708	IDP dMRI TBSS L2 Inferior cerebellar peduncle R	Mean L2 in inferior cerebellar peduncle (right) on FA (fractional anisotropy) skeleton (from dMRI data)

IDP number	IDP short name	IDP description
1709	IDP dMRI TBSS L2 Inferior cerebellar peduncle L	Mean L2 in inferior cerebellar peduncle (left) on FA (fractional anisotropy) skeleton (from dMRI data)
1710	IDP dMRI TBSS L2 Superior cerebellar peduncle R	Mean L2 in superior cerebellar peduncle (right) on FA (fractional anisotropy) skeleton (from dMRI data)
1711	IDP dMRI TBSS L2 Superior cerebellar peduncle L	Mean L2 in superior cerebellar peduncle (left) on FA (fractional anisotropy) skeleton (from dMRI data)
1712	IDP dMRI TBSS L2 Cerebral peduncle R	Mean L2 in cerebral peduncle (right) on FA (fractional anisotropy) skeleton (from dMRI data)
1713	IDP dMRI TBSS L2 Cerebral peduncle L	Mean L2 in cerebral peduncle (left) on FA (fractional anisotropy) skeleton (from dMRI data)
1714	IDP dMRI TBSS L2 Anterior limb of internal capsule R	Mean L2 in anterior limb of internal capsule (right) on FA (fractional anisotropy) skeleton (from dMRI data)
1715	IDP dMRI TBSS L2 Anterior limb of internal capsule L	Mean L2 in anterior limb of internal capsule (left) on FA (fractional anisotropy) skeleton (from dMRI data)
1716	IDP dMRI TBSS L2 Posterior limb of internal capsule R	Mean L2 in posterior limb of internal capsule (right) on FA (fractional anisotropy) skeleton (from dMRI data)
1717	IDP dMRI TBSS L2 Posterior limb of internal capsule L	Mean L2 in posterior limb of internal capsule (left) on FA (fractional anisotropy) skeleton (from dMRI data)
1718	IDP dMRI TBSS L2 Retrolenticular part of internal capsule R	Mean L2 in retrolenticular part of internal capsule (right) on FA (fractional anisotropy) skeleton (from dMRI data)
1719	IDP dMRI TBSS L2 Retrolenticular part of internal capsule L	Mean L2 in retrolenticular part of internal capsule (left) on FA (fractional anisotropy) skeleton (from dMRI data)
1720	IDP dMRI TBSS L2 Anterior corona radiata R	Mean L2 in anterior corona radiata (right) on FA (fractional anisotropy) skeleton (from dMRI data)
1721	IDP dMRI TBSS L2 Anterior corona radiata L	Mean L2 in anterior corona radiata (left) on FA (fractional anisotropy) skeleton (from dMRI data)
1722	IDP dMRI TBSS L2 Superior corona radiata R	Mean L2 in superior corona radiata (right) on FA (fractional anisotropy) skeleton (from dMRI data)
1723	IDP dMRI TBSS L2 Superior corona radiata L	Mean L2 in superior corona radiata (left) on FA (fractional anisotropy) skeleton (from dMRI data)
1724	IDP dMRI TBSS L2 Posterior corona radiata R	Mean L2 in posterior corona radiata (right) on FA (fractional anisotropy) skeleton (from dMRI data)
1725	IDP dMRI TBSS L2 Posterior corona radiata L	Mean L2 in posterior corona radiata (left) on FA (fractional anisotropy) skeleton (from dMRI data)
1726	IDP dMRI TBSS L2 Posterior thalamic radiation R	Mean L2 in posterior thalamic radiation (right) on FA (fractional anisotropy) skeleton (from dMRI data)
1727	IDP dMRI TBSS L2 Posterior thalamic radiation L	Mean L2 in posterior thalamic radiation (left) on FA (fractional anisotropy) skeleton (from dMRI data)
1728	IDP dMRI TBSS L2 Sagittal stratum R	Mean L2 in sagittal stratum (right) on FA (fractional anisotropy) skeleton (from dMRI data)
1729	IDP dMRI TBSS L2 Sagittal stratum L	Mean L2 in sagittal stratum (left) on FA (fractional anisotropy) skeleton (from dMRI data)
1730	IDP dMRI TBSS L2 External capsule R	Mean L2 in external capsule (right) on FA (fractional anisotropy) skeleton (from dMRI data)
1731	IDP dMRI TBSS L2 External capsule L	Mean L2 in external capsule (left) on FA (fractional anisotropy) skeleton (from dMRI data)
1732	IDP dMRI TBSS L2 Cingulum cingulate gyrus R	Mean L2 in cingulum cingulate gyrus (right) on FA (fractional anisotropy) skeleton (from dMRI data)
1733	IDP dMRI TBSS L2 Cingulum cingulate gyrus L	Mean L2 in cingulum cingulate gyrus (left) on FA (fractional anisotropy) skeleton (from dMRI data)
1734	IDP dMRI TBSS L2 Cingulum hippocampus R	Mean L2 in cingulum hippocampus (right) on FA (fractional anisotropy) skeleton (from dMRI data)
1735	IDP dMRI TBSS L2 Cingulum hippocampus L	Mean L2 in cingulum hippocampus (left) on FA (fractional anisotropy) skeleton (from dMRI data)
1736	IDP dMRI TBSS L2 Fornix cres+Stria terminalis R	Mean L2 in fornix cres+stria terminalis (right) on FA (fractional anisotropy) skeleton (from dMRI data)
1737	IDP dMRI TBSS L2 Fornix cres+Stria terminalis L	Mean L2 in fornix cres+stria terminalis (left) on FA (fractional anisotropy) skeleton (from dMRI data)
1738	IDP dMRI TBSS L2 Superior longitudinal fasciculus R	Mean L2 in superior longitudinal fasciculus (right) on FA (fractional anisotropy) skeleton (from dMRI data)
1739	IDP dMRI TBSS L2 Superior longitudinal fasciculus L	Mean L2 in superior longitudinal fasciculus (left) on FA (fractional anisotropy) skeleton (from dMRI data)
1740	IDP dMRI TBSS L2 Superior fronto-occipital fasciculus R	Mean L2 in superior fronto-occipital fasciculus (right) on FA (fractional anisotropy) skeleton (from dMRI data)
1741	IDP dMRI TBSS L2 Superior fronto-occipital fasciculus L	Mean L2 in superior fronto-occipital fasciculus (left) on FA (fractional anisotropy) skeleton (from dMRI data)
1742	IDP dMRI TBSS L2 Uncinate fasciculus R	Mean L2 in uncinate fasciculus (right) on FA (fractional anisotropy) skeleton (from dMRI data)
1743	IDP dMRI TBSS L2 Uncinate fasciculus L	Mean L2 in uncinate fasciculus (left) on FA (fractional anisotropy) skeleton (from dMRI data)
1744	IDP dMRI TBSS L2 Tapetum R	Mean L2 in tapetum (right) on FA (fractional anisotropy) skeleton (from dMRI data)
1745	IDP dMRI TBSS L2 Tapetum L	Mean L2 in tapetum (left) on FA (fractional anisotropy) skeleton (from dMRI data)
1746	IDP dMRI TBSS L3 Middle cerebellar peduncle	Mean L3 in middle cerebellar peduncle on FA (fractional anisotropy) skeleton (from dMRI data)
1747	IDP dMRI TBSS L3 Pontine crossing tract	Mean L3 in pontine crossing tract on FA (fractional anisotropy) skeleton (from dMRI data)
1748	IDP dMRI TBSS L3 Genu of corpus callosum	Mean L3 in genu of corpus callosum on FA (fractional anisotropy) skeleton (from dMRI data)
1749	IDP dMRI TBSS L3 Body of corpus callosum	Mean L3 in body of corpus callosum on FA (fractional anisotropy) skeleton (from dMRI data)
1750	IDP dMRI TBSS L3 Splenium of corpus callosum	Mean L3 in splenium of corpus callosum on FA (fractional anisotropy) skeleton (from dMRI data)
1751	IDP dMRI TBSS L3 Fornix	Mean L3 in fornix on FA (fractional anisotropy) skeleton (from dMRI data)
1752	IDP dMRI TBSS L3 Corticospinal tract R	Mean L3 in corticospinal tract (right) on FA (fractional anisotropy) skeleton (from dMRI data)
1753	IDP dMRI TBSS L3 Corticospinal tract L	Mean L3 in corticospinal tract (left) on FA (fractional anisotropy) skeleton (from dMRI data)
1754	IDP dMRI TBSS L3 Medial lemniscus R	Mean L3 in medial lemniscus (right) on FA (fractional anisotropy) skeleton (from dMRI data)
1755	IDP dMRI TBSS L3 Medial lemniscus L	Mean L3 in medial lemniscus (left) on FA (fractional anisotropy) skeleton (from dMRI data)
1756	IDP dMRI TBSS L3 Inferior cerebellar peduncle R	Mean L3 in inferior cerebellar peduncle (right) on FA (fractional anisotropy) skeleton (from dMRI data)
1757	IDP dMRI TBSS L3 Inferior cerebellar peduncle L	Mean L3 in inferior cerebellar peduncle (left) on FA (fractional anisotropy) skeleton (from dMRI data)
1758	IDP dMRI TBSS L3 Superior cerebellar peduncle R	Mean L3 in superior cerebellar peduncle (right) on FA (fractional anisotropy) skeleton (from dMRI data)
1759	IDP dMRI TBSS L3 Superior cerebellar peduncle L	Mean L3 in superior cerebellar peduncle (left) on FA (fractional anisotropy) skeleton (from dMRI data)
1760	IDP dMRI TBSS L3 Cerebral peduncle R	Mean L3 in cerebral peduncle (right) on FA (fractional anisotropy) skeleton (from dMRI data)
1761	IDP dMRI TBSS L3 Cerebral peduncle L	Mean L3 in cerebral peduncle (left) on FA (fractional anisotropy) skeleton (from dMRI data)
1762	IDP dMRI TBSS L3 Anterior limb of internal capsule R	Mean L3 in anterior limb of internal capsule (right) on FA (fractional anisotropy) skeleton (from dMRI data)
1763	IDP dMRI TBSS L3 Anterior limb of internal capsule L	Mean L3 in anterior limb of internal capsule (left) on FA (fractional anisotropy) skeleton (from dMRI data)
1764	IDP dMRI TBSS L3 Posterior limb of internal capsule R	Mean L3 in posterior limb of internal capsule (right) on FA (fractional anisotropy) skeleton (from dMRI data)
1765	IDP dMRI TBSS L3 Posterior limb of internal capsule L	Mean L3 in posterior limb of internal capsule (left) on FA (fractional anisotropy) skeleton (from dMRI data)

IDP number	IDP short name	IDP description
1766	IDP dMRI TBSS L3 Retrolenticular part of internal capsule R	Mean L3 in retrolenticular part of internal capsule (right) on FA (fractional anisotropy) skeleton (from dMRI data)
1767	IDP dMRI TBSS L3 Retrolenticular part of internal capsule L	Mean L3 in retrolenticular part of internal capsule (left) on FA (fractional anisotropy) skeleton (from dMRI data)
1768	IDP dMRI TBSS L3 Anterior corona radiata R	Mean L3 in anterior corona radiata (right) on FA (fractional anisotropy) skeleton (from dMRI data)
1769	IDP dMRI TBSS L3 Anterior corona radiata L	Mean L3 in anterior corona radiata (left) on FA (fractional anisotropy) skeleton (from dMRI data)
1770	IDP dMRI TBSS L3 Superior corona radiata R	Mean L3 in superior corona radiata (right) on FA (fractional anisotropy) skeleton (from dMRI data)
1771	IDP dMRI TBSS L3 Superior corona radiata L	Mean L3 in superior corona radiata (left) on FA (fractional anisotropy) skeleton (from dMRI data)
1772	IDP dMRI TBSS L3 Posterior corona radiata R	Mean L3 in posterior corona radiata (right) on FA (fractional anisotropy) skeleton (from dMRI data)
1773	IDP dMRI TBSS L3 Posterior corona radiata L	Mean L3 in posterior corona radiata (left) on FA (fractional anisotropy) skeleton (from dMRI data)
1774	IDP dMRI TBSS L3 Posterior thalamic radiation R	Mean L3 in posterior thalamic radiation (right) on FA (fractional anisotropy) skeleton (from dMRI data)
1775	IDP dMRI TBSS L3 Posterior thalamic radiation L	Mean L3 in posterior thalamic radiation (left) on FA (fractional anisotropy) skeleton (from dMRI data)
1776	IDP dMRI TBSS L3 Sagittal stratum R	Mean L3 in sagittal stratum (right) on FA (fractional anisotropy) skeleton (from dMRI data)
1777	IDP dMRI TBSS L3 Sagittal stratum L	Mean L3 in sagittal stratum (left) on FA (fractional anisotropy) skeleton (from dMRI data)
1778	IDP dMRI TBSS L3 External capsule R	Mean L3 in external capsule (right) on FA (fractional anisotropy) skeleton (from dMRI data)
1779	IDP dMRI TBSS L3 External capsule L	Mean L3 in external capsule (left) on FA (fractional anisotropy) skeleton (from dMRI data)
1780	IDP dMRI TBSS L3 Cingulum cingulate gyrus R	Mean L3 in cingulum cingulate gyrus (right) on FA (fractional anisotropy) skeleton (from dMRI data)
1781	IDP dMRI TBSS L3 Cingulum cingulate gyrus L	Mean L3 in cingulum cingulate gyrus (left) on FA (fractional anisotropy) skeleton (from dMRI data)
1782	IDP dMRI TBSS L3 Cingulum hippocampus R	Mean L3 in cingulum hippocampus (right) on FA (fractional anisotropy) skeleton (from dMRI data)
1783	IDP dMRI TBSS L3 Cingulum hippocampus L	Mean L3 in cingulum hippocampus (left) on FA (fractional anisotropy) skeleton (from dMRI data)
1784	IDP dMRI TBSS L3 Fornix cres+Stria terminalis R	Mean L3 in fornix cres+stria terminalis (right) on FA (fractional anisotropy) skeleton (from dMRI data)
1785	IDP dMRI TBSS L3 Fornix cres+Stria terminalis L	Mean L3 in fornix cres+stria terminalis (left) on FA (fractional anisotropy) skeleton (from dMRI data)
1786	IDP dMRI TBSS L3 Superior longitudinal fasciculus R	Mean L3 in superior longitudinal fasciculus (right) on FA (fractional anisotropy) skeleton (from dMRI data)
1787	IDP dMRI TBSS L3 Superior longitudinal fasciculus L	Mean L3 in superior longitudinal fasciculus (left) on FA (fractional anisotropy) skeleton (from dMRI data)
1788	IDP dMRI TBSS L3 Superior fronto-occipital fasciculus R	Mean L3 in superior fronto-occipital fasciculus (right) on FA (fractional anisotropy) skeleton (from dMRI data)
1789	IDP dMRI TBSS L3 Superior fronto-occipital fasciculus L	Mean L3 in superior fronto-occipital fasciculus (left) on FA (fractional anisotropy) skeleton (from dMRI data)
1790	IDP dMRI TBSS L3 Uncinate fasciculus R	Mean L3 in uncinate fasciculus (right) on FA (fractional anisotropy) skeleton (from dMRI data)
1791	IDP dMRI TBSS L3 Uncinate fasciculus L	Mean L3 in uncinate fasciculus (left) on FA (fractional anisotropy) skeleton (from dMRI data)
1792	IDP dMRI TBSS L3 Tapetum R	Mean L3 in tapetum (right) on FA (fractional anisotropy) skeleton (from dMRI data)
1793	IDP dMRI TBSS L3 Tapetum L	Mean L3 in tapetum (left) on FA (fractional anisotropy) skeleton (from dMRI data)
1794	IDP dMRI ProbtrackX MD ar l	Weighted-mean MD (mean diffusivity) in tract left acoustic radiation (from dMRI data)
1795	IDP dMRI ProbtrackX MD ar r	Weighted-mean MD (mean diffusivity) in tract right acoustic radiation (from dMRI data)
1796	IDP dMRI ProbtrackX MD atr l	Weighted-mean MD (mean diffusivity) in tract left anterior thalamic radiation (from dMRI data)
1797	IDP dMRI ProbtrackX MD atr r	Weighted-mean MD (mean diffusivity) in tract right anterior thalamic radiation (from dMRI data)
1798	IDP dMRI ProbtrackX MD cgc l	Weighted-mean MD (mean diffusivity) in tract left cingulate gyrus part of cingulum (from dMRI data)
1799	IDP dMRI ProbtrackX MD cgc r	Weighted-mean MD (mean diffusivity) in tract right cingulate gyrus part of cingulum (from dMRI data)
1800	IDP dMRI ProbtrackX MD cgh l	Weighted-mean MD (mean diffusivity) in tract left parahippocampal part of cingulum (from dMRI data)
1801	IDP dMRI ProbtrackX MD cgh r	Weighted-mean MD (mean diffusivity) in tract right parahippocampal part of cingulum (from dMRI data)
1802	IDP dMRI ProbtrackX MD cst l	Weighted-mean MD (mean diffusivity) in tract left corticospinal tract (from dMRI data)
1803	IDP dMRI ProbtrackX MD cst r	Weighted-mean MD (mean diffusivity) in tract right corticospinal tract (from dMRI data)
1804	IDP dMRI ProbtrackX MD fma	Weighted-mean MD (mean diffusivity) in tract forceps major (from dMRI data)
1805	IDP dMRI ProbtrackX MD fmi	Weighted-mean MD (mean diffusivity) in tract forceps minor (from dMRI data)
1806	IDP dMRI ProbtrackX MD ifo l	Weighted-mean MD (mean diffusivity) in tract left inferior fronto-occipital fasciculus (from dMRI data)
1807	IDP dMRI ProbtrackX MD ifo r	Weighted-mean MD (mean diffusivity) in tract right inferior fronto-occipital fasciculus (from dMRI data)
1808	IDP dMRI ProbtrackX MD ilf l	Weighted-mean MD (mean diffusivity) in tract left inferior longitudinal fasciculus (from dMRI data)
1809	IDP dMRI ProbtrackX MD ilf r	Weighted-mean MD (mean diffusivity) in tract right inferior longitudinal fasciculus (from dMRI data)
1810	IDP dMRI ProbtrackX MD mcp	Weighted-mean MD (mean diffusivity) in tract middle cerebellar peduncle (from dMRI data)
1811	IDP dMRI ProbtrackX MD ml l	Weighted-mean MD (mean diffusivity) in tract left medial lemniscus (from dMRI data)
1812	IDP dMRI ProbtrackX MD ml r	Weighted-mean MD (mean diffusivity) in tract right medial lemniscus (from dMRI data)
1813	IDP dMRI ProbtrackX MD ptr l	Weighted-mean MD (mean diffusivity) in tract left posterior thalamic radiation (from dMRI data)
1814	IDP dMRI ProbtrackX MD ptr r	Weighted-mean MD (mean diffusivity) in tract right posterior thalamic radiation (from dMRI data)
1815	IDP dMRI ProbtrackX MD slf l	Weighted-mean MD (mean diffusivity) in tract left superior longitudinal fasciculus (from dMRI data)
1816	IDP dMRI ProbtrackX MD slf r	Weighted-mean MD (mean diffusivity) in tract right superior longitudinal fasciculus (from dMRI data)
1817	IDP dMRI ProbtrackX MD str l	Weighted-mean MD (mean diffusivity) in tract left superior thalamic radiation (from dMRI data)
1818	IDP dMRI ProbtrackX MD str r	Weighted-mean MD (mean diffusivity) in tract right superior thalamic radiation (from dMRI data)
1819	IDP dMRI ProbtrackX MD unc l	Weighted-mean MD (mean diffusivity) in tract left uncinate fasciculus (from dMRI data)
1820	IDP dMRI ProbtrackX MD unc r	Weighted-mean MD (mean diffusivity) in tract right uncinate fasciculus (from dMRI data)
1821	IDP dMRI ProbtrackX L1 ar l	Weighted-mean L1 in tract left acoustic radiation (from dMRI data)
1822	IDP dMRI ProbtrackX L1 ar r	Weighted-mean L1 in tract right acoustic radiation (from dMRI data)

IDP number	IDP short name	IDP description
1823	IDP dMRI ProbtrackX L1 atr l	Weighted-mean L1 in tract left anterior thalamic radiation (from dMRI data)
1824	IDP dMRI ProbtrackX L1 atr r	Weighted-mean L1 in tract right anterior thalamic radiation (from dMRI data)
1825	IDP dMRI ProbtrackX L1 cgc l	Weighted-mean L1 in tract left cingulate gyrus part of cingulum (from dMRI data)
1826	IDP dMRI ProbtrackX L1 cgc r	Weighted-mean L1 in tract right cingulate gyrus part of cingulum (from dMRI data)
1827	IDP dMRI ProbtrackX L1 cgh l	Weighted-mean L1 in tract left parahippocampal part of cingulum (from dMRI data)
1828	IDP dMRI ProbtrackX L1 cgh r	Weighted-mean L1 in tract right parahippocampal part of cingulum (from dMRI data)
1829	IDP dMRI ProbtrackX L1 cst l	Weighted-mean L1 in tract left corticospinal tract (from dMRI data)
1830	IDP dMRI ProbtrackX L1 cst r	Weighted-mean L1 in tract right corticospinal tract (from dMRI data)
1831	IDP dMRI ProbtrackX L1 fma	Weighted-mean L1 in tract forceps major (from dMRI data)
1832	IDP dMRI ProbtrackX L1 fmi	Weighted-mean L1 in tract forceps minor (from dMRI data)
1833	IDP dMRI ProbtrackX L1 ifo l	Weighted-mean L1 in tract left inferior fronto-occipital fasciculus (from dMRI data)
1834	IDP dMRI ProbtrackX L1 ifo r	Weighted-mean L1 in tract right inferior fronto-occipital fasciculus (from dMRI data)
1835	IDP dMRI ProbtrackX L1 ilf l	Weighted-mean L1 in tract left inferior longitudinal fasciculus (from dMRI data)
1836	IDP dMRI ProbtrackX L1 ilf r	Weighted-mean L1 in tract right inferior longitudinal fasciculus (from dMRI data)
1837	IDP dMRI ProbtrackX L1 mcp	Weighted-mean L1 in tract middle cerebellar peduncle (from dMRI data)
1838	IDP dMRI ProbtrackX L1 ml l	Weighted-mean L1 in tract left medial lemniscus (from dMRI data)
1839	IDP dMRI ProbtrackX L1 ml r	Weighted-mean L1 in tract right medial lemniscus (from dMRI data)
1840	IDP dMRI ProbtrackX L1 ptr l	Weighted-mean L1 in tract left posterior thalamic radiation (from dMRI data)
1841	IDP dMRI ProbtrackX L1 ptr r	Weighted-mean L1 in tract right posterior thalamic radiation (from dMRI data)
1842	IDP dMRI ProbtrackX L1 slf l	Weighted-mean L1 in tract left superior longitudinal fasciculus (from dMRI data)
1843	IDP dMRI ProbtrackX L1 slf r	Weighted-mean L1 in tract right superior longitudinal fasciculus (from dMRI data)
1844	IDP dMRI ProbtrackX L1 str l	Weighted-mean L1 in tract left superior thalamic radiation (from dMRI data)
1845	IDP dMRI ProbtrackX L1 str r	Weighted-mean L1 in tract right superior thalamic radiation (from dMRI data)
1846	IDP dMRI ProbtrackX L1 unc l	Weighted-mean L1 in tract left uncinate fasciculus (from dMRI data)
1847	IDP dMRI ProbtrackX L1 unc r	Weighted-mean L1 in tract right uncinate fasciculus (from dMRI data)
1848	IDP dMRI ProbtrackX L2 ar l	Weighted-mean L2 in tract left acoustic radiation (from dMRI data)
1849	IDP dMRI ProbtrackX L2 ar r	Weighted-mean L2 in tract right acoustic radiation (from dMRI data)
1850	IDP dMRI ProbtrackX L2 atr l	Weighted-mean L2 in tract left anterior thalamic radiation (from dMRI data)
1851	IDP dMRI ProbtrackX L2 atr r	Weighted-mean L2 in tract right anterior thalamic radiation (from dMRI data)
1852	IDP dMRI ProbtrackX L2 cgc l	Weighted-mean L2 in tract left cingulate gyrus part of cingulum (from dMRI data)
1853	IDP dMRI ProbtrackX L2 cgc r	Weighted-mean L2 in tract right cingulate gyrus part of cingulum (from dMRI data)
1854	IDP dMRI ProbtrackX L2 cgh l	Weighted-mean L2 in tract left parahippocampal part of cingulum (from dMRI data)
1855	IDP dMRI ProbtrackX L2 cgh r	Weighted-mean L2 in tract right parahippocampal part of cingulum (from dMRI data)
1856	IDP dMRI ProbtrackX L2 cst l	Weighted-mean L2 in tract left corticospinal tract (from dMRI data)
1857	IDP dMRI ProbtrackX L2 cst r	Weighted-mean L2 in tract right corticospinal tract (from dMRI data)
1858	IDP dMRI ProbtrackX L2 fma	Weighted-mean L2 in tract forceps major (from dMRI data)
1859	IDP dMRI ProbtrackX L2 fmi	Weighted-mean L2 in tract forceps minor (from dMRI data)
1860	IDP dMRI ProbtrackX L2 ifo l	Weighted-mean L2 in tract left inferior fronto-occipital fasciculus (from dMRI data)
1861	IDP dMRI ProbtrackX L2 ifo r	Weighted-mean L2 in tract right inferior fronto-occipital fasciculus (from dMRI data)
1862	IDP dMRI ProbtrackX L2 ilf l	Weighted-mean L2 in tract left inferior longitudinal fasciculus (from dMRI data)
1863	IDP dMRI ProbtrackX L2 ilf r	Weighted-mean L2 in tract right inferior longitudinal fasciculus (from dMRI data)
1864	IDP dMRI ProbtrackX L2 mcp	Weighted-mean L2 in tract middle cerebellar peduncle (from dMRI data)
1865	IDP dMRI ProbtrackX L2 ml l	Weighted-mean L2 in tract left medial lemniscus (from dMRI data)
1866	IDP dMRI ProbtrackX L2 ml r	Weighted-mean L2 in tract right medial lemniscus (from dMRI data)
1867	IDP dMRI ProbtrackX L2 ptr l	Weighted-mean L2 in tract left posterior thalamic radiation (from dMRI data)
1868	IDP dMRI ProbtrackX L2 ptr r	Weighted-mean L2 in tract right posterior thalamic radiation (from dMRI data)
1869	IDP dMRI ProbtrackX L2 slf l	Weighted-mean L2 in tract left superior longitudinal fasciculus (from dMRI data)
1870	IDP dMRI ProbtrackX L2 slf r	Weighted-mean L2 in tract right superior longitudinal fasciculus (from dMRI data)
1871	IDP dMRI ProbtrackX L2 str l	Weighted-mean L2 in tract left superior thalamic radiation (from dMRI data)
1872	IDP dMRI ProbtrackX L2 str r	Weighted-mean L2 in tract right superior thalamic radiation (from dMRI data)
1873	IDP dMRI ProbtrackX L2 unc l	Weighted-mean L2 in tract left uncinate fasciculus (from dMRI data)
1874	IDP dMRI ProbtrackX L2 unc r	Weighted-mean L2 in tract right uncinate fasciculus (from dMRI data)
1875	IDP dMRI ProbtrackX L3 ar l	Weighted-mean L3 in tract left acoustic radiation (from dMRI data)
1876	IDP dMRI ProbtrackX L3 ar r	Weighted-mean L3 in tract right acoustic radiation (from dMRI data)
1877	IDP dMRI ProbtrackX L3 atr l	Weighted-mean L3 in tract left anterior thalamic radiation (from dMRI data)
1878	IDP dMRI ProbtrackX L3 atr r	Weighted-mean L3 in tract right anterior thalamic radiation (from dMRI data)
1879	IDP dMRI ProbtrackX L3 cgc l	Weighted-mean L3 in tract left cingulate gyrus part of cingulum (from dMRI data)

IDP number	IDP short name	IDP description
1880	IDP dMRI ProbtrackX L3 cgc r	Weighted-mean L3 in tract right cingulate gyrus part of cingulum (from dMRI data)
1881	IDP dMRI ProbtrackX L3 cgh l	Weighted-mean L3 in tract left parahippocampal part of cingulum (from dMRI data)
1882	IDP dMRI ProbtrackX L3 cgh r	Weighted-mean L3 in tract right parahippocampal part of cingulum (from dMRI data)
1883	IDP dMRI ProbtrackX L3 cst l	Weighted-mean L3 in tract left corticospinal tract (from dMRI data)
1884	IDP dMRI ProbtrackX L3 cst r	Weighted-mean L3 in tract right corticospinal tract (from dMRI data)
1885	IDP dMRI ProbtrackX L3 fma	Weighted-mean L3 in tract forceps major (from dMRI data)
1886	IDP dMRI ProbtrackX L3 fmi	Weighted-mean L3 in tract forceps minor (from dMRI data)
1887	IDP dMRI ProbtrackX L3 ifo l	Weighted-mean L3 in tract left inferior fronto-occipital fasciculus (from dMRI data)
1888	IDP dMRI ProbtrackX L3 ifo r	Weighted-mean L3 in tract right inferior fronto-occipital fasciculus (from dMRI data)
1889	IDP dMRI ProbtrackX L3 ilf l	Weighted-mean L3 in tract left inferior longitudinal fasciculus (from dMRI data)
1890	IDP dMRI ProbtrackX L3 ilf r	Weighted-mean L3 in tract right inferior longitudinal fasciculus (from dMRI data)
1891	IDP dMRI ProbtrackX L3 mcp	Weighted-mean L3 in tract middle cerebellar peduncle (from dMRI data)
1892	IDP dMRI ProbtrackX L3 ml l	Weighted-mean L3 in tract left medial lemniscus (from dMRI data)
1893	IDP dMRI ProbtrackX L3 ml r	Weighted-mean L3 in tract right medial lemniscus (from dMRI data)
1894	IDP dMRI ProbtrackX L3 ptr l	Weighted-mean L3 in tract left posterior thalamic radiation (from dMRI data)
1895	IDP dMRI ProbtrackX L3 ptr r	Weighted-mean L3 in tract right posterior thalamic radiation (from dMRI data)
1896	IDP dMRI ProbtrackX L3 slf l	Weighted-mean L3 in tract left superior longitudinal fasciculus (from dMRI data)
1897	IDP dMRI ProbtrackX L3 slf r	Weighted-mean L3 in tract right superior longitudinal fasciculus (from dMRI data)
1898	IDP dMRI ProbtrackX L3 str l	Weighted-mean L3 in tract left superior thalamic radiation (from dMRI data)
1899	IDP dMRI ProbtrackX L3 str r	Weighted-mean L3 in tract right superior thalamic radiation (from dMRI data)
1900	IDP dMRI ProbtrackX L3 unc l	Weighted-mean L3 in tract left uncinate fasciculus (from dMRI data)
1901	IDP dMRI ProbtrackX L3 unc r	Weighted-mean L3 in tract right uncinate fasciculus (from dMRI data)
1902	IDP dMRI TBSS ICVF Middle cerebellar peduncle	Mean ICVF (intra-cellular volume fraction) in middle cerebellar peduncle on FA (fractional anisotropy) skeleton (from dM
1903	IDP dMRI TBSS ICVF Pontine crossing tract	Mean ICVF (intra-cellular volume fraction) in pontine crossing tract on FA (fractional anisotropy) skeleton (from dMRI da
1904	IDP dMRI TBSS ICVF Genu of corpus callosum	Mean ICVF (intra-cellular volume fraction) in genu of corpus callosum on FA (fractional anisotropy) skeleton (from dMRI
1905	IDP dMRI TBSS ICVF Body of corpus callosum	Mean ICVF (intra-cellular volume fraction) in body of corpus callosum on FA (fractional anisotropy) skeleton (from dMRI
1906	IDP dMRI TBSS ICVF Splenium of corpus callosum	Mean ICVF (intra-cellular volume fraction) in splenium of corpus callosum on FA (fractional anisotropy) skeleton (from d
1907	IDP dMRI TBSS ICVF Fornix	Mean ICVF (intra-cellular volume fraction) in fornix on FA (fractional anisotropy) skeleton (from dMRI data)
1908	IDP dMRI TBSS ICVF Corticospinal tract R	Mean ICVF (intra-cellular volume fraction) in corticospinal tract (right) on FA (fractional anisotropy) skeleton (from dMRI
1909	IDP dMRI TBSS ICVF Corticospinal tract L	Mean ICVF (intra-cellular volume fraction) in corticospinal tract (left) on FA (fractional anisotropy) skeleton (from dMRI d
1910	IDP dMRI TBSS ICVF Medial lemniscus R	Mean ICVF (intra-cellular volume fraction) in medial lemniscus (right) on FA (fractional anisotropy) skeleton (from dMRI
1911	IDP dMRI TBSS ICVF Medial lemniscus L	Mean ICVF (intra-cellular volume fraction) in medial lemniscus (left) on FA (fractional anisotropy) skeleton (from dMRI d
1912	IDP dMRI TBSS ICVF Inferior cerebellar peduncle R	Mean ICVF (intra-cellular volume fraction) in inferior cerebellar peduncle (right) on FA (fractional anisotropy) skeleton (fr
1913	IDP dMRI TBSS ICVF Inferior cerebellar peduncle L	Mean ICVF (intra-cellular volume fraction) in inferior cerebellar peduncle (left) on FA (fractional anisotropy) skeleton (fro
1914	IDP dMRI TBSS ICVF Superior cerebellar peduncle R	Mean ICVF (intra-cellular volume fraction) in superior cerebellar peduncle (right) on FA (fractional anisotropy) skeleton (
1915	IDP dMRI TBSS ICVF Superior cerebellar peduncle L	Mean ICVF (intra-cellular volume fraction) in superior cerebellar peduncle (left) on FA (fractional anisotropy) skeleton (fr
1916	IDP dMRI TBSS ICVF Cerebral peduncle R	Mean ICVF (intra-cellular volume fraction) in cerebral peduncle (right) on FA (fractional anisotropy) skeleton (from dMRI
1917	IDP dMRI TBSS ICVF Cerebral peduncle L	Mean ICVF (intra-cellular volume fraction) in cerebral peduncle (left) on FA (fractional anisotropy) skeleton (from dMRI d
1918	IDP dMRI TBSS ICVF Anterior limb of internal capsule R	Mean ICVF (intra-cellular volume fraction) in anterior limb of internal capsule (right) on FA (fractional anisotropy) skeleto
1919	IDP dMRI TBSS ICVF Anterior limb of internal capsule L	Mean ICVF (intra-cellular volume fraction) in anterior limb of internal capsule (left) on FA (fractional anisotropy) skeleton
1920	IDP dMRI TBSS ICVF Posterior limb of internal capsule R	Mean ICVF (intra-cellular volume fraction) in posterior limb of internal capsule (right) on FA (fractional anisotropy) skelet
1921	IDP dMRI TBSS ICVF Posterior limb of internal capsule L	Mean ICVF (intra-cellular volume fraction) in posterior limb of internal capsule (left) on FA (fractional anisotropy) skeleto
1922	IDP dMRI TBSS ICVF Retrolenticular part of internal capsule R	Mean ICVF (intra-cellular volume fraction) in retrolenticular part of internal capsule (right) on FA (fractional anisotropy) s
1923	IDP dMRI TBSS ICVF Retrolenticular part of internal capsule L	Mean ICVF (intra-cellular volume fraction) in retrolenticular part of internal capsule (left) on FA (fractional anisotropy) sk
1924	IDP dMRI TBSS ICVF Anterior corona radiata R	Mean ICVF (intra-cellular volume fraction) in anterior corona radiata (right) on FA (fractional anisotropy) skeleton (from d
1925	IDP dMRI TBSS ICVF Anterior corona radiata L	Mean ICVF (intra-cellular volume fraction) in anterior corona radiata (left) on FA (fractional anisotropy) skeleton (from dN
1926	IDP dMRI TBSS ICVF Superior corona radiata R	Mean ICVF (intra-cellular volume fraction) in superior corona radiata (right) on FA (fractional anisotropy) skeleton (from
1927	IDP dMRI TBSS ICVF Superior corona radiata L	Mean ICVF (intra-cellular volume fraction) in superior corona radiata (left) on FA (fractional anisotropy) skeleton (from d
1928	IDP dMRI TBSS ICVF Posterior corona radiata R	Mean ICVF (intra-cellular volume fraction) in posterior corona radiata (right) on FA (fractional anisotropy) skeleton (from
1929	IDP dMRI TBSS ICVF Posterior corona radiata L	Mean ICVF (intra-cellular volume fraction) in posterior corona radiata (left) on FA (fractional anisotropy) skeleton (from d
1930	IDP dMRI TBSS ICVF Posterior thalamic radiation R	Mean ICVF (intra-cellular volume fraction) in posterior thalamic radiation (right) on FA (fractional anisotropy) skeleton (fr
1931	IDP dMRI TBSS ICVF Posterior thalamic radiation L	Mean ICVF (intra-cellular volume fraction) in posterior thalamic radiation (left) on FA (fractional anisotropy) skeleton (fro
1932	IDP dMRI TBSS ICVF Sagittal stratum R	Mean ICVF (intra-cellular volume fraction) in sagittal stratum (right) on FA (fractional anisotropy) skeleton (from dMRI da
1933	IDP dMRI TBSS ICVF Sagittal stratum L	Mean ICVF (intra-cellular volume fraction) in sagittal stratum (left) on FA (fractional anisotropy) skeleton (from dMRI dat
1934	IDP dMRI TBSS ICVF External capsule R	Mean ICVF (intra-cellular volume fraction) in external capsule (right) on FA (fractional anisotropy) skeleton (from dMRI d
1935	IDP dMRI TBSS ICVF External capsule L	Mean ICVF (intra-cellular volume fraction) in external capsule (left) on FA (fractional anisotropy) skeleton (from dMRI da
1936	IDP dMRI TBSS ICVF Cingulum cingulate gyrus R	Mean ICVF (intra-cellular volume fraction) in cingulum cingulate gyrus (right) on FA (fractional anisotropy) skeleton (fror

IDP number	IDP short name	IDP description
1937	IDP dMRI TBSS ICVF Cingulum cingulate gyrus L	Mean ICVF (intra-cellular volume fraction) in cingulum cingulate gyrus (left) on FA (fractional anisotropy) skeleton (from
1938	IDP dMRI TBSS ICVF Cingulum hippocampus R	Mean ICVF (intra-cellular volume fraction) in cingulum hippocampus (right) on FA (fractional anisotropy) skeleton (from
1939	IDP dMRI TBSS ICVF Cingulum hippocampus L	Mean ICVF (intra-cellular volume fraction) in cingulum hippocampus (left) on FA (fractional anisotropy) skeleton (from d
1940	IDP dMRI TBSS ICVF Fornix cres+Stria terminalis R	Mean ICVF (intra-cellular volume fraction) in fornix cres+stria terminalis (right) on FA (fractional anisotropy) skeleton (fro
1941	IDP dMRI TBSS ICVF Fornix cres+Stria terminalis L	Mean ICVF (intra-cellular volume fraction) in fornix cres+stria terminalis (left) on FA (fractional anisotropy) skeleton (from
1942	IDP dMRI TBSS ICVF Superior longitudinal fasciculus R	Mean ICVF (intra-cellular volume fraction) in superior longitudinal fasciculus (right) on FA (fractional anisotropy) skeleto
1943	IDP dMRI TBSS ICVF Superior longitudinal fasciculus L	Mean ICVF (intra-cellular volume fraction) in superior longitudinal fasciculus (left) on FA (fractional anisotropy) skeleton
1944	IDP dMRI TBSS ICVF Superior fronto-occipital fasciculus R	Mean ICVF (intra-cellular volume fraction) in superior fronto-occipital fasciculus (right) on FA (fractional anisotropy) skel
1945	IDP dMRI TBSS ICVF Superior fronto-occipital fasciculus L	Mean ICVF (intra-cellular volume fraction) in superior fronto-occipital fasciculus (left) on FA (fractional anisotropy) skele
1946	IDP dMRI TBSS ICVF Uncinate fasciculus R	Mean ICVF (intra-cellular volume fraction) in uncinate fasciculus (right) on FA (fractional anisotropy) skeleton (from dMF
1947	IDP dMRI TBSS ICVF Uncinate fasciculus L	Mean ICVF (intra-cellular volume fraction) in uncinate fasciculus (left) on FA (fractional anisotropy) skeleton (from dMRI
1948	IDP dMRI TBSS ICVF Tapetum R	Mean ICVF (intra-cellular volume fraction) in tapetum (right) on FA (fractional anisotropy) skeleton (from dMRI data)
1949	IDP dMRI TBSS ICVF Tapetum L	Mean ICVF (intra-cellular volume fraction) in tapetum (left) on FA (fractional anisotropy) skeleton (from dMRI data)
1950	IDP dMRI ProbtrackX ICVF ar l	Weighted-mean ICVF (intra-cellular volume fraction) in tract left acoustic radiation (from dMRI data)
1951	IDP dMRI ProbtrackX ICVF ar r	Weighted-mean ICVF (intra-cellular volume fraction) in tract right acoustic radiation (from dMRI data)
1952	IDP dMRI ProbtrackX ICVF atr l	Weighted-mean ICVF (intra-cellular volume fraction) in tract left anterior thalamic radiation (from dMRI data)
1953	IDP dMRI ProbtrackX ICVF atr r	Weighted-mean ICVF (intra-cellular volume fraction) in tract right anterior thalamic radiation (from dMRI data)
1954	IDP dMRI ProbtrackX ICVF cgc l	Weighted-mean ICVF (intra-cellular volume fraction) in tract left cingulate gyrus part of cingulum (from dMRI data)
1955	IDP dMRI ProbtrackX ICVF cgc r	Weighted-mean ICVF (intra-cellular volume fraction) in tract right cingulate gyrus part of cingulum (from dMRI data)
1956	IDP dMRI ProbtrackX ICVF cgh l	Weighted-mean ICVF (intra-cellular volume fraction) in tract left parahippocampal part of cingulum (from dMRI data)
1957	IDP dMRI ProbtrackX ICVF cgh r	Weighted-mean ICVF (intra-cellular volume fraction) in tract right parahippocampal part of cingulum (from dMRI data)
1958	IDP dMRI ProbtrackX ICVF cst l	Weighted-mean ICVF (intra-cellular volume fraction) in tract left corticospinal tract (from dMRI data)
1959	IDP dMRI ProbtrackX ICVF cst r	Weighted-mean ICVF (intra-cellular volume fraction) in tract right corticospinal tract (from dMRI data)
1960	IDP dMRI ProbtrackX ICVF fma	Weighted-mean ICVF (intra-cellular volume fraction) in tract forceps major (from dMRI data)
1961	IDP dMRI ProbtrackX ICVF fmi	Weighted-mean ICVF (intra-cellular volume fraction) in tract forceps minor (from dMRI data)
1962	IDP dMRI ProbtrackX ICVF ifo l	Weighted-mean ICVF (intra-cellular volume fraction) in tract left inferior fronto-occipital fasciculus (from dMRI data)
1963	IDP dMRI ProbtrackX ICVF ifo r	Weighted-mean ICVF (intra-cellular volume fraction) in tract right inferior fronto-occipital fasciculus (from dMRI data)
1964	IDP dMRI ProbtrackX ICVF ilf l	Weighted-mean ICVF (intra-cellular volume fraction) in tract left inferior longitudinal fasciculus (from dMRI data)
1965	IDP dMRI ProbtrackX ICVF ilf r	Weighted-mean ICVF (intra-cellular volume fraction) in tract right inferior longitudinal fasciculus (from dMRI data)
1966	IDP dMRI ProbtrackX ICVF mcp	Weighted-mean ICVF (intra-cellular volume fraction) in tract middle cerebellar peduncle (from dMRI data)
1967	IDP dMRI ProbtrackX ICVF ml l	Weighted-mean ICVF (intra-cellular volume fraction) in tract left medial lemniscus (from dMRI data)
1968	IDP dMRI ProbtrackX ICVF ml r	Weighted-mean ICVF (intra-cellular volume fraction) in tract right medial lemniscus (from dMRI data)
1969	IDP dMRI ProbtrackX ICVF ptr l	Weighted-mean ICVF (intra-cellular volume fraction) in tract left posterior thalamic radiation (from dMRI data)
1970	IDP dMRI ProbtrackX ICVF ptr r	Weighted-mean ICVF (intra-cellular volume fraction) in tract right posterior thalamic radiation (from dMRI data)
1971	IDP dMRI ProbtrackX ICVF slf l	Weighted-mean ICVF (intra-cellular volume fraction) in tract left superior longitudinal fasciculus (from dMRI data)
1972	IDP dMRI ProbtrackX ICVF slf r	Weighted-mean ICVF (intra-cellular volume fraction) in tract right superior longitudinal fasciculus (from dMRI data)
1973	IDP dMRI ProbtrackX ICVF str l	Weighted-mean ICVF (intra-cellular volume fraction) in tract left superior thalamic radiation (from dMRI data)
1974	IDP dMRI ProbtrackX ICVF str r	Weighted-mean ICVF (intra-cellular volume fraction) in tract right superior thalamic radiation (from dMRI data)
1975	IDP dMRI ProbtrackX ICVF unc l	Weighted-mean ICVF (intra-cellular volume fraction) in tract left uncinate fasciculus (from dMRI data)
1976	IDP dMRI ProbtrackX ICVF unc r	Weighted-mean ICVF (intra-cellular volume fraction) in tract right uncinate fasciculus (from dMRI data)
1977	IDP dMRI TBSS OD Middle cerebellar peduncle	Mean OD (orientation dispersion index) in middle cerebellar peduncle on FA (fractional anisotropy) skeleton (from dMRI
1978	IDP dMRI TBSS OD Pontine crossing tract	Mean OD (orientation dispersion index) in pontine crossing tract on FA (fractional anisotropy) skeleton (from dMRI data)
1979	IDP dMRI TBSS OD Genu of corpus callosum	Mean OD (orientation dispersion index) in genu of corpus callosum on FA (fractional anisotropy) skeleton (from dMRI da
1980	IDP dMRI TBSS OD Body of corpus callosum	Mean OD (orientation dispersion index) in body of corpus callosum on FA (fractional anisotropy) skeleton (from dMRI da
1981	IDP dMRI TBSS OD Splenium of corpus callosum	Mean OD (orientation dispersion index) in splenium of corpus callosum on FA (fractional anisotropy) skeleton (from dMI
1982	IDP dMRI TBSS OD Fornix	Mean OD (orientation dispersion index) in fornix on FA (fractional anisotropy) skeleton (from dMRI data)
1983	IDP dMRI TBSS OD Corticospinal tract R	Mean OD (orientation dispersion index) in corticospinal tract (right) on FA (fractional anisotropy) skeleton (from dMRI da
1984	IDP dMRI TBSS OD Corticospinal tract L	Mean OD (orientation dispersion index) in corticospinal tract (left) on FA (fractional anisotropy) skeleton (from dMRI data
1985	IDP dMRI TBSS OD Medial lemniscus R	Mean OD (orientation dispersion index) in medial lemniscus (right) on FA (fractional anisotropy) skeleton (from dMRI da
1986	IDP dMRI TBSS OD Medial lemniscus L	Mean OD (orientation dispersion index) in medial lemniscus (left) on FA (fractional anisotropy) skeleton (from dMRI data
1987	IDP dMRI TBSS OD Inferior cerebellar peduncle R	Mean OD (orientation dispersion index) in inferior cerebellar peduncle (right) on FA (fractional anisotropy) skeleton (fron
1988	IDP dMRI TBSS OD Inferior cerebellar peduncle L	Mean OD (orientation dispersion index) in inferior cerebellar peduncle (left) on FA (fractional anisotropy) skeleton (from
1989	IDP dMRI TBSS OD Superior cerebellar peduncle R	Mean OD (orientation dispersion index) in superior cerebellar peduncle (right) on FA (fractional anisotropy) skeleton (fro
1990	IDP dMRI TBSS OD Superior cerebellar peduncle L	Mean OD (orientation dispersion index) in superior cerebellar peduncle (left) on FA (fractional anisotropy) skeleton (fron
1991	IDP dMRI TBSS OD Cerebral peduncle R	Mean OD (orientation dispersion index) in cerebral peduncle (right) on FA (fractional anisotropy) skeleton (from dMRI da
1992	IDP dMRI TBSS OD Cerebral peduncle L	Mean OD (orientation dispersion index) in cerebral peduncle (left) on FA (fractional anisotropy) skeleton (from dMRI dat
1993	IDP dMRI TBSS OD Anterior limb of internal capsule R	Mean OD (orientation dispersion index) in anterior limb of internal capsule (right) on FA (fractional anisotropy) skeleton

IDP number	IDP short name	IDP description
1994	IDP dMRI TBSS OD Anterior limb of internal capsule L	Mean OD (orientation dispersion index) in anterior limb of internal capsule (left) on FA (fractional anisotropy) skeleton (f
1995	IDP dMRI TBSS OD Posterior limb of internal capsule R	Mean OD (orientation dispersion index) in posterior limb of internal capsule (right) on FA (fractional anisotropy) skeletor
1996	IDP dMRI TBSS OD Posterior limb of internal capsule L	Mean OD (orientation dispersion index) in posterior limb of internal capsule (left) on FA (fractional anisotropy) skeleton (
1997	IDP dMRI TBSS OD Retrolenticular part of internal capsule R	Mean OD (orientation dispersion index) in retrolenticular part of internal capsule (right) on FA (fractional anisotropy) ske
1998	IDP dMRI TBSS OD Retrolenticular part of internal capsule L	Mean OD (orientation dispersion index) in retrolenticular part of internal capsule (left) on FA (fractional anisotropy) skele
1999	IDP dMRI TBSS OD Anterior corona radiata R	Mean OD (orientation dispersion index) in anterior corona radiata (right) on FA (fractional anisotropy) skeleton (from dM
2000	IDP dMRI TBSS OD Anterior corona radiata L	Mean OD (orientation dispersion index) in anterior corona radiata (left) on FA (fractional anisotropy) skeleton (from dMF
2001	IDP dMRI TBSS OD Superior corona radiata R	Mean OD (orientation dispersion index) in superior corona radiata (right) on FA (fractional anisotropy) skeleton (from dM
2002	IDP dMRI TBSS OD Superior corona radiata L	Mean OD (orientation dispersion index) in superior corona radiata (left) on FA (fractional anisotropy) skeleton (from dMF
2003	IDP dMRI TBSS OD Posterior corona radiata R	Mean OD (orientation dispersion index) in posterior corona radiata (right) on FA (fractional anisotropy) skeleton (from dI
2004	IDP dMRI TBSS OD Posterior corona radiata L	Mean OD (orientation dispersion index) in posterior corona radiata (left) on FA (fractional anisotropy) skeleton (from dM
2005	IDP dMRI TBSS OD Posterior thalamic radiation R	Mean OD (orientation dispersion index) in posterior thalamic radiation (right) on FA (fractional anisotropy) skeleton (fron
2006	IDP dMRI TBSS OD Posterior thalamic radiation L	Mean OD (orientation dispersion index) in posterior thalamic radiation (left) on FA (fractional anisotropy) skeleton (from
2007	IDP dMRI TBSS OD Sagittal stratum R	Mean OD (orientation dispersion index) in sagittal stratum (right) on FA (fractional anisotropy) skeleton (from dMRI data
2008	IDP dMRI TBSS OD Sagittal stratum L	Mean OD (orientation dispersion index) in sagittal stratum (left) on FA (fractional anisotropy) skeleton (from dMRI data)
2009	IDP dMRI TBSS OD External capsule R	Mean OD (orientation dispersion index) in external capsule (right) on FA (fractional anisotropy) skeleton (from dMRI dat
2010	IDP dMRI TBSS OD External capsule L	Mean OD (orientation dispersion index) in external capsule (left) on FA (fractional anisotropy) skeleton (from dMRI data)
2011	IDP dMRI TBSS OD Cingulum cingulate gyrus R	Mean OD (orientation dispersion index) in cingulum cingulate gyrus (right) on FA (fractional anisotropy) skeleton (from c
2012	IDP dMRI TBSS OD Cingulum cingulate gyrus L	Mean OD (orientation dispersion index) in cingulum cingulate gyrus (left) on FA (fractional anisotropy) skeleton (from dM
2013	IDP dMRI TBSS OD Cingulum hippocampus R	Mean OD (orientation dispersion index) in cingulum hippocampus (right) on FA (fractional anisotropy) skeleton (from dM
2014	IDP dMRI TBSS OD Cingulum hippocampus L	Mean OD (orientation dispersion index) in cingulum hippocampus (left) on FA (fractional anisotropy) skeleton (from dMF
2015	IDP dMRI TBSS OD Fornix cres+Stria terminalis R	Mean OD (orientation dispersion index) in fornix cres+stria terminalis (right) on FA (fractional anisotropy) skeleton (from
2016	IDP dMRI TBSS OD Fornix cres+Stria terminalis L	Mean OD (orientation dispersion index) in fornix cres+stria terminalis (left) on FA (fractional anisotropy) skeleton (from c
2017	IDP dMRI TBSS OD Superior longitudinal fasciculus R	Mean OD (orientation dispersion index) in superior longitudinal fasciculus (right) on FA (fractional anisotropy) skeleton (
2018	IDP dMRI TBSS OD Superior longitudinal fasciculus L	Mean OD (orientation dispersion index) in superior longitudinal fasciculus (left) on FA (fractional anisotropy) skeleton (fr
2019	IDP dMRI TBSS OD Superior fronto-occipital fasciculus R	Mean OD (orientation dispersion index) in superior fronto-occipital fasciculus (right) on FA (fractional anisotropy) skeletc
2020	IDP dMRI TBSS OD Superior fronto-occipital fasciculus L	Mean OD (orientation dispersion index) in superior fronto-occipital fasciculus (left) on FA (fractional anisotropy) skeletor
2021	IDP dMRI TBSS OD Uncinate fasciculus R	Mean OD (orientation dispersion index) in uncinate fasciculus (right) on FA (fractional anisotropy) skeleton (from dMRI c
2022	IDP dMRI TBSS OD Uncinate fasciculus L	Mean OD (orientation dispersion index) in uncinate fasciculus (left) on FA (fractional anisotropy) skeleton (from dMRI da
2023	IDP dMRI TBSS OD Tapetum R	Mean OD (orientation dispersion index) in tapetum (right) on FA (fractional anisotropy) skeleton (from dMRI data)
2024	IDP dMRI TBSS OD Tapetum L	Mean OD (orientation dispersion index) in tapetum (left) on FA (fractional anisotropy) skeleton (from dMRI data)
2025	IDP dMRI ProbtrackX OD ar l	Weighted-mean OD (orientation dispersion index) in tract left acoustic radiation (from dMRI data)
2026	IDP dMRI ProbtrackX OD ar r	Weighted-mean OD (orientation dispersion index) in tract right acoustic radiation (from dMRI data)
2027	IDP dMRI ProbtrackX OD atr l	Weighted-mean OD (orientation dispersion index) in tract left anterior thalamic radiation (from dMRI data)
2028	IDP dMRI ProbtrackX OD atr r	Weighted-mean OD (orientation dispersion index) in tract right anterior thalamic radiation (from dMRI data)
2029	IDP dMRI ProbtrackX OD cgc l	Weighted-mean OD (orientation dispersion index) in tract left cingulate gyrus part of cingulum (from dMRI data)
2030	IDP dMRI ProbtrackX OD cgc r	Weighted-mean OD (orientation dispersion index) in tract right cingulate gyrus part of cingulum (from dMRI data)
2031	IDP dMRI ProbtrackX OD cgh l	Weighted-mean OD (orientation dispersion index) in tract left parahippocampal part of cingulum (from dMRI data)
2032	IDP dMRI ProbtrackX OD cgh r	Weighted-mean OD (orientation dispersion index) in tract right parahippocampal part of cingulum (from dMRI data)
2033	IDP dMRI ProbtrackX OD cst l	Weighted-mean OD (orientation dispersion index) in tract left corticospinal tract (from dMRI data)
2034	IDP dMRI ProbtrackX OD cst r	Weighted-mean OD (orientation dispersion index) in tract right corticospinal tract (from dMRI data)
2035	IDP dMRI ProbtrackX OD fma	Weighted-mean OD (orientation dispersion index) in tract forceps major (from dMRI data)
2036	IDP dMRI ProbtrackX OD fmi	Weighted-mean OD (orientation dispersion index) in tract forceps minor (from dMRI data)
2037	IDP dMRI ProbtrackX OD ifo l	Weighted-mean OD (orientation dispersion index) in tract left inferior fronto-occipital fasciculus (from dMRI data)
2038	IDP dMRI ProbtrackX OD ifo r	Weighted-mean OD (orientation dispersion index) in tract right inferior fronto-occipital fasciculus (from dMRI data)
2039	IDP dMRI ProbtrackX OD ilf l	Weighted-mean OD (orientation dispersion index) in tract left inferior longitudinal fasciculus (from dMRI data)
2040	IDP dMRI ProbtrackX OD ilf r	Weighted-mean OD (orientation dispersion index) in tract right inferior longitudinal fasciculus (from dMRI data)
2041	IDP dMRI ProbtrackX OD mcp	Weighted-mean OD (orientation dispersion index) in tract middle cerebellar peduncle (from dMRI data)
2042	IDP dMRI ProbtrackX OD ml l	Weighted-mean OD (orientation dispersion index) in tract left medial lemniscus (from dMRI data)
2043	IDP dMRI ProbtrackX OD ml r	Weighted-mean OD (orientation dispersion index) in tract right medial lemniscus (from dMRI data)
2044	IDP dMRI ProbtrackX OD ptr l	Weighted-mean OD (orientation dispersion index) in tract left posterior thalamic radiation (from dMRI data)
2045	IDP dMRI ProbtrackX OD ptr r	Weighted-mean OD (orientation dispersion index) in tract right posterior thalamic radiation (from dMRI data)
2046	IDP dMRI ProbtrackX OD slf l	Weighted-mean OD (orientation dispersion index) in tract left superior longitudinal fasciculus (from dMRI data)
2047	IDP dMRI ProbtrackX OD slf r	Weighted-mean OD (orientation dispersion index) in tract right superior longitudinal fasciculus (from dMRI data)
2048	IDP dMRI ProbtrackX OD str l	Weighted-mean OD (orientation dispersion index) in tract left superior thalamic radiation (from dMRI data)
2049	IDP dMRI ProbtrackX OD str r	Weighted-mean OD (orientation dispersion index) in tract right superior thalamic radiation (from dMRI data)
2050	IDP dMRI ProbtrackX OD unc l	Weighted-mean OD (orientation dispersion index) in tract left uncinate fasciculus (from dMRI data)

IDP number	IDP short name	IDP description
2051	IDP dMRI ProbtrackX OD unc r	Weighted-mean OD (orientation dispersion index) in tract right uncinate fasciculus (from dMRI data)
2052	IDP dMRI TBSS ISOVF Middle cerebellar peduncle	Mean ISOVF (isotropic or free water volume fraction) in middle cerebellar peduncle on FA (fractional anisotropy) skeleto
2053	IDP dMRI TBSS ISOVF Pontine crossing tract	Mean ISOVF (isotropic or free water volume fraction) in pontine crossing tract on FA (fractional anisotropy) skeleton (fro
2054	IDP dMRI TBSS ISOVF Genu of corpus callosum	Mean ISOVF (isotropic or free water volume fraction) in genu of corpus callosum on FA (fractional anisotropy) skeleton
2055	IDP dMRI TBSS ISOVF Body of corpus callosum	Mean ISOVF (isotropic or free water volume fraction) in body of corpus callosum on FA (fractional anisotropy) skeleton
2056	IDP dMRI TBSS ISOVF Splenium of corpus callosum	Mean ISOVF (isotropic or free water volume fraction) in splenium of corpus callosum on FA (fractional anisotropy) skele
2057	IDP dMRI TBSS ISOVF Fornix	Mean ISOVF (isotropic or free water volume fraction) in fornix on FA (fractional anisotropy) skeleton (from dMRI data)
2058	IDP dMRI TBSS ISOVF Corticospinal tract R	Mean ISOVF (isotropic or free water volume fraction) in corticospinal tract (right) on FA (fractional anisotropy) skeleton (
2059	IDP dMRI TBSS ISOVF Corticospinal tract L	Mean ISOVF (isotropic or free water volume fraction) in corticospinal tract (left) on FA (fractional anisotropy) skeleton (fr
2060	IDP dMRI TBSS ISOVF Medial lemniscus R	Mean ISOVF (isotropic or free water volume fraction) in medial lemniscus (right) on FA (fractional anisotropy) skeleton (
2061	IDP dMRI TBSS ISOVF Medial lemniscus L	Mean ISOVF (isotropic or free water volume fraction) in medial lemniscus (left) on FA (fractional anisotropy) skeleton (fr
2062	IDP dMRI TBSS ISOVF Inferior cerebellar peduncle R	Mean ISOVF (isotropic or free water volume fraction) in inferior cerebellar peduncle (right) on FA (fractional anisotropy)
2063	IDP dMRI TBSS ISOVF Inferior cerebellar peduncle L	Mean ISOVF (isotropic or free water volume fraction) in inferior cerebellar peduncle (left) on FA (fractional anisotropy) s
2064	IDP dMRI TBSS ISOVF Superior cerebellar peduncle R	Mean ISOVF (isotropic or free water volume fraction) in superior cerebellar peduncle (right) on FA (fractional anisotropy
2065	IDP dMRI TBSS ISOVF Superior cerebellar peduncle L	Mean ISOVF (isotropic or free water volume fraction) in superior cerebellar peduncle (left) on FA (fractional anisotropy)
2066	IDP dMRI TBSS ISOVF Cerebral peduncle R	Mean ISOVF (isotropic or free water volume fraction) in cerebral peduncle (right) on FA (fractional anisotropy) skeleton
2067	IDP dMRI TBSS ISOVF Cerebral peduncle L	Mean ISOVF (isotropic or free water volume fraction) in cerebral peduncle (left) on FA (fractional anisotropy) skeleton (f
2068	IDP dMRI TBSS ISOVF Anterior limb of internal capsule R	Mean ISOVF (isotropic or free water volume fraction) in anterior limb of internal capsule (right) on FA (fractional anisotro
2069	IDP dMRI TBSS ISOVF Anterior limb of internal capsule L	Mean ISOVF (isotropic or free water volume fraction) in anterior limb of internal capsule (left) on FA (fractional anisotrop
2070	IDP dMRI TBSS ISOVF Posterior limb of internal capsule R	Mean ISOVF (isotropic or free water volume fraction) in posterior limb of internal capsule (right) on FA (fractional anisot
2071	IDP dMRI TBSS ISOVF Posterior limb of internal capsule L	Mean ISOVF (isotropic or free water volume fraction) in posterior limb of internal capsule (left) on FA (fractional anisotro
2072	IDP dMRI TBSS ISOVF Retrolenticular part of internal capsule R	Mean ISOVF (isotropic or free water volume fraction) in retrolenticular part of internal capsule (right) on FA (fractional ar
2073	IDP dMRI TBSS ISOVF Retrolenticular part of internal capsule L	Mean ISOVF (isotropic or free water volume fraction) in retrolenticular part of internal capsule (left) on FA (fractional ani
2074	IDP dMRI TBSS ISOVF Anterior corona radiata R	Mean ISOVF (isotropic or free water volume fraction) in anterior corona radiata (right) on FA (fractional anisotropy) skel
2075	IDP dMRI TBSS ISOVF Anterior corona radiata L	Mean ISOVF (isotropic or free water volume fraction) in anterior corona radiata (left) on FA (fractional anisotropy) skelet
2076	IDP dMRI TBSS ISOVF Superior corona radiata R	Mean ISOVF (isotropic or free water volume fraction) in superior corona radiata (right) on FA (fractional anisotropy) skel
2077	IDP dMRI TBSS ISOVF Superior corona radiata L	Mean ISOVF (isotropic or free water volume fraction) in superior corona radiata (left) on FA (fractional anisotropy) skele
2078	IDP dMRI TBSS ISOVF Posterior corona radiata R	Mean ISOVF (isotropic or free water volume fraction) in posterior corona radiata (right) on FA (fractional anisotropy) ske
2079	IDP dMRI TBSS ISOVF Posterior corona radiata L	Mean ISOVF (isotropic or free water volume fraction) in posterior corona radiata (left) on FA (fractional anisotropy) skele
2080	IDP dMRI TBSS ISOVF Posterior thalamic radiation R	Mean ISOVF (isotropic or free water volume fraction) in posterior thalamic radiation (right) on FA (fractional anisotropy)
2081	IDP dMRI TBSS ISOVF Posterior thalamic radiation L	Mean ISOVF (isotropic or free water volume fraction) in posterior thalamic radiation (left) on FA (fractional anisotropy) s
2082	IDP dMRI TBSS ISOVF Sagittal stratum R	Mean ISOVF (isotropic or free water volume fraction) in sagittal stratum (right) on FA (fractional anisotropy) skeleton (fro
2083	IDP dMRI TBSS ISOVF Sagittal stratum L	Mean ISOVF (isotropic or free water volume fraction) in sagittal stratum (left) on FA (fractional anisotropy) skeleton (fror
2084	IDP dMRI TBSS ISOVF External capsule R	Mean ISOVF (isotropic or free water volume fraction) in external capsule (right) on FA (fractional anisotropy) skeleton (f
2085	IDP dMRI TBSS ISOVF External capsule L	Mean ISOVF (isotropic or free water volume fraction) in external capsule (left) on FA (fractional anisotropy) skeleton (fro
2086	IDP dMRI TBSS ISOVF Cingulum cingulate gyrus R	Mean ISOVF (isotropic or free water volume fraction) in cingulum cingulate gyrus (right) on FA (fractional anisotropy) sk
2087	IDP dMRI TBSS ISOVF Cingulum cingulate gyrus L	Mean ISOVF (isotropic or free water volume fraction) in cingulum cingulate gyrus (left) on FA (fractional anisotropy) ske
2088	IDP dMRI TBSS ISOVF Cingulum hippocampus R	Mean ISOVF (isotropic or free water volume fraction) in cingulum hippocampus (right) on FA (fractional anisotropy) skel
2089	IDP dMRI TBSS ISOVF Cingulum hippocampus L	Mean ISOVF (isotropic or free water volume fraction) in cingulum hippocampus (left) on FA (fractional anisotropy) skele
2090	IDP dMRI TBSS ISOVF Fornix cres+Stria terminalis R	Mean ISOVF (isotropic or free water volume fraction) in fornix cres+stria terminalis (right) on FA (fractional anisotropy) s
2091	IDP dMRI TBSS ISOVF Fornix cres+Stria terminalis L	Mean ISOVF (isotropic or free water volume fraction) in fornix cres+stria terminalis (left) on FA (fractional anisotropy) sk
2092	IDP dMRI TBSS ISOVF Superior longitudinal fasciculus R	Mean ISOVF (isotropic or free water volume fraction) in superior longitudinal fasciculus (right) on FA (fractional anisotro
2093	IDP dMRI TBSS ISOVF Superior longitudinal fasciculus L	Mean ISOVF (isotropic or free water volume fraction) in superior longitudinal fasciculus (left) on FA (fractional anisotrop
2094	IDP dMRI TBSS ISOVF Superior fronto-occipital fasciculus R	Mean ISOVF (isotropic or free water volume fraction) in superior fronto-occipital fasciculus (right) on FA (fractional aniso
2095	IDP dMRI TBSS ISOVF Superior fronto-occipital fasciculus L	Mean ISOVF (isotropic or free water volume fraction) in superior fronto-occipital fasciculus (left) on FA (fractional anisot
2096	IDP dMRI TBSS ISOVF Uncinate fasciculus R	Mean ISOVF (isotropic or free water volume fraction) in uncinate fasciculus (right) on FA (fractional anisotropy) skeletor
2097	IDP dMRI TBSS ISOVF Uncinate fasciculus L	Mean ISOVF (isotropic or free water volume fraction) in uncinate fasciculus (left) on FA (fractional anisotropy) skeleton (
2098	IDP dMRI TBSS ISOVF Tapetum R	Mean ISOVF (isotropic or free water volume fraction) in tapetum (right) on FA (fractional anisotropy) skeleton (from dMF
2099	IDP dMRI TBSS ISOVF Tapetum L	Mean ISOVF (isotropic or free water volume fraction) in tapetum (left) on FA (fractional anisotropy) skeleton (from dMRI
2100	IDP dMRI ProbtrackX ISOVF ar l	Weighted-mean ISOVF (isotropic or free water volume fraction) in tract left acoustic radiation (from dMRI data)
2101	IDP dMRI ProbtrackX ISOVF ar r	Weighted-mean ISOVF (isotropic or free water volume fraction) in tract right acoustic radiation (from dMRI data)
2102	IDP dMRI ProbtrackX ISOVF atr l	Weighted-mean ISOVF (isotropic or free water volume fraction) in tract left anterior thalamic radiation (from dMRI data)
2103	IDP dMRI ProbtrackX ISOVF atr r	Weighted-mean ISOVF (isotropic or free water volume fraction) in tract right anterior thalamic radiation (from dMRI data
2104	IDP dMRI ProbtrackX ISOVF cgc l	Weighted-mean ISOVF (isotropic or free water volume fraction) in tract left cingulate gyrus part of cingulum (from dMRI
2105	IDP dMRI ProbtrackX ISOVF cgc r	Weighted-mean ISOVF (isotropic or free water volume fraction) in tract right cingulate gyrus part of cingulum (from dMR
2106	IDP dMRI ProbtrackX ISOVF cgh l	Weighted-mean ISOVF (isotropic or free water volume fraction) in tract left parahippocampal part of cingulum (from dMF
2107	IDP dMRI ProbtrackX ISOVF cgh r	Weighted-mean ISOVF (isotropic or free water volume fraction) in tract right parahippocampal part of cingulum (from dN

IDP number	IDP short name	IDP description
2108	IDP dMRI ProbtrackX ISOVF cst l	Weighted-mean ISOVF (isotropic or free water volume fraction) in tract left corticospinal tract (from dMRI data)
2109	IDP dMRI ProbtrackX ISOVF cst r	Weighted-mean ISOVF (isotropic or free water volume fraction) in tract right corticospinal tract (from dMRI data)
2110	IDP dMRI ProbtrackX ISOVF fma	Weighted-mean ISOVF (isotropic or free water volume fraction) in tract forceps major (from dMRI data)
2111	IDP dMRI ProbtrackX ISOVF fmi	Weighted-mean ISOVF (isotropic or free water volume fraction) in tract forceps minor (from dMRI data)
2112	IDP dMRI ProbtrackX ISOVF ifo l	Weighted-mean ISOVF (isotropic or free water volume fraction) in tract left inferior fronto-occipital fasciculus (from dMR
2113	IDP dMRI ProbtrackX ISOVF ifo r	Weighted-mean ISOVF (isotropic or free water volume fraction) in tract right inferior fronto-occipital fasciculus (from dMI
2114	IDP dMRI ProbtrackX ISOVF ilf l	Weighted-mean ISOVF (isotropic or free water volume fraction) in tract left inferior longitudinal fasciculus (from dMRI da
2115	IDP dMRI ProbtrackX ISOVF ilf r	Weighted-mean ISOVF (isotropic or free water volume fraction) in tract right inferior longitudinal fasciculus (from dMRI c
2116	IDP dMRI ProbtrackX ISOVF mcp	Weighted-mean ISOVF (isotropic or free water volume fraction) in tract middle cerebellar peduncle (from dMRI data)
2117	IDP dMRI ProbtrackX ISOVF ml l	Weighted-mean ISOVF (isotropic or free water volume fraction) in tract left medial lemniscus (from dMRI data)
2118	IDP dMRI ProbtrackX ISOVF ml r	Weighted-mean ISOVF (isotropic or free water volume fraction) in tract right medial lemniscus (from dMRI data)
2119	IDP dMRI ProbtrackX ISOVF ptr l	Weighted-mean ISOVF (isotropic or free water volume fraction) in tract left posterior thalamic radiation (from dMRI data)
2120	IDP dMRI ProbtrackX ISOVF ptr r	Weighted-mean ISOVF (isotropic or free water volume fraction) in tract right posterior thalamic radiation (from dMRI dat
2121	IDP dMRI ProbtrackX ISOVF slf l	Weighted-mean ISOVF (isotropic or free water volume fraction) in tract left superior longitudinal fasciculus (from dMRI d
2122	IDP dMRI ProbtrackX ISOVF slf r	Weighted-mean ISOVF (isotropic or free water volume fraction) in tract right superior longitudinal fasciculus (from dMRI
2123	IDP dMRI ProbtrackX ISOVF str l	Weighted-mean ISOVF (isotropic or free water volume fraction) in tract left superior thalamic radiation (from dMRI data)
2124	IDP dMRI ProbtrackX ISOVF str r	Weighted-mean ISOVF (isotropic or free water volume fraction) in tract right superior thalamic radiation (from dMRI data
2125	IDP dMRI ProbtrackX ISOVF unc l	Weighted-mean ISOVF (isotropic or free water volume fraction) in tract left uncinate fasciculus (from dMRI data)
2126	IDP dMRI ProbtrackX ISOVF unc r	Weighted-mean ISOVF (isotropic or free water volume fraction) in tract right uncinate fasciculus (from dMRI data)
2127	IDP tfMRI median BOLD shapes	Median BOLD (blood-oxygen-level dependent) effect (in group-defined mask) for shapes activation (in task fMRI data)
2128	IDP tfMRI 90th-percentile BOLD shapes	90th percentile of the BOLD (blood-oxygen-level dependent) effect (in group-defined mask) for shapes activation (in tas
2129	IDP tfMRI median zstat shapes	Median z-statistic (in group-defined mask) for shapes activation (in task fMRI data)
2130	IDP tfMRI 90th-percentile zstat shapes	90th percentile of the z-statistic (in group-defined mask) for shapes activation (in task fMRI data)
2131	IDP tfMRI median BOLD faces	Median BOLD (blood-oxygen-level dependent) effect (in group-defined mask) for faces activation (in task fMRI data)
2132	IDP tfMRI 90th-percentile BOLD faces	90th percentile of the BOLD (blood-oxygen-level dependent) effect (in group-defined mask) for faces activation (in task
2133	IDP tfMRI median zstat faces	Median z-statistic (in group-defined mask) for faces activation (in task fMRI data)
2134	IDP tfMRI 90th-percentile zstat faces	90th percentile of the z-statistic (in group-defined mask) for faces activation (in task fMRI data)
2135	IDP tfMRI median BOLD faces-shapes	Median BOLD (blood-oxygen-level dependent) effect (in group-defined mask) for faces-shapes contrast (in task fMRI da
2136	IDP tfMRI 90th-percentile BOLD faces-shapes	90th percentile of the BOLD (blood-oxygen-level dependent) effect (in group-defined mask) for faces-shapes contrast (i
2137	IDP tfMRI median zstat faces-shapes	Median z-statistic (in group-defined mask) for faces-shapes contrast (in task fMRI data)
2138	IDP tfMRI 90th-percentile zstat faces-shapes	90th percentile of the z-statistic (in group-defined mask) for faces-shapes contrast (in task fMRI data)
2139	IDP tfMRI median BOLD faces-shapes amygdala	Median BOLD (blood-oxygen-level dependent) effect (in group-defined amygdala activation mask) for faces-shapes cor
2140	IDP tfMRI 90th-percentile BOLD faces-shapes amygdala	90th percentile of the BOLD (blood-oxygen-level dependent) effect (in group-defined amygdala activation mask) for face
2141	IDP tfMRI median zstat faces-shapes amygdala	Median z-statistic (in group-defined amygdala activation mask) for faces-shapes contrast (in task fMRI data)
2142	IDP tfMRI 90th-percentile zstat faces-shapes amygdala	90th percentile of the z-statistic (in group-defined amygdala activation mask) for faces-shapes contrast (in task fMRI dat
2143	rfMRI amplitudes (ICA25 node 1)	rfMRI amplitudes (ICA25 node 1)
2144	rfMRI amplitudes (ICA25 node 2)	rfMRI amplitudes (ICA25 node 2)
2145	rfMRI amplitudes (ICA25 node 3)	rfMRI amplitudes (ICA25 node 3)
2146	rfMRI amplitudes (ICA25 node 4)	rfMRI amplitudes (ICA25 node 4)
2147	rfMRI amplitudes (ICA25 node 5)	rfMRI amplitudes (ICA25 node 5)
2148	rfMRI amplitudes (ICA25 node 6)	rfMRI amplitudes (ICA25 node 6)
2149	rfMRI amplitudes (ICA25 node 7)	rfMRI amplitudes (ICA25 node 7)
2150	rfMRI amplitudes (ICA25 node 8)	rfMRI amplitudes (ICA25 node 8)
2151	rfMRI amplitudes (ICA25 node 9)	rfMRI amplitudes (ICA25 node 9)
2152	rfMRI amplitudes (ICA25 node 10)	rfMRI amplitudes (ICA25 node 10)
2153	rfMRI amplitudes (ICA25 node 11)	rfMRI amplitudes (ICA25 node 11)
2154	rfMRI amplitudes (ICA25 node 12)	rfMRI amplitudes (ICA25 node 12)
2155	rfMRI amplitudes (ICA25 node 13)	rfMRI amplitudes (ICA25 node 13)
2156	rfMRI amplitudes (ICA25 node 14)	rfMRI amplitudes (ICA25 node 14)
2157	rfMRI amplitudes (ICA25 node 15)	rfMRI amplitudes (ICA25 node 15)
2158	rfMRI amplitudes (ICA25 node 16)	rfMRI amplitudes (ICA25 node 16)
2159	rfMRI amplitudes (ICA25 node 17)	rfMRI amplitudes (ICA25 node 17)
2160	rfMRI amplitudes (ICA25 node 18)	rfMRI amplitudes (ICA25 node 18)
2161	rfMRI amplitudes (ICA25 node 19)	rfMRI amplitudes (ICA25 node 19)
2162	rfMRI amplitudes (ICA25 node 20)	rfMRI amplitudes (ICA25 node 20)
2163	rfMRI amplitudes (ICA25 node 21)	rfMRI amplitudes (ICA25 node 21)
2164	rfMRI amplitudes (ICA100 node 1)	rfMRI amplitudes (ICA100 node 1)

IDP number	IDP short name	IDP description
2165	rfMRI amplitudes (ICA100 node 2)	rfMRI amplitudes (ICA100 node 2)
2166	rfMRI amplitudes (ICA100 node 3)	rfMRI amplitudes (ICA100 node 3)
2167	rfMRI amplitudes (ICA100 node 4)	rfMRI amplitudes (ICA100 node 4)
2168	rfMRI amplitudes (ICA100 node 5)	rfMRI amplitudes (ICA100 node 5)
2169	rfMRI amplitudes (ICA100 node 6)	rfMRI amplitudes (ICA100 node 6)
2170	rfMRI amplitudes (ICA100 node 7)	rfMRI amplitudes (ICA100 node 7)
2171	rfMRI amplitudes (ICA100 node 8)	rfMRI amplitudes (ICA100 node 8)
2172	rfMRI amplitudes (ICA100 node 9)	rfMRI amplitudes (ICA100 node 9)
2173	rfMRI amplitudes (ICA100 node 10)	rfMRI amplitudes (ICA100 node 10)
2174	rfMRI amplitudes (ICA100 node 11)	rfMRI amplitudes (ICA100 node 11)
2175	rfMRI amplitudes (ICA100 node 12)	rfMRI amplitudes (ICA100 node 12)
2176	rfMRI amplitudes (ICA100 node 13)	rfMRI amplitudes (ICA100 node 13)
2177	rfMRI amplitudes (ICA100 node 14)	rfMRI amplitudes (ICA100 node 14)
2178	rfMRI amplitudes (ICA100 node 15)	rfMRI amplitudes (ICA100 node 15)
2179	rfMRI amplitudes (ICA100 node 16)	rfMRI amplitudes (ICA100 node 16)
2180	rfMRI amplitudes (ICA100 node 17)	rfMRI amplitudes (ICA100 node 17)
2181	rfMRI amplitudes (ICA100 node 18)	rfMRI amplitudes (ICA100 node 18)
2182	rfMRI amplitudes (ICA100 node 19)	rfMRI amplitudes (ICA100 node 19)
2183	rfMRI amplitudes (ICA100 node 20)	rfMRI amplitudes (ICA100 node 20)
2184	rfMRI amplitudes (ICA100 node 21)	rfMRI amplitudes (ICA100 node 21)
2185	rfMRI amplitudes (ICA100 node 22)	rfMRI amplitudes (ICA100 node 22)
2186	rfMRI amplitudes (ICA100 node 23)	rfMRI amplitudes (ICA100 node 23)
2187	rfMRI amplitudes (ICA100 node 24)	rfMRI amplitudes (ICA100 node 24)
2188	rfMRI amplitudes (ICA100 node 25)	rfMRI amplitudes (ICA100 node 25)
2189	rfMRI amplitudes (ICA100 node 26)	rfMRI amplitudes (ICA100 node 26)
2190	rfMRI amplitudes (ICA100 node 27)	rfMRI amplitudes (ICA100 node 27)
2191	rfMRI amplitudes (ICA100 node 28)	rfMRI amplitudes (ICA100 node 28)
2192	rfMRI amplitudes (ICA100 node 29)	rfMRI amplitudes (ICA100 node 29)
2193	rfMRI amplitudes (ICA100 node 30)	rfMRI amplitudes (ICA100 node 30)
2194	rfMRI amplitudes (ICA100 node 31)	rfMRI amplitudes (ICA100 node 31)
2195	rfMRI amplitudes (ICA100 node 32)	rfMRI amplitudes (ICA100 node 32)
2196	rfMRI amplitudes (ICA100 node 33)	rfMRI amplitudes (ICA100 node 33)
2197	rfMRI amplitudes (ICA100 node 34)	rfMRI amplitudes (ICA100 node 34)
2198	rfMRI amplitudes (ICA100 node 35)	rfMRI amplitudes (ICA100 node 35)
2199	rfMRI amplitudes (ICA100 node 36)	rfMRI amplitudes (ICA100 node 36)
2200	rfMRI amplitudes (ICA100 node 37)	rfMRI amplitudes (ICA100 node 37)
2201	rfMRI amplitudes (ICA100 node 38)	rfMRI amplitudes (ICA100 node 38)
2202	rfMRI amplitudes (ICA100 node 39)	rfMRI amplitudes (ICA100 node 39)
2203	rfMRI amplitudes (ICA100 node 40)	rfMRI amplitudes (ICA100 node 40)
2204	rfMRI amplitudes (ICA100 node 41)	rfMRI amplitudes (ICA100 node 41)
2205	rfMRI amplitudes (ICA100 node 42)	rfMRI amplitudes (ICA100 node 42)
2206	rfMRI amplitudes (ICA100 node 43)	rfMRI amplitudes (ICA100 node 43)
2207	rfMRI amplitudes (ICA100 node 44)	rfMRI amplitudes (ICA100 node 44)
2208	rfMRI amplitudes (ICA100 node 45)	rfMRI amplitudes (ICA100 node 45)
2209	rfMRI amplitudes (ICA100 node 46)	rfMRI amplitudes (ICA100 node 46)
2210	rfMRI amplitudes (ICA100 node 47)	rfMRI amplitudes (ICA100 node 47)
2211	rfMRI amplitudes (ICA100 node 48)	rfMRI amplitudes (ICA100 node 48)
2212	rfMRI amplitudes (ICA100 node 49)	rfMRI amplitudes (ICA100 node 49)
2213	rfMRI amplitudes (ICA100 node 50)	rfMRI amplitudes (ICA100 node 50)
2214	rfMRI amplitudes (ICA100 node 51)	rfMRI amplitudes (ICA100 node 51)
2215	rfMRI amplitudes (ICA100 node 52)	rfMRI amplitudes (ICA100 node 52)
2216	rfMRI amplitudes (ICA100 node 53)	rfMRI amplitudes (ICA100 node 53)
2217	rfMRI amplitudes (ICA100 node 54)	rfMRI amplitudes (ICA100 node 54)
2218	rfMRI amplitudes (ICA100 node 55)	rfMRI amplitudes (ICA100 node 55)
2219	rfMRI connectivity (ICA25 edge 1)	rfMRI connectivity (ICA25 edge 1)
2220	rfMRI connectivity (ICA25 edge 2)	rfMRI connectivity (ICA25 edge 2)
2221	rfMRI connectivity (ICA25 edge 3)	rfMRI connectivity (ICA25 edge 3)

IDP number	IDP short name	IDP description
2222	rfMRI connectivity (ICA25 edge 4)	rfMRI connectivity (ICA25 edge 4)
2223	rfMRI connectivity (ICA25 edge 5)	rfMRI connectivity (ICA25 edge 5)
2224	rfMRI connectivity (ICA25 edge 6)	rfMRI connectivity (ICA25 edge 6)
2225	rfMRI connectivity (ICA25 edge 7)	rfMRI connectivity (ICA25 edge 7)
2226	rfMRI connectivity (ICA25 edge 8)	rfMRI connectivity (ICA25 edge 8)
2227	rfMRI connectivity (ICA25 edge 9)	rfMRI connectivity (ICA25 edge 9)
2228	rfMRI connectivity (ICA25 edge 10)	rfMRI connectivity (ICA25 edge 10)
2229	rfMRI connectivity (ICA25 edge 11)	rfMRI connectivity (ICA25 edge 11)
2230	rfMRI connectivity (ICA25 edge 12)	rfMRI connectivity (ICA25 edge 12)
2231	rfMRI connectivity (ICA25 edge 13)	rfMRI connectivity (ICA25 edge 13)
2232	rfMRI connectivity (ICA25 edge 14)	rfMRI connectivity (ICA25 edge 14)
2233	rfMRI connectivity (ICA25 edge 15)	rfMRI connectivity (ICA25 edge 15)
2234	rfMRI connectivity (ICA25 edge 16)	rfMRI connectivity (ICA25 edge 16)
2235	rfMRI connectivity (ICA25 edge 17)	rfMRI connectivity (ICA25 edge 17)
2236	rfMRI connectivity (ICA25 edge 18)	rfMRI connectivity (ICA25 edge 18)
2237	rfMRI connectivity (ICA25 edge 19)	rfMRI connectivity (ICA25 edge 19)
2238	rfMRI connectivity (ICA25 edge 20)	rfMRI connectivity (ICA25 edge 20)
2239	rfMRI connectivity (ICA25 edge 21)	rfMRI connectivity (ICA25 edge 21)
2240	rfMRI connectivity (ICA25 edge 22)	rfMRI connectivity (ICA25 edge 22)
2241	rfMRI connectivity (ICA25 edge 23)	rfMRI connectivity (ICA25 edge 23)
2242	rfMRI connectivity (ICA25 edge 24)	rfMRI connectivity (ICA25 edge 24)
2243	rfMRI connectivity (ICA25 edge 25)	rfMRI connectivity (ICA25 edge 25)
2244	rfMRI connectivity (ICA25 edge 26)	rfMRI connectivity (ICA25 edge 26)
2245	rfMRI connectivity (ICA25 edge 27)	rfMRI connectivity (ICA25 edge 27)
2246	rfMRI connectivity (ICA25 edge 28)	rfMRI connectivity (ICA25 edge 28)
2247	rfMRI connectivity (ICA25 edge 29)	rfMRI connectivity (ICA25 edge 29)
2248	rfMRI connectivity (ICA25 edge 30)	rfMRI connectivity (ICA25 edge 30)
2249	rfMRI connectivity (ICA25 edge 31)	rfMRI connectivity (ICA25 edge 31)
2250	rfMRI connectivity (ICA25 edge 32)	rfMRI connectivity (ICA25 edge 32)
2251	rfMRI connectivity (ICA25 edge 33)	rfMRI connectivity (ICA25 edge 33)
2252	rfMRI connectivity (ICA25 edge 34)	rfMRI connectivity (ICA25 edge 34)
2253	rfMRI connectivity (ICA25 edge 35)	rfMRI connectivity (ICA25 edge 35)
2254	rfMRI connectivity (ICA25 edge 36)	rfMRI connectivity (ICA25 edge 36)
2255	rfMRI connectivity (ICA25 edge 37)	rfMRI connectivity (ICA25 edge 37)
2256	rfMRI connectivity (ICA25 edge 38)	rfMRI connectivity (ICA25 edge 38)
2257	rfMRI connectivity (ICA25 edge 39)	rfMRI connectivity (ICA25 edge 39)
2258	rfMRI connectivity (ICA25 edge 40)	rfMRI connectivity (ICA25 edge 40)
2259	rfMRI connectivity (ICA25 edge 41)	rfMRI connectivity (ICA25 edge 41)
2260	rfMRI connectivity (ICA25 edge 42)	rfMRI connectivity (ICA25 edge 42)
2261	rfMRI connectivity (ICA25 edge 43)	rfMRI connectivity (ICA25 edge 43)
2262	rfMRI connectivity (ICA25 edge 44)	rfMRI connectivity (ICA25 edge 44)
2263	rfMRI connectivity (ICA25 edge 45)	rfMRI connectivity (ICA25 edge 45)
2264	rfMRI connectivity (ICA25 edge 46)	rfMRI connectivity (ICA25 edge 46)
2265	rfMRI connectivity (ICA25 edge 47)	rfMRI connectivity (ICA25 edge 47)
2266	rfMRI connectivity (ICA25 edge 48)	rfMRI connectivity (ICA25 edge 48)
2267	rfMRI connectivity (ICA25 edge 49)	rfMRI connectivity (ICA25 edge 49)
2268	rfMRI connectivity (ICA25 edge 50)	rfMRI connectivity (ICA25 edge 50)
2269	rfMRI connectivity (ICA25 edge 51)	rfMRI connectivity (ICA25 edge 51)
2270	rfMRI connectivity (ICA25 edge 52)	rfMRI connectivity (ICA25 edge 52)
2271	rfMRI connectivity (ICA25 edge 53)	rfMRI connectivity (ICA25 edge 53)
2272	rfMRI connectivity (ICA25 edge 54)	rfMRI connectivity (ICA25 edge 54)
2273	rfMRI connectivity (ICA25 edge 55)	rfMRI connectivity (ICA25 edge 55)
2274	rfMRI connectivity (ICA25 edge 56)	rfMRI connectivity (ICA25 edge 56)
2275	rfMRI connectivity (ICA25 edge 57)	rfMRI connectivity (ICA25 edge 57)
2276	rfMRI connectivity (ICA25 edge 58)	rfMRI connectivity (ICA25 edge 58)
2277	rfMRI connectivity (ICA25 edge 59)	rfMRI connectivity (ICA25 edge 59)
2278	rfMRI connectivity (ICA25 edge 60)	rfMRI connectivity (ICA25 edge 60)

IDP number	IDP short name	IDP description
2279	rfMRI connectivity (ICA25 edge 61)	rfMRI connectivity (ICA25 edge 61)
2280	rfMRI connectivity (ICA25 edge 62)	rfMRI connectivity (ICA25 edge 62)
2281	rfMRI connectivity (ICA25 edge 63)	rfMRI connectivity (ICA25 edge 63)
2282	rfMRI connectivity (ICA25 edge 64)	rfMRI connectivity (ICA25 edge 64)
2283	rfMRI connectivity (ICA25 edge 65)	rfMRI connectivity (ICA25 edge 65)
2284	rfMRI connectivity (ICA25 edge 66)	rfMRI connectivity (ICA25 edge 66)
2285	rfMRI connectivity (ICA25 edge 67)	rfMRI connectivity (ICA25 edge 67)
2286	rfMRI connectivity (ICA25 edge 68)	rfMRI connectivity (ICA25 edge 68)
2287	rfMRI connectivity (ICA25 edge 69)	rfMRI connectivity (ICA25 edge 69)
2288	rfMRI connectivity (ICA25 edge 70)	rfMRI connectivity (ICA25 edge 70)
2289	rfMRI connectivity (ICA25 edge 71)	rfMRI connectivity (ICA25 edge 71)
2290	rfMRI connectivity (ICA25 edge 72)	rfMRI connectivity (ICA25 edge 72)
2291	rfMRI connectivity (ICA25 edge 73)	rfMRI connectivity (ICA25 edge 73)
2292	rfMRI connectivity (ICA25 edge 74)	rfMRI connectivity (ICA25 edge 74)
2293	rfMRI connectivity (ICA25 edge 75)	rfMRI connectivity (ICA25 edge 75)
2294	rfMRI connectivity (ICA25 edge 76)	rfMRI connectivity (ICA25 edge 76)
2295	rfMRI connectivity (ICA25 edge 77)	rfMRI connectivity (ICA25 edge 77)
2296	rfMRI connectivity (ICA25 edge 78)	rfMRI connectivity (ICA25 edge 78)
2297	rfMRI connectivity (ICA25 edge 79)	rfMRI connectivity (ICA25 edge 79)
2298	rfMRI connectivity (ICA25 edge 80)	rfMRI connectivity (ICA25 edge 80)
2299	rfMRI connectivity (ICA25 edge 81)	rfMRI connectivity (ICA25 edge 81)
2300	rfMRI connectivity (ICA25 edge 82)	rfMRI connectivity (ICA25 edge 82)
2301	rfMRI connectivity (ICA25 edge 83)	rfMRI connectivity (ICA25 edge 83)
2302	rfMRI connectivity (ICA25 edge 84)	rfMRI connectivity (ICA25 edge 84)
2303	rfMRI connectivity (ICA25 edge 85)	rfMRI connectivity (ICA25 edge 85)
2304	rfMRI connectivity (ICA25 edge 86)	rfMRI connectivity (ICA25 edge 86)
2305	rfMRI connectivity (ICA25 edge 87)	rfMRI connectivity (ICA25 edge 87)
2306	rfMRI connectivity (ICA25 edge 88)	rfMRI connectivity (ICA25 edge 88)
2307	rfMRI connectivity (ICA25 edge 89)	rfMRI connectivity (ICA25 edge 89)
2308	rfMRI connectivity (ICA25 edge 90)	rfMRI connectivity (ICA25 edge 90)
2309	rfMRI connectivity (ICA25 edge 91)	rfMRI connectivity (ICA25 edge 91)
2310	rfMRI connectivity (ICA25 edge 92)	rfMRI connectivity (ICA25 edge 92)
2311	rfMRI connectivity (ICA25 edge 93)	rfMRI connectivity (ICA25 edge 93)
2312	rfMRI connectivity (ICA25 edge 94)	rfMRI connectivity (ICA25 edge 94)
2313	rfMRI connectivity (ICA25 edge 95)	rfMRI connectivity (ICA25 edge 95)
2314	rfMRI connectivity (ICA25 edge 96)	rfMRI connectivity (ICA25 edge 96)
2315	rfMRI connectivity (ICA25 edge 97)	rfMRI connectivity (ICA25 edge 97)
2316	rfMRI connectivity (ICA25 edge 98)	rfMRI connectivity (ICA25 edge 98)
2317	rfMRI connectivity (ICA25 edge 99)	rfMRI connectivity (ICA25 edge 99)
2318	rfMRI connectivity (ICA25 edge 100)	rfMRI connectivity (ICA25 edge 100)
2319	rfMRI connectivity (ICA25 edge 101)	rfMRI connectivity (ICA25 edge 101)
2320	rfMRI connectivity (ICA25 edge 102)	rfMRI connectivity (ICA25 edge 102)
2321	rfMRI connectivity (ICA25 edge 103)	rfMRI connectivity (ICA25 edge 103)
2322	rfMRI connectivity (ICA25 edge 104)	rfMRI connectivity (ICA25 edge 104)
2323	rfMRI connectivity (ICA25 edge 105)	rfMRI connectivity (ICA25 edge 105)
2324	rfMRI connectivity (ICA25 edge 106)	rfMRI connectivity (ICA25 edge 106)
2325	rfMRI connectivity (ICA25 edge 107)	rfMRI connectivity (ICA25 edge 107)
2326	rfMRI connectivity (ICA25 edge 108)	rfMRI connectivity (ICA25 edge 108)
2327	rfMRI connectivity (ICA25 edge 109)	rfMRI connectivity (ICA25 edge 109)
2328	rfMRI connectivity (ICA25 edge 110)	rfMRI connectivity (ICA25 edge 110)
2329	rfMRI connectivity (ICA25 edge 111)	rfMRI connectivity (ICA25 edge 111)
2330	rfMRI connectivity (ICA25 edge 112)	rfMRI connectivity (ICA25 edge 112)
2331	rfMRI connectivity (ICA25 edge 113)	rfMRI connectivity (ICA25 edge 113)
2332	rfMRI connectivity (ICA25 edge 114)	rfMRI connectivity (ICA25 edge 114)
2333	rfMRI connectivity (ICA25 edge 115)	rfMRI connectivity (ICA25 edge 115)
2334	rfMRI connectivity (ICA25 edge 116)	rfMRI connectivity (ICA25 edge 116)
2335	rfMRI connectivity (ICA25 edge 117)	rfMRI connectivity (ICA25 edge 117)

IDP number	IDP short name	IDP description
2336	rfMRI connectivity (ICA25 edge 118)	rfMRI connectivity (ICA25 edge 118)
2337	rfMRI connectivity (ICA25 edge 119)	rfMRI connectivity (ICA25 edge 119)
2338	rfMRI connectivity (ICA25 edge 120)	rfMRI connectivity (ICA25 edge 120)
2339	rfMRI connectivity (ICA25 edge 121)	rfMRI connectivity (ICA25 edge 121)
2340	rfMRI connectivity (ICA25 edge 122)	rfMRI connectivity (ICA25 edge 122)
2341	rfMRI connectivity (ICA25 edge 123)	rfMRI connectivity (ICA25 edge 123)
2342	rfMRI connectivity (ICA25 edge 124)	rfMRI connectivity (ICA25 edge 124)
2343	rfMRI connectivity (ICA25 edge 125)	rfMRI connectivity (ICA25 edge 125)
2344	rfMRI connectivity (ICA25 edge 126)	rfMRI connectivity (ICA25 edge 126)
2345	rfMRI connectivity (ICA25 edge 127)	rfMRI connectivity (ICA25 edge 127)
2346	rfMRI connectivity (ICA25 edge 128)	rfMRI connectivity (ICA25 edge 128)
2347	rfMRI connectivity (ICA25 edge 129)	rfMRI connectivity (ICA25 edge 129)
2348	rfMRI connectivity (ICA25 edge 130)	rfMRI connectivity (ICA25 edge 130)
2349	rfMRI connectivity (ICA25 edge 131)	rfMRI connectivity (ICA25 edge 131)
2350	rfMRI connectivity (ICA25 edge 132)	rfMRI connectivity (ICA25 edge 132)
2351	rfMRI connectivity (ICA25 edge 133)	rfMRI connectivity (ICA25 edge 133)
2352	rfMRI connectivity (ICA25 edge 134)	rfMRI connectivity (ICA25 edge 134)
2353	rfMRI connectivity (ICA25 edge 135)	rfMRI connectivity (ICA25 edge 135)
2354	rfMRI connectivity (ICA25 edge 136)	rfMRI connectivity (ICA25 edge 136)
2355	rfMRI connectivity (ICA25 edge 137)	rfMRI connectivity (ICA25 edge 137)
2356	rfMRI connectivity (ICA25 edge 138)	rfMRI connectivity (ICA25 edge 138)
2357	rfMRI connectivity (ICA25 edge 139)	rfMRI connectivity (ICA25 edge 139)
2358	rfMRI connectivity (ICA25 edge 140)	rfMRI connectivity (ICA25 edge 140)
2359	rfMRI connectivity (ICA25 edge 141)	rfMRI connectivity (ICA25 edge 141)
2360	rfMRI connectivity (ICA25 edge 142)	rfMRI connectivity (ICA25 edge 142)
2361	rfMRI connectivity (ICA25 edge 143)	rfMRI connectivity (ICA25 edge 143)
2362	rfMRI connectivity (ICA25 edge 144)	rfMRI connectivity (ICA25 edge 144)
2363	rfMRI connectivity (ICA25 edge 145)	rfMRI connectivity (ICA25 edge 145)
2364	rfMRI connectivity (ICA25 edge 146)	rfMRI connectivity (ICA25 edge 146)
2365	rfMRI connectivity (ICA25 edge 147)	rfMRI connectivity (ICA25 edge 147)
2366	rfMRI connectivity (ICA25 edge 148)	rfMRI connectivity (ICA25 edge 148)
2367	rfMRI connectivity (ICA25 edge 149)	rfMRI connectivity (ICA25 edge 149)
2368	rfMRI connectivity (ICA25 edge 150)	rfMRI connectivity (ICA25 edge 150)
2369	rfMRI connectivity (ICA25 edge 151)	rfMRI connectivity (ICA25 edge 151)
2370	rfMRI connectivity (ICA25 edge 152)	rfMRI connectivity (ICA25 edge 152)
2371	rfMRI connectivity (ICA25 edge 153)	rfMRI connectivity (ICA25 edge 153)
2372	rfMRI connectivity (ICA25 edge 154)	rfMRI connectivity (ICA25 edge 154)
2373	rfMRI connectivity (ICA25 edge 155)	rfMRI connectivity (ICA25 edge 155)
2374	rfMRI connectivity (ICA25 edge 156)	rfMRI connectivity (ICA25 edge 156)
2375	rfMRI connectivity (ICA25 edge 157)	rfMRI connectivity (ICA25 edge 157)
2376	rfMRI connectivity (ICA25 edge 158)	rfMRI connectivity (ICA25 edge 158)
2377	rfMRI connectivity (ICA25 edge 159)	rfMRI connectivity (ICA25 edge 159)
2378	rfMRI connectivity (ICA25 edge 160)	rfMRI connectivity (ICA25 edge 160)
2379	rfMRI connectivity (ICA25 edge 161)	rfMRI connectivity (ICA25 edge 161)
2380	rfMRI connectivity (ICA25 edge 162)	rfMRI connectivity (ICA25 edge 162)
2381	rfMRI connectivity (ICA25 edge 163)	rfMRI connectivity (ICA25 edge 163)
2382	rfMRI connectivity (ICA25 edge 164)	rfMRI connectivity (ICA25 edge 164)
2383	rfMRI connectivity (ICA25 edge 165)	rfMRI connectivity (ICA25 edge 165)
2384	rfMRI connectivity (ICA25 edge 166)	rfMRI connectivity (ICA25 edge 166)
2385	rfMRI connectivity (ICA25 edge 167)	rfMRI connectivity (ICA25 edge 167)
2386	rfMRI connectivity (ICA25 edge 168)	rfMRI connectivity (ICA25 edge 168)
2387	rfMRI connectivity (ICA25 edge 169)	rfMRI connectivity (ICA25 edge 169)
2388	rfMRI connectivity (ICA25 edge 170)	rfMRI connectivity (ICA25 edge 170)
2389	rfMRI connectivity (ICA25 edge 171)	rfMRI connectivity (ICA25 edge 171)
2390	rfMRI connectivity (ICA25 edge 172)	rfMRI connectivity (ICA25 edge 172)
2391	rfMRI connectivity (ICA25 edge 173)	rfMRI connectivity (ICA25 edge 173)
2392	rfMRI connectivity (ICA25 edge 174)	rfMRI connectivity (ICA25 edge 174)

IDP number	IDP short name	IDP description
2393	rfMRI connectivity (ICA25 edge 175)	rfMRI connectivity (ICA25 edge 175)
2394	rfMRI connectivity (ICA25 edge 176)	rfMRI connectivity (ICA25 edge 176)
2395	rfMRI connectivity (ICA25 edge 177)	rfMRI connectivity (ICA25 edge 177)
2396	rfMRI connectivity (ICA25 edge 178)	rfMRI connectivity (ICA25 edge 178)
2397	rfMRI connectivity (ICA25 edge 179)	rfMRI connectivity (ICA25 edge 179)
2398	rfMRI connectivity (ICA25 edge 180)	rfMRI connectivity (ICA25 edge 180)
2399	rfMRI connectivity (ICA25 edge 181)	rfMRI connectivity (ICA25 edge 181)
2400	rfMRI connectivity (ICA25 edge 182)	rfMRI connectivity (ICA25 edge 182)
2401	rfMRI connectivity (ICA25 edge 183)	rfMRI connectivity (ICA25 edge 183)
2402	rfMRI connectivity (ICA25 edge 184)	rfMRI connectivity (ICA25 edge 184)
2403	rfMRI connectivity (ICA25 edge 185)	rfMRI connectivity (ICA25 edge 185)
2404	rfMRI connectivity (ICA25 edge 186)	rfMRI connectivity (ICA25 edge 186)
2405	rfMRI connectivity (ICA25 edge 187)	rfMRI connectivity (ICA25 edge 187)
2406	rfMRI connectivity (ICA25 edge 188)	rfMRI connectivity (ICA25 edge 188)
2407	rfMRI connectivity (ICA25 edge 189)	rfMRI connectivity (ICA25 edge 189)
2408	rfMRI connectivity (ICA25 edge 190)	rfMRI connectivity (ICA25 edge 190)
2409	rfMRI connectivity (ICA25 edge 191)	rfMRI connectivity (ICA25 edge 191)
2410	rfMRI connectivity (ICA25 edge 192)	rfMRI connectivity (ICA25 edge 192)
2411	rfMRI connectivity (ICA25 edge 193)	rfMRI connectivity (ICA25 edge 193)
2412	rfMRI connectivity (ICA25 edge 194)	rfMRI connectivity (ICA25 edge 194)
2413	rfMRI connectivity (ICA25 edge 195)	rfMRI connectivity (ICA25 edge 195)
2414	rfMRI connectivity (ICA25 edge 196)	rfMRI connectivity (ICA25 edge 196)
2415	rfMRI connectivity (ICA25 edge 197)	rfMRI connectivity (ICA25 edge 197)
2416	rfMRI connectivity (ICA25 edge 198)	rfMRI connectivity (ICA25 edge 198)
2417	rfMRI connectivity (ICA25 edge 199)	rfMRI connectivity (ICA25 edge 199)
2418	rfMRI connectivity (ICA25 edge 200)	rfMRI connectivity (ICA25 edge 200)
2419	rfMRI connectivity (ICA25 edge 201)	rfMRI connectivity (ICA25 edge 201)
2420	rfMRI connectivity (ICA25 edge 202)	rfMRI connectivity (ICA25 edge 202)
2421	rfMRI connectivity (ICA25 edge 203)	rfMRI connectivity (ICA25 edge 203)
2422	rfMRI connectivity (ICA25 edge 204)	rfMRI connectivity (ICA25 edge 204)
2423	rfMRI connectivity (ICA25 edge 205)	rfMRI connectivity (ICA25 edge 205)
2424	rfMRI connectivity (ICA25 edge 206)	rfMRI connectivity (ICA25 edge 206)
2425	rfMRI connectivity (ICA25 edge 207)	rfMRI connectivity (ICA25 edge 207)
2426	rfMRI connectivity (ICA25 edge 208)	rfMRI connectivity (ICA25 edge 208)
2427	rfMRI connectivity (ICA25 edge 209)	rfMRI connectivity (ICA25 edge 209)
2428	rfMRI connectivity (ICA25 edge 210)	rfMRI connectivity (ICA25 edge 210)
2429	rfMRI connectivity (ICA100 edge 1)	rfMRI connectivity (ICA100 edge 1)
2430	rfMRI connectivity (ICA100 edge 2)	rfMRI connectivity (ICA100 edge 2)
2431	rfMRI connectivity (ICA100 edge 3)	rfMRI connectivity (ICA100 edge 3)
2432	rfMRI connectivity (ICA100 edge 4)	rfMRI connectivity (ICA100 edge 4)
2433	rfMRI connectivity (ICA100 edge 5)	rfMRI connectivity (ICA100 edge 5)
2434	rfMRI connectivity (ICA100 edge 6)	rfMRI connectivity (ICA100 edge 6)
2435	rfMRI connectivity (ICA100 edge 7)	rfMRI connectivity (ICA100 edge 7)
2436	rfMRI connectivity (ICA100 edge 8)	rfMRI connectivity (ICA100 edge 8)
2437	rfMRI connectivity (ICA100 edge 9)	rfMRI connectivity (ICA100 edge 9)
2438	rfMRI connectivity (ICA100 edge 10)	rfMRI connectivity (ICA100 edge 10)
2439	rfMRI connectivity (ICA100 edge 11)	rfMRI connectivity (ICA100 edge 11)
2440	rfMRI connectivity (ICA100 edge 12)	rfMRI connectivity (ICA100 edge 12)
2441	rfMRI connectivity (ICA100 edge 13)	rfMRI connectivity (ICA100 edge 13)
2442	rfMRI connectivity (ICA100 edge 14)	rfMRI connectivity (ICA100 edge 14)
2443	rfMRI connectivity (ICA100 edge 15)	rfMRI connectivity (ICA100 edge 15)
2444	rfMRI connectivity (ICA100 edge 16)	rfMRI connectivity (ICA100 edge 16)
2445	rfMRI connectivity (ICA100 edge 17)	rfMRI connectivity (ICA100 edge 17)
2446	rfMRI connectivity (ICA100 edge 18)	rfMRI connectivity (ICA100 edge 18)
2447	rfMRI connectivity (ICA100 edge 19)	rfMRI connectivity (ICA100 edge 19)
2448	rfMRI connectivity (ICA100 edge 20)	rfMRI connectivity (ICA100 edge 20)
2449	rfMRI connectivity (ICA100 edge 21)	rfMRI connectivity (ICA100 edge 21)

IDP number	IDP short name	IDP description
2450	rfMRI connectivity (ICA100 edge 22)	rfMRI connectivity (ICA100 edge 22)
2451	rfMRI connectivity (ICA100 edge 23)	rfMRI connectivity (ICA100 edge 23)
2452	rfMRI connectivity (ICA100 edge 24)	rfMRI connectivity (ICA100 edge 24)
2453	rfMRI connectivity (ICA100 edge 25)	rfMRI connectivity (ICA100 edge 25)
2454	rfMRI connectivity (ICA100 edge 26)	rfMRI connectivity (ICA100 edge 26)
2455	rfMRI connectivity (ICA100 edge 27)	rfMRI connectivity (ICA100 edge 27)
2456	rfMRI connectivity (ICA100 edge 28)	rfMRI connectivity (ICA100 edge 28)
2457	rfMRI connectivity (ICA100 edge 29)	rfMRI connectivity (ICA100 edge 29)
2458	rfMRI connectivity (ICA100 edge 30)	rfMRI connectivity (ICA100 edge 30)
2459	rfMRI connectivity (ICA100 edge 31)	rfMRI connectivity (ICA100 edge 31)
2460	rfMRI connectivity (ICA100 edge 32)	rfMRI connectivity (ICA100 edge 32)
2461	rfMRI connectivity (ICA100 edge 33)	rfMRI connectivity (ICA100 edge 33)
2462	rfMRI connectivity (ICA100 edge 34)	rfMRI connectivity (ICA100 edge 34)
2463	rfMRI connectivity (ICA100 edge 35)	rfMRI connectivity (ICA100 edge 35)
2464	rfMRI connectivity (ICA100 edge 36)	rfMRI connectivity (ICA100 edge 36)
2465	rfMRI connectivity (ICA100 edge 37)	rfMRI connectivity (ICA100 edge 37)
2466	rfMRI connectivity (ICA100 edge 38)	rfMRI connectivity (ICA100 edge 38)
2467	rfMRI connectivity (ICA100 edge 39)	rfMRI connectivity (ICA100 edge 39)
2468	rfMRI connectivity (ICA100 edge 40)	rfMRI connectivity (ICA100 edge 40)
2469	rfMRI connectivity (ICA100 edge 41)	rfMRI connectivity (ICA100 edge 41)
2470	rfMRI connectivity (ICA100 edge 42)	rfMRI connectivity (ICA100 edge 42)
2471	rfMRI connectivity (ICA100 edge 43)	rfMRI connectivity (ICA100 edge 43)
2472	rfMRI connectivity (ICA100 edge 44)	rfMRI connectivity (ICA100 edge 44)
2473	rfMRI connectivity (ICA100 edge 45)	rfMRI connectivity (ICA100 edge 45)
2474	rfMRI connectivity (ICA100 edge 46)	rfMRI connectivity (ICA100 edge 46)
2475	rfMRI connectivity (ICA100 edge 47)	rfMRI connectivity (ICA100 edge 47)
2476	rfMRI connectivity (ICA100 edge 48)	rfMRI connectivity (ICA100 edge 48)
2477	rfMRI connectivity (ICA100 edge 49)	rfMRI connectivity (ICA100 edge 49)
2478	rfMRI connectivity (ICA100 edge 50)	rfMRI connectivity (ICA100 edge 50)
2479	rfMRI connectivity (ICA100 edge 51)	rfMRI connectivity (ICA100 edge 51)
2480	rfMRI connectivity (ICA100 edge 52)	rfMRI connectivity (ICA100 edge 52)
2481	rfMRI connectivity (ICA100 edge 53)	rfMRI connectivity (ICA100 edge 53)
2482	rfMRI connectivity (ICA100 edge 54)	rfMRI connectivity (ICA100 edge 54)
2483	rfMRI connectivity (ICA100 edge 55)	rfMRI connectivity (ICA100 edge 55)
2484	rfMRI connectivity (ICA100 edge 56)	rfMRI connectivity (ICA100 edge 56)
2485	rfMRI connectivity (ICA100 edge 57)	rfMRI connectivity (ICA100 edge 57)
2486	rfMRI connectivity (ICA100 edge 58)	rfMRI connectivity (ICA100 edge 58)
2487	rfMRI connectivity (ICA100 edge 59)	rfMRI connectivity (ICA100 edge 59)
2488	rfMRI connectivity (ICA100 edge 60)	rfMRI connectivity (ICA100 edge 60)
2489	rfMRI connectivity (ICA100 edge 61)	rfMRI connectivity (ICA100 edge 61)
2490	rfMRI connectivity (ICA100 edge 62)	rfMRI connectivity (ICA100 edge 62)
2491	rfMRI connectivity (ICA100 edge 63)	rfMRI connectivity (ICA100 edge 63)
2492	rfMRI connectivity (ICA100 edge 64)	rfMRI connectivity (ICA100 edge 64)
2493	rfMRI connectivity (ICA100 edge 65)	rfMRI connectivity (ICA100 edge 65)
2494	rfMRI connectivity (ICA100 edge 66)	rfMRI connectivity (ICA100 edge 66)
2495	rfMRI connectivity (ICA100 edge 67)	rfMRI connectivity (ICA100 edge 67)
2496	rfMRI connectivity (ICA100 edge 68)	rfMRI connectivity (ICA100 edge 68)
2497	rfMRI connectivity (ICA100 edge 69)	rfMRI connectivity (ICA100 edge 69)
2498	rfMRI connectivity (ICA100 edge 70)	rfMRI connectivity (ICA100 edge 70)
2499	rfMRI connectivity (ICA100 edge 71)	rfMRI connectivity (ICA100 edge 71)
2500	rfMRI connectivity (ICA100 edge 72)	rfMRI connectivity (ICA100 edge 72)
2501	rfMRI connectivity (ICA100 edge 73)	rfMRI connectivity (ICA100 edge 73)
2502	rfMRI connectivity (ICA100 edge 74)	rfMRI connectivity (ICA100 edge 74)
2503	rfMRI connectivity (ICA100 edge 75)	rfMRI connectivity (ICA100 edge 75)
2504	rfMRI connectivity (ICA100 edge 76)	rfMRI connectivity (ICA100 edge 76)
2505	rfMRI connectivity (ICA100 edge 77)	rfMRI connectivity (ICA100 edge 77)
2506	rfMRI connectivity (ICA100 edge 78)	rfMRI connectivity (ICA100 edge 78)

IDP number	IDP short name	IDP description
2507	rfMRI connectivity (ICA100 edge 79)	rfMRI connectivity (ICA100 edge 79)
2508	rfMRI connectivity (ICA100 edge 80)	rfMRI connectivity (ICA100 edge 80)
2509	rfMRI connectivity (ICA100 edge 81)	rfMRI connectivity (ICA100 edge 81)
2510	rfMRI connectivity (ICA100 edge 82)	rfMRI connectivity (ICA100 edge 82)
2511	rfMRI connectivity (ICA100 edge 83)	rfMRI connectivity (ICA100 edge 83)
2512	rfMRI connectivity (ICA100 edge 84)	rfMRI connectivity (ICA100 edge 84)
2513	rfMRI connectivity (ICA100 edge 85)	rfMRI connectivity (ICA100 edge 85)
2514	rfMRI connectivity (ICA100 edge 86)	rfMRI connectivity (ICA100 edge 86)
2515	rfMRI connectivity (ICA100 edge 87)	rfMRI connectivity (ICA100 edge 87)
2516	rfMRI connectivity (ICA100 edge 88)	rfMRI connectivity (ICA100 edge 88)
2517	rfMRI connectivity (ICA100 edge 89)	rfMRI connectivity (ICA100 edge 89)
2518	rfMRI connectivity (ICA100 edge 90)	rfMRI connectivity (ICA100 edge 90)
2519	rfMRI connectivity (ICA100 edge 91)	rfMRI connectivity (ICA100 edge 91)
2520	rfMRI connectivity (ICA100 edge 92)	rfMRI connectivity (ICA100 edge 92)
2521	rfMRI connectivity (ICA100 edge 93)	rfMRI connectivity (ICA100 edge 93)
2522	rfMRI connectivity (ICA100 edge 94)	rfMRI connectivity (ICA100 edge 94)
2523	rfMRI connectivity (ICA100 edge 95)	rfMRI connectivity (ICA100 edge 95)
2524	rfMRI connectivity (ICA100 edge 96)	rfMRI connectivity (ICA100 edge 96)
2525	rfMRI connectivity (ICA100 edge 97)	rfMRI connectivity (ICA100 edge 97)
2526	rfMRI connectivity (ICA100 edge 98)	rfMRI connectivity (ICA100 edge 98)
2527	rfMRI connectivity (ICA100 edge 99)	rfMRI connectivity (ICA100 edge 99)
2528	rfMRI connectivity (ICA100 edge 100)	rfMRI connectivity (ICA100 edge 100)
2529	rfMRI connectivity (ICA100 edge 101)	rfMRI connectivity (ICA100 edge 101)
2530	rfMRI connectivity (ICA100 edge 102)	rfMRI connectivity (ICA100 edge 102)
2531	rfMRI connectivity (ICA100 edge 103)	rfMRI connectivity (ICA100 edge 103)
2532	rfMRI connectivity (ICA100 edge 104)	rfMRI connectivity (ICA100 edge 104)
2533	rfMRI connectivity (ICA100 edge 105)	rfMRI connectivity (ICA100 edge 105)
2534	rfMRI connectivity (ICA100 edge 106)	rfMRI connectivity (ICA100 edge 106)
2535	rfMRI connectivity (ICA100 edge 107)	rfMRI connectivity (ICA100 edge 107)
2536	rfMRI connectivity (ICA100 edge 108)	rfMRI connectivity (ICA100 edge 108)
2537	rfMRI connectivity (ICA100 edge 109)	rfMRI connectivity (ICA100 edge 109)
2538	rfMRI connectivity (ICA100 edge 110)	rfMRI connectivity (ICA100 edge 110)
2539	rfMRI connectivity (ICA100 edge 111)	rfMRI connectivity (ICA100 edge 111)
2540	rfMRI connectivity (ICA100 edge 112)	rfMRI connectivity (ICA100 edge 112)
2541	rfMRI connectivity (ICA100 edge 113)	rfMRI connectivity (ICA100 edge 113)
2542	rfMRI connectivity (ICA100 edge 114)	rfMRI connectivity (ICA100 edge 114)
2543	rfMRI connectivity (ICA100 edge 115)	rfMRI connectivity (ICA100 edge 115)
2544	rfMRI connectivity (ICA100 edge 116)	rfMRI connectivity (ICA100 edge 116)
2545	rfMRI connectivity (ICA100 edge 117)	rfMRI connectivity (ICA100 edge 117)
2546	rfMRI connectivity (ICA100 edge 118)	rfMRI connectivity (ICA100 edge 118)
2547	rfMRI connectivity (ICA100 edge 119)	rfMRI connectivity (ICA100 edge 119)
2548	rfMRI connectivity (ICA100 edge 120)	rfMRI connectivity (ICA100 edge 120)
2549	rfMRI connectivity (ICA100 edge 121)	rfMRI connectivity (ICA100 edge 121)
2550	rfMRI connectivity (ICA100 edge 122)	rfMRI connectivity (ICA100 edge 122)
2551	rfMRI connectivity (ICA100 edge 123)	rfMRI connectivity (ICA100 edge 123)
2552	rfMRI connectivity (ICA100 edge 124)	rfMRI connectivity (ICA100 edge 124)
2553	rfMRI connectivity (ICA100 edge 125)	rfMRI connectivity (ICA100 edge 125)
2554	rfMRI connectivity (ICA100 edge 126)	rfMRI connectivity (ICA100 edge 126)
2555	rfMRI connectivity (ICA100 edge 127)	rfMRI connectivity (ICA100 edge 127)
2556	rfMRI connectivity (ICA100 edge 128)	rfMRI connectivity (ICA100 edge 128)
2557	rfMRI connectivity (ICA100 edge 129)	rfMRI connectivity (ICA100 edge 129)
2558	rfMRI connectivity (ICA100 edge 130)	rfMRI connectivity (ICA100 edge 130)
2559	rfMRI connectivity (ICA100 edge 131)	rfMRI connectivity (ICA100 edge 131)
2560	rfMRI connectivity (ICA100 edge 132)	rfMRI connectivity (ICA100 edge 132)
2561	rfMRI connectivity (ICA100 edge 133)	rfMRI connectivity (ICA100 edge 133)
2562	rfMRI connectivity (ICA100 edge 134)	rfMRI connectivity (ICA100 edge 134)
2563	rfMRI connectivity (ICA100 edge 135)	rfMRI connectivity (ICA100 edge 135)

IDP number	IDP short name	IDP description
2564	rfMRI connectivity (ICA100 edge 136)	rfMRI connectivity (ICA100 edge 136)
2565	rfMRI connectivity (ICA100 edge 137)	rfMRI connectivity (ICA100 edge 137)
2566	rfMRI connectivity (ICA100 edge 138)	rfMRI connectivity (ICA100 edge 138)
2567	rfMRI connectivity (ICA100 edge 139)	rfMRI connectivity (ICA100 edge 139)
2568	rfMRI connectivity (ICA100 edge 140)	rfMRI connectivity (ICA100 edge 140)
2569	rfMRI connectivity (ICA100 edge 141)	rfMRI connectivity (ICA100 edge 141)
2570	rfMRI connectivity (ICA100 edge 142)	rfMRI connectivity (ICA100 edge 142)
2571	rfMRI connectivity (ICA100 edge 143)	rfMRI connectivity (ICA100 edge 143)
2572	rfMRI connectivity (ICA100 edge 144)	rfMRI connectivity (ICA100 edge 144)
2573	rfMRI connectivity (ICA100 edge 145)	rfMRI connectivity (ICA100 edge 145)
2574	rfMRI connectivity (ICA100 edge 146)	rfMRI connectivity (ICA100 edge 146)
2575	rfMRI connectivity (ICA100 edge 147)	rfMRI connectivity (ICA100 edge 147)
2576	rfMRI connectivity (ICA100 edge 148)	rfMRI connectivity (ICA100 edge 148)
2577	rfMRI connectivity (ICA100 edge 149)	rfMRI connectivity (ICA100 edge 149)
2578	rfMRI connectivity (ICA100 edge 150)	rfMRI connectivity (ICA100 edge 150)
2579	rfMRI connectivity (ICA100 edge 151)	rfMRI connectivity (ICA100 edge 151)
2580	rfMRI connectivity (ICA100 edge 152)	rfMRI connectivity (ICA100 edge 152)
2581	rfMRI connectivity (ICA100 edge 153)	rfMRI connectivity (ICA100 edge 153)
2582	rfMRI connectivity (ICA100 edge 154)	rfMRI connectivity (ICA100 edge 154)
2583	rfMRI connectivity (ICA100 edge 155)	rfMRI connectivity (ICA100 edge 155)
2584	rfMRI connectivity (ICA100 edge 156)	rfMRI connectivity (ICA100 edge 156)
2585	rfMRI connectivity (ICA100 edge 157)	rfMRI connectivity (ICA100 edge 157)
2586	rfMRI connectivity (ICA100 edge 158)	rfMRI connectivity (ICA100 edge 158)
2587	rfMRI connectivity (ICA100 edge 159)	rfMRI connectivity (ICA100 edge 159)
2588	rfMRI connectivity (ICA100 edge 160)	rfMRI connectivity (ICA100 edge 160)
2589	rfMRI connectivity (ICA100 edge 161)	rfMRI connectivity (ICA100 edge 161)
2590	rfMRI connectivity (ICA100 edge 162)	rfMRI connectivity (ICA100 edge 162)
2591	rfMRI connectivity (ICA100 edge 163)	rfMRI connectivity (ICA100 edge 163)
2592	rfMRI connectivity (ICA100 edge 164)	rfMRI connectivity (ICA100 edge 164)
2593	rfMRI connectivity (ICA100 edge 165)	rfMRI connectivity (ICA100 edge 165)
2594	rfMRI connectivity (ICA100 edge 166)	rfMRI connectivity (ICA100 edge 166)
2595	rfMRI connectivity (ICA100 edge 167)	rfMRI connectivity (ICA100 edge 167)
2596	rfMRI connectivity (ICA100 edge 168)	rfMRI connectivity (ICA100 edge 168)
2597	rfMRI connectivity (ICA100 edge 169)	rfMRI connectivity (ICA100 edge 169)
2598	rfMRI connectivity (ICA100 edge 170)	rfMRI connectivity (ICA100 edge 170)
2599	rfMRI connectivity (ICA100 edge 171)	rfMRI connectivity (ICA100 edge 171)
2600	rfMRI connectivity (ICA100 edge 172)	rfMRI connectivity (ICA100 edge 172)
2601	rfMRI connectivity (ICA100 edge 173)	rfMRI connectivity (ICA100 edge 173)
2602	rfMRI connectivity (ICA100 edge 174)	rfMRI connectivity (ICA100 edge 174)
2603	rfMRI connectivity (ICA100 edge 175)	rfMRI connectivity (ICA100 edge 175)
2604	rfMRI connectivity (ICA100 edge 176)	rfMRI connectivity (ICA100 edge 176)
2605	rfMRI connectivity (ICA100 edge 177)	rfMRI connectivity (ICA100 edge 177)
2606	rfMRI connectivity (ICA100 edge 178)	rfMRI connectivity (ICA100 edge 178)
2607	rfMRI connectivity (ICA100 edge 179)	rfMRI connectivity (ICA100 edge 179)
2608	rfMRI connectivity (ICA100 edge 180)	rfMRI connectivity (ICA100 edge 180)
2609	rfMRI connectivity (ICA100 edge 181)	rfMRI connectivity (ICA100 edge 181)
2610	rfMRI connectivity (ICA100 edge 182)	rfMRI connectivity (ICA100 edge 182)
2611	rfMRI connectivity (ICA100 edge 183)	rfMRI connectivity (ICA100 edge 183)
2612	rfMRI connectivity (ICA100 edge 184)	rfMRI connectivity (ICA100 edge 184)
2613	rfMRI connectivity (ICA100 edge 185)	rfMRI connectivity (ICA100 edge 185)
2614	rfMRI connectivity (ICA100 edge 186)	rfMRI connectivity (ICA100 edge 186)
2615	rfMRI connectivity (ICA100 edge 187)	rfMRI connectivity (ICA100 edge 187)
2616	rfMRI connectivity (ICA100 edge 188)	rfMRI connectivity (ICA100 edge 188)
2617	rfMRI connectivity (ICA100 edge 189)	rfMRI connectivity (ICA100 edge 189)
2618	rfMRI connectivity (ICA100 edge 190)	rfMRI connectivity (ICA100 edge 190)
2619	rfMRI connectivity (ICA100 edge 191)	rfMRI connectivity (ICA100 edge 191)
2620	rfMRI connectivity (ICA100 edge 192)	rfMRI connectivity (ICA100 edge 192)

IDP number	IDP short name	IDP description
2621	rfMRI connectivity (ICA100 edge 193)	rfMRI connectivity (ICA100 edge 193)
2622	rfMRI connectivity (ICA100 edge 194)	rfMRI connectivity (ICA100 edge 194)
2623	rfMRI connectivity (ICA100 edge 195)	rfMRI connectivity (ICA100 edge 195)
2624	rfMRI connectivity (ICA100 edge 196)	rfMRI connectivity (ICA100 edge 196)
2625	rfMRI connectivity (ICA100 edge 197)	rfMRI connectivity (ICA100 edge 197)
2626	rfMRI connectivity (ICA100 edge 198)	rfMRI connectivity (ICA100 edge 198)
2627	rfMRI connectivity (ICA100 edge 199)	rfMRI connectivity (ICA100 edge 199)
2628	rfMRI connectivity (ICA100 edge 200)	rfMRI connectivity (ICA100 edge 200)
2629	rfMRI connectivity (ICA100 edge 201)	rfMRI connectivity (ICA100 edge 201)
2630	rfMRI connectivity (ICA100 edge 202)	rfMRI connectivity (ICA100 edge 202)
2631	rfMRI connectivity (ICA100 edge 203)	rfMRI connectivity (ICA100 edge 203)
2632	rfMRI connectivity (ICA100 edge 204)	rfMRI connectivity (ICA100 edge 204)
2633	rfMRI connectivity (ICA100 edge 205)	rfMRI connectivity (ICA100 edge 205)
2634	rfMRI connectivity (ICA100 edge 206)	rfMRI connectivity (ICA100 edge 206)
2635	rfMRI connectivity (ICA100 edge 207)	rfMRI connectivity (ICA100 edge 207)
2636	rfMRI connectivity (ICA100 edge 208)	rfMRI connectivity (ICA100 edge 208)
2637	rfMRI connectivity (ICA100 edge 209)	rfMRI connectivity (ICA100 edge 209)
2638	rfMRI connectivity (ICA100 edge 210)	rfMRI connectivity (ICA100 edge 210)
2639	rfMRI connectivity (ICA100 edge 211)	rfMRI connectivity (ICA100 edge 211)
2640	rfMRI connectivity (ICA100 edge 212)	rfMRI connectivity (ICA100 edge 212)
2641	rfMRI connectivity (ICA100 edge 213)	rfMRI connectivity (ICA100 edge 213)
2642	rfMRI connectivity (ICA100 edge 214)	rfMRI connectivity (ICA100 edge 214)
2643	rfMRI connectivity (ICA100 edge 215)	rfMRI connectivity (ICA100 edge 215)
2644	rfMRI connectivity (ICA100 edge 216)	rfMRI connectivity (ICA100 edge 216)
2645	rfMRI connectivity (ICA100 edge 217)	rfMRI connectivity (ICA100 edge 217)
2646	rfMRI connectivity (ICA100 edge 218)	rfMRI connectivity (ICA100 edge 218)
2647	rfMRI connectivity (ICA100 edge 219)	rfMRI connectivity (ICA100 edge 219)
2648	rfMRI connectivity (ICA100 edge 220)	rfMRI connectivity (ICA100 edge 220)
2649	rfMRI connectivity (ICA100 edge 221)	rfMRI connectivity (ICA100 edge 221)
2650	rfMRI connectivity (ICA100 edge 222)	rfMRI connectivity (ICA100 edge 222)
2651	rfMRI connectivity (ICA100 edge 223)	rfMRI connectivity (ICA100 edge 223)
2652	rfMRI connectivity (ICA100 edge 224)	rfMRI connectivity (ICA100 edge 224)
2653	rfMRI connectivity (ICA100 edge 225)	rfMRI connectivity (ICA100 edge 225)
2654	rfMRI connectivity (ICA100 edge 226)	rfMRI connectivity (ICA100 edge 226)
2655	rfMRI connectivity (ICA100 edge 227)	rfMRI connectivity (ICA100 edge 227)
2656	rfMRI connectivity (ICA100 edge 228)	rfMRI connectivity (ICA100 edge 228)
2657	rfMRI connectivity (ICA100 edge 229)	rfMRI connectivity (ICA100 edge 229)
2658	rfMRI connectivity (ICA100 edge 230)	rfMRI connectivity (ICA100 edge 230)
2659	rfMRI connectivity (ICA100 edge 231)	rfMRI connectivity (ICA100 edge 231)
2660	rfMRI connectivity (ICA100 edge 232)	rfMRI connectivity (ICA100 edge 232)
2661	rfMRI connectivity (ICA100 edge 233)	rfMRI connectivity (ICA100 edge 233)
2662	rfMRI connectivity (ICA100 edge 234)	rfMRI connectivity (ICA100 edge 234)
2663	rfMRI connectivity (ICA100 edge 235)	rfMRI connectivity (ICA100 edge 235)
2664	rfMRI connectivity (ICA100 edge 236)	rfMRI connectivity (ICA100 edge 236)
2665	rfMRI connectivity (ICA100 edge 237)	rfMRI connectivity (ICA100 edge 237)
2666	rfMRI connectivity (ICA100 edge 238)	rfMRI connectivity (ICA100 edge 238)
2667	rfMRI connectivity (ICA100 edge 239)	rfMRI connectivity (ICA100 edge 239)
2668	rfMRI connectivity (ICA100 edge 240)	rfMRI connectivity (ICA100 edge 240)
2669	rfMRI connectivity (ICA100 edge 241)	rfMRI connectivity (ICA100 edge 241)
2670	rfMRI connectivity (ICA100 edge 242)	rfMRI connectivity (ICA100 edge 242)
2671	rfMRI connectivity (ICA100 edge 243)	rfMRI connectivity (ICA100 edge 243)
2672	rfMRI connectivity (ICA100 edge 244)	rfMRI connectivity (ICA100 edge 244)
2673	rfMRI connectivity (ICA100 edge 245)	rfMRI connectivity (ICA100 edge 245)
2674	rfMRI connectivity (ICA100 edge 246)	rfMRI connectivity (ICA100 edge 246)
2675	rfMRI connectivity (ICA100 edge 247)	rfMRI connectivity (ICA100 edge 247)
2676	rfMRI connectivity (ICA100 edge 248)	rfMRI connectivity (ICA100 edge 248)
2677	rfMRI connectivity (ICA100 edge 249)	rfMRI connectivity (ICA100 edge 249)

IDP number	IDP short name	IDP description
2678	rfMRI connectivity (ICA100 edge 250)	rfMRI connectivity (ICA100 edge 250)
2679	rfMRI connectivity (ICA100 edge 251)	rfMRI connectivity (ICA100 edge 251)
2680	rfMRI connectivity (ICA100 edge 252)	rfMRI connectivity (ICA100 edge 252)
2681	rfMRI connectivity (ICA100 edge 253)	rfMRI connectivity (ICA100 edge 253)
2682	rfMRI connectivity (ICA100 edge 254)	rfMRI connectivity (ICA100 edge 254)
2683	rfMRI connectivity (ICA100 edge 255)	rfMRI connectivity (ICA100 edge 255)
2684	rfMRI connectivity (ICA100 edge 256)	rfMRI connectivity (ICA100 edge 256)
2685	rfMRI connectivity (ICA100 edge 257)	rfMRI connectivity (ICA100 edge 257)
2686	rfMRI connectivity (ICA100 edge 258)	rfMRI connectivity (ICA100 edge 258)
2687	rfMRI connectivity (ICA100 edge 259)	rfMRI connectivity (ICA100 edge 259)
2688	rfMRI connectivity (ICA100 edge 260)	rfMRI connectivity (ICA100 edge 260)
2689	rfMRI connectivity (ICA100 edge 261)	rfMRI connectivity (ICA100 edge 261)
2690	rfMRI connectivity (ICA100 edge 262)	rfMRI connectivity (ICA100 edge 262)
2691	rfMRI connectivity (ICA100 edge 263)	rfMRI connectivity (ICA100 edge 263)
2692	rfMRI connectivity (ICA100 edge 264)	rfMRI connectivity (ICA100 edge 264)
2693	rfMRI connectivity (ICA100 edge 265)	rfMRI connectivity (ICA100 edge 265)
2694	rfMRI connectivity (ICA100 edge 266)	rfMRI connectivity (ICA100 edge 266)
2695	rfMRI connectivity (ICA100 edge 267)	rfMRI connectivity (ICA100 edge 267)
2696	rfMRI connectivity (ICA100 edge 268)	rfMRI connectivity (ICA100 edge 268)
2697	rfMRI connectivity (ICA100 edge 269)	rfMRI connectivity (ICA100 edge 269)
2698	rfMRI connectivity (ICA100 edge 270)	rfMRI connectivity (ICA100 edge 270)
2699	rfMRI connectivity (ICA100 edge 271)	rfMRI connectivity (ICA100 edge 271)
2700	rfMRI connectivity (ICA100 edge 272)	rfMRI connectivity (ICA100 edge 272)
2701	rfMRI connectivity (ICA100 edge 273)	rfMRI connectivity (ICA100 edge 273)
2702	rfMRI connectivity (ICA100 edge 274)	rfMRI connectivity (ICA100 edge 274)
2703	rfMRI connectivity (ICA100 edge 275)	rfMRI connectivity (ICA100 edge 275)
2704	rfMRI connectivity (ICA100 edge 276)	rfMRI connectivity (ICA100 edge 276)
2705	rfMRI connectivity (ICA100 edge 277)	rfMRI connectivity (ICA100 edge 277)
2706	rfMRI connectivity (ICA100 edge 278)	rfMRI connectivity (ICA100 edge 278)
2707	rfMRI connectivity (ICA100 edge 279)	rfMRI connectivity (ICA100 edge 279)
2708	rfMRI connectivity (ICA100 edge 280)	rfMRI connectivity (ICA100 edge 280)
2709	rfMRI connectivity (ICA100 edge 281)	rfMRI connectivity (ICA100 edge 281)
2710	rfMRI connectivity (ICA100 edge 282)	rfMRI connectivity (ICA100 edge 282)
2711	rfMRI connectivity (ICA100 edge 283)	rfMRI connectivity (ICA100 edge 283)
2712	rfMRI connectivity (ICA100 edge 284)	rfMRI connectivity (ICA100 edge 284)
2713	rfMRI connectivity (ICA100 edge 285)	rfMRI connectivity (ICA100 edge 285)
2714	rfMRI connectivity (ICA100 edge 286)	rfMRI connectivity (ICA100 edge 286)
2715	rfMRI connectivity (ICA100 edge 287)	rfMRI connectivity (ICA100 edge 287)
2716	rfMRI connectivity (ICA100 edge 288)	rfMRI connectivity (ICA100 edge 288)
2717	rfMRI connectivity (ICA100 edge 289)	rfMRI connectivity (ICA100 edge 289)
2718	rfMRI connectivity (ICA100 edge 290)	rfMRI connectivity (ICA100 edge 290)
2719	rfMRI connectivity (ICA100 edge 291)	rfMRI connectivity (ICA100 edge 291)
2720	rfMRI connectivity (ICA100 edge 292)	rfMRI connectivity (ICA100 edge 292)
2721	rfMRI connectivity (ICA100 edge 293)	rfMRI connectivity (ICA100 edge 293)
2722	rfMRI connectivity (ICA100 edge 294)	rfMRI connectivity (ICA100 edge 294)
2723	rfMRI connectivity (ICA100 edge 295)	rfMRI connectivity (ICA100 edge 295)
2724	rfMRI connectivity (ICA100 edge 296)	rfMRI connectivity (ICA100 edge 296)
2725	rfMRI connectivity (ICA100 edge 297)	rfMRI connectivity (ICA100 edge 297)
2726	rfMRI connectivity (ICA100 edge 298)	rfMRI connectivity (ICA100 edge 298)
2727	rfMRI connectivity (ICA100 edge 299)	rfMRI connectivity (ICA100 edge 299)
2728	rfMRI connectivity (ICA100 edge 300)	rfMRI connectivity (ICA100 edge 300)
2729	rfMRI connectivity (ICA100 edge 301)	rfMRI connectivity (ICA100 edge 301)
2730	rfMRI connectivity (ICA100 edge 302)	rfMRI connectivity (ICA100 edge 302)
2731	rfMRI connectivity (ICA100 edge 303)	rfMRI connectivity (ICA100 edge 303)
2732	rfMRI connectivity (ICA100 edge 304)	rfMRI connectivity (ICA100 edge 304)
2733	rfMRI connectivity (ICA100 edge 305)	rfMRI connectivity (ICA100 edge 305)
2734	rfMRI connectivity (ICA100 edge 306)	rfMRI connectivity (ICA100 edge 306)

IDP number	IDP short name	IDP description
2735	rfMRI connectivity (ICA100 edge 307)	rfMRI connectivity (ICA100 edge 307)
2736	rfMRI connectivity (ICA100 edge 308)	rfMRI connectivity (ICA100 edge 308)
2737	rfMRI connectivity (ICA100 edge 309)	rfMRI connectivity (ICA100 edge 309)
2738	rfMRI connectivity (ICA100 edge 310)	rfMRI connectivity (ICA100 edge 310)
2739	rfMRI connectivity (ICA100 edge 311)	rfMRI connectivity (ICA100 edge 311)
2740	rfMRI connectivity (ICA100 edge 312)	rfMRI connectivity (ICA100 edge 312)
2741	rfMRI connectivity (ICA100 edge 313)	rfMRI connectivity (ICA100 edge 313)
2742	rfMRI connectivity (ICA100 edge 314)	rfMRI connectivity (ICA100 edge 314)
2743	rfMRI connectivity (ICA100 edge 315)	rfMRI connectivity (ICA100 edge 315)
2744	rfMRI connectivity (ICA100 edge 316)	rfMRI connectivity (ICA100 edge 316)
2745	rfMRI connectivity (ICA100 edge 317)	rfMRI connectivity (ICA100 edge 317)
2746	rfMRI connectivity (ICA100 edge 318)	rfMRI connectivity (ICA100 edge 318)
2747	rfMRI connectivity (ICA100 edge 319)	rfMRI connectivity (ICA100 edge 319)
2748	rfMRI connectivity (ICA100 edge 320)	rfMRI connectivity (ICA100 edge 320)
2749	rfMRI connectivity (ICA100 edge 321)	rfMRI connectivity (ICA100 edge 321)
2750	rfMRI connectivity (ICA100 edge 322)	rfMRI connectivity (ICA100 edge 322)
2751	rfMRI connectivity (ICA100 edge 323)	rfMRI connectivity (ICA100 edge 323)
2752	rfMRI connectivity (ICA100 edge 324)	rfMRI connectivity (ICA100 edge 324)
2753	rfMRI connectivity (ICA100 edge 325)	rfMRI connectivity (ICA100 edge 325)
2754	rfMRI connectivity (ICA100 edge 326)	rfMRI connectivity (ICA100 edge 326)
2755	rfMRI connectivity (ICA100 edge 327)	rfMRI connectivity (ICA100 edge 327)
2756	rfMRI connectivity (ICA100 edge 328)	rfMRI connectivity (ICA100 edge 328)
2757	rfMRI connectivity (ICA100 edge 329)	rfMRI connectivity (ICA100 edge 329)
2758	rfMRI connectivity (ICA100 edge 330)	rfMRI connectivity (ICA100 edge 330)
2759	rfMRI connectivity (ICA100 edge 331)	rfMRI connectivity (ICA100 edge 331)
2760	rfMRI connectivity (ICA100 edge 332)	rfMRI connectivity (ICA100 edge 332)
2761	rfMRI connectivity (ICA100 edge 333)	rfMRI connectivity (ICA100 edge 333)
2762	rfMRI connectivity (ICA100 edge 334)	rfMRI connectivity (ICA100 edge 334)
2763	rfMRI connectivity (ICA100 edge 335)	rfMRI connectivity (ICA100 edge 335)
2764	rfMRI connectivity (ICA100 edge 336)	rfMRI connectivity (ICA100 edge 336)
2765	rfMRI connectivity (ICA100 edge 337)	rfMRI connectivity (ICA100 edge 337)
2766	rfMRI connectivity (ICA100 edge 338)	rfMRI connectivity (ICA100 edge 338)
2767	rfMRI connectivity (ICA100 edge 339)	rfMRI connectivity (ICA100 edge 339)
2768	rfMRI connectivity (ICA100 edge 340)	rfMRI connectivity (ICA100 edge 340)
2769	rfMRI connectivity (ICA100 edge 341)	rfMRI connectivity (ICA100 edge 341)
2770	rfMRI connectivity (ICA100 edge 342)	rfMRI connectivity (ICA100 edge 342)
2771	rfMRI connectivity (ICA100 edge 343)	rfMRI connectivity (ICA100 edge 343)
2772	rfMRI connectivity (ICA100 edge 344)	rfMRI connectivity (ICA100 edge 344)
2773	rfMRI connectivity (ICA100 edge 345)	rfMRI connectivity (ICA100 edge 345)
2774	rfMRI connectivity (ICA100 edge 346)	rfMRI connectivity (ICA100 edge 346)
2775	rfMRI connectivity (ICA100 edge 347)	rfMRI connectivity (ICA100 edge 347)
2776	rfMRI connectivity (ICA100 edge 348)	rfMRI connectivity (ICA100 edge 348)
2777	rfMRI connectivity (ICA100 edge 349)	rfMRI connectivity (ICA100 edge 349)
2778	rfMRI connectivity (ICA100 edge 350)	rfMRI connectivity (ICA100 edge 350)
2779	rfMRI connectivity (ICA100 edge 351)	rfMRI connectivity (ICA100 edge 351)
2780	rfMRI connectivity (ICA100 edge 352)	rfMRI connectivity (ICA100 edge 352)
2781	rfMRI connectivity (ICA100 edge 353)	rfMRI connectivity (ICA100 edge 353)
2782	rfMRI connectivity (ICA100 edge 354)	rfMRI connectivity (ICA100 edge 354)
2783	rfMRI connectivity (ICA100 edge 355)	rfMRI connectivity (ICA100 edge 355)
2784	rfMRI connectivity (ICA100 edge 356)	rfMRI connectivity (ICA100 edge 356)
2785	rfMRI connectivity (ICA100 edge 357)	rfMRI connectivity (ICA100 edge 357)
2786	rfMRI connectivity (ICA100 edge 358)	rfMRI connectivity (ICA100 edge 358)
2787	rfMRI connectivity (ICA100 edge 359)	rfMRI connectivity (ICA100 edge 359)
2788	rfMRI connectivity (ICA100 edge 360)	rfMRI connectivity (ICA100 edge 360)
2789	rfMRI connectivity (ICA100 edge 361)	rfMRI connectivity (ICA100 edge 361)
2790	rfMRI connectivity (ICA100 edge 362)	rfMRI connectivity (ICA100 edge 362)
2791	rfMRI connectivity (ICA100 edge 363)	rfMRI connectivity (ICA100 edge 363)

IDP number	IDP short name	IDP description
2792	rfMRI connectivity (ICA100 edge 364)	rfMRI connectivity (ICA100 edge 364)
2793	rfMRI connectivity (ICA100 edge 365)	rfMRI connectivity (ICA100 edge 365)
2794	rfMRI connectivity (ICA100 edge 366)	rfMRI connectivity (ICA100 edge 366)
2795	rfMRI connectivity (ICA100 edge 367)	rfMRI connectivity (ICA100 edge 367)
2796	rfMRI connectivity (ICA100 edge 368)	rfMRI connectivity (ICA100 edge 368)
2797	rfMRI connectivity (ICA100 edge 369)	rfMRI connectivity (ICA100 edge 369)
2798	rfMRI connectivity (ICA100 edge 370)	rfMRI connectivity (ICA100 edge 370)
2799	rfMRI connectivity (ICA100 edge 371)	rfMRI connectivity (ICA100 edge 371)
2800	rfMRI connectivity (ICA100 edge 372)	rfMRI connectivity (ICA100 edge 372)
2801	rfMRI connectivity (ICA100 edge 373)	rfMRI connectivity (ICA100 edge 373)
2802	rfMRI connectivity (ICA100 edge 374)	rfMRI connectivity (ICA100 edge 374)
2803	rfMRI connectivity (ICA100 edge 375)	rfMRI connectivity (ICA100 edge 375)
2804	rfMRI connectivity (ICA100 edge 376)	rfMRI connectivity (ICA100 edge 376)
2805	rfMRI connectivity (ICA100 edge 377)	rfMRI connectivity (ICA100 edge 377)
2806	rfMRI connectivity (ICA100 edge 378)	rfMRI connectivity (ICA100 edge 378)
2807	rfMRI connectivity (ICA100 edge 379)	rfMRI connectivity (ICA100 edge 379)
2808	rfMRI connectivity (ICA100 edge 380)	rfMRI connectivity (ICA100 edge 380)
2809	rfMRI connectivity (ICA100 edge 381)	rfMRI connectivity (ICA100 edge 381)
2810	rfMRI connectivity (ICA100 edge 382)	rfMRI connectivity (ICA100 edge 382)
2811	rfMRI connectivity (ICA100 edge 383)	rfMRI connectivity (ICA100 edge 383)
2812	rfMRI connectivity (ICA100 edge 384)	rfMRI connectivity (ICA100 edge 384)
2813	rfMRI connectivity (ICA100 edge 385)	rfMRI connectivity (ICA100 edge 385)
2814	rfMRI connectivity (ICA100 edge 386)	rfMRI connectivity (ICA100 edge 386)
2815	rfMRI connectivity (ICA100 edge 387)	rfMRI connectivity (ICA100 edge 387)
2816	rfMRI connectivity (ICA100 edge 388)	rfMRI connectivity (ICA100 edge 388)
2817	rfMRI connectivity (ICA100 edge 389)	rfMRI connectivity (ICA100 edge 389)
2818	rfMRI connectivity (ICA100 edge 390)	rfMRI connectivity (ICA100 edge 390)
2819	rfMRI connectivity (ICA100 edge 391)	rfMRI connectivity (ICA100 edge 391)
2820	rfMRI connectivity (ICA100 edge 392)	rfMRI connectivity (ICA100 edge 392)
2821	rfMRI connectivity (ICA100 edge 393)	rfMRI connectivity (ICA100 edge 393)
2822	rfMRI connectivity (ICA100 edge 394)	rfMRI connectivity (ICA100 edge 394)
2823	rfMRI connectivity (ICA100 edge 395)	rfMRI connectivity (ICA100 edge 395)
2824	rfMRI connectivity (ICA100 edge 396)	rfMRI connectivity (ICA100 edge 396)
2825	rfMRI connectivity (ICA100 edge 397)	rfMRI connectivity (ICA100 edge 397)
2826	rfMRI connectivity (ICA100 edge 398)	rfMRI connectivity (ICA100 edge 398)
2827	rfMRI connectivity (ICA100 edge 399)	rfMRI connectivity (ICA100 edge 399)
2828	rfMRI connectivity (ICA100 edge 400)	rfMRI connectivity (ICA100 edge 400)
2829	rfMRI connectivity (ICA100 edge 401)	rfMRI connectivity (ICA100 edge 401)
2830	rfMRI connectivity (ICA100 edge 402)	rfMRI connectivity (ICA100 edge 402)
2831	rfMRI connectivity (ICA100 edge 403)	rfMRI connectivity (ICA100 edge 403)
2832	rfMRI connectivity (ICA100 edge 404)	rfMRI connectivity (ICA100 edge 404)
2833	rfMRI connectivity (ICA100 edge 405)	rfMRI connectivity (ICA100 edge 405)
2834	rfMRI connectivity (ICA100 edge 406)	rfMRI connectivity (ICA100 edge 406)
2835	rfMRI connectivity (ICA100 edge 407)	rfMRI connectivity (ICA100 edge 407)
2836	rfMRI connectivity (ICA100 edge 408)	rfMRI connectivity (ICA100 edge 408)
2837	rfMRI connectivity (ICA100 edge 409)	rfMRI connectivity (ICA100 edge 409)
2838	rfMRI connectivity (ICA100 edge 410)	rfMRI connectivity (ICA100 edge 410)
2839	rfMRI connectivity (ICA100 edge 411)	rfMRI connectivity (ICA100 edge 411)
2840	rfMRI connectivity (ICA100 edge 412)	rfMRI connectivity (ICA100 edge 412)
2841	rfMRI connectivity (ICA100 edge 413)	rfMRI connectivity (ICA100 edge 413)
2842	rfMRI connectivity (ICA100 edge 414)	rfMRI connectivity (ICA100 edge 414)
2843	rfMRI connectivity (ICA100 edge 415)	rfMRI connectivity (ICA100 edge 415)
2844	rfMRI connectivity (ICA100 edge 416)	rfMRI connectivity (ICA100 edge 416)
2845	rfMRI connectivity (ICA100 edge 417)	rfMRI connectivity (ICA100 edge 417)
2846	rfMRI connectivity (ICA100 edge 418)	rfMRI connectivity (ICA100 edge 418)
2847	rfMRI connectivity (ICA100 edge 419)	rfMRI connectivity (ICA100 edge 419)
2848	rfMRI connectivity (ICA100 edge 420)	rfMRI connectivity (ICA100 edge 420)

IDP number	IDP short name	IDP description
2849	rfMRI connectivity (ICA100 edge 421)	rfMRI connectivity (ICA100 edge 421)
2850	rfMRI connectivity (ICA100 edge 422)	rfMRI connectivity (ICA100 edge 422)
2851	rfMRI connectivity (ICA100 edge 423)	rfMRI connectivity (ICA100 edge 423)
2852	rfMRI connectivity (ICA100 edge 424)	rfMRI connectivity (ICA100 edge 424)
2853	rfMRI connectivity (ICA100 edge 425)	rfMRI connectivity (ICA100 edge 425)
2854	rfMRI connectivity (ICA100 edge 426)	rfMRI connectivity (ICA100 edge 426)
2855	rfMRI connectivity (ICA100 edge 427)	rfMRI connectivity (ICA100 edge 427)
2856	rfMRI connectivity (ICA100 edge 428)	rfMRI connectivity (ICA100 edge 428)
2857	rfMRI connectivity (ICA100 edge 429)	rfMRI connectivity (ICA100 edge 429)
2858	rfMRI connectivity (ICA100 edge 430)	rfMRI connectivity (ICA100 edge 430)
2859	rfMRI connectivity (ICA100 edge 431)	rfMRI connectivity (ICA100 edge 431)
2860	rfMRI connectivity (ICA100 edge 432)	rfMRI connectivity (ICA100 edge 432)
2861	rfMRI connectivity (ICA100 edge 433)	rfMRI connectivity (ICA100 edge 433)
2862	rfMRI connectivity (ICA100 edge 434)	rfMRI connectivity (ICA100 edge 434)
2863	rfMRI connectivity (ICA100 edge 435)	rfMRI connectivity (ICA100 edge 435)
2864	rfMRI connectivity (ICA100 edge 436)	rfMRI connectivity (ICA100 edge 436)
2865	rfMRI connectivity (ICA100 edge 437)	rfMRI connectivity (ICA100 edge 437)
2866	rfMRI connectivity (ICA100 edge 438)	rfMRI connectivity (ICA100 edge 438)
2867	rfMRI connectivity (ICA100 edge 439)	rfMRI connectivity (ICA100 edge 439)
2868	rfMRI connectivity (ICA100 edge 440)	rfMRI connectivity (ICA100 edge 440)
2869	rfMRI connectivity (ICA100 edge 441)	rfMRI connectivity (ICA100 edge 441)
2870	rfMRI connectivity (ICA100 edge 442)	rfMRI connectivity (ICA100 edge 442)
2871	rfMRI connectivity (ICA100 edge 443)	rfMRI connectivity (ICA100 edge 443)
2872	rfMRI connectivity (ICA100 edge 444)	rfMRI connectivity (ICA100 edge 444)
2873	rfMRI connectivity (ICA100 edge 445)	rfMRI connectivity (ICA100 edge 445)
2874	rfMRI connectivity (ICA100 edge 446)	rfMRI connectivity (ICA100 edge 446)
2875	rfMRI connectivity (ICA100 edge 447)	rfMRI connectivity (ICA100 edge 447)
2876	rfMRI connectivity (ICA100 edge 448)	rfMRI connectivity (ICA100 edge 448)
2877	rfMRI connectivity (ICA100 edge 449)	rfMRI connectivity (ICA100 edge 449)
2878	rfMRI connectivity (ICA100 edge 450)	rfMRI connectivity (ICA100 edge 450)
2879	rfMRI connectivity (ICA100 edge 451)	rfMRI connectivity (ICA100 edge 451)
2880	rfMRI connectivity (ICA100 edge 452)	rfMRI connectivity (ICA100 edge 452)
2881	rfMRI connectivity (ICA100 edge 453)	rfMRI connectivity (ICA100 edge 453)
2882	rfMRI connectivity (ICA100 edge 454)	rfMRI connectivity (ICA100 edge 454)
2883	rfMRI connectivity (ICA100 edge 455)	rfMRI connectivity (ICA100 edge 455)
2884	rfMRI connectivity (ICA100 edge 456)	rfMRI connectivity (ICA100 edge 456)
2885	rfMRI connectivity (ICA100 edge 457)	rfMRI connectivity (ICA100 edge 457)
2886	rfMRI connectivity (ICA100 edge 458)	rfMRI connectivity (ICA100 edge 458)
2887	rfMRI connectivity (ICA100 edge 459)	rfMRI connectivity (ICA100 edge 459)
2888	rfMRI connectivity (ICA100 edge 460)	rfMRI connectivity (ICA100 edge 460)
2889	rfMRI connectivity (ICA100 edge 461)	rfMRI connectivity (ICA100 edge 461)
2890	rfMRI connectivity (ICA100 edge 462)	rfMRI connectivity (ICA100 edge 462)
2891	rfMRI connectivity (ICA100 edge 463)	rfMRI connectivity (ICA100 edge 463)
2892	rfMRI connectivity (ICA100 edge 464)	rfMRI connectivity (ICA100 edge 464)
2893	rfMRI connectivity (ICA100 edge 465)	rfMRI connectivity (ICA100 edge 465)
2894	rfMRI connectivity (ICA100 edge 466)	rfMRI connectivity (ICA100 edge 466)
2895	rfMRI connectivity (ICA100 edge 467)	rfMRI connectivity (ICA100 edge 467)
2896	rfMRI connectivity (ICA100 edge 468)	rfMRI connectivity (ICA100 edge 468)
2897	rfMRI connectivity (ICA100 edge 469)	rfMRI connectivity (ICA100 edge 469)
2898	rfMRI connectivity (ICA100 edge 470)	rfMRI connectivity (ICA100 edge 470)
2899	rfMRI connectivity (ICA100 edge 471)	rfMRI connectivity (ICA100 edge 471)
2900	rfMRI connectivity (ICA100 edge 472)	rfMRI connectivity (ICA100 edge 472)
2901	rfMRI connectivity (ICA100 edge 473)	rfMRI connectivity (ICA100 edge 473)
2902	rfMRI connectivity (ICA100 edge 474)	rfMRI connectivity (ICA100 edge 474)
2903	rfMRI connectivity (ICA100 edge 475)	rfMRI connectivity (ICA100 edge 475)
2904	rfMRI connectivity (ICA100 edge 476)	rfMRI connectivity (ICA100 edge 476)
2905	rfMRI connectivity (ICA100 edge 477)	rfMRI connectivity (ICA100 edge 477)

IDP number	IDP short name	IDP description
2906	rfMRI connectivity (ICA100 edge 478)	rfMRI connectivity (ICA100 edge 478)
2907	rfMRI connectivity (ICA100 edge 479)	rfMRI connectivity (ICA100 edge 479)
2908	rfMRI connectivity (ICA100 edge 480)	rfMRI connectivity (ICA100 edge 480)
2909	rfMRI connectivity (ICA100 edge 481)	rfMRI connectivity (ICA100 edge 481)
2910	rfMRI connectivity (ICA100 edge 482)	rfMRI connectivity (ICA100 edge 482)
2911	rfMRI connectivity (ICA100 edge 483)	rfMRI connectivity (ICA100 edge 483)
2912	rfMRI connectivity (ICA100 edge 484)	rfMRI connectivity (ICA100 edge 484)
2913	rfMRI connectivity (ICA100 edge 485)	rfMRI connectivity (ICA100 edge 485)
2914	rfMRI connectivity (ICA100 edge 486)	rfMRI connectivity (ICA100 edge 486)
2915	rfMRI connectivity (ICA100 edge 487)	rfMRI connectivity (ICA100 edge 487)
2916	rfMRI connectivity (ICA100 edge 488)	rfMRI connectivity (ICA100 edge 488)
2917	rfMRI connectivity (ICA100 edge 489)	rfMRI connectivity (ICA100 edge 489)
2918	rfMRI connectivity (ICA100 edge 490)	rfMRI connectivity (ICA100 edge 490)
2919	rfMRI connectivity (ICA100 edge 491)	rfMRI connectivity (ICA100 edge 491)
2920	rfMRI connectivity (ICA100 edge 492)	rfMRI connectivity (ICA100 edge 492)
2921	rfMRI connectivity (ICA100 edge 493)	rfMRI connectivity (ICA100 edge 493)
2922	rfMRI connectivity (ICA100 edge 494)	rfMRI connectivity (ICA100 edge 494)
2923	rfMRI connectivity (ICA100 edge 495)	rfMRI connectivity (ICA100 edge 495)
2924	rfMRI connectivity (ICA100 edge 496)	rfMRI connectivity (ICA100 edge 496)
2925	rfMRI connectivity (ICA100 edge 497)	rfMRI connectivity (ICA100 edge 497)
2926	rfMRI connectivity (ICA100 edge 498)	rfMRI connectivity (ICA100 edge 498)
2927	rfMRI connectivity (ICA100 edge 499)	rfMRI connectivity (ICA100 edge 499)
2928	rfMRI connectivity (ICA100 edge 500)	rfMRI connectivity (ICA100 edge 500)
2929	rfMRI connectivity (ICA100 edge 501)	rfMRI connectivity (ICA100 edge 501)
2930	rfMRI connectivity (ICA100 edge 502)	rfMRI connectivity (ICA100 edge 502)
2931	rfMRI connectivity (ICA100 edge 503)	rfMRI connectivity (ICA100 edge 503)
2932	rfMRI connectivity (ICA100 edge 504)	rfMRI connectivity (ICA100 edge 504)
2933	rfMRI connectivity (ICA100 edge 505)	rfMRI connectivity (ICA100 edge 505)
2934	rfMRI connectivity (ICA100 edge 506)	rfMRI connectivity (ICA100 edge 506)
2935	rfMRI connectivity (ICA100 edge 507)	rfMRI connectivity (ICA100 edge 507)
2936	rfMRI connectivity (ICA100 edge 508)	rfMRI connectivity (ICA100 edge 508)
2937	rfMRI connectivity (ICA100 edge 509)	rfMRI connectivity (ICA100 edge 509)
2938	rfMRI connectivity (ICA100 edge 510)	rfMRI connectivity (ICA100 edge 510)
2939	rfMRI connectivity (ICA100 edge 511)	rfMRI connectivity (ICA100 edge 511)
2940	rfMRI connectivity (ICA100 edge 512)	rfMRI connectivity (ICA100 edge 512)
2941	rfMRI connectivity (ICA100 edge 513)	rfMRI connectivity (ICA100 edge 513)
2942	rfMRI connectivity (ICA100 edge 514)	rfMRI connectivity (ICA100 edge 514)
2943	rfMRI connectivity (ICA100 edge 515)	rfMRI connectivity (ICA100 edge 515)
2944	rfMRI connectivity (ICA100 edge 516)	rfMRI connectivity (ICA100 edge 516)
2945	rfMRI connectivity (ICA100 edge 517)	rfMRI connectivity (ICA100 edge 517)
2946	rfMRI connectivity (ICA100 edge 518)	rfMRI connectivity (ICA100 edge 518)
2947	rfMRI connectivity (ICA100 edge 519)	rfMRI connectivity (ICA100 edge 519)
2948	rfMRI connectivity (ICA100 edge 520)	rfMRI connectivity (ICA100 edge 520)
2949	rfMRI connectivity (ICA100 edge 521)	rfMRI connectivity (ICA100 edge 521)
2950	rfMRI connectivity (ICA100 edge 522)	rfMRI connectivity (ICA100 edge 522)
2951	rfMRI connectivity (ICA100 edge 523)	rfMRI connectivity (ICA100 edge 523)
2952	rfMRI connectivity (ICA100 edge 524)	rfMRI connectivity (ICA100 edge 524)
2953	rfMRI connectivity (ICA100 edge 525)	rfMRI connectivity (ICA100 edge 525)
2954	rfMRI connectivity (ICA100 edge 526)	rfMRI connectivity (ICA100 edge 526)
2955	rfMRI connectivity (ICA100 edge 527)	rfMRI connectivity (ICA100 edge 527)
2956	rfMRI connectivity (ICA100 edge 528)	rfMRI connectivity (ICA100 edge 528)
2957	rfMRI connectivity (ICA100 edge 529)	rfMRI connectivity (ICA100 edge 529)
2958	rfMRI connectivity (ICA100 edge 530)	rfMRI connectivity (ICA100 edge 530)
2959	rfMRI connectivity (ICA100 edge 531)	rfMRI connectivity (ICA100 edge 531)
2960	rfMRI connectivity (ICA100 edge 532)	rfMRI connectivity (ICA100 edge 532)
2961	rfMRI connectivity (ICA100 edge 533)	rfMRI connectivity (ICA100 edge 533)
2962	rfMRI connectivity (ICA100 edge 534)	rfMRI connectivity (ICA100 edge 534)

IDP number	IDP short name	IDP description
2963	rfMRI connectivity (ICA100 edge 535)	rfMRI connectivity (ICA100 edge 535)
2964	rfMRI connectivity (ICA100 edge 536)	rfMRI connectivity (ICA100 edge 536)
2965	rfMRI connectivity (ICA100 edge 537)	rfMRI connectivity (ICA100 edge 537)
2966	rfMRI connectivity (ICA100 edge 538)	rfMRI connectivity (ICA100 edge 538)
2967	rfMRI connectivity (ICA100 edge 539)	rfMRI connectivity (ICA100 edge 539)
2968	rfMRI connectivity (ICA100 edge 540)	rfMRI connectivity (ICA100 edge 540)
2969	rfMRI connectivity (ICA100 edge 541)	rfMRI connectivity (ICA100 edge 541)
2970	rfMRI connectivity (ICA100 edge 542)	rfMRI connectivity (ICA100 edge 542)
2971	rfMRI connectivity (ICA100 edge 543)	rfMRI connectivity (ICA100 edge 543)
2972	rfMRI connectivity (ICA100 edge 544)	rfMRI connectivity (ICA100 edge 544)
2973	rfMRI connectivity (ICA100 edge 545)	rfMRI connectivity (ICA100 edge 545)
2974	rfMRI connectivity (ICA100 edge 546)	rfMRI connectivity (ICA100 edge 546)
2975	rfMRI connectivity (ICA100 edge 547)	rfMRI connectivity (ICA100 edge 547)
2976	rfMRI connectivity (ICA100 edge 548)	rfMRI connectivity (ICA100 edge 548)
2977	rfMRI connectivity (ICA100 edge 549)	rfMRI connectivity (ICA100 edge 549)
2978	rfMRI connectivity (ICA100 edge 550)	rfMRI connectivity (ICA100 edge 550)
2979	rfMRI connectivity (ICA100 edge 551)	rfMRI connectivity (ICA100 edge 551)
2980	rfMRI connectivity (ICA100 edge 552)	rfMRI connectivity (ICA100 edge 552)
2981	rfMRI connectivity (ICA100 edge 553)	rfMRI connectivity (ICA100 edge 553)
2982	rfMRI connectivity (ICA100 edge 554)	rfMRI connectivity (ICA100 edge 554)
2983	rfMRI connectivity (ICA100 edge 555)	rfMRI connectivity (ICA100 edge 555)
2984	rfMRI connectivity (ICA100 edge 556)	rfMRI connectivity (ICA100 edge 556)
2985	rfMRI connectivity (ICA100 edge 557)	rfMRI connectivity (ICA100 edge 557)
2986	rfMRI connectivity (ICA100 edge 558)	rfMRI connectivity (ICA100 edge 558)
2987	rfMRI connectivity (ICA100 edge 559)	rfMRI connectivity (ICA100 edge 559)
2988	rfMRI connectivity (ICA100 edge 560)	rfMRI connectivity (ICA100 edge 560)
2989	rfMRI connectivity (ICA100 edge 561)	rfMRI connectivity (ICA100 edge 561)
2990	rfMRI connectivity (ICA100 edge 562)	rfMRI connectivity (ICA100 edge 562)
2991	rfMRI connectivity (ICA100 edge 563)	rfMRI connectivity (ICA100 edge 563)
2992	rfMRI connectivity (ICA100 edge 564)	rfMRI connectivity (ICA100 edge 564)
2993	rfMRI connectivity (ICA100 edge 565)	rfMRI connectivity (ICA100 edge 565)
2994	rfMRI connectivity (ICA100 edge 566)	rfMRI connectivity (ICA100 edge 566)
2995	rfMRI connectivity (ICA100 edge 567)	rfMRI connectivity (ICA100 edge 567)
2996	rfMRI connectivity (ICA100 edge 568)	rfMRI connectivity (ICA100 edge 568)
2997	rfMRI connectivity (ICA100 edge 569)	rfMRI connectivity (ICA100 edge 569)
2998	rfMRI connectivity (ICA100 edge 570)	rfMRI connectivity (ICA100 edge 570)
2999	rfMRI connectivity (ICA100 edge 571)	rfMRI connectivity (ICA100 edge 571)
3000	rfMRI connectivity (ICA100 edge 572)	rfMRI connectivity (ICA100 edge 572)
3001	rfMRI connectivity (ICA100 edge 573)	rfMRI connectivity (ICA100 edge 573)
3002	rfMRI connectivity (ICA100 edge 574)	rfMRI connectivity (ICA100 edge 574)
3003	rfMRI connectivity (ICA100 edge 575)	rfMRI connectivity (ICA100 edge 575)
3004	rfMRI connectivity (ICA100 edge 576)	rfMRI connectivity (ICA100 edge 576)
3005	rfMRI connectivity (ICA100 edge 577)	rfMRI connectivity (ICA100 edge 577)
3006	rfMRI connectivity (ICA100 edge 578)	rfMRI connectivity (ICA100 edge 578)
3007	rfMRI connectivity (ICA100 edge 579)	rfMRI connectivity (ICA100 edge 579)
3008	rfMRI connectivity (ICA100 edge 580)	rfMRI connectivity (ICA100 edge 580)
3009	rfMRI connectivity (ICA100 edge 581)	rfMRI connectivity (ICA100 edge 581)
3010	rfMRI connectivity (ICA100 edge 582)	rfMRI connectivity (ICA100 edge 582)
3011	rfMRI connectivity (ICA100 edge 583)	rfMRI connectivity (ICA100 edge 583)
3012	rfMRI connectivity (ICA100 edge 584)	rfMRI connectivity (ICA100 edge 584)
3013	rfMRI connectivity (ICA100 edge 585)	rfMRI connectivity (ICA100 edge 585)
3014	rfMRI connectivity (ICA100 edge 586)	rfMRI connectivity (ICA100 edge 586)
3015	rfMRI connectivity (ICA100 edge 587)	rfMRI connectivity (ICA100 edge 587)
3016	rfMRI connectivity (ICA100 edge 588)	rfMRI connectivity (ICA100 edge 588)
3017	rfMRI connectivity (ICA100 edge 589)	rfMRI connectivity (ICA100 edge 589)
3018	rfMRI connectivity (ICA100 edge 590)	rfMRI connectivity (ICA100 edge 590)
3019	rfMRI connectivity (ICA100 edge 591)	rfMRI connectivity (ICA100 edge 591)

IDP number	IDP short name	IDP description
3020	rfMRI connectivity (ICA100 edge 592)	rfMRI connectivity (ICA100 edge 592)
3021	rfMRI connectivity (ICA100 edge 593)	rfMRI connectivity (ICA100 edge 593)
3022	rfMRI connectivity (ICA100 edge 594)	rfMRI connectivity (ICA100 edge 594)
3023	rfMRI connectivity (ICA100 edge 595)	rfMRI connectivity (ICA100 edge 595)
3024	rfMRI connectivity (ICA100 edge 596)	rfMRI connectivity (ICA100 edge 596)
3025	rfMRI connectivity (ICA100 edge 597)	rfMRI connectivity (ICA100 edge 597)
3026	rfMRI connectivity (ICA100 edge 598)	rfMRI connectivity (ICA100 edge 598)
3027	rfMRI connectivity (ICA100 edge 599)	rfMRI connectivity (ICA100 edge 599)
3028	rfMRI connectivity (ICA100 edge 600)	rfMRI connectivity (ICA100 edge 600)
3029	rfMRI connectivity (ICA100 edge 601)	rfMRI connectivity (ICA100 edge 601)
3030	rfMRI connectivity (ICA100 edge 602)	rfMRI connectivity (ICA100 edge 602)
3031	rfMRI connectivity (ICA100 edge 603)	rfMRI connectivity (ICA100 edge 603)
3032	rfMRI connectivity (ICA100 edge 604)	rfMRI connectivity (ICA100 edge 604)
3033	rfMRI connectivity (ICA100 edge 605)	rfMRI connectivity (ICA100 edge 605)
3034	rfMRI connectivity (ICA100 edge 606)	rfMRI connectivity (ICA100 edge 606)
3035	rfMRI connectivity (ICA100 edge 607)	rfMRI connectivity (ICA100 edge 607)
3036	rfMRI connectivity (ICA100 edge 608)	rfMRI connectivity (ICA100 edge 608)
3037	rfMRI connectivity (ICA100 edge 609)	rfMRI connectivity (ICA100 edge 609)
3038	rfMRI connectivity (ICA100 edge 610)	rfMRI connectivity (ICA100 edge 610)
3039	rfMRI connectivity (ICA100 edge 611)	rfMRI connectivity (ICA100 edge 611)
3040	rfMRI connectivity (ICA100 edge 612)	rfMRI connectivity (ICA100 edge 612)
3041	rfMRI connectivity (ICA100 edge 613)	rfMRI connectivity (ICA100 edge 613)
3042	rfMRI connectivity (ICA100 edge 614)	rfMRI connectivity (ICA100 edge 614)
3043	rfMRI connectivity (ICA100 edge 615)	rfMRI connectivity (ICA100 edge 615)
3044	rfMRI connectivity (ICA100 edge 616)	rfMRI connectivity (ICA100 edge 616)
3045	rfMRI connectivity (ICA100 edge 617)	rfMRI connectivity (ICA100 edge 617)
3046	rfMRI connectivity (ICA100 edge 618)	rfMRI connectivity (ICA100 edge 618)
3047	rfMRI connectivity (ICA100 edge 619)	rfMRI connectivity (ICA100 edge 619)
3048	rfMRI connectivity (ICA100 edge 620)	rfMRI connectivity (ICA100 edge 620)
3049	rfMRI connectivity (ICA100 edge 621)	rfMRI connectivity (ICA100 edge 621)
3050	rfMRI connectivity (ICA100 edge 622)	rfMRI connectivity (ICA100 edge 622)
3051	rfMRI connectivity (ICA100 edge 623)	rfMRI connectivity (ICA100 edge 623)
3052	rfMRI connectivity (ICA100 edge 624)	rfMRI connectivity (ICA100 edge 624)
3053	rfMRI connectivity (ICA100 edge 625)	rfMRI connectivity (ICA100 edge 625)
3054	rfMRI connectivity (ICA100 edge 626)	rfMRI connectivity (ICA100 edge 626)
3055	rfMRI connectivity (ICA100 edge 627)	rfMRI connectivity (ICA100 edge 627)
3056	rfMRI connectivity (ICA100 edge 628)	rfMRI connectivity (ICA100 edge 628)
3057	rfMRI connectivity (ICA100 edge 629)	rfMRI connectivity (ICA100 edge 629)
3058	rfMRI connectivity (ICA100 edge 630)	rfMRI connectivity (ICA100 edge 630)
3059	rfMRI connectivity (ICA100 edge 631)	rfMRI connectivity (ICA100 edge 631)
3060	rfMRI connectivity (ICA100 edge 632)	rfMRI connectivity (ICA100 edge 632)
3061	rfMRI connectivity (ICA100 edge 633)	rfMRI connectivity (ICA100 edge 633)
3062	rfMRI connectivity (ICA100 edge 634)	rfMRI connectivity (ICA100 edge 634)
3063	rfMRI connectivity (ICA100 edge 635)	rfMRI connectivity (ICA100 edge 635)
3064	rfMRI connectivity (ICA100 edge 636)	rfMRI connectivity (ICA100 edge 636)
3065	rfMRI connectivity (ICA100 edge 637)	rfMRI connectivity (ICA100 edge 637)
3066	rfMRI connectivity (ICA100 edge 638)	rfMRI connectivity (ICA100 edge 638)
3067	rfMRI connectivity (ICA100 edge 639)	rfMRI connectivity (ICA100 edge 639)
3068	rfMRI connectivity (ICA100 edge 640)	rfMRI connectivity (ICA100 edge 640)
3069	rfMRI connectivity (ICA100 edge 641)	rfMRI connectivity (ICA100 edge 641)
3070	rfMRI connectivity (ICA100 edge 642)	rfMRI connectivity (ICA100 edge 642)
3071	rfMRI connectivity (ICA100 edge 643)	rfMRI connectivity (ICA100 edge 643)
3072	rfMRI connectivity (ICA100 edge 644)	rfMRI connectivity (ICA100 edge 644)
3073	rfMRI connectivity (ICA100 edge 645)	rfMRI connectivity (ICA100 edge 645)
3074	rfMRI connectivity (ICA100 edge 646)	rfMRI connectivity (ICA100 edge 646)
3075	rfMRI connectivity (ICA100 edge 647)	rfMRI connectivity (ICA100 edge 647)
3076	rfMRI connectivity (ICA100 edge 648)	rfMRI connectivity (ICA100 edge 648)

IDP number	IDP short name	IDP description
3077	rfMRI connectivity (ICA100 edge 649)	rfMRI connectivity (ICA100 edge 649)
3078	rfMRI connectivity (ICA100 edge 650)	rfMRI connectivity (ICA100 edge 650)
3079	rfMRI connectivity (ICA100 edge 651)	rfMRI connectivity (ICA100 edge 651)
3080	rfMRI connectivity (ICA100 edge 652)	rfMRI connectivity (ICA100 edge 652)
3081	rfMRI connectivity (ICA100 edge 653)	rfMRI connectivity (ICA100 edge 653)
3082	rfMRI connectivity (ICA100 edge 654)	rfMRI connectivity (ICA100 edge 654)
3083	rfMRI connectivity (ICA100 edge 655)	rfMRI connectivity (ICA100 edge 655)
3084	rfMRI connectivity (ICA100 edge 656)	rfMRI connectivity (ICA100 edge 656)
3085	rfMRI connectivity (ICA100 edge 657)	rfMRI connectivity (ICA100 edge 657)
3086	rfMRI connectivity (ICA100 edge 658)	rfMRI connectivity (ICA100 edge 658)
3087	rfMRI connectivity (ICA100 edge 659)	rfMRI connectivity (ICA100 edge 659)
3088	rfMRI connectivity (ICA100 edge 660)	rfMRI connectivity (ICA100 edge 660)
3089	rfMRI connectivity (ICA100 edge 661)	rfMRI connectivity (ICA100 edge 661)
3090	rfMRI connectivity (ICA100 edge 662)	rfMRI connectivity (ICA100 edge 662)
3091	rfMRI connectivity (ICA100 edge 663)	rfMRI connectivity (ICA100 edge 663)
3092	rfMRI connectivity (ICA100 edge 664)	rfMRI connectivity (ICA100 edge 664)
3093	rfMRI connectivity (ICA100 edge 665)	rfMRI connectivity (ICA100 edge 665)
3094	rfMRI connectivity (ICA100 edge 666)	rfMRI connectivity (ICA100 edge 666)
3095	rfMRI connectivity (ICA100 edge 667)	rfMRI connectivity (ICA100 edge 667)
3096	rfMRI connectivity (ICA100 edge 668)	rfMRI connectivity (ICA100 edge 668)
3097	rfMRI connectivity (ICA100 edge 669)	rfMRI connectivity (ICA100 edge 669)
3098	rfMRI connectivity (ICA100 edge 670)	rfMRI connectivity (ICA100 edge 670)
3099	rfMRI connectivity (ICA100 edge 671)	rfMRI connectivity (ICA100 edge 671)
3100	rfMRI connectivity (ICA100 edge 672)	rfMRI connectivity (ICA100 edge 672)
3101	rfMRI connectivity (ICA100 edge 673)	rfMRI connectivity (ICA100 edge 673)
3102	rfMRI connectivity (ICA100 edge 674)	rfMRI connectivity (ICA100 edge 674)
3103	rfMRI connectivity (ICA100 edge 675)	rfMRI connectivity (ICA100 edge 675)
3104	rfMRI connectivity (ICA100 edge 676)	rfMRI connectivity (ICA100 edge 676)
3105	rfMRI connectivity (ICA100 edge 677)	rfMRI connectivity (ICA100 edge 677)
3106	rfMRI connectivity (ICA100 edge 678)	rfMRI connectivity (ICA100 edge 678)
3107	rfMRI connectivity (ICA100 edge 679)	rfMRI connectivity (ICA100 edge 679)
3108	rfMRI connectivity (ICA100 edge 680)	rfMRI connectivity (ICA100 edge 680)
3109	rfMRI connectivity (ICA100 edge 681)	rfMRI connectivity (ICA100 edge 681)
3110	rfMRI connectivity (ICA100 edge 682)	rfMRI connectivity (ICA100 edge 682)
3111	rfMRI connectivity (ICA100 edge 683)	rfMRI connectivity (ICA100 edge 683)
3112	rfMRI connectivity (ICA100 edge 684)	rfMRI connectivity (ICA100 edge 684)
3113	rfMRI connectivity (ICA100 edge 685)	rfMRI connectivity (ICA100 edge 685)
3114	rfMRI connectivity (ICA100 edge 686)	rfMRI connectivity (ICA100 edge 686)
3115	rfMRI connectivity (ICA100 edge 687)	rfMRI connectivity (ICA100 edge 687)
3116	rfMRI connectivity (ICA100 edge 688)	rfMRI connectivity (ICA100 edge 688)
3117	rfMRI connectivity (ICA100 edge 689)	rfMRI connectivity (ICA100 edge 689)
3118	rfMRI connectivity (ICA100 edge 690)	rfMRI connectivity (ICA100 edge 690)
3119	rfMRI connectivity (ICA100 edge 691)	rfMRI connectivity (ICA100 edge 691)
3120	rfMRI connectivity (ICA100 edge 692)	rfMRI connectivity (ICA100 edge 692)
3121	rfMRI connectivity (ICA100 edge 693)	rfMRI connectivity (ICA100 edge 693)
3122	rfMRI connectivity (ICA100 edge 694)	rfMRI connectivity (ICA100 edge 694)
3123	rfMRI connectivity (ICA100 edge 695)	rfMRI connectivity (ICA100 edge 695)
3124	rfMRI connectivity (ICA100 edge 696)	rfMRI connectivity (ICA100 edge 696)
3125	rfMRI connectivity (ICA100 edge 697)	rfMRI connectivity (ICA100 edge 697)
3126	rfMRI connectivity (ICA100 edge 698)	rfMRI connectivity (ICA100 edge 698)
3127	rfMRI connectivity (ICA100 edge 699)	rfMRI connectivity (ICA100 edge 699)
3128	rfMRI connectivity (ICA100 edge 700)	rfMRI connectivity (ICA100 edge 700)
3129	rfMRI connectivity (ICA100 edge 701)	rfMRI connectivity (ICA100 edge 701)
3130	rfMRI connectivity (ICA100 edge 702)	rfMRI connectivity (ICA100 edge 702)
3131	rfMRI connectivity (ICA100 edge 703)	rfMRI connectivity (ICA100 edge 703)
3132	rfMRI connectivity (ICA100 edge 704)	rfMRI connectivity (ICA100 edge 704)
3133	rfMRI connectivity (ICA100 edge 705)	rfMRI connectivity (ICA100 edge 705)

IDP number	IDP short name	IDP description
3134	rfMRI connectivity (ICA100 edge 706)	rfMRI connectivity (ICA100 edge 706)
3135	rfMRI connectivity (ICA100 edge 707)	rfMRI connectivity (ICA100 edge 707)
3136	rfMRI connectivity (ICA100 edge 708)	rfMRI connectivity (ICA100 edge 708)
3137	rfMRI connectivity (ICA100 edge 709)	rfMRI connectivity (ICA100 edge 709)
3138	rfMRI connectivity (ICA100 edge 710)	rfMRI connectivity (ICA100 edge 710)
3139	rfMRI connectivity (ICA100 edge 711)	rfMRI connectivity (ICA100 edge 711)
3140	rfMRI connectivity (ICA100 edge 712)	rfMRI connectivity (ICA100 edge 712)
3141	rfMRI connectivity (ICA100 edge 713)	rfMRI connectivity (ICA100 edge 713)
3142	rfMRI connectivity (ICA100 edge 714)	rfMRI connectivity (ICA100 edge 714)
3143	rfMRI connectivity (ICA100 edge 715)	rfMRI connectivity (ICA100 edge 715)
3144	rfMRI connectivity (ICA100 edge 716)	rfMRI connectivity (ICA100 edge 716)
3145	rfMRI connectivity (ICA100 edge 717)	rfMRI connectivity (ICA100 edge 717)
3146	rfMRI connectivity (ICA100 edge 718)	rfMRI connectivity (ICA100 edge 718)
3147	rfMRI connectivity (ICA100 edge 719)	rfMRI connectivity (ICA100 edge 719)
3148	rfMRI connectivity (ICA100 edge 720)	rfMRI connectivity (ICA100 edge 720)
3149	rfMRI connectivity (ICA100 edge 721)	rfMRI connectivity (ICA100 edge 721)
3150	rfMRI connectivity (ICA100 edge 722)	rfMRI connectivity (ICA100 edge 722)
3151	rfMRI connectivity (ICA100 edge 723)	rfMRI connectivity (ICA100 edge 723)
3152	rfMRI connectivity (ICA100 edge 724)	rfMRI connectivity (ICA100 edge 724)
3153	rfMRI connectivity (ICA100 edge 725)	rfMRI connectivity (ICA100 edge 725)
3154	rfMRI connectivity (ICA100 edge 726)	rfMRI connectivity (ICA100 edge 726)
3155	rfMRI connectivity (ICA100 edge 727)	rfMRI connectivity (ICA100 edge 727)
3156	rfMRI connectivity (ICA100 edge 728)	rfMRI connectivity (ICA100 edge 728)
3157	rfMRI connectivity (ICA100 edge 729)	rfMRI connectivity (ICA100 edge 729)
3158	rfMRI connectivity (ICA100 edge 730)	rfMRI connectivity (ICA100 edge 730)
3159	rfMRI connectivity (ICA100 edge 731)	rfMRI connectivity (ICA100 edge 731)
3160	rfMRI connectivity (ICA100 edge 732)	rfMRI connectivity (ICA100 edge 732)
3161	rfMRI connectivity (ICA100 edge 733)	rfMRI connectivity (ICA100 edge 733)
3162	rfMRI connectivity (ICA100 edge 734)	rfMRI connectivity (ICA100 edge 734)
3163	rfMRI connectivity (ICA100 edge 735)	rfMRI connectivity (ICA100 edge 735)
3164	rfMRI connectivity (ICA100 edge 736)	rfMRI connectivity (ICA100 edge 736)
3165	rfMRI connectivity (ICA100 edge 737)	rfMRI connectivity (ICA100 edge 737)
3166	rfMRI connectivity (ICA100 edge 738)	rfMRI connectivity (ICA100 edge 738)
3167	rfMRI connectivity (ICA100 edge 739)	rfMRI connectivity (ICA100 edge 739)
3168	rfMRI connectivity (ICA100 edge 740)	rfMRI connectivity (ICA100 edge 740)
3169	rfMRI connectivity (ICA100 edge 741)	rfMRI connectivity (ICA100 edge 741)
3170	rfMRI connectivity (ICA100 edge 742)	rfMRI connectivity (ICA100 edge 742)
3171	rfMRI connectivity (ICA100 edge 743)	rfMRI connectivity (ICA100 edge 743)
3172	rfMRI connectivity (ICA100 edge 744)	rfMRI connectivity (ICA100 edge 744)
3173	rfMRI connectivity (ICA100 edge 745)	rfMRI connectivity (ICA100 edge 745)
3174	rfMRI connectivity (ICA100 edge 746)	rfMRI connectivity (ICA100 edge 746)
3175	rfMRI connectivity (ICA100 edge 747)	rfMRI connectivity (ICA100 edge 747)
3176	rfMRI connectivity (ICA100 edge 748)	rfMRI connectivity (ICA100 edge 748)
3177	rfMRI connectivity (ICA100 edge 749)	rfMRI connectivity (ICA100 edge 749)
3178	rfMRI connectivity (ICA100 edge 750)	rfMRI connectivity (ICA100 edge 750)
3179	rfMRI connectivity (ICA100 edge 751)	rfMRI connectivity (ICA100 edge 751)
3180	rfMRI connectivity (ICA100 edge 752)	rfMRI connectivity (ICA100 edge 752)
3181	rfMRI connectivity (ICA100 edge 753)	rfMRI connectivity (ICA100 edge 753)
3182	rfMRI connectivity (ICA100 edge 754)	rfMRI connectivity (ICA100 edge 754)
3183	rfMRI connectivity (ICA100 edge 755)	rfMRI connectivity (ICA100 edge 755)
3184	rfMRI connectivity (ICA100 edge 756)	rfMRI connectivity (ICA100 edge 756)
3185	rfMRI connectivity (ICA100 edge 757)	rfMRI connectivity (ICA100 edge 757)
3186	rfMRI connectivity (ICA100 edge 758)	rfMRI connectivity (ICA100 edge 758)
3187	rfMRI connectivity (ICA100 edge 759)	rfMRI connectivity (ICA100 edge 759)
3188	rfMRI connectivity (ICA100 edge 760)	rfMRI connectivity (ICA100 edge 760)
3189	rfMRI connectivity (ICA100 edge 761)	rfMRI connectivity (ICA100 edge 761)
3190	rfMRI connectivity (ICA100 edge 762)	rfMRI connectivity (ICA100 edge 762)

IDP number	IDP short name	IDP description
3191	rfMRI connectivity (ICA100 edge 763)	rfMRI connectivity (ICA100 edge 763)
3192	rfMRI connectivity (ICA100 edge 764)	rfMRI connectivity (ICA100 edge 764)
3193	rfMRI connectivity (ICA100 edge 765)	rfMRI connectivity (ICA100 edge 765)
3194	rfMRI connectivity (ICA100 edge 766)	rfMRI connectivity (ICA100 edge 766)
3195	rfMRI connectivity (ICA100 edge 767)	rfMRI connectivity (ICA100 edge 767)
3196	rfMRI connectivity (ICA100 edge 768)	rfMRI connectivity (ICA100 edge 768)
3197	rfMRI connectivity (ICA100 edge 769)	rfMRI connectivity (ICA100 edge 769)
3198	rfMRI connectivity (ICA100 edge 770)	rfMRI connectivity (ICA100 edge 770)
3199	rfMRI connectivity (ICA100 edge 771)	rfMRI connectivity (ICA100 edge 771)
3200	rfMRI connectivity (ICA100 edge 772)	rfMRI connectivity (ICA100 edge 772)
3201	rfMRI connectivity (ICA100 edge 773)	rfMRI connectivity (ICA100 edge 773)
3202	rfMRI connectivity (ICA100 edge 774)	rfMRI connectivity (ICA100 edge 774)
3203	rfMRI connectivity (ICA100 edge 775)	rfMRI connectivity (ICA100 edge 775)
3204	rfMRI connectivity (ICA100 edge 776)	rfMRI connectivity (ICA100 edge 776)
3205	rfMRI connectivity (ICA100 edge 777)	rfMRI connectivity (ICA100 edge 777)
3206	rfMRI connectivity (ICA100 edge 778)	rfMRI connectivity (ICA100 edge 778)
3207	rfMRI connectivity (ICA100 edge 779)	rfMRI connectivity (ICA100 edge 779)
3208	rfMRI connectivity (ICA100 edge 780)	rfMRI connectivity (ICA100 edge 780)
3209	rfMRI connectivity (ICA100 edge 781)	rfMRI connectivity (ICA100 edge 781)
3210	rfMRI connectivity (ICA100 edge 782)	rfMRI connectivity (ICA100 edge 782)
3211	rfMRI connectivity (ICA100 edge 783)	rfMRI connectivity (ICA100 edge 783)
3212	rfMRI connectivity (ICA100 edge 784)	rfMRI connectivity (ICA100 edge 784)
3213	rfMRI connectivity (ICA100 edge 785)	rfMRI connectivity (ICA100 edge 785)
3214	rfMRI connectivity (ICA100 edge 786)	rfMRI connectivity (ICA100 edge 786)
3215	rfMRI connectivity (ICA100 edge 787)	rfMRI connectivity (ICA100 edge 787)
3216	rfMRI connectivity (ICA100 edge 788)	rfMRI connectivity (ICA100 edge 788)
3217	rfMRI connectivity (ICA100 edge 789)	rfMRI connectivity (ICA100 edge 789)
3218	rfMRI connectivity (ICA100 edge 790)	rfMRI connectivity (ICA100 edge 790)
3219	rfMRI connectivity (ICA100 edge 791)	rfMRI connectivity (ICA100 edge 791)
3220	rfMRI connectivity (ICA100 edge 792)	rfMRI connectivity (ICA100 edge 792)
3221	rfMRI connectivity (ICA100 edge 793)	rfMRI connectivity (ICA100 edge 793)
3222	rfMRI connectivity (ICA100 edge 794)	rfMRI connectivity (ICA100 edge 794)
3223	rfMRI connectivity (ICA100 edge 795)	rfMRI connectivity (ICA100 edge 795)
3224	rfMRI connectivity (ICA100 edge 796)	rfMRI connectivity (ICA100 edge 796)
3225	rfMRI connectivity (ICA100 edge 797)	rfMRI connectivity (ICA100 edge 797)
3226	rfMRI connectivity (ICA100 edge 798)	rfMRI connectivity (ICA100 edge 798)
3227	rfMRI connectivity (ICA100 edge 799)	rfMRI connectivity (ICA100 edge 799)
3228	rfMRI connectivity (ICA100 edge 800)	rfMRI connectivity (ICA100 edge 800)
3229	rfMRI connectivity (ICA100 edge 801)	rfMRI connectivity (ICA100 edge 801)
3230	rfMRI connectivity (ICA100 edge 802)	rfMRI connectivity (ICA100 edge 802)
3231	rfMRI connectivity (ICA100 edge 803)	rfMRI connectivity (ICA100 edge 803)
3232	rfMRI connectivity (ICA100 edge 804)	rfMRI connectivity (ICA100 edge 804)
3233	rfMRI connectivity (ICA100 edge 805)	rfMRI connectivity (ICA100 edge 805)
3234	rfMRI connectivity (ICA100 edge 806)	rfMRI connectivity (ICA100 edge 806)
3235	rfMRI connectivity (ICA100 edge 807)	rfMRI connectivity (ICA100 edge 807)
3236	rfMRI connectivity (ICA100 edge 808)	rfMRI connectivity (ICA100 edge 808)
3237	rfMRI connectivity (ICA100 edge 809)	rfMRI connectivity (ICA100 edge 809)
3238	rfMRI connectivity (ICA100 edge 810)	rfMRI connectivity (ICA100 edge 810)
3239	rfMRI connectivity (ICA100 edge 811)	rfMRI connectivity (ICA100 edge 811)
3240	rfMRI connectivity (ICA100 edge 812)	rfMRI connectivity (ICA100 edge 812)
3241	rfMRI connectivity (ICA100 edge 813)	rfMRI connectivity (ICA100 edge 813)
3242	rfMRI connectivity (ICA100 edge 814)	rfMRI connectivity (ICA100 edge 814)
3243	rfMRI connectivity (ICA100 edge 815)	rfMRI connectivity (ICA100 edge 815)
3244	rfMRI connectivity (ICA100 edge 816)	rfMRI connectivity (ICA100 edge 816)
3245	rfMRI connectivity (ICA100 edge 817)	rfMRI connectivity (ICA100 edge 817)
3246	rfMRI connectivity (ICA100 edge 818)	rfMRI connectivity (ICA100 edge 818)
3247	rfMRI connectivity (ICA100 edge 819)	rfMRI connectivity (ICA100 edge 819)

IDP number	IDP short name	IDP description
3248	rfMRI connectivity (ICA100 edge 820)	rfMRI connectivity (ICA100 edge 820)
3249	rfMRI connectivity (ICA100 edge 821)	rfMRI connectivity (ICA100 edge 821)
3250	rfMRI connectivity (ICA100 edge 822)	rfMRI connectivity (ICA100 edge 822)
3251	rfMRI connectivity (ICA100 edge 823)	rfMRI connectivity (ICA100 edge 823)
3252	rfMRI connectivity (ICA100 edge 824)	rfMRI connectivity (ICA100 edge 824)
3253	rfMRI connectivity (ICA100 edge 825)	rfMRI connectivity (ICA100 edge 825)
3254	rfMRI connectivity (ICA100 edge 826)	rfMRI connectivity (ICA100 edge 826)
3255	rfMRI connectivity (ICA100 edge 827)	rfMRI connectivity (ICA100 edge 827)
3256	rfMRI connectivity (ICA100 edge 828)	rfMRI connectivity (ICA100 edge 828)
3257	rfMRI connectivity (ICA100 edge 829)	rfMRI connectivity (ICA100 edge 829)
3258	rfMRI connectivity (ICA100 edge 830)	rfMRI connectivity (ICA100 edge 830)
3259	rfMRI connectivity (ICA100 edge 831)	rfMRI connectivity (ICA100 edge 831)
3260	rfMRI connectivity (ICA100 edge 832)	rfMRI connectivity (ICA100 edge 832)
3261	rfMRI connectivity (ICA100 edge 833)	rfMRI connectivity (ICA100 edge 833)
3262	rfMRI connectivity (ICA100 edge 834)	rfMRI connectivity (ICA100 edge 834)
3263	rfMRI connectivity (ICA100 edge 835)	rfMRI connectivity (ICA100 edge 835)
3264	rfMRI connectivity (ICA100 edge 836)	rfMRI connectivity (ICA100 edge 836)
3265	rfMRI connectivity (ICA100 edge 837)	rfMRI connectivity (ICA100 edge 837)
3266	rfMRI connectivity (ICA100 edge 838)	rfMRI connectivity (ICA100 edge 838)
3267	rfMRI connectivity (ICA100 edge 839)	rfMRI connectivity (ICA100 edge 839)
3268	rfMRI connectivity (ICA100 edge 840)	rfMRI connectivity (ICA100 edge 840)
3269	rfMRI connectivity (ICA100 edge 841)	rfMRI connectivity (ICA100 edge 841)
3270	rfMRI connectivity (ICA100 edge 842)	rfMRI connectivity (ICA100 edge 842)
3271	rfMRI connectivity (ICA100 edge 843)	rfMRI connectivity (ICA100 edge 843)
3272	rfMRI connectivity (ICA100 edge 844)	rfMRI connectivity (ICA100 edge 844)
3273	rfMRI connectivity (ICA100 edge 845)	rfMRI connectivity (ICA100 edge 845)
3274	rfMRI connectivity (ICA100 edge 846)	rfMRI connectivity (ICA100 edge 846)
3275	rfMRI connectivity (ICA100 edge 847)	rfMRI connectivity (ICA100 edge 847)
3276	rfMRI connectivity (ICA100 edge 848)	rfMRI connectivity (ICA100 edge 848)
3277	rfMRI connectivity (ICA100 edge 849)	rfMRI connectivity (ICA100 edge 849)
3278	rfMRI connectivity (ICA100 edge 850)	rfMRI connectivity (ICA100 edge 850)
3279	rfMRI connectivity (ICA100 edge 851)	rfMRI connectivity (ICA100 edge 851)
3280	rfMRI connectivity (ICA100 edge 852)	rfMRI connectivity (ICA100 edge 852)
3281	rfMRI connectivity (ICA100 edge 853)	rfMRI connectivity (ICA100 edge 853)
3282	rfMRI connectivity (ICA100 edge 854)	rfMRI connectivity (ICA100 edge 854)
3283	rfMRI connectivity (ICA100 edge 855)	rfMRI connectivity (ICA100 edge 855)
3284	rfMRI connectivity (ICA100 edge 856)	rfMRI connectivity (ICA100 edge 856)
3285	rfMRI connectivity (ICA100 edge 857)	rfMRI connectivity (ICA100 edge 857)
3286	rfMRI connectivity (ICA100 edge 858)	rfMRI connectivity (ICA100 edge 858)
3287	rfMRI connectivity (ICA100 edge 859)	rfMRI connectivity (ICA100 edge 859)
3288	rfMRI connectivity (ICA100 edge 860)	rfMRI connectivity (ICA100 edge 860)
3289	rfMRI connectivity (ICA100 edge 861)	rfMRI connectivity (ICA100 edge 861)
3290	rfMRI connectivity (ICA100 edge 862)	rfMRI connectivity (ICA100 edge 862)
3291	rfMRI connectivity (ICA100 edge 863)	rfMRI connectivity (ICA100 edge 863)
3292	rfMRI connectivity (ICA100 edge 864)	rfMRI connectivity (ICA100 edge 864)
3293	rfMRI connectivity (ICA100 edge 865)	rfMRI connectivity (ICA100 edge 865)
3294	rfMRI connectivity (ICA100 edge 866)	rfMRI connectivity (ICA100 edge 866)
3295	rfMRI connectivity (ICA100 edge 867)	rfMRI connectivity (ICA100 edge 867)
3296	rfMRI connectivity (ICA100 edge 868)	rfMRI connectivity (ICA100 edge 868)
3297	rfMRI connectivity (ICA100 edge 869)	rfMRI connectivity (ICA100 edge 869)
3298	rfMRI connectivity (ICA100 edge 870)	rfMRI connectivity (ICA100 edge 870)
3299	rfMRI connectivity (ICA100 edge 871)	rfMRI connectivity (ICA100 edge 871)
3300	rfMRI connectivity (ICA100 edge 872)	rfMRI connectivity (ICA100 edge 872)
3301	rfMRI connectivity (ICA100 edge 873)	rfMRI connectivity (ICA100 edge 873)
3302	rfMRI connectivity (ICA100 edge 874)	rfMRI connectivity (ICA100 edge 874)
3303	rfMRI connectivity (ICA100 edge 875)	rfMRI connectivity (ICA100 edge 875)
3304	rfMRI connectivity (ICA100 edge 876)	rfMRI connectivity (ICA100 edge 876)

IDP number	IDP short name	IDP description
3305	rfMRI connectivity (ICA100 edge 877)	rfMRI connectivity (ICA100 edge 877)
3306	rfMRI connectivity (ICA100 edge 878)	rfMRI connectivity (ICA100 edge 878)
3307	rfMRI connectivity (ICA100 edge 879)	rfMRI connectivity (ICA100 edge 879)
3308	rfMRI connectivity (ICA100 edge 880)	rfMRI connectivity (ICA100 edge 880)
3309	rfMRI connectivity (ICA100 edge 881)	rfMRI connectivity (ICA100 edge 881)
3310	rfMRI connectivity (ICA100 edge 882)	rfMRI connectivity (ICA100 edge 882)
3311	rfMRI connectivity (ICA100 edge 883)	rfMRI connectivity (ICA100 edge 883)
3312	rfMRI connectivity (ICA100 edge 884)	rfMRI connectivity (ICA100 edge 884)
3313	rfMRI connectivity (ICA100 edge 885)	rfMRI connectivity (ICA100 edge 885)
3314	rfMRI connectivity (ICA100 edge 886)	rfMRI connectivity (ICA100 edge 886)
3315	rfMRI connectivity (ICA100 edge 887)	rfMRI connectivity (ICA100 edge 887)
3316	rfMRI connectivity (ICA100 edge 888)	rfMRI connectivity (ICA100 edge 888)
3317	rfMRI connectivity (ICA100 edge 889)	rfMRI connectivity (ICA100 edge 889)
3318	rfMRI connectivity (ICA100 edge 890)	rfMRI connectivity (ICA100 edge 890)
3319	rfMRI connectivity (ICA100 edge 891)	rfMRI connectivity (ICA100 edge 891)
3320	rfMRI connectivity (ICA100 edge 892)	rfMRI connectivity (ICA100 edge 892)
3321	rfMRI connectivity (ICA100 edge 893)	rfMRI connectivity (ICA100 edge 893)
3322	rfMRI connectivity (ICA100 edge 894)	rfMRI connectivity (ICA100 edge 894)
3323	rfMRI connectivity (ICA100 edge 895)	rfMRI connectivity (ICA100 edge 895)
3324	rfMRI connectivity (ICA100 edge 896)	rfMRI connectivity (ICA100 edge 896)
3325	rfMRI connectivity (ICA100 edge 897)	rfMRI connectivity (ICA100 edge 897)
3326	rfMRI connectivity (ICA100 edge 898)	rfMRI connectivity (ICA100 edge 898)
3327	rfMRI connectivity (ICA100 edge 899)	rfMRI connectivity (ICA100 edge 899)
3328	rfMRI connectivity (ICA100 edge 900)	rfMRI connectivity (ICA100 edge 900)
3329	rfMRI connectivity (ICA100 edge 901)	rfMRI connectivity (ICA100 edge 901)
3330	rfMRI connectivity (ICA100 edge 902)	rfMRI connectivity (ICA100 edge 902)
3331	rfMRI connectivity (ICA100 edge 903)	rfMRI connectivity (ICA100 edge 903)
3332	rfMRI connectivity (ICA100 edge 904)	rfMRI connectivity (ICA100 edge 904)
3333	rfMRI connectivity (ICA100 edge 905)	rfMRI connectivity (ICA100 edge 905)
3334	rfMRI connectivity (ICA100 edge 906)	rfMRI connectivity (ICA100 edge 906)
3335	rfMRI connectivity (ICA100 edge 907)	rfMRI connectivity (ICA100 edge 907)
3336	rfMRI connectivity (ICA100 edge 908)	rfMRI connectivity (ICA100 edge 908)
3337	rfMRI connectivity (ICA100 edge 909)	rfMRI connectivity (ICA100 edge 909)
3338	rfMRI connectivity (ICA100 edge 910)	rfMRI connectivity (ICA100 edge 910)
3339	rfMRI connectivity (ICA100 edge 911)	rfMRI connectivity (ICA100 edge 911)
3340	rfMRI connectivity (ICA100 edge 912)	rfMRI connectivity (ICA100 edge 912)
3341	rfMRI connectivity (ICA100 edge 913)	rfMRI connectivity (ICA100 edge 913)
3342	rfMRI connectivity (ICA100 edge 914)	rfMRI connectivity (ICA100 edge 914)
3343	rfMRI connectivity (ICA100 edge 915)	rfMRI connectivity (ICA100 edge 915)
3344	rfMRI connectivity (ICA100 edge 916)	rfMRI connectivity (ICA100 edge 916)
3345	rfMRI connectivity (ICA100 edge 917)	rfMRI connectivity (ICA100 edge 917)
3346	rfMRI connectivity (ICA100 edge 918)	rfMRI connectivity (ICA100 edge 918)
3347	rfMRI connectivity (ICA100 edge 919)	rfMRI connectivity (ICA100 edge 919)
3348	rfMRI connectivity (ICA100 edge 920)	rfMRI connectivity (ICA100 edge 920)
3349	rfMRI connectivity (ICA100 edge 921)	rfMRI connectivity (ICA100 edge 921)
3350	rfMRI connectivity (ICA100 edge 922)	rfMRI connectivity (ICA100 edge 922)
3351	rfMRI connectivity (ICA100 edge 923)	rfMRI connectivity (ICA100 edge 923)
3352	rfMRI connectivity (ICA100 edge 924)	rfMRI connectivity (ICA100 edge 924)
3353	rfMRI connectivity (ICA100 edge 925)	rfMRI connectivity (ICA100 edge 925)
3354	rfMRI connectivity (ICA100 edge 926)	rfMRI connectivity (ICA100 edge 926)
3355	rfMRI connectivity (ICA100 edge 927)	rfMRI connectivity (ICA100 edge 927)
3356	rfMRI connectivity (ICA100 edge 928)	rfMRI connectivity (ICA100 edge 928)
3357	rfMRI connectivity (ICA100 edge 929)	rfMRI connectivity (ICA100 edge 929)
3358	rfMRI connectivity (ICA100 edge 930)	rfMRI connectivity (ICA100 edge 930)
3359	rfMRI connectivity (ICA100 edge 931)	rfMRI connectivity (ICA100 edge 931)
3360	rfMRI connectivity (ICA100 edge 932)	rfMRI connectivity (ICA100 edge 932)
3361	rfMRI connectivity (ICA100 edge 933)	rfMRI connectivity (ICA100 edge 933)

IDP number	IDP short name	IDP description
3362	rfMRI connectivity (ICA100 edge 934)	rfMRI connectivity (ICA100 edge 934)
3363	rfMRI connectivity (ICA100 edge 935)	rfMRI connectivity (ICA100 edge 935)
3364	rfMRI connectivity (ICA100 edge 936)	rfMRI connectivity (ICA100 edge 936)
3365	rfMRI connectivity (ICA100 edge 937)	rfMRI connectivity (ICA100 edge 937)
3366	rfMRI connectivity (ICA100 edge 938)	rfMRI connectivity (ICA100 edge 938)
3367	rfMRI connectivity (ICA100 edge 939)	rfMRI connectivity (ICA100 edge 939)
3368	rfMRI connectivity (ICA100 edge 940)	rfMRI connectivity (ICA100 edge 940)
3369	rfMRI connectivity (ICA100 edge 941)	rfMRI connectivity (ICA100 edge 941)
3370	rfMRI connectivity (ICA100 edge 942)	rfMRI connectivity (ICA100 edge 942)
3371	rfMRI connectivity (ICA100 edge 943)	rfMRI connectivity (ICA100 edge 943)
3372	rfMRI connectivity (ICA100 edge 944)	rfMRI connectivity (ICA100 edge 944)
3373	rfMRI connectivity (ICA100 edge 945)	rfMRI connectivity (ICA100 edge 945)
3374	rfMRI connectivity (ICA100 edge 946)	rfMRI connectivity (ICA100 edge 946)
3375	rfMRI connectivity (ICA100 edge 947)	rfMRI connectivity (ICA100 edge 947)
3376	rfMRI connectivity (ICA100 edge 948)	rfMRI connectivity (ICA100 edge 948)
3377	rfMRI connectivity (ICA100 edge 949)	rfMRI connectivity (ICA100 edge 949)
3378	rfMRI connectivity (ICA100 edge 950)	rfMRI connectivity (ICA100 edge 950)
3379	rfMRI connectivity (ICA100 edge 951)	rfMRI connectivity (ICA100 edge 951)
3380	rfMRI connectivity (ICA100 edge 952)	rfMRI connectivity (ICA100 edge 952)
3381	rfMRI connectivity (ICA100 edge 953)	rfMRI connectivity (ICA100 edge 953)
3382	rfMRI connectivity (ICA100 edge 954)	rfMRI connectivity (ICA100 edge 954)
3383	rfMRI connectivity (ICA100 edge 955)	rfMRI connectivity (ICA100 edge 955)
3384	rfMRI connectivity (ICA100 edge 956)	rfMRI connectivity (ICA100 edge 956)
3385	rfMRI connectivity (ICA100 edge 957)	rfMRI connectivity (ICA100 edge 957)
3386	rfMRI connectivity (ICA100 edge 958)	rfMRI connectivity (ICA100 edge 958)
3387	rfMRI connectivity (ICA100 edge 959)	rfMRI connectivity (ICA100 edge 959)
3388	rfMRI connectivity (ICA100 edge 960)	rfMRI connectivity (ICA100 edge 960)
3389	rfMRI connectivity (ICA100 edge 961)	rfMRI connectivity (ICA100 edge 961)
3390	rfMRI connectivity (ICA100 edge 962)	rfMRI connectivity (ICA100 edge 962)
3391	rfMRI connectivity (ICA100 edge 963)	rfMRI connectivity (ICA100 edge 963)
3392	rfMRI connectivity (ICA100 edge 964)	rfMRI connectivity (ICA100 edge 964)
3393	rfMRI connectivity (ICA100 edge 965)	rfMRI connectivity (ICA100 edge 965)
3394	rfMRI connectivity (ICA100 edge 966)	rfMRI connectivity (ICA100 edge 966)
3395	rfMRI connectivity (ICA100 edge 967)	rfMRI connectivity (ICA100 edge 967)
3396	rfMRI connectivity (ICA100 edge 968)	rfMRI connectivity (ICA100 edge 968)
3397	rfMRI connectivity (ICA100 edge 969)	rfMRI connectivity (ICA100 edge 969)
3398	rfMRI connectivity (ICA100 edge 970)	rfMRI connectivity (ICA100 edge 970)
3399	rfMRI connectivity (ICA100 edge 971)	rfMRI connectivity (ICA100 edge 971)
3400	rfMRI connectivity (ICA100 edge 972)	rfMRI connectivity (ICA100 edge 972)
3401	rfMRI connectivity (ICA100 edge 973)	rfMRI connectivity (ICA100 edge 973)
3402	rfMRI connectivity (ICA100 edge 974)	rfMRI connectivity (ICA100 edge 974)
3403	rfMRI connectivity (ICA100 edge 975)	rfMRI connectivity (ICA100 edge 975)
3404	rfMRI connectivity (ICA100 edge 976)	rfMRI connectivity (ICA100 edge 976)
3405	rfMRI connectivity (ICA100 edge 977)	rfMRI connectivity (ICA100 edge 977)
3406	rfMRI connectivity (ICA100 edge 978)	rfMRI connectivity (ICA100 edge 978)
3407	rfMRI connectivity (ICA100 edge 979)	rfMRI connectivity (ICA100 edge 979)
3408	rfMRI connectivity (ICA100 edge 980)	rfMRI connectivity (ICA100 edge 980)
3409	rfMRI connectivity (ICA100 edge 981)	rfMRI connectivity (ICA100 edge 981)
3410	rfMRI connectivity (ICA100 edge 982)	rfMRI connectivity (ICA100 edge 982)
3411	rfMRI connectivity (ICA100 edge 983)	rfMRI connectivity (ICA100 edge 983)
3412	rfMRI connectivity (ICA100 edge 984)	rfMRI connectivity (ICA100 edge 984)
3413	rfMRI connectivity (ICA100 edge 985)	rfMRI connectivity (ICA100 edge 985)
3414	rfMRI connectivity (ICA100 edge 986)	rfMRI connectivity (ICA100 edge 986)
3415	rfMRI connectivity (ICA100 edge 987)	rfMRI connectivity (ICA100 edge 987)
3416	rfMRI connectivity (ICA100 edge 988)	rfMRI connectivity (ICA100 edge 988)
3417	rfMRI connectivity (ICA100 edge 989)	rfMRI connectivity (ICA100 edge 989)
3418	rfMRI connectivity (ICA100 edge 990)	rfMRI connectivity (ICA100 edge 990)

IDP number	IDP short name	IDP description
3419	rfMRI connectivity (ICA100 edge 991)	rfMRI connectivity (ICA100 edge 991)
3420	rfMRI connectivity (ICA100 edge 992)	rfMRI connectivity (ICA100 edge 992)
3421	rfMRI connectivity (ICA100 edge 993)	rfMRI connectivity (ICA100 edge 993)
3422	rfMRI connectivity (ICA100 edge 994)	rfMRI connectivity (ICA100 edge 994)
3423	rfMRI connectivity (ICA100 edge 995)	rfMRI connectivity (ICA100 edge 995)
3424	rfMRI connectivity (ICA100 edge 996)	rfMRI connectivity (ICA100 edge 996)
3425	rfMRI connectivity (ICA100 edge 997)	rfMRI connectivity (ICA100 edge 997)
3426	rfMRI connectivity (ICA100 edge 998)	rfMRI connectivity (ICA100 edge 998)
3427	rfMRI connectivity (ICA100 edge 999)	rfMRI connectivity (ICA100 edge 999)
3428	rfMRI connectivity (ICA100 edge 1000)	rfMRI connectivity (ICA100 edge 1000)
3429	rfMRI connectivity (ICA100 edge 1001)	rfMRI connectivity (ICA100 edge 1001)
3430	rfMRI connectivity (ICA100 edge 1002)	rfMRI connectivity (ICA100 edge 1002)
3431	rfMRI connectivity (ICA100 edge 1003)	rfMRI connectivity (ICA100 edge 1003)
3432	rfMRI connectivity (ICA100 edge 1004)	rfMRI connectivity (ICA100 edge 1004)
3433	rfMRI connectivity (ICA100 edge 1005)	rfMRI connectivity (ICA100 edge 1005)
3434	rfMRI connectivity (ICA100 edge 1006)	rfMRI connectivity (ICA100 edge 1006)
3435	rfMRI connectivity (ICA100 edge 1007)	rfMRI connectivity (ICA100 edge 1007)
3436	rfMRI connectivity (ICA100 edge 1008)	rfMRI connectivity (ICA100 edge 1008)
3437	rfMRI connectivity (ICA100 edge 1009)	rfMRI connectivity (ICA100 edge 1009)
3438	rfMRI connectivity (ICA100 edge 1010)	rfMRI connectivity (ICA100 edge 1010)
3439	rfMRI connectivity (ICA100 edge 1011)	rfMRI connectivity (ICA100 edge 1011)
3440	rfMRI connectivity (ICA100 edge 1012)	rfMRI connectivity (ICA100 edge 1012)
3441	rfMRI connectivity (ICA100 edge 1013)	rfMRI connectivity (ICA100 edge 1013)
3442	rfMRI connectivity (ICA100 edge 1014)	rfMRI connectivity (ICA100 edge 1014)
3443	rfMRI connectivity (ICA100 edge 1015)	rfMRI connectivity (ICA100 edge 1015)
3444	rfMRI connectivity (ICA100 edge 1016)	rfMRI connectivity (ICA100 edge 1016)
3445	rfMRI connectivity (ICA100 edge 1017)	rfMRI connectivity (ICA100 edge 1017)
3446	rfMRI connectivity (ICA100 edge 1018)	rfMRI connectivity (ICA100 edge 1018)
3447	rfMRI connectivity (ICA100 edge 1019)	rfMRI connectivity (ICA100 edge 1019)
3448	rfMRI connectivity (ICA100 edge 1020)	rfMRI connectivity (ICA100 edge 1020)
3449	rfMRI connectivity (ICA100 edge 1021)	rfMRI connectivity (ICA100 edge 1021)
3450	rfMRI connectivity (ICA100 edge 1022)	rfMRI connectivity (ICA100 edge 1022)
3451	rfMRI connectivity (ICA100 edge 1023)	rfMRI connectivity (ICA100 edge 1023)
3452	rfMRI connectivity (ICA100 edge 1024)	rfMRI connectivity (ICA100 edge 1024)
3453	rfMRI connectivity (ICA100 edge 1025)	rfMRI connectivity (ICA100 edge 1025)
3454	rfMRI connectivity (ICA100 edge 1026)	rfMRI connectivity (ICA100 edge 1026)
3455	rfMRI connectivity (ICA100 edge 1027)	rfMRI connectivity (ICA100 edge 1027)
3456	rfMRI connectivity (ICA100 edge 1028)	rfMRI connectivity (ICA100 edge 1028)
3457	rfMRI connectivity (ICA100 edge 1029)	rfMRI connectivity (ICA100 edge 1029)
3458	rfMRI connectivity (ICA100 edge 1030)	rfMRI connectivity (ICA100 edge 1030)
3459	rfMRI connectivity (ICA100 edge 1031)	rfMRI connectivity (ICA100 edge 1031)
3460	rfMRI connectivity (ICA100 edge 1032)	rfMRI connectivity (ICA100 edge 1032)
3461	rfMRI connectivity (ICA100 edge 1033)	rfMRI connectivity (ICA100 edge 1033)
3462	rfMRI connectivity (ICA100 edge 1034)	rfMRI connectivity (ICA100 edge 1034)
3463	rfMRI connectivity (ICA100 edge 1035)	rfMRI connectivity (ICA100 edge 1035)
3464	rfMRI connectivity (ICA100 edge 1036)	rfMRI connectivity (ICA100 edge 1036)
3465	rfMRI connectivity (ICA100 edge 1037)	rfMRI connectivity (ICA100 edge 1037)
3466	rfMRI connectivity (ICA100 edge 1038)	rfMRI connectivity (ICA100 edge 1038)
3467	rfMRI connectivity (ICA100 edge 1039)	rfMRI connectivity (ICA100 edge 1039)
3468	rfMRI connectivity (ICA100 edge 1040)	rfMRI connectivity (ICA100 edge 1040)
3469	rfMRI connectivity (ICA100 edge 1041)	rfMRI connectivity (ICA100 edge 1041)
3470	rfMRI connectivity (ICA100 edge 1042)	rfMRI connectivity (ICA100 edge 1042)
3471	rfMRI connectivity (ICA100 edge 1043)	rfMRI connectivity (ICA100 edge 1043)
3472	rfMRI connectivity (ICA100 edge 1044)	rfMRI connectivity (ICA100 edge 1044)
3473	rfMRI connectivity (ICA100 edge 1045)	rfMRI connectivity (ICA100 edge 1045)
3474	rfMRI connectivity (ICA100 edge 1046)	rfMRI connectivity (ICA100 edge 1046)
3475	rfMRI connectivity (ICA100 edge 1047)	rfMRI connectivity (ICA100 edge 1047)

IDP number	IDP short name	IDP description
3476	rfMRI connectivity (ICA100 edge 1048)	rfMRI connectivity (ICA100 edge 1048)
3477	rfMRI connectivity (ICA100 edge 1049)	rfMRI connectivity (ICA100 edge 1049)
3478	rfMRI connectivity (ICA100 edge 1050)	rfMRI connectivity (ICA100 edge 1050)
3479	rfMRI connectivity (ICA100 edge 1051)	rfMRI connectivity (ICA100 edge 1051)
3480	rfMRI connectivity (ICA100 edge 1052)	rfMRI connectivity (ICA100 edge 1052)
3481	rfMRI connectivity (ICA100 edge 1053)	rfMRI connectivity (ICA100 edge 1053)
3482	rfMRI connectivity (ICA100 edge 1054)	rfMRI connectivity (ICA100 edge 1054)
3483	rfMRI connectivity (ICA100 edge 1055)	rfMRI connectivity (ICA100 edge 1055)
3484	rfMRI connectivity (ICA100 edge 1056)	rfMRI connectivity (ICA100 edge 1056)
3485	rfMRI connectivity (ICA100 edge 1057)	rfMRI connectivity (ICA100 edge 1057)
3486	rfMRI connectivity (ICA100 edge 1058)	rfMRI connectivity (ICA100 edge 1058)
3487	rfMRI connectivity (ICA100 edge 1059)	rfMRI connectivity (ICA100 edge 1059)
3488	rfMRI connectivity (ICA100 edge 1060)	rfMRI connectivity (ICA100 edge 1060)
3489	rfMRI connectivity (ICA100 edge 1061)	rfMRI connectivity (ICA100 edge 1061)
3490	rfMRI connectivity (ICA100 edge 1062)	rfMRI connectivity (ICA100 edge 1062)
3491	rfMRI connectivity (ICA100 edge 1063)	rfMRI connectivity (ICA100 edge 1063)
3492	rfMRI connectivity (ICA100 edge 1064)	rfMRI connectivity (ICA100 edge 1064)
3493	rfMRI connectivity (ICA100 edge 1065)	rfMRI connectivity (ICA100 edge 1065)
3494	rfMRI connectivity (ICA100 edge 1066)	rfMRI connectivity (ICA100 edge 1066)
3495	rfMRI connectivity (ICA100 edge 1067)	rfMRI connectivity (ICA100 edge 1067)
3496	rfMRI connectivity (ICA100 edge 1068)	rfMRI connectivity (ICA100 edge 1068)
3497	rfMRI connectivity (ICA100 edge 1069)	rfMRI connectivity (ICA100 edge 1069)
3498	rfMRI connectivity (ICA100 edge 1070)	rfMRI connectivity (ICA100 edge 1070)
3499	rfMRI connectivity (ICA100 edge 1071)	rfMRI connectivity (ICA100 edge 1071)
3500	rfMRI connectivity (ICA100 edge 1072)	rfMRI connectivity (ICA100 edge 1072)
3501	rfMRI connectivity (ICA100 edge 1073)	rfMRI connectivity (ICA100 edge 1073)
3502	rfMRI connectivity (ICA100 edge 1074)	rfMRI connectivity (ICA100 edge 1074)
3503	rfMRI connectivity (ICA100 edge 1075)	rfMRI connectivity (ICA100 edge 1075)
3504	rfMRI connectivity (ICA100 edge 1076)	rfMRI connectivity (ICA100 edge 1076)
3505	rfMRI connectivity (ICA100 edge 1077)	rfMRI connectivity (ICA100 edge 1077)
3506	rfMRI connectivity (ICA100 edge 1078)	rfMRI connectivity (ICA100 edge 1078)
3507	rfMRI connectivity (ICA100 edge 1079)	rfMRI connectivity (ICA100 edge 1079)
3508	rfMRI connectivity (ICA100 edge 1080)	rfMRI connectivity (ICA100 edge 1080)
3509	rfMRI connectivity (ICA100 edge 1081)	rfMRI connectivity (ICA100 edge 1081)
3510	rfMRI connectivity (ICA100 edge 1082)	rfMRI connectivity (ICA100 edge 1082)
3511	rfMRI connectivity (ICA100 edge 1083)	rfMRI connectivity (ICA100 edge 1083)
3512	rfMRI connectivity (ICA100 edge 1084)	rfMRI connectivity (ICA100 edge 1084)
3513	rfMRI connectivity (ICA100 edge 1085)	rfMRI connectivity (ICA100 edge 1085)
3514	rfMRI connectivity (ICA100 edge 1086)	rfMRI connectivity (ICA100 edge 1086)
3515	rfMRI connectivity (ICA100 edge 1087)	rfMRI connectivity (ICA100 edge 1087)
3516	rfMRI connectivity (ICA100 edge 1088)	rfMRI connectivity (ICA100 edge 1088)
3517	rfMRI connectivity (ICA100 edge 1089)	rfMRI connectivity (ICA100 edge 1089)
3518	rfMRI connectivity (ICA100 edge 1090)	rfMRI connectivity (ICA100 edge 1090)
3519	rfMRI connectivity (ICA100 edge 1091)	rfMRI connectivity (ICA100 edge 1091)
3520	rfMRI connectivity (ICA100 edge 1092)	rfMRI connectivity (ICA100 edge 1092)
3521	rfMRI connectivity (ICA100 edge 1093)	rfMRI connectivity (ICA100 edge 1093)
3522	rfMRI connectivity (ICA100 edge 1094)	rfMRI connectivity (ICA100 edge 1094)
3523	rfMRI connectivity (ICA100 edge 1095)	rfMRI connectivity (ICA100 edge 1095)
3524	rfMRI connectivity (ICA100 edge 1096)	rfMRI connectivity (ICA100 edge 1096)
3525	rfMRI connectivity (ICA100 edge 1097)	rfMRI connectivity (ICA100 edge 1097)
3526	rfMRI connectivity (ICA100 edge 1098)	rfMRI connectivity (ICA100 edge 1098)
3527	rfMRI connectivity (ICA100 edge 1099)	rfMRI connectivity (ICA100 edge 1099)
3528	rfMRI connectivity (ICA100 edge 1100)	rfMRI connectivity (ICA100 edge 1100)
3529	rfMRI connectivity (ICA100 edge 1101)	rfMRI connectivity (ICA100 edge 1101)
3530	rfMRI connectivity (ICA100 edge 1102)	rfMRI connectivity (ICA100 edge 1102)
3531	rfMRI connectivity (ICA100 edge 1103)	rfMRI connectivity (ICA100 edge 1103)
3532	rfMRI connectivity (ICA100 edge 1104)	rfMRI connectivity (ICA100 edge 1104)

IDP number	IDP short name	IDP description
3533	rfMRI connectivity (ICA100 edge 1105)	rfMRI connectivity (ICA100 edge 1105)
3534	rfMRI connectivity (ICA100 edge 1106)	rfMRI connectivity (ICA100 edge 1106)
3535	rfMRI connectivity (ICA100 edge 1107)	rfMRI connectivity (ICA100 edge 1107)
3536	rfMRI connectivity (ICA100 edge 1108)	rfMRI connectivity (ICA100 edge 1108)
3537	rfMRI connectivity (ICA100 edge 1109)	rfMRI connectivity (ICA100 edge 1109)
3538	rfMRI connectivity (ICA100 edge 1110)	rfMRI connectivity (ICA100 edge 1110)
3539	rfMRI connectivity (ICA100 edge 1111)	rfMRI connectivity (ICA100 edge 1111)
3540	rfMRI connectivity (ICA100 edge 1112)	rfMRI connectivity (ICA100 edge 1112)
3541	rfMRI connectivity (ICA100 edge 1113)	rfMRI connectivity (ICA100 edge 1113)
3542	rfMRI connectivity (ICA100 edge 1114)	rfMRI connectivity (ICA100 edge 1114)
3543	rfMRI connectivity (ICA100 edge 1115)	rfMRI connectivity (ICA100 edge 1115)
3544	rfMRI connectivity (ICA100 edge 1116)	rfMRI connectivity (ICA100 edge 1116)
3545	rfMRI connectivity (ICA100 edge 1117)	rfMRI connectivity (ICA100 edge 1117)
3546	rfMRI connectivity (ICA100 edge 1118)	rfMRI connectivity (ICA100 edge 1118)
3547	rfMRI connectivity (ICA100 edge 1119)	rfMRI connectivity (ICA100 edge 1119)
3548	rfMRI connectivity (ICA100 edge 1120)	rfMRI connectivity (ICA100 edge 1120)
3549	rfMRI connectivity (ICA100 edge 1121)	rfMRI connectivity (ICA100 edge 1121)
3550	rfMRI connectivity (ICA100 edge 1122)	rfMRI connectivity (ICA100 edge 1122)
3551	rfMRI connectivity (ICA100 edge 1123)	rfMRI connectivity (ICA100 edge 1123)
3552	rfMRI connectivity (ICA100 edge 1124)	rfMRI connectivity (ICA100 edge 1124)
3553	rfMRI connectivity (ICA100 edge 1125)	rfMRI connectivity (ICA100 edge 1125)
3554	rfMRI connectivity (ICA100 edge 1126)	rfMRI connectivity (ICA100 edge 1126)
3555	rfMRI connectivity (ICA100 edge 1127)	rfMRI connectivity (ICA100 edge 1127)
3556	rfMRI connectivity (ICA100 edge 1128)	rfMRI connectivity (ICA100 edge 1128)
3557	rfMRI connectivity (ICA100 edge 1129)	rfMRI connectivity (ICA100 edge 1129)
3558	rfMRI connectivity (ICA100 edge 1130)	rfMRI connectivity (ICA100 edge 1130)
3559	rfMRI connectivity (ICA100 edge 1131)	rfMRI connectivity (ICA100 edge 1131)
3560	rfMRI connectivity (ICA100 edge 1132)	rfMRI connectivity (ICA100 edge 1132)
3561	rfMRI connectivity (ICA100 edge 1133)	rfMRI connectivity (ICA100 edge 1133)
3562	rfMRI connectivity (ICA100 edge 1134)	rfMRI connectivity (ICA100 edge 1134)
3563	rfMRI connectivity (ICA100 edge 1135)	rfMRI connectivity (ICA100 edge 1135)
3564	rfMRI connectivity (ICA100 edge 1136)	rfMRI connectivity (ICA100 edge 1136)
3565	rfMRI connectivity (ICA100 edge 1137)	rfMRI connectivity (ICA100 edge 1137)
3566	rfMRI connectivity (ICA100 edge 1138)	rfMRI connectivity (ICA100 edge 1138)
3567	rfMRI connectivity (ICA100 edge 1139)	rfMRI connectivity (ICA100 edge 1139)
3568	rfMRI connectivity (ICA100 edge 1140)	rfMRI connectivity (ICA100 edge 1140)
3569	rfMRI connectivity (ICA100 edge 1141)	rfMRI connectivity (ICA100 edge 1141)
3570	rfMRI connectivity (ICA100 edge 1142)	rfMRI connectivity (ICA100 edge 1142)
3571	rfMRI connectivity (ICA100 edge 1143)	rfMRI connectivity (ICA100 edge 1143)
3572	rfMRI connectivity (ICA100 edge 1144)	rfMRI connectivity (ICA100 edge 1144)
3573	rfMRI connectivity (ICA100 edge 1145)	rfMRI connectivity (ICA100 edge 1145)
3574	rfMRI connectivity (ICA100 edge 1146)	rfMRI connectivity (ICA100 edge 1146)
3575	rfMRI connectivity (ICA100 edge 1147)	rfMRI connectivity (ICA100 edge 1147)
3576	rfMRI connectivity (ICA100 edge 1148)	rfMRI connectivity (ICA100 edge 1148)
3577	rfMRI connectivity (ICA100 edge 1149)	rfMRI connectivity (ICA100 edge 1149)
3578	rfMRI connectivity (ICA100 edge 1150)	rfMRI connectivity (ICA100 edge 1150)
3579	rfMRI connectivity (ICA100 edge 1151)	rfMRI connectivity (ICA100 edge 1151)
3580	rfMRI connectivity (ICA100 edge 1152)	rfMRI connectivity (ICA100 edge 1152)
3581	rfMRI connectivity (ICA100 edge 1153)	rfMRI connectivity (ICA100 edge 1153)
3582	rfMRI connectivity (ICA100 edge 1154)	rfMRI connectivity (ICA100 edge 1154)
3583	rfMRI connectivity (ICA100 edge 1155)	rfMRI connectivity (ICA100 edge 1155)
3584	rfMRI connectivity (ICA100 edge 1156)	rfMRI connectivity (ICA100 edge 1156)
3585	rfMRI connectivity (ICA100 edge 1157)	rfMRI connectivity (ICA100 edge 1157)
3586	rfMRI connectivity (ICA100 edge 1158)	rfMRI connectivity (ICA100 edge 1158)
3587	rfMRI connectivity (ICA100 edge 1159)	rfMRI connectivity (ICA100 edge 1159)
3588	rfMRI connectivity (ICA100 edge 1160)	rfMRI connectivity (ICA100 edge 1160)
3589	rfMRI connectivity (ICA100 edge 1161)	rfMRI connectivity (ICA100 edge 1161)

IDP number	IDP short name	IDP description
3590	rfMRI connectivity (ICA100 edge 1162)	rfMRI connectivity (ICA100 edge 1162)
3591	rfMRI connectivity (ICA100 edge 1163)	rfMRI connectivity (ICA100 edge 1163)
3592	rfMRI connectivity (ICA100 edge 1164)	rfMRI connectivity (ICA100 edge 1164)
3593	rfMRI connectivity (ICA100 edge 1165)	rfMRI connectivity (ICA100 edge 1165)
3594	rfMRI connectivity (ICA100 edge 1166)	rfMRI connectivity (ICA100 edge 1166)
3595	rfMRI connectivity (ICA100 edge 1167)	rfMRI connectivity (ICA100 edge 1167)
3596	rfMRI connectivity (ICA100 edge 1168)	rfMRI connectivity (ICA100 edge 1168)
3597	rfMRI connectivity (ICA100 edge 1169)	rfMRI connectivity (ICA100 edge 1169)
3598	rfMRI connectivity (ICA100 edge 1170)	rfMRI connectivity (ICA100 edge 1170)
3599	rfMRI connectivity (ICA100 edge 1171)	rfMRI connectivity (ICA100 edge 1171)
3600	rfMRI connectivity (ICA100 edge 1172)	rfMRI connectivity (ICA100 edge 1172)
3601	rfMRI connectivity (ICA100 edge 1173)	rfMRI connectivity (ICA100 edge 1173)
3602	rfMRI connectivity (ICA100 edge 1174)	rfMRI connectivity (ICA100 edge 1174)
3603	rfMRI connectivity (ICA100 edge 1175)	rfMRI connectivity (ICA100 edge 1175)
3604	rfMRI connectivity (ICA100 edge 1176)	rfMRI connectivity (ICA100 edge 1176)
3605	rfMRI connectivity (ICA100 edge 1177)	rfMRI connectivity (ICA100 edge 1177)
3606	rfMRI connectivity (ICA100 edge 1178)	rfMRI connectivity (ICA100 edge 1178)
3607	rfMRI connectivity (ICA100 edge 1179)	rfMRI connectivity (ICA100 edge 1179)
3608	rfMRI connectivity (ICA100 edge 1180)	rfMRI connectivity (ICA100 edge 1180)
3609	rfMRI connectivity (ICA100 edge 1181)	rfMRI connectivity (ICA100 edge 1181)
3610	rfMRI connectivity (ICA100 edge 1182)	rfMRI connectivity (ICA100 edge 1182)
3611	rfMRI connectivity (ICA100 edge 1183)	rfMRI connectivity (ICA100 edge 1183)
3612	rfMRI connectivity (ICA100 edge 1184)	rfMRI connectivity (ICA100 edge 1184)
3613	rfMRI connectivity (ICA100 edge 1185)	rfMRI connectivity (ICA100 edge 1185)
3614	rfMRI connectivity (ICA100 edge 1186)	rfMRI connectivity (ICA100 edge 1186)
3615	rfMRI connectivity (ICA100 edge 1187)	rfMRI connectivity (ICA100 edge 1187)
3616	rfMRI connectivity (ICA100 edge 1188)	rfMRI connectivity (ICA100 edge 1188)
3617	rfMRI connectivity (ICA100 edge 1189)	rfMRI connectivity (ICA100 edge 1189)
3618	rfMRI connectivity (ICA100 edge 1190)	rfMRI connectivity (ICA100 edge 1190)
3619	rfMRI connectivity (ICA100 edge 1191)	rfMRI connectivity (ICA100 edge 1191)
3620	rfMRI connectivity (ICA100 edge 1192)	rfMRI connectivity (ICA100 edge 1192)
3621	rfMRI connectivity (ICA100 edge 1193)	rfMRI connectivity (ICA100 edge 1193)
3622	rfMRI connectivity (ICA100 edge 1194)	rfMRI connectivity (ICA100 edge 1194)
3623	rfMRI connectivity (ICA100 edge 1195)	rfMRI connectivity (ICA100 edge 1195)
3624	rfMRI connectivity (ICA100 edge 1196)	rfMRI connectivity (ICA100 edge 1196)
3625	rfMRI connectivity (ICA100 edge 1197)	rfMRI connectivity (ICA100 edge 1197)
3626	rfMRI connectivity (ICA100 edge 1198)	rfMRI connectivity (ICA100 edge 1198)
3627	rfMRI connectivity (ICA100 edge 1199)	rfMRI connectivity (ICA100 edge 1199)
3628	rfMRI connectivity (ICA100 edge 1200)	rfMRI connectivity (ICA100 edge 1200)
3629	rfMRI connectivity (ICA100 edge 1201)	rfMRI connectivity (ICA100 edge 1201)
3630	rfMRI connectivity (ICA100 edge 1202)	rfMRI connectivity (ICA100 edge 1202)
3631	rfMRI connectivity (ICA100 edge 1203)	rfMRI connectivity (ICA100 edge 1203)
3632	rfMRI connectivity (ICA100 edge 1204)	rfMRI connectivity (ICA100 edge 1204)
3633	rfMRI connectivity (ICA100 edge 1205)	rfMRI connectivity (ICA100 edge 1205)
3634	rfMRI connectivity (ICA100 edge 1206)	rfMRI connectivity (ICA100 edge 1206)
3635	rfMRI connectivity (ICA100 edge 1207)	rfMRI connectivity (ICA100 edge 1207)
3636	rfMRI connectivity (ICA100 edge 1208)	rfMRI connectivity (ICA100 edge 1208)
3637	rfMRI connectivity (ICA100 edge 1209)	rfMRI connectivity (ICA100 edge 1209)
3638	rfMRI connectivity (ICA100 edge 1210)	rfMRI connectivity (ICA100 edge 1210)
3639	rfMRI connectivity (ICA100 edge 1211)	rfMRI connectivity (ICA100 edge 1211)
3640	rfMRI connectivity (ICA100 edge 1212)	rfMRI connectivity (ICA100 edge 1212)
3641	rfMRI connectivity (ICA100 edge 1213)	rfMRI connectivity (ICA100 edge 1213)
3642	rfMRI connectivity (ICA100 edge 1214)	rfMRI connectivity (ICA100 edge 1214)
3643	rfMRI connectivity (ICA100 edge 1215)	rfMRI connectivity (ICA100 edge 1215)
3644	rfMRI connectivity (ICA100 edge 1216)	rfMRI connectivity (ICA100 edge 1216)
3645	rfMRI connectivity (ICA100 edge 1217)	rfMRI connectivity (ICA100 edge 1217)
3646	rfMRI connectivity (ICA100 edge 1218)	rfMRI connectivity (ICA100 edge 1218)

IDP number	IDP short name	IDP description
3647	rfMRI connectivity (ICA100 edge 1219)	rfMRI connectivity (ICA100 edge 1219)
3648	rfMRI connectivity (ICA100 edge 1220)	rfMRI connectivity (ICA100 edge 1220)
3649	rfMRI connectivity (ICA100 edge 1221)	rfMRI connectivity (ICA100 edge 1221)
3650	rfMRI connectivity (ICA100 edge 1222)	rfMRI connectivity (ICA100 edge 1222)
3651	rfMRI connectivity (ICA100 edge 1223)	rfMRI connectivity (ICA100 edge 1223)
3652	rfMRI connectivity (ICA100 edge 1224)	rfMRI connectivity (ICA100 edge 1224)
3653	rfMRI connectivity (ICA100 edge 1225)	rfMRI connectivity (ICA100 edge 1225)
3654	rfMRI connectivity (ICA100 edge 1226)	rfMRI connectivity (ICA100 edge 1226)
3655	rfMRI connectivity (ICA100 edge 1227)	rfMRI connectivity (ICA100 edge 1227)
3656	rfMRI connectivity (ICA100 edge 1228)	rfMRI connectivity (ICA100 edge 1228)
3657	rfMRI connectivity (ICA100 edge 1229)	rfMRI connectivity (ICA100 edge 1229)
3658	rfMRI connectivity (ICA100 edge 1230)	rfMRI connectivity (ICA100 edge 1230)
3659	rfMRI connectivity (ICA100 edge 1231)	rfMRI connectivity (ICA100 edge 1231)
3660	rfMRI connectivity (ICA100 edge 1232)	rfMRI connectivity (ICA100 edge 1232)
3661	rfMRI connectivity (ICA100 edge 1233)	rfMRI connectivity (ICA100 edge 1233)
3662	rfMRI connectivity (ICA100 edge 1234)	rfMRI connectivity (ICA100 edge 1234)
3663	rfMRI connectivity (ICA100 edge 1235)	rfMRI connectivity (ICA100 edge 1235)
3664	rfMRI connectivity (ICA100 edge 1236)	rfMRI connectivity (ICA100 edge 1236)
3665	rfMRI connectivity (ICA100 edge 1237)	rfMRI connectivity (ICA100 edge 1237)
3666	rfMRI connectivity (ICA100 edge 1238)	rfMRI connectivity (ICA100 edge 1238)
3667	rfMRI connectivity (ICA100 edge 1239)	rfMRI connectivity (ICA100 edge 1239)
3668	rfMRI connectivity (ICA100 edge 1240)	rfMRI connectivity (ICA100 edge 1240)
3669	rfMRI connectivity (ICA100 edge 1241)	rfMRI connectivity (ICA100 edge 1241)
3670	rfMRI connectivity (ICA100 edge 1242)	rfMRI connectivity (ICA100 edge 1242)
3671	rfMRI connectivity (ICA100 edge 1243)	rfMRI connectivity (ICA100 edge 1243)
3672	rfMRI connectivity (ICA100 edge 1244)	rfMRI connectivity (ICA100 edge 1244)
3673	rfMRI connectivity (ICA100 edge 1245)	rfMRI connectivity (ICA100 edge 1245)
3674	rfMRI connectivity (ICA100 edge 1246)	rfMRI connectivity (ICA100 edge 1246)
3675	rfMRI connectivity (ICA100 edge 1247)	rfMRI connectivity (ICA100 edge 1247)
3676	rfMRI connectivity (ICA100 edge 1248)	rfMRI connectivity (ICA100 edge 1248)
3677	rfMRI connectivity (ICA100 edge 1249)	rfMRI connectivity (ICA100 edge 1249)
3678	rfMRI connectivity (ICA100 edge 1250)	rfMRI connectivity (ICA100 edge 1250)
3679	rfMRI connectivity (ICA100 edge 1251)	rfMRI connectivity (ICA100 edge 1251)
3680	rfMRI connectivity (ICA100 edge 1252)	rfMRI connectivity (ICA100 edge 1252)
3681	rfMRI connectivity (ICA100 edge 1253)	rfMRI connectivity (ICA100 edge 1253)
3682	rfMRI connectivity (ICA100 edge 1254)	rfMRI connectivity (ICA100 edge 1254)
3683	rfMRI connectivity (ICA100 edge 1255)	rfMRI connectivity (ICA100 edge 1255)
3684	rfMRI connectivity (ICA100 edge 1256)	rfMRI connectivity (ICA100 edge 1256)
3685	rfMRI connectivity (ICA100 edge 1257)	rfMRI connectivity (ICA100 edge 1257)
3686	rfMRI connectivity (ICA100 edge 1258)	rfMRI connectivity (ICA100 edge 1258)
3687	rfMRI connectivity (ICA100 edge 1259)	rfMRI connectivity (ICA100 edge 1259)
3688	rfMRI connectivity (ICA100 edge 1260)	rfMRI connectivity (ICA100 edge 1260)
3689	rfMRI connectivity (ICA100 edge 1261)	rfMRI connectivity (ICA100 edge 1261)
3690	rfMRI connectivity (ICA100 edge 1262)	rfMRI connectivity (ICA100 edge 1262)
3691	rfMRI connectivity (ICA100 edge 1263)	rfMRI connectivity (ICA100 edge 1263)
3692	rfMRI connectivity (ICA100 edge 1264)	rfMRI connectivity (ICA100 edge 1264)
3693	rfMRI connectivity (ICA100 edge 1265)	rfMRI connectivity (ICA100 edge 1265)
3694	rfMRI connectivity (ICA100 edge 1266)	rfMRI connectivity (ICA100 edge 1266)
3695	rfMRI connectivity (ICA100 edge 1267)	rfMRI connectivity (ICA100 edge 1267)
3696	rfMRI connectivity (ICA100 edge 1268)	rfMRI connectivity (ICA100 edge 1268)
3697	rfMRI connectivity (ICA100 edge 1269)	rfMRI connectivity (ICA100 edge 1269)
3698	rfMRI connectivity (ICA100 edge 1270)	rfMRI connectivity (ICA100 edge 1270)
3699	rfMRI connectivity (ICA100 edge 1271)	rfMRI connectivity (ICA100 edge 1271)
3700	rfMRI connectivity (ICA100 edge 1272)	rfMRI connectivity (ICA100 edge 1272)
3701	rfMRI connectivity (ICA100 edge 1273)	rfMRI connectivity (ICA100 edge 1273)
3702	rfMRI connectivity (ICA100 edge 1274)	rfMRI connectivity (ICA100 edge 1274)
3703	rfMRI connectivity (ICA100 edge 1275)	rfMRI connectivity (ICA100 edge 1275)

IDP number	IDP short name	IDP description
3704	rfMRI connectivity (ICA100 edge 1276)	rfMRI connectivity (ICA100 edge 1276)
3705	rfMRI connectivity (ICA100 edge 1277)	rfMRI connectivity (ICA100 edge 1277)
3706	rfMRI connectivity (ICA100 edge 1278)	rfMRI connectivity (ICA100 edge 1278)
3707	rfMRI connectivity (ICA100 edge 1279)	rfMRI connectivity (ICA100 edge 1279)
3708	rfMRI connectivity (ICA100 edge 1280)	rfMRI connectivity (ICA100 edge 1280)
3709	rfMRI connectivity (ICA100 edge 1281)	rfMRI connectivity (ICA100 edge 1281)
3710	rfMRI connectivity (ICA100 edge 1282)	rfMRI connectivity (ICA100 edge 1282)
3711	rfMRI connectivity (ICA100 edge 1283)	rfMRI connectivity (ICA100 edge 1283)
3712	rfMRI connectivity (ICA100 edge 1284)	rfMRI connectivity (ICA100 edge 1284)
3713	rfMRI connectivity (ICA100 edge 1285)	rfMRI connectivity (ICA100 edge 1285)
3714	rfMRI connectivity (ICA100 edge 1286)	rfMRI connectivity (ICA100 edge 1286)
3715	rfMRI connectivity (ICA100 edge 1287)	rfMRI connectivity (ICA100 edge 1287)
3716	rfMRI connectivity (ICA100 edge 1288)	rfMRI connectivity (ICA100 edge 1288)
3717	rfMRI connectivity (ICA100 edge 1289)	rfMRI connectivity (ICA100 edge 1289)
3718	rfMRI connectivity (ICA100 edge 1290)	rfMRI connectivity (ICA100 edge 1290)
3719	rfMRI connectivity (ICA100 edge 1291)	rfMRI connectivity (ICA100 edge 1291)
3720	rfMRI connectivity (ICA100 edge 1292)	rfMRI connectivity (ICA100 edge 1292)
3721	rfMRI connectivity (ICA100 edge 1293)	rfMRI connectivity (ICA100 edge 1293)
3722	rfMRI connectivity (ICA100 edge 1294)	rfMRI connectivity (ICA100 edge 1294)
3723	rfMRI connectivity (ICA100 edge 1295)	rfMRI connectivity (ICA100 edge 1295)
3724	rfMRI connectivity (ICA100 edge 1296)	rfMRI connectivity (ICA100 edge 1296)
3725	rfMRI connectivity (ICA100 edge 1297)	rfMRI connectivity (ICA100 edge 1297)
3726	rfMRI connectivity (ICA100 edge 1298)	rfMRI connectivity (ICA100 edge 1298)
3727	rfMRI connectivity (ICA100 edge 1299)	rfMRI connectivity (ICA100 edge 1299)
3728	rfMRI connectivity (ICA100 edge 1300)	rfMRI connectivity (ICA100 edge 1300)
3729	rfMRI connectivity (ICA100 edge 1301)	rfMRI connectivity (ICA100 edge 1301)
3730	rfMRI connectivity (ICA100 edge 1302)	rfMRI connectivity (ICA100 edge 1302)
3731	rfMRI connectivity (ICA100 edge 1303)	rfMRI connectivity (ICA100 edge 1303)
3732	rfMRI connectivity (ICA100 edge 1304)	rfMRI connectivity (ICA100 edge 1304)
3733	rfMRI connectivity (ICA100 edge 1305)	rfMRI connectivity (ICA100 edge 1305)
3734	rfMRI connectivity (ICA100 edge 1306)	rfMRI connectivity (ICA100 edge 1306)
3735	rfMRI connectivity (ICA100 edge 1307)	rfMRI connectivity (ICA100 edge 1307)
3736	rfMRI connectivity (ICA100 edge 1308)	rfMRI connectivity (ICA100 edge 1308)
3737	rfMRI connectivity (ICA100 edge 1309)	rfMRI connectivity (ICA100 edge 1309)
3738	rfMRI connectivity (ICA100 edge 1310)	rfMRI connectivity (ICA100 edge 1310)
3739	rfMRI connectivity (ICA100 edge 1311)	rfMRI connectivity (ICA100 edge 1311)
3740	rfMRI connectivity (ICA100 edge 1312)	rfMRI connectivity (ICA100 edge 1312)
3741	rfMRI connectivity (ICA100 edge 1313)	rfMRI connectivity (ICA100 edge 1313)
3742	rfMRI connectivity (ICA100 edge 1314)	rfMRI connectivity (ICA100 edge 1314)
3743	rfMRI connectivity (ICA100 edge 1315)	rfMRI connectivity (ICA100 edge 1315)
3744	rfMRI connectivity (ICA100 edge 1316)	rfMRI connectivity (ICA100 edge 1316)
3745	rfMRI connectivity (ICA100 edge 1317)	rfMRI connectivity (ICA100 edge 1317)
3746	rfMRI connectivity (ICA100 edge 1318)	rfMRI connectivity (ICA100 edge 1318)
3747	rfMRI connectivity (ICA100 edge 1319)	rfMRI connectivity (ICA100 edge 1319)
3748	rfMRI connectivity (ICA100 edge 1320)	rfMRI connectivity (ICA100 edge 1320)
3749	rfMRI connectivity (ICA100 edge 1321)	rfMRI connectivity (ICA100 edge 1321)
3750	rfMRI connectivity (ICA100 edge 1322)	rfMRI connectivity (ICA100 edge 1322)
3751	rfMRI connectivity (ICA100 edge 1323)	rfMRI connectivity (ICA100 edge 1323)
3752	rfMRI connectivity (ICA100 edge 1324)	rfMRI connectivity (ICA100 edge 1324)
3753	rfMRI connectivity (ICA100 edge 1325)	rfMRI connectivity (ICA100 edge 1325)
3754	rfMRI connectivity (ICA100 edge 1326)	rfMRI connectivity (ICA100 edge 1326)
3755	rfMRI connectivity (ICA100 edge 1327)	rfMRI connectivity (ICA100 edge 1327)
3756	rfMRI connectivity (ICA100 edge 1328)	rfMRI connectivity (ICA100 edge 1328)
3757	rfMRI connectivity (ICA100 edge 1329)	rfMRI connectivity (ICA100 edge 1329)
3758	rfMRI connectivity (ICA100 edge 1330)	rfMRI connectivity (ICA100 edge 1330)
3759	rfMRI connectivity (ICA100 edge 1331)	rfMRI connectivity (ICA100 edge 1331)
3760	rfMRI connectivity (ICA100 edge 1332)	rfMRI connectivity (ICA100 edge 1332)

IDP number	IDP short name	IDP description
3761	rfMRI connectivity (ICA100 edge 1333)	rfMRI connectivity (ICA100 edge 1333)
3762	rfMRI connectivity (ICA100 edge 1334)	rfMRI connectivity (ICA100 edge 1334)
3763	rfMRI connectivity (ICA100 edge 1335)	rfMRI connectivity (ICA100 edge 1335)
3764	rfMRI connectivity (ICA100 edge 1336)	rfMRI connectivity (ICA100 edge 1336)
3765	rfMRI connectivity (ICA100 edge 1337)	rfMRI connectivity (ICA100 edge 1337)
3766	rfMRI connectivity (ICA100 edge 1338)	rfMRI connectivity (ICA100 edge 1338)
3767	rfMRI connectivity (ICA100 edge 1339)	rfMRI connectivity (ICA100 edge 1339)
3768	rfMRI connectivity (ICA100 edge 1340)	rfMRI connectivity (ICA100 edge 1340)
3769	rfMRI connectivity (ICA100 edge 1341)	rfMRI connectivity (ICA100 edge 1341)
3770	rfMRI connectivity (ICA100 edge 1342)	rfMRI connectivity (ICA100 edge 1342)
3771	rfMRI connectivity (ICA100 edge 1343)	rfMRI connectivity (ICA100 edge 1343)
3772	rfMRI connectivity (ICA100 edge 1344)	rfMRI connectivity (ICA100 edge 1344)
3773	rfMRI connectivity (ICA100 edge 1345)	rfMRI connectivity (ICA100 edge 1345)
3774	rfMRI connectivity (ICA100 edge 1346)	rfMRI connectivity (ICA100 edge 1346)
3775	rfMRI connectivity (ICA100 edge 1347)	rfMRI connectivity (ICA100 edge 1347)
3776	rfMRI connectivity (ICA100 edge 1348)	rfMRI connectivity (ICA100 edge 1348)
3777	rfMRI connectivity (ICA100 edge 1349)	rfMRI connectivity (ICA100 edge 1349)
3778	rfMRI connectivity (ICA100 edge 1350)	rfMRI connectivity (ICA100 edge 1350)
3779	rfMRI connectivity (ICA100 edge 1351)	rfMRI connectivity (ICA100 edge 1351)
3780	rfMRI connectivity (ICA100 edge 1352)	rfMRI connectivity (ICA100 edge 1352)
3781	rfMRI connectivity (ICA100 edge 1353)	rfMRI connectivity (ICA100 edge 1353)
3782	rfMRI connectivity (ICA100 edge 1354)	rfMRI connectivity (ICA100 edge 1354)
3783	rfMRI connectivity (ICA100 edge 1355)	rfMRI connectivity (ICA100 edge 1355)
3784	rfMRI connectivity (ICA100 edge 1356)	rfMRI connectivity (ICA100 edge 1356)
3785	rfMRI connectivity (ICA100 edge 1357)	rfMRI connectivity (ICA100 edge 1357)
3786	rfMRI connectivity (ICA100 edge 1358)	rfMRI connectivity (ICA100 edge 1358)
3787	rfMRI connectivity (ICA100 edge 1359)	rfMRI connectivity (ICA100 edge 1359)
3788	rfMRI connectivity (ICA100 edge 1360)	rfMRI connectivity (ICA100 edge 1360)
3789	rfMRI connectivity (ICA100 edge 1361)	rfMRI connectivity (ICA100 edge 1361)
3790	rfMRI connectivity (ICA100 edge 1362)	rfMRI connectivity (ICA100 edge 1362)
3791	rfMRI connectivity (ICA100 edge 1363)	rfMRI connectivity (ICA100 edge 1363)
3792	rfMRI connectivity (ICA100 edge 1364)	rfMRI connectivity (ICA100 edge 1364)
3793	rfMRI connectivity (ICA100 edge 1365)	rfMRI connectivity (ICA100 edge 1365)
3794	rfMRI connectivity (ICA100 edge 1366)	rfMRI connectivity (ICA100 edge 1366)
3795	rfMRI connectivity (ICA100 edge 1367)	rfMRI connectivity (ICA100 edge 1367)
3796	rfMRI connectivity (ICA100 edge 1368)	rfMRI connectivity (ICA100 edge 1368)
3797	rfMRI connectivity (ICA100 edge 1369)	rfMRI connectivity (ICA100 edge 1369)
3798	rfMRI connectivity (ICA100 edge 1370)	rfMRI connectivity (ICA100 edge 1370)
3799	rfMRI connectivity (ICA100 edge 1371)	rfMRI connectivity (ICA100 edge 1371)
3800	rfMRI connectivity (ICA100 edge 1372)	rfMRI connectivity (ICA100 edge 1372)
3801	rfMRI connectivity (ICA100 edge 1373)	rfMRI connectivity (ICA100 edge 1373)
3802	rfMRI connectivity (ICA100 edge 1374)	rfMRI connectivity (ICA100 edge 1374)
3803	rfMRI connectivity (ICA100 edge 1375)	rfMRI connectivity (ICA100 edge 1375)
3804	rfMRI connectivity (ICA100 edge 1376)	rfMRI connectivity (ICA100 edge 1376)
3805	rfMRI connectivity (ICA100 edge 1377)	rfMRI connectivity (ICA100 edge 1377)
3806	rfMRI connectivity (ICA100 edge 1378)	rfMRI connectivity (ICA100 edge 1378)
3807	rfMRI connectivity (ICA100 edge 1379)	rfMRI connectivity (ICA100 edge 1379)
3808	rfMRI connectivity (ICA100 edge 1380)	rfMRI connectivity (ICA100 edge 1380)
3809	rfMRI connectivity (ICA100 edge 1381)	rfMRI connectivity (ICA100 edge 1381)
3810	rfMRI connectivity (ICA100 edge 1382)	rfMRI connectivity (ICA100 edge 1382)
3811	rfMRI connectivity (ICA100 edge 1383)	rfMRI connectivity (ICA100 edge 1383)
3812	rfMRI connectivity (ICA100 edge 1384)	rfMRI connectivity (ICA100 edge 1384)
3813	rfMRI connectivity (ICA100 edge 1385)	rfMRI connectivity (ICA100 edge 1385)
3814	rfMRI connectivity (ICA100 edge 1386)	rfMRI connectivity (ICA100 edge 1386)
3815	rfMRI connectivity (ICA100 edge 1387)	rfMRI connectivity (ICA100 edge 1387)
3816	rfMRI connectivity (ICA100 edge 1388)	rfMRI connectivity (ICA100 edge 1388)
3817	rfMRI connectivity (ICA100 edge 1389)	rfMRI connectivity (ICA100 edge 1389)

IDP number	IDP short name	IDP description
3818	rfMRI connectivity (ICA100 edge 1390)	rfMRI connectivity (ICA100 edge 1390)
3819	rfMRI connectivity (ICA100 edge 1391)	rfMRI connectivity (ICA100 edge 1391)
3820	rfMRI connectivity (ICA100 edge 1392)	rfMRI connectivity (ICA100 edge 1392)
3821	rfMRI connectivity (ICA100 edge 1393)	rfMRI connectivity (ICA100 edge 1393)
3822	rfMRI connectivity (ICA100 edge 1394)	rfMRI connectivity (ICA100 edge 1394)
3823	rfMRI connectivity (ICA100 edge 1395)	rfMRI connectivity (ICA100 edge 1395)
3824	rfMRI connectivity (ICA100 edge 1396)	rfMRI connectivity (ICA100 edge 1396)
3825	rfMRI connectivity (ICA100 edge 1397)	rfMRI connectivity (ICA100 edge 1397)
3826	rfMRI connectivity (ICA100 edge 1398)	rfMRI connectivity (ICA100 edge 1398)
3827	rfMRI connectivity (ICA100 edge 1399)	rfMRI connectivity (ICA100 edge 1399)
3828	rfMRI connectivity (ICA100 edge 1400)	rfMRI connectivity (ICA100 edge 1400)
3829	rfMRI connectivity (ICA100 edge 1401)	rfMRI connectivity (ICA100 edge 1401)
3830	rfMRI connectivity (ICA100 edge 1402)	rfMRI connectivity (ICA100 edge 1402)
3831	rfMRI connectivity (ICA100 edge 1403)	rfMRI connectivity (ICA100 edge 1403)
3832	rfMRI connectivity (ICA100 edge 1404)	rfMRI connectivity (ICA100 edge 1404)
3833	rfMRI connectivity (ICA100 edge 1405)	rfMRI connectivity (ICA100 edge 1405)
3834	rfMRI connectivity (ICA100 edge 1406)	rfMRI connectivity (ICA100 edge 1406)
3835	rfMRI connectivity (ICA100 edge 1407)	rfMRI connectivity (ICA100 edge 1407)
3836	rfMRI connectivity (ICA100 edge 1408)	rfMRI connectivity (ICA100 edge 1408)
3837	rfMRI connectivity (ICA100 edge 1409)	rfMRI connectivity (ICA100 edge 1409)
3838	rfMRI connectivity (ICA100 edge 1410)	rfMRI connectivity (ICA100 edge 1410)
3839	rfMRI connectivity (ICA100 edge 1411)	rfMRI connectivity (ICA100 edge 1411)
3840	rfMRI connectivity (ICA100 edge 1412)	rfMRI connectivity (ICA100 edge 1412)
3841	rfMRI connectivity (ICA100 edge 1413)	rfMRI connectivity (ICA100 edge 1413)
3842	rfMRI connectivity (ICA100 edge 1414)	rfMRI connectivity (ICA100 edge 1414)
3843	rfMRI connectivity (ICA100 edge 1415)	rfMRI connectivity (ICA100 edge 1415)
3844	rfMRI connectivity (ICA100 edge 1416)	rfMRI connectivity (ICA100 edge 1416)
3845	rfMRI connectivity (ICA100 edge 1417)	rfMRI connectivity (ICA100 edge 1417)
3846	rfMRI connectivity (ICA100 edge 1418)	rfMRI connectivity (ICA100 edge 1418)
3847	rfMRI connectivity (ICA100 edge 1419)	rfMRI connectivity (ICA100 edge 1419)
3848	rfMRI connectivity (ICA100 edge 1420)	rfMRI connectivity (ICA100 edge 1420)
3849	rfMRI connectivity (ICA100 edge 1421)	rfMRI connectivity (ICA100 edge 1421)
3850	rfMRI connectivity (ICA100 edge 1422)	rfMRI connectivity (ICA100 edge 1422)
3851	rfMRI connectivity (ICA100 edge 1423)	rfMRI connectivity (ICA100 edge 1423)
3852	rfMRI connectivity (ICA100 edge 1424)	rfMRI connectivity (ICA100 edge 1424)
3853	rfMRI connectivity (ICA100 edge 1425)	rfMRI connectivity (ICA100 edge 1425)
3854	rfMRI connectivity (ICA100 edge 1426)	rfMRI connectivity (ICA100 edge 1426)
3855	rfMRI connectivity (ICA100 edge 1427)	rfMRI connectivity (ICA100 edge 1427)
3856	rfMRI connectivity (ICA100 edge 1428)	rfMRI connectivity (ICA100 edge 1428)
3857	rfMRI connectivity (ICA100 edge 1429)	rfMRI connectivity (ICA100 edge 1429)
3858	rfMRI connectivity (ICA100 edge 1430)	rfMRI connectivity (ICA100 edge 1430)
3859	rfMRI connectivity (ICA100 edge 1431)	rfMRI connectivity (ICA100 edge 1431)
3860	rfMRI connectivity (ICA100 edge 1432)	rfMRI connectivity (ICA100 edge 1432)
3861	rfMRI connectivity (ICA100 edge 1433)	rfMRI connectivity (ICA100 edge 1433)
3862	rfMRI connectivity (ICA100 edge 1434)	rfMRI connectivity (ICA100 edge 1434)
3863	rfMRI connectivity (ICA100 edge 1435)	rfMRI connectivity (ICA100 edge 1435)
3864	rfMRI connectivity (ICA100 edge 1436)	rfMRI connectivity (ICA100 edge 1436)
3865	rfMRI connectivity (ICA100 edge 1437)	rfMRI connectivity (ICA100 edge 1437)
3866	rfMRI connectivity (ICA100 edge 1438)	rfMRI connectivity (ICA100 edge 1438)
3867	rfMRI connectivity (ICA100 edge 1439)	rfMRI connectivity (ICA100 edge 1439)
3868	rfMRI connectivity (ICA100 edge 1440)	rfMRI connectivity (ICA100 edge 1440)
3869	rfMRI connectivity (ICA100 edge 1441)	rfMRI connectivity (ICA100 edge 1441)
3870	rfMRI connectivity (ICA100 edge 1442)	rfMRI connectivity (ICA100 edge 1442)
3871	rfMRI connectivity (ICA100 edge 1443)	rfMRI connectivity (ICA100 edge 1443)
3872	rfMRI connectivity (ICA100 edge 1444)	rfMRI connectivity (ICA100 edge 1444)
3873	rfMRI connectivity (ICA100 edge 1445)	rfMRI connectivity (ICA100 edge 1445)
3874	rfMRI connectivity (ICA100 edge 1446)	rfMRI connectivity (ICA100 edge 1446)

IDP number	IDP short name	IDP description
3875	rfMRI connectivity (ICA100 edge 1447)	rfMRI connectivity (ICA100 edge 1447)
3876	rfMRI connectivity (ICA100 edge 1448)	rfMRI connectivity (ICA100 edge 1448)
3877	rfMRI connectivity (ICA100 edge 1449)	rfMRI connectivity (ICA100 edge 1449)
3878	rfMRI connectivity (ICA100 edge 1450)	rfMRI connectivity (ICA100 edge 1450)
3879	rfMRI connectivity (ICA100 edge 1451)	rfMRI connectivity (ICA100 edge 1451)
3880	rfMRI connectivity (ICA100 edge 1452)	rfMRI connectivity (ICA100 edge 1452)
3881	rfMRI connectivity (ICA100 edge 1453)	rfMRI connectivity (ICA100 edge 1453)
3882	rfMRI connectivity (ICA100 edge 1454)	rfMRI connectivity (ICA100 edge 1454)
3883	rfMRI connectivity (ICA100 edge 1455)	rfMRI connectivity (ICA100 edge 1455)
3884	rfMRI connectivity (ICA100 edge 1456)	rfMRI connectivity (ICA100 edge 1456)
3885	rfMRI connectivity (ICA100 edge 1457)	rfMRI connectivity (ICA100 edge 1457)
3886	rfMRI connectivity (ICA100 edge 1458)	rfMRI connectivity (ICA100 edge 1458)
3887	rfMRI connectivity (ICA100 edge 1459)	rfMRI connectivity (ICA100 edge 1459)
3888	rfMRI connectivity (ICA100 edge 1460)	rfMRI connectivity (ICA100 edge 1460)
3889	rfMRI connectivity (ICA100 edge 1461)	rfMRI connectivity (ICA100 edge 1461)
3890	rfMRI connectivity (ICA100 edge 1462)	rfMRI connectivity (ICA100 edge 1462)
3891	rfMRI connectivity (ICA100 edge 1463)	rfMRI connectivity (ICA100 edge 1463)
3892	rfMRI connectivity (ICA100 edge 1464)	rfMRI connectivity (ICA100 edge 1464)
3893	rfMRI connectivity (ICA100 edge 1465)	rfMRI connectivity (ICA100 edge 1465)
3894	rfMRI connectivity (ICA100 edge 1466)	rfMRI connectivity (ICA100 edge 1466)
3895	rfMRI connectivity (ICA100 edge 1467)	rfMRI connectivity (ICA100 edge 1467)
3896	rfMRI connectivity (ICA100 edge 1468)	rfMRI connectivity (ICA100 edge 1468)
3897	rfMRI connectivity (ICA100 edge 1469)	rfMRI connectivity (ICA100 edge 1469)
3898	rfMRI connectivity (ICA100 edge 1470)	rfMRI connectivity (ICA100 edge 1470)
3899	rfMRI connectivity (ICA100 edge 1471)	rfMRI connectivity (ICA100 edge 1471)
3900	rfMRI connectivity (ICA100 edge 1472)	rfMRI connectivity (ICA100 edge 1472)
3901	rfMRI connectivity (ICA100 edge 1473)	rfMRI connectivity (ICA100 edge 1473)
3902	rfMRI connectivity (ICA100 edge 1474)	rfMRI connectivity (ICA100 edge 1474)
3903	rfMRI connectivity (ICA100 edge 1475)	rfMRI connectivity (ICA100 edge 1475)
3904	rfMRI connectivity (ICA100 edge 1476)	rfMRI connectivity (ICA100 edge 1476)
3905	rfMRI connectivity (ICA100 edge 1477)	rfMRI connectivity (ICA100 edge 1477)
3906	rfMRI connectivity (ICA100 edge 1478)	rfMRI connectivity (ICA100 edge 1478)
3907	rfMRI connectivity (ICA100 edge 1479)	rfMRI connectivity (ICA100 edge 1479)
3908	rfMRI connectivity (ICA100 edge 1480)	rfMRI connectivity (ICA100 edge 1480)
3909	rfMRI connectivity (ICA100 edge 1481)	rfMRI connectivity (ICA100 edge 1481)
3910	rfMRI connectivity (ICA100 edge 1482)	rfMRI connectivity (ICA100 edge 1482)
3911	rfMRI connectivity (ICA100 edge 1483)	rfMRI connectivity (ICA100 edge 1483)
3912	rfMRI connectivity (ICA100 edge 1484)	rfMRI connectivity (ICA100 edge 1484)
3913	rfMRI connectivity (ICA100 edge 1485)	rfMRI connectivity (ICA100 edge 1485)
3914	rfMRI connectivity ICA-features 1	rfMRI connectivity ICA-features 1
3915	rfMRI connectivity ICA-features 2	rfMRI connectivity ICA-features 2
3916	rfMRI connectivity ICA-features 3	rfMRI connectivity ICA-features 3
3917	rfMRI connectivity ICA-features 4	rfMRI connectivity ICA-features 4
3918	rfMRI connectivity ICA-features 5	rfMRI connectivity ICA-features 5
3919	rfMRI connectivity ICA-features 6	rfMRI connectivity ICA-features 6
3920	QC T1-to-standard linear alignment discrepancy	Discrepancy between the T1 brain image (linearly-aligned to standard space) and the standard-space brain template
3921	QC T1-to-standard nonlinear alignment discrepancy	Discrepancy between the T1 brain image (nonlinearly-aligned to standard space) and the standard-space brain templat
3922	QC T1-to-standard nonlinear alignment warping	Amount of warping (averaged distortion) applied to nonlinearly align the T1 brain image to standard-space
3923	QC T1 inverse SNR	Inverted signal-to-noise ratio in the T1 - background spatial standard deviation divided by mean brain intensity
3924	QC T1 inverse CNR	Inverted contrast-to-noise ratio in the T1 - background spatial standard deviation divided by contrast between white and
3925	QC T2 FLAIR-to-T1 linear alignment discrepancy	Discrepancy between the T2 FLAIR brain image (linearly-aligned to the T1) and the T1 brain image
3926	QC dMRI-to-T1 linear alignment discrepancy	Discrepancy between the (EPI-unwarped b=0) dMRI brain image (linearly-aligned to the T1) and the T1 brain image
3927	QC SWI-to-T1 linear alignment discrepancy	Discrepancy between the (magnitude) SWI brain image (linearly-aligned to the T1) and the T1 brain image
3928	QC rfMRI-to-T1 linear alignment discrepancy	Discrepancy between the (EPI-unwarped SBref) rfMRI brain image (linearly-aligned to the T1) and the T1 brain image
3929	QC tfMRI-to-T1 linear alignment discrepancy	Discrepancy between the (EPI-unwarped SBref) tfMRI brain image (linearly-aligned to the T1) and the T1 brain image
3930	QC rfMRI head motion	Mean rfMRI head motion, averaged across space and time points
3931	QC tfMRI head motion	Mean tfMRI head motion, averaged across space and time points

IDP number	IDP short name	IDP description
3932	QC rfMRI inverse tSNR	Inverted temporal signal-to-noise ratio in the pre-processed rfMRI. Reciprocal of median (across brain voxels) of voxelw
3933	QC rfMRI cleaned inverse tSNR	Inverted temporal signal-to-noise ratio in the artefact-cleaned pre-processed rfMRI. Reciprocal of median (across brain
3934	QC tfMRI inverse tSNR	Inverted temporal signal-to-noise ratio in the pre-processed tfMRI. Reciprocal of median (across brain voxels) of voxelw
3935	QC dMRI eddy outlier slices	Number of dMRI outlier slices (across all slices in all volumes) detected and corrected

Supplemental Table 2: Genetic correlations (rg) among outcomes (severe respiratory symptoms, 13,769 cases and 1,072,442 controls; hospitalization 32,519 cases and 2,062,805 controls; infection, 122,616 cases and 2,475,240 controls) and brain imaging-derived phenotypes (IDP, assessed in 33,224 participants). Signficant estimate (p<0.01) are indicated in red.

IDP ID	IDP Description	Severe Symptoms		Hospitalization		Infection	
		rg	p	rg	p	rg	p
766	Area of parsopercularis in the right hemisphere generated by parcellation of the pial surface using Desikan-Killiany parcellation	-0.079	0.279	-0.191	0.008	-0.217	0.010
910	Area of Lat-Fis-ant-Horizont in the left hemisphere generated by parcellation of the white surface using Destrieux (a2009s) parcellation	-0.275	0.002	-0.264	0.002	-0.122	0.256
957	Area of G-front-inf-Opercular in the right hemisphere generated by parcellation of the white surface using Destrieux (a2009s) parcellation	-0.100	0.155	-0.216	0.002	-0.284	0.001
1069	Mean thickness of parahippocampal in the right hemisphere generated by parcellation of the white surface using Desikan-Killiany parcellation	0.169	0.006	0.211	0.002	0.158	0.071
1115	Mean thickness of entorhinal in the right hemisphere generated by parcellation of the white surface using BA_exvivo parcellation	0.221	0.002	0.258	0.001	0.164	0.102
1160	Mean thickness of parahippocampal in the right hemisphere generated by parcellation of the white surface using DKT parcellation	0.176	0.004	0.218	0.001	0.181	0.039
1274	Mean thickness of G-oc-temp-med-Parahip in the right hemisphere generated by parcellation of the white surface using Destrieux (a2009s) parcellation	0.212	0.006	0.259	0.001	0.267	0.016
1985	Mean OD (orientation dispersion index) in medial lemniscus (right) on FA (fractional anisotropy) skeleton (from dMRI data)	0.185	0.009	0.175	0.009	0.146	0.059
2195	rfMRI amplitudes (ICA100 node 32)	0.280	0.004	0.161	0.067	0.351	0.002
2831	rfMRI connectivity (ICA100 edge 403)	-0.174	0.006	-0.173	0.004	-0.174	0.022
2834	rfMRI connectivity (ICA100 edge 406)	0.261	0.009	0.305	0.001	0.295	0.004

Supplemental Table 3: Fraction of shared causal variants estimated by ***MiXer bivariate mixture models*** testing COVID-19 outcomes (severe respiratory symptoms, 13,769 cases and 1,072,442 controls; hospitalization 32,519 cases and 2,062,805 controls; infection, 122,616 cases and 2,475,240 controls) and brain imaging-derived phenotypes (IDP, assessed in 33,224 participants). The fit of these Bayesian models was assessed using Akaike information criterion (AIC). "Best vs. Min AIC": AIC for the best model versus model with minimal possible polygenic overlap. "Best vs. Max AIC": AIC for the best model versus model with maximum possible polygenic overlap.

COVID-19	Brain IDP	Fraction shared causal variants	Fraction shared causal variants with concorant effects	Best vs min AIC	Best vs max AIC
severe respiratory symptoms	2195	30.5%	0.228+-0.107	3.33	4.75
	0910	21.8%	0.771+-0.101	7.84	4.05
	1274	14.8%	0.325+-0.115	4.89	3.93
	1160	14.1%	0.175+-0.103	2.98	5.36
	1115	13.9%	0.205+-0.108	3.99	5.13
	2831	13.8%	0.864+-0.078	4.42	2.31
	1069	13.4%	0.179+-0.084	2.72	5.43
	1985	12.3%	0.148+-0.089	2.46	4.54
	2834	7.1%	0.013+-0.039	-1.70	-1.44
Hospitalization	2834	55.5%	0.239+-0.126	2.64	1.30
	1115	47.4%	0.358+-0.032	4.70	1.63
	1160	44.5%	0.348+-0.095	3.32	1.93
	1274	43.1%	0.395+-0.078	5.14	1.91
	1069	41.3%	0.348+-0.086	3.61	1.68
	2831	40.6%	0.728+-0.135	2.15	2.67
	1985	31.3%	0.282+-0.113	2.30	2.65
	0910	28.4%	0.736+-0.107	1.90	1.09
	0957	26.1%	0.608+-0.121	2.89	1.09
	0766	1.3%	0.985+-0.032	-1.45	-1.61
Infection	2834	43.1%	0.287+-0.129	4.07	3.04
	0957	38.5%	0.689+-0.091	4.01	3.89
	2195	31.5%	0.147+-0.109	2.59	2.57
	0766	3.0%	0.963+-0.101	-1.85	-1.58

Supplemental Table 4: Genetic causal proportion (gcp) and genetic correlation (rho) between COVID-19 outcomes (severe respiratory symptoms, 13,769 cases and 1,072,442 controls; hospitalization 32,519 cases and 2,062,805 controls; infection, 122,616 cases and 2,475,240 controls) and brain imaging-derived phenotypes (IDP, assessed in 33,224 participants) estimated by the ***latent causal variable (LCV)*** approach. SE: standard error. Results surviving Bonferroni multiple testing correction (p<0.002) are indicated in red.

COVID-19	Brain IDP	gcp	gcp_se	gcp_pval	rho	rho_se
severe respiratory symptoms	2834	0.532	0.337	0.058	0.247	0.109
	1115	0.054	0.592	0.455	0.209	0.066
	1274	-0.276	0.482	0.474	0.214	0.077
	1985	0.242	0.236	0.486	0.179	0.080
	1069	-0.257	0.454	0.544	0.169	0.061
	1160	-0.228	0.472	0.559	0.178	0.061
	2831	-0.063	0.535	0.712	-0.166	0.073
	910	0.015	0.578	0.724	-0.275	0.096
	2195	-0.080	0.528	0.810	0.282	0.108
Hospitalization	0766	0.842	0.109	2.89E-11	-0.193	0.071
	1985	0.573	0.248	0.004	0.171	0.077
	2834	0.559	0.276	0.081	0.292	0.103
	957	0.046	0.598	0.358	-0.220	0.069
	1115	0.004	0.600	0.421	0.242	0.072
	1069	-0.121	0.535	0.582	0.216	0.069
	1160	-0.094	0.546	0.591	0.225	0.067
	1274	-0.021	0.585	0.592	0.258	0.080
	2831	-0.078	0.527	0.718	-0.172	0.066
	910	-0.089	0.527	0.735	-0.270	0.094
Infection	0766	0.377	0.355	1.19E-11	-0.220	0.094
	0957	0.658	0.221	0.001	-0.288	0.089
	2195	-0.313	0.440	0.369	0.334	0.114
	2834	0.138	0.514	0.631	0.281	0.108

Supplemental Table 5: Genomic regions showing evidene of local genetic correlation (rho) between COVID-19 outcomes (severe respiratory symptoms, 13,769 cases and 1,072,442 controls; hospitalization 32,519 cases and 2,062,805 controls; infection, 122,616 cases and 2,475,240 controls) and brain imaging-derived phenotypes (IDP, assessed in 33,224 participants). Rho estimates surviving multiple testing correction (FDR q<0.05) are reported in bolded red text highlighted in yellow.Rho estimates surviving strict Bonferroni significance threshold (p<2.4e-7) are reported in bolded red text highlighted in light blue. Nominally significant results (p<0.05) are indicated in red. The description of the brain IDPs is available in Supplemental Table 1.

locus	n.snps	n.pcs	COVID-19	Brain IDP	rho	rho.lower	rho.upper	p	FDR q
2:84,395,900-85,992,794	545	140	Severe Symptoms	2834	0.386	0.085	0.847	0.017	0.330
				1160	0.264	0.014	0.541	0.039	0.426
				910	-0.303	-0.692	-0.004	0.046	0.437
				2195	-0.272	-0.625	0.011	0.062	0.476
				1069	0.246	-0.013	0.531	0.063	0.478
				1274	0.346	-0.031	1.000	0.079	0.509
				957	0.251	-0.039	0.603	0.088	0.524
				1985	-0.110	-0.591	0.287	0.546	0.876
				1115	0.070	-0.380	0.594	0.716	0.935
				766	-0.028	-0.274	0.215	0.818	0.964
			Hospitalization	2834	0.708	0.292	1.000	0.002	0.206
				1160	0.411	0.058	0.824	0.026	0.367
				1069	0.384	0.015	0.824	0.043	0.429
				1274	0.412	-0.121	1.000	0.127	0.591
				1115	0.338	-0.221	1.000	0.209	0.684
				2195	-0.237	-0.727	0.169	0.251	0.715
				957	0.209	-0.216	0.671	0.308	0.753
				910	-0.095	-0.578	0.360	0.659	0.916
				1985	-0.062	-0.708	0.512	0.804	0.960
				766	0.021	-0.334	0.383	0.906	0.982
			Infection	**2834**	**1.000**	**0.540**	**1.000**	**2.12E-05**	**0.055**
				1274	0.960	0.393	1.000	0.002	0.206
				1160	0.503	0.097	1.000	0.019	0.340
				1069	0.514	0.095	1.000	0.020	0.343
				2195	-0.554	-1.000	-0.095	0.021	0.346
				1985	-0.569	-1.000	-0.010	0.049	0.446
				957	0.148	-0.356	0.701	0.532	0.872
				910	0.051	-0.496	0.612	0.838	0.967
				766	0.023	-0.409	0.453	0.908	0.982
				1115	-0.005	-0.785	0.793	0.988	0.998
3:181,424,324-183,203,155	519	176	Hospitalization	**1069**	**0.848**	**0.568**	**1.000**	**1.46E-07**	**0.003**
				**1160**	**0.831**	**0.553**	**1.000**	**2.38E-07**	**0.003**
				**1115**	**0.628**	**0.363**	**0.946**	**1.91E-05**	**0.055**
				1274	0.643	0.311	1.000	4.40E-04	0.140
				2195	0.469	0.184	0.811	0.002	0.206
				2834	0.336	-0.094	1.000	0.119	0.578
				2831	0.123	-0.196	0.457	0.434	0.830
				766	0.093	-0.326	0.558	0.626	0.905
				910	-0.082	-0.681	0.450	0.722	0.936
				1985	0.023	-0.395	0.473	0.906	0.981
				957	-0.016	-0.339	0.300	0.916	0.983
				**1160**	**0.832**	**0.479**	**1.000**	**1.77E-05**	**0.055**
				**1069**	**0.880**	**0.513**	**1.000**	**2.19E-05**	**0.055**

locus	n.snps	n.pcs	COVID-19	Brain IDP	rho	rho.lower	rho.upper	p	FDR q
10:129,831,970-130,694,008	482	186	Hospitalization	1274	0.649	0.315	1.000	2.98E-04	0.134
				1115	0.410	0.020	1.000	0.043	0.430
				1985	0.366	0.004	0.863	0.048	0.444
				766	0.203	-0.145	0.605	0.241	0.707
				2834	0.226	-0.230	0.855	0.297	0.746
				957	-0.139	-0.474	0.172	0.374	0.796
				2195	-0.044	-0.571	0.454	0.840	0.968
				2831	-0.014	-0.408	0.381	0.936	0.988
			Infection	1160	0.688	0.225	1.000	0.005	0.254
				1069	0.730	0.248	1.000	0.006	0.255
				1274	0.525	0.083	1.000	0.024	0.361
				2831	-0.426	-1.000	0.044	0.070	0.491
				1985	0.384	-0.092	1.000	0.110	0.565
				766	0.345	-0.101	0.914	0.127	0.591
				2195	0.238	-0.377	1.000	0.391	0.804
				957	0.092	-0.349	0.547	0.654	0.914
				2834	0.104	-0.560	0.842	0.707	0.931
				1115	0.071	-0.533	0.696	0.784	0.956
20:51,533,751-52,411,532	424	176	Hospitalization	957	0.371	-0.039	0.987	0.077	0.503
				766	0.392	-0.103	1.000	0.123	0.584
				2834	-0.224	-0.693	0.177	0.250	0.715
				2831	-0.128	-0.558	0.254	0.494	0.856
				1115	-0.104	-0.450	0.230	0.527	0.871
				1985	0.118	-0.287	0.571	0.541	0.875
				1274	-0.043	-0.404	0.303	0.799	0.960
				2195	0.033	-0.309	0.371	0.839	0.968
				1069	-0.030	-0.417	0.360	0.868	0.974
				1160	-0.003	-0.399	0.397	0.985	0.997
			Infection	**2834**	**-1.000**	**-1.000**	**-0.588**	**1.97E-05**	**0.055**
				1274	-0.373	-0.848	0.019	0.066	0.485
				1115	-0.350	-0.801	0.034	0.077	0.504
				766	0.346	-0.284	1.000	0.252	0.716
				957	0.271	-0.231	0.947	0.276	0.735
				1985	-0.207	-0.736	0.272	0.368	0.792
				2831	-0.123	-0.641	0.349	0.581	0.890
				2195	0.088	-0.314	0.506	0.652	0.914
				1069	-0.050	-0.522	0.418	0.818	0.964
				1160	-0.018	-0.494	0.482	0.934	0.987
22:49,559,208-50,347,996	464	175	Hospitalization	**957**	**-0.973**	**-1.000**	**-0.588**	**1.01E-05**	**0.055**
				**766**	**-0.973**	**-1.000**	**-0.584**	**1.10E-05**	**0.055**
				2831	-0.330	-0.692	0.009	0.054	0.457
				910	-0.265	-0.818	0.180	0.232	0.702
				2195	-0.249	-0.805	0.215	0.266	0.726
				1985	0.151	-0.166	0.479	0.342	0.777
				1115	-0.183	-0.757	0.308	0.431	0.828
			Infection	910	-0.449	-1.000	0.237	0.175	0.653
				2831	-0.338	-1.000	0.180	0.195	0.672
				2195	-0.353	-1.000	0.400	0.293	0.743
				1115	-0.228	-1.000	0.574	0.511	0.863
				1985	0.032	-0.539	0.634	0.894	0.979

Supplemental Table 6: COVID-19 variants colocalizing with brain imaging-derived phenotypes (IDP; assessed in 33,224 participants) and brain expression quantitative trait loci (eQTL).

Locus	COVID-19	Brain IDP	eQTL - tissue	eQTL - gene	colocalization posterior probability	SNP	posterior probability explained by SNP
17:43390353-45389407	Hospitalization	IDP0957	NA	NA	0.838	rs79724577	1.000
17:43503521-45507570		IDP0957	NA	NA	0.831	rs56328224	0.991
4:100348074-104197934		IDP1115	NA	NA	0.964	rs13107325	0.656
1:153405826-155403057		NA	Cortex	ENSG00000160712.12	0.887	rs41264915	0.720
1:154245167-156244130		NA	Nucleus accumbens (basal ganglia)	ENSG00000177628.15	0.827	rs41264915	0.739
6:29006488-31005888		NA	Hypothalamus	ENSG00000204622.11	0.862	rs9260078	0.630
6:29006488-31005888		NA	Frontal Cortex (BA9)	ENSG00000204622.11	0.855	rs2517723	0.518
6:29006488-31005888		NA	Hippocampus	ENSG00000204622.11	0.760	rs2517723	0.510
10:78526820-80525777		NA	Cerebellar Hemisphere	ENSG00000271985.1	0.729	rs1250991	0.543
15:29488487-31487930		NA	Nucleus accumbens (basal ganglia)	ENSG00000270055.1	0.859	rs72714666	0.962
15:29488487-31487930		NA	Hypothalamus	ENSG00000270055.1	0.838	rs72714666	0.949
16:88088912-90088305		NA	Cerebellar Hemisphere	ENSG00000176715.15	0.807	rs117169628	0.965
6:30198489-32197806	Infection	NA	Caudate (basal ganglia)	ENSG00000272501.1	0.762	rs1128175	0.935
6:30272297-32271955		NA	Caudate (basal ganglia)	ENSG00000204525.16	0.816	rs2245731	0.955
7:98977207-100974920		NA	Caudate (basal ganglia)	ENSG00000160862.12	0.718	rs13243708	0.851
19:9310160-11309618		NA	Cerebellar Hemisphere	ENSG00000220201.7	0.858	rs142770866	0.694

Supplemental Table 7: Gene ontology (GO) enrichments surviving Bonferroni multiple testing correction (p<6.31E-07) across the three COVID-19 outcomes tested (severe respiratory symptoms, 13,769 cases and 1,072,442 controls; hospitalization 32,519 cases and 2,062,805 controls; infection, 122,616 cases and 2,475,240 controls) and the 11 brain imaging-derived pheotypes (IDP, assessed in 33,224 participants) identified. Enrichment estimate (enrich) and p-value (p) are reported for each result. GOCC: gene ontology cellular component; GOBP: gene ontology biological process; GOMF: gene ontology molecular function.

GO Name	GO ID	Infection		Hospitalization		Severe Symptoms		IDP2834		IDP2831		IDP2195		IDP1985		IDP1274		IDP1160		IDP1115		IDP1069		IDP0957		IDP0910		IDP0766	
		enrich	p	enrich	p	enrich	p	enrich	p	enrich	p	enrich	p	enrich	p	enrich	p	enrich	p	enrich	p	enrich	p	enrich	p	enrich	P	enrich	p
GOCC_SYNAPSE	GO:0045202	0.912	1.44E-26	0.988	6.07E-15	0.916	9.07E-54	0.834	9.21E-52	0.912	7.34E-46	0.941	1.49E-76	0.896	2.41E-45	0.942	4.86E-43	0.897	3.25E-56	0.934	1.05E-54	0.904	4.19E-57	0.841	1.27E-56	0.847	6.90E-45	0.913	2.87E-47
GOBP_POSITIVE_REGULATION_OF_MOLECULAR_FUNCTION	GO:0044093	0.971	2.14E-26	1.015	2.03E-15	0.970	9.10E-35	0.980	2.70E-44	0.872	8.26E-38	1.017	6.42E-53	0.970	2.88E-49	0.920	2.84E-51	0.988	3.39E-56	0.928	2.62E-53	0.977	6.98E-75	0.895	4.70E-54	1.073	3.45E-46	0.909	2.53E-44
GOBP_CELL_PROJECTION_ORGANIZATION	GO:0030030	0.910	5.52E-25	0.927	1.91E-17	1.025	1.37E-47	0.856	1.33E-44	0.892	2.66E-61	0.932	1.03E-51	0.914	1.80E-43	0.991	1.42E-56	1.027	1.71E-67	0.924	1.24E-59	1.025	9.73E-69	0.880	8.80E-46	0.947	3.92E-59	0.984	2.56E-44
GOCC_INTRINSIC_COMPONENT_OF_PLASMA_MEMBRANE	GO:0031226	1.033	6.88E-25	1.106	1.16E-17	1.343	7.07E-24	0.935	2.04E-42	1.109	1.85E-55	1.026	1.84E-70	0.965	6.10E-48	1.011	9.83E-58	1.008	3.00E-75	0.949	2.95E-55	1.010	3.59E-55	0.918	3.32E-56	0.947	6.15E-49	0.930	4.59E-48
GOBP_POSITIVE_REGULATION_OF_CELLULAR_BIOSYNTHETIC_PROCESS	GO:0009891	1.107	1.17E-24	1.095	3.82E-18	1.005	3.64E-46	0.965	1.42E-41	0.871	3.77E-49	1.047	6.88E-45	1.120	9.43E-65	0.975	1.31E-52	0.980	3.15E-72	0.934	1.66E-46	0.977	1.90E-76	0.967	5.65E-45	0.904	1.73E-67	0.958	4.18E-60
GOBP_SMALL_MOLECULE_METABOLIC_PROCESS	GO:0044281	1.194	3.09E-24	1.155	1.37E-18	0.999	7.08E-12	1.224	8.88E-39	1.004	4.34E-43	1.030	6.39E-59	1.124	4.18E-59	1.028	1.49E-44	0.976	1.10E-53	1.063	4.38E-53	0.965	2.70E-48	1.051	1.52E-39	1.031	3.00E-45	1.081	2.90E-48
GOBP_NITROGEN_COMPOUND_TRANSPORT	GO:0071705	1.132	3.24E-24	1.165	8.59E-18	0.963	8.80E-47	1.089	1.08E-53	1.064	2.52E-63	1.017	8.65E-46	0.983	1.96E-47	1.076	5.05E-54	1.115	1.51E-56	1.044	9.83E-53	1.122	1.43E-53	0.965	1.02E-57	0.990	2.11E-51	0.936	1.91E-57
GOMF_MOLECULAR_FUNCTION_REGULATOR	GO:0098772	1.040	2.00E-23	1.106	3.10E-22	0.845	3.21E-49	1.035	4.88E-53	0.960	5.14E-43	1.042	8.56E-52	1.043	4.41E-55	1.036	3.20E-47	1.110	1.37E-78	1.011	2.84E-48	1.126	6.43E-72	0.993	4.69E-62	1.036	7.95E-49	0.940	4.86E-51
GOBP_POSITIVE_REGULATION_OF_NUCLEOBASE_CONTAINING_COMPOUND_METABOLIC_PROCESS	GO:0045935	1.110	9.09E-23	1.114	1.99E-17	1.008	4.44E-47	0.970	6.30E-41	0.885	3.68E-51	1.033	9.55E-45	1.139	1.01E-61	1.008	9.62E-56	0.992	8.96E-81	0.978	1.74E-50	0.990	3.58E-69	0.986	8.73E-45	0.927	1.04E-55	0.990	6.83E-55
GOCC_ENDOPLASMIC_RETICULUM	GO:0005783	1.207	2.09E-22	1.259	2.72E-23	0.928	3.98E-20	1.083	3.91E-52	1.091	8.22E-48	1.080	7.43E-50	1.061	1.18E-58	1.049	3.73E-63	1.041	2.56E-53	1.012	2.07E-73	1.049	1.96E-57	0.928	4.11E-63	1.040	9.96E-54	0.917	1.71E-57
GOBP_CELLULAR_MACROMOLECULE_LOCALIZATION	GO:0070727	1.117	2.27E-22	1.128	9.94E-18	0.930	1.97E-43	0.960	1.33E-36	1.003	5.76E-47	0.946	1.82E-37	0.929	3.55E-47	0.859	8.67E-53	1.005	5.27E-49	0.889	4.03E-42	1.017	6.63E-48	0.949	1.22E-44	1.029	7.72E-42	0.963	5.57E-44
GOBP_LOCOMOTION	GO:0040011	0.977	3.66E-22	0.973	3.24E-21	1.024	1.74E-46	0.934	1.19E-51	0.866	7.25E-62	0.926	4.69E-72	0.969	1.19E-62	0.830	5.63E-56	0.959	1.82E-69	0.883	7.68E-61	0.960	2.23E-112	0.882	2.77E-66	0.981	6.41E-53	0.993	6.65E-65
GOBP_NEURON_DIFFERENTIATION	GO:0030182	0.930	8.59E-22	0.931	1.12E-15	1.064	3.76E-45	0.812	7.10E-32	0.849	6.78E-63	0.932	5.35E-56	0.962	7.34E-42	0.899	3.74E-46	0.930	7.89E-56	0.921	1.41E-54	0.937	1.80E-73	0.913	1.51E-50	0.919	2.46E-50	0.975	9.06E-46
GOBP_REGULATION_OF_INTRACELLULAR_SIGNAL_TRANSDUCTION	GO:1902531	1.012	1.14E-21	1.031	8.53E-19	1.039	2.07E-34	1.039	3.27E-40	0.876	4.81E-43	0.945	1.90E-53	0.971	9.89E-58	0.961	8.05E-47	1.024	6.19E-65	0.942	9.76E-74	1.009	2.15E-88	0.944	1.24E-56	1.049	2.62E-50	0.935	4.95E-58
GOMF_SIGNALING_RECEPTOR_BINDING	GO:0005102	1.158	1.49E-21	1.192	1.34E-15	1.165	5.54E-14	1.013	1.31E-42	0.998	7.62E-36	1.077	4.83E-47	1.076	3.37E-45	1.007	2.16E-33	1.034	3.36E-38	1.018	2.04E-41	1.030	2.97E-42	1.002	2.28E-41	1.018	1.12E-37	1.089	7.16E-51
GOBP_NEUROGENESIS	GO:0022008	0.972	2.17E-21	0.971	4.69E-16	1.023	4.34E-48	0.827	8.23E-39	0.850	1.25E-62	0.961	2.22E-65	0.949	1.04E-50	0.889	1.01E-57	0.950	2.22E-72	0.908	1.00E-61	0.958	2.18E-90	0.940	1.13E-58	0.949	5.70E-60	0.972	2.88E-57
GOBP_TISSUE_DEVELOPMENT	GO:0009888	1.108	3.10E-21	1.093	1.25E-23	1.035	3.59E-44	0.926	3.27E-40	0.820	2.24E-62	0.981	1.51E-81	1.033	3.42E-62	0.922	4.23E-64	1.019	5.01E-59	0.981	2.55E-67	1.014	1.31E-90	0.969	3.25E-58	0.929	1.11E-52	1.029	2.30E-61
GOBP_REGULATION_OF_PROTEIN_MODIFICATION_PROCESS	GO:0031399	1.121	1.29E-20	1.160	1.17E-14	0.952	1.69E-32	0.934	2.81E-35	0.833	1.93E-34	0.963	1.59E-38	1.037	1.05E-39	0.938	2.42E-46	1.008	1.78E-51	1.017	4.66E-38	0.995	1.15E-53	0.867	1.64E-36	1.018	1.56E-39	0.994	1.96E-46
GOBP_REGULATION_OF_TRANSPORT	GO:0051049	1.052	1.63E-20	1.103	9.87E-17	0.933	8.31E-48	1.032	7.70E-53	1.112	3.94E-70	0.991	6.81E-48	0.952	4.44E-42	0.961	2.37E-57	1.052	5.86E-63	1.002	9.78E-58	1.040	6.12E-57	0.944	3.82E-67	0.945	1.39E-39	1.002	2.60E-45
GOBP_NEURON_DEVELOPMENT	GO:0048666	0.889	3.02E-20	0.895	1.59E-13	1.082	1.01E-32	0.796	1.57E-31	0.843	1.46E-50	0.884	1.68E-43	0.921	1.57E-30	0.885	9.26E-36	0.934	1.05E-47	0.899	6.67E-44	0.943	1.64E-57	0.875	1.29E-40	0.943	2.32E-43	0.924	7.66E-37
GOBP_PEPTIDYL_AMINO_ACID_MODIFICATION	GO:0018193	1.117	5.40E-20	1.238	7.70E-13	0.991	6.59E-25	1.032	4.11E-36	0.943	1.94E-32	0.873	3.32E-35	0.955	2.58E-46	0.933	2.29E-37	0.981	4.54E-54	0.992	7.34E-25	0.968	2.47E-38	0.846	4.27E-34	0.924	2.38E-32	0.878	7.46E-30
GOBP_PHOSPHORYLATION	GO:0016310	1.043	6.19E-20	1.093	2.89E-17	0.894	1.55E-30	1.026	8.18E-48	0.857	2.92E-45	1.005	5.55E-52	1.044	2.39E-58	0.951	7.19E-53	0.996	9.38E-55	0.940	4.83E-63	0.981	9.01E-56	0.895	7.20E-60	1.009	3.20E-48	1.008	7.07E-58
GOBP_ESTABLISHMENT_OF_PROTEIN_LOCALIZATION	GO:0045184	1.154	7.18E-20	1.152	1.34E-15	0.901	9.34E-43	1.066	5.78E-35	0.953	6.69E-50	0.975	3.85E-38	0.901	1.07E-38	1.003	1.23E-45	1.063	3.52E-48	1.022	2.57E-43	1.078	2.58E-39	0.944	5.93E-49	1.007	3.89E-40	0.889	1.00E-45
GOBP_POSITIVE_REGULATION_OF_RNA_METABOLIC_PROCESS	GO:0051254	1.109	8.50E-20	1.093	1.67E-15	1.028	6.54E-44	0.958	3.42E-41	0.863	2.44E-46	1.027	1.53E-38	1.123	9.20E-59	1.004	2.89E-48	0.985	2.79E-75	0.974	7.38E-47	0.981	2.59E-66	0.989	3.30E-39	0.922	1.32E-57	0.986	1.16E-50
GOBP_POSITIVE_REGULATION_OF_SIGNALING	GO:0023056	1.119	1.07E-19	1.147	1.39E-23	1.141	1.15E-30	1.045	4.41E-43	0.984	1.31E-42	1.044	3.61E-50	1.119	1.11E-47	1.003	6.24E-45	1.124	8.68E-67	1.003	9.82E-60	1.107	5.38E-79	1.033	2.26E-51	1.145	2.16E-52	1.072	9.01E-65
GOBP_TUBE_DEVELOPMENT	GO:0035295	1.046	1.20E-19	1.080	1.64E-12	1.222	5.05E-26	1.041	1.68E-29	0.927	5.63E-38	0.957	4.52E-42	1.081	6.13E-43	1.027	4.34E-48	1.115	5.62E-49	0.972	3.72E-45	1.115	2.03E-57	1.050	1.00E-43	1.101	5.32E-35	1.092	1.54E-35
GOMF_RNA_BINDING	GO:0003723	1.353	1.26E-19	1.420	5.46E-22	0.941	1.15E-25	1.079	1.18E-31	0.975	3.57E-35	1.051	1.18E-38	1.107	6.59E-41	0.985	1.11E-54	1.041	2.73E-29	1.044	4.69E-39	1.045	3.39E-44	1.150	7.79E-47	1.012	4.78E-36	1.187	6.91E-59
GOBP_ION_TRANSPORT	GO:0006811	1.056	1.27E-19	1.052	4.78E-19	1.026	4.35E-43	1.058	1.16E-39	1.284	3.19E-59	1.178	3.12E-59	1.013	7.91E-35	1.032	5.02E-50	1.036	7.35E-51	1.054	7.02E-43	1.032	7.67E-53	0.878	3.61E-46	0.929	4.59E-43	0.910	1.37E-39
GOBP_TRANSMEMBRANE_TRANSPORT	GO:0055085	1.100	4.13E-19	1.106	1.04E-16	1.005	6.21E-39	1.027	8.60E-37	1.239	3.59E-47	1.177	2.65E-58	1.009	9.54E-41	1.003	3.99E-47	1.002	4.53E-61	0.995	2.82E-44	0.995	6.57E-49	0.918	5.00E-50	0.929	7.73E-39	0.901	3.88E-42
GOBP_CELL_MIGRATION	GO:0016477	0.954	6.72E-19	0.916	2.28E-18	0.977	7.39E-34	0.950	2.33E-44	0.831	1.12E-49	0.907	2.07E-59	0.949	1.33E-53	0.813	1.87E-48	0.900	3.77E-51	0.887	1.10E-56	0.892	3.55E-82	0.892	2.15E-59	0.971	1.65E-43	0.975	5.99E-59
GOBP_REGULATION_OF_PHOSPHORUS_METABOLIC_PROCESS	GO:0051174	1.086	8.87E-19	1.151	6.30E-14	0.918	6.13E-26	0.953	1.94E-33	0.805	5.38E-31	0.986	1.46E-42	1.080	3.58E-40	0.888	6.21E-49	1.031	1.25E-50	0.952	3.90E-43	1.007	3.96E-52	0.904	2.98E-46	1.023	1.07E-39	0.994	1.48E-39
GOMF_RIBONUCLEOTIDE_BINDING	GO:0032553	1.129	1.41E-18	1.205	1.26E-12	0.876	1.50E-12	1.033	4.72E-51	0.907	6.46E-48	1.063	2.01E-71	0.924	1.49E-48	0.962	5.28E-57	0.954	1.75E-45	0.932	8.59E-53	0.961	4.04E-62	0.979	9.64E-73	1.024	1.00E-57	0.950	6.98E-49
GOBP_RESPONSE_TO_OXYGEN_CONTAINING_COMPOUND	GO:1901700	1.156	1.77E-18	1.195	3.90E-17	1.024	1.26E-39	1.122	5.37E-44	0.958	3.46E-51	1.087	5.44E-51	1.086	2.82E-48	1.001	2.26E-60	1.016	1.17E-48	1.030	6.42E-49	1.012	1.19E-56	1.001	8.33E-55	0.939	8.59E-46	1.040	1.53E-40
GOBP_ORGANONITROGEN_COMPOUND_BIOSYNTHETIC_PROCESS	GO:1901566	1.283	2.56E-18	1.315	1.77E-17	0.985	9.69E-60	1.136	5.76E-52	1.093	6.64E-37	1.012	1.53E-39	1.135	1.01E-52	0.977	5.11E-49	0.992	1.39E-66	1.095	1.28E-60	1.003	1.88E-58	1.178	2.29E-46	1.069	7.06E-41	1.198	2.17E-32
GOBP_NEGATIVE_REGULATION_OF_RESPONSE_TO_STIMULUS	GO:0048585	1.089	2.99E-18	1.102	2.06E-14	1.032	2.89E-49	0.989	4.73E-40	0.906	1.03E-42	0.936	3.31E-47	0.998	2.30E-46	0.986	8.20E-42	0.987	1.53E-52	1.004	3.68E-47	0.992	7.70E-66	0.978	6.93E-65	0.968	1.30E-42	1.012	4.49E-66
GOCC_SUPRAMOLECULAR_COMPLEX	GO:0099080	1.159	3.45E-18	1.175	9.98E-15	0.984	5.95E-27	0.916	4.42E-31	0.989	4.12E-34	0.890	3.84E-31	0.944	2.03E-41	1.022	4.04E-41	0.980	1.18E-39	1.052	9.97E-26	0.987	1.44E-36	1.026	2.11E-35	1.035	9.10E-35	0.975	4.00E-36
GOBP_RESPONSE_TO_ABIOTIC_STIMULUS	GO:0009628	1.098	4.94E-18	1.139	6.79E-13	0.902	1.53E-16	1.111	5.42E-31	0.994	3.31E-38	1.162	4.35E-26	1.035	7.96E-27	1.051	6.66E-35	1.010	5.37E-29	1.076	1.82E-36	1.010	8.69E-33	0.970	7.63E-36	1.060	6.34E-28	1.019	1.55E-26
GOBP_NEGATIVE_REGULATION_OF_MOLECULAR_FUNCTION	GO:0044092	1.103	4.95E-18	1.174	1.42E-13	0.894	2.55E-18	1.043	2.72E-23	1.051	4.72E-29	1.000	3.21E-26	1.095	3.87E-30	0.980	1.18E-27	1.229	3.24E-37	1.014	7.87E-31	1.214	4.54E-29	0.992	5.80E-32	0.976	2.14E-29	0.882	1.52E-19
GOBP_ORGANOPHOSPHATE_METABOLIC_PROCESS	GO:0019637	1.184	5.12E-18	1.145	2.26E-10	0.951	5.52E-22	1.320	3.85E-22	1.100	4.08E-22	1.114	9.51E-25	1.157	3.96E-26	1.059	3.27E-24	1.040	1.13E-36	1.023	1.81E-31	1.044	4.96E-28	0.973	2.04E-28	0.992	9.01E-31	1.064	4.28E-25
GOBP_TUBE_MORPHOGENESIS	GO:0035239	1.027	5.27E-18	1.075	1.84E-10	1.143	6.60E-24	1.108	1.59E-25	0.881	5.78E-31	0.917	6.15E-35	1.148	5.76E-34	1.027	9.47E-39	1.095	2.15E-39	0.975	2.95E-40	1.097	5.12E-52	1.026	5.77E-34	1.159	9.11E-32	1.056	2.88E-28
GOBP_ENZYME_LINKED_RECEPTOR_PROTEIN_SIGNALING_PATHWAY	GO:0007167	1.018	9.86E-18	1.029	5.00E-13	0.922	2.56E-26	1.019	3.27E-26	0.838	3.67E-26	0.908	1.04E-38	1.042	6.24E-32	0.953	5.44E-30	1.026	4.66E-39	0.983	5.99E-41	0.997	8.28E-40	0.837	3.72E-29	1.068	1.20E-30	0.892	8.40E-34
GOBP_POSITIVE_REGULATION_OF_CATALYTIC_ACTIVITY	GO:0043085	0.995	1.22E-17	1.047	1.12E-12	0.944	2.90E-27	1.010	2.28E-32	0.898	4.92E-31	1.038	2.38E-42	0.966	7.99E-41	0.958	3.91E-42	1.038	3.11E-47	0.969	5.61E-39	1.016	9.36E-53	0.955	2.11E-39	1.128	5.91E-36	0.996	1.13E-31
GOCC_NEURON_PROJECTION	GO:0043005	1.009	1.28E-17	1.088	7.32E-14	0.853	9.87E-46	0.898	1.86E-50	0.908	1.16E-45	0.906	9.84E-45	0.858	1.20E-56	0.942	1.23E-46	1.005	1.74E-57	0.943	4.18E-66	1.008	2.86E-42	0.735	9.22E-39	0.888	1.79E-48	0.815	1.06E-47
GOBP_CELL_CELL_SIGNALING	GO:0007267	1.054	1.44E-17	1.113	7.62E-13	1.044	2.40E-33	0.925	4.36E-56	0.932	3.28E-44	1.029	2.20E-67	1.030	2.37E-51	0.982	4.48E-51	0.966	6.76E-60	0.994	4.93E-64	0.968	1.34E-51	0.932	8.62E-69	0.968	1.66E-44	0.968	5.28E-73
GOMF_ENZYME_REGULATOR_ACTIVITY	GO:0030234	0.927	1.94E-17	0.979	5.73E-16	0.737	1.06E-32	0.914	1.05E-36	0.877	1.69E-25	0.929	1.31E-33	0.947	3.77E-36	0.949	1.46E-33	0.993	6.74E-55	0.923	9.12E-34	1.020	2.94E-52	0.885	1.38E-48	0.982	1.82E-38	0.858	2.17E-27
GOBP_REGULATION_OF_HYDROLASE_ACTIVITY	GO:0051336	0.931	1.95E-17	0.998	2.62E-09	0.864	2.54E-25	1.026	6.54E-29	0.929	1.20E-30	0.987	2.08E-36	0.859	4.80E-33	0.982	8.72E-38	1.058	7.76E-36	0.972	1.22E-30	1.066	8.09E-38	0.955	3.01E-31	0.992	5.31E-29	0.910	1.74E-21
GOBP_RESPONSE_TO_ENDOGENOUS_STIMULUS	GO:0009719	1.047	2.27E-17	1.048	2.15E-19	1.009	8.01E-47	1.008	6.61E-36	0.898	2.66E-46	1.018	1.98E-46	0.965	1.71E-47	0.931	1.69E-43	1.024	1.64E-54	1.003	1.47E-47	1.021	1.08E-58	0.918	1.12E-45	1.008	4.50E-39	1.009	1.17E-56
GOBP_REGULATION_OF_ANATOMICAL_STRUCTURE_MORPHOGENESIS	GO:0022603	0.978	2.70E-17	1.033	8.74E-12	1.113	2.71E-27	0.837	9.50E-25	0.890	6.88E-27	0.949	1.61E-37	1.007	1.82E-33	0.887	3.95E-29	1.073	3.61E-39	0.928	5.66E-47	1.093	6.45E-36	0.884	9.80E-37	0.966	8.39E-32	0.947	6.70E-35
GOBP_HOMEOSTATIC_PROCESS	GO:0042592	1.160	2.96E-17	1.178	2.25E-19	0.997	1.89E-38	1.001	2.76E-42	1.045	2.49E-51	1.089	8.33E-53	0.980	1.97E-49	0.967	4.58E-50	1.003	1.29E-50	0.983	2.07E-54	1.009	1.05E-53	0.914	1.35E-48	0.975	9.53E-55	0.924	1.37E-43
GOBP_PROTEOLYSIS	GO:0006508	1.213	3.18E-17	1.266	2.89E-17	1.029	1.28E-35	1.187	3.12E-39	1.009	2.08E-39	0.965	1.09E-40	1.047	3.15E-54	1.066	7.44E-49	1.104	1.65E-57	1.099	3.87E-60	1.106	1.44E-64	1.095	1.71E-52	1.092	9.28E-44	1.003	8.57E-41
GOBP_REGULATION_OF_CELL_POPULATION_PROLIFERATION	GO:0042127	1.197	3.63E-17	1.229	4.55E-15	1.054	4.30E-39	1.083	6.61E-53	0.947	1.77E-39	1.015	1.96E-53	1.125	1.09E-37	1.035	7.19E-62	1.006	1.53E-54	1.005	4.23E-56	0.994	1.65E-58	1.061	2.16E-57	0.988	4.97E-49	1.086	2.76E-54
GOBP_CELLULAR_COMPONENT_MORPHOGENESIS	GO:0032989	0.873	5.69E-17	0.854	2.49E-10	1.112	2.04E-24	0.742	1.06E-22	0.854	1.01E-34	0.841	7.61E-35	0.910	4.07E-30	0.829	3.44E-23	0.994	6.09E-36	0.853	2.43E-35	1.013	6.26E-35	0.854	1.14E-28	0.943	7.61E-40	0.927	9.56E-27
GOBP_NEGATIVE_REGULATION_OF_SIGNALING	GO:0023057	1.099	6.17E-17	1.122	2.04E-14	1.069	6.39E-43	0.962	3.38E-34	0.884	8.51E-42	0.936	3.07E-43	0.954	3.47E-39	0.978	5.10E-35	0.922	9.84E-38	1.012	5.19E-45	0.913	2.60E-47	0.947	2.19E-53	0.953	1.51E-42	0.987	4.39E-53
GOCC_ENVELOPE	GO:0031975	1.313	7.62E-17	1.420	3.33E-17	1.077	6.55E-27	1.032	3.44E-22	1.053	5.32E-31	0.996	3.33E-26	1.095	1.76E-29	1.098	3.68E-31	1.076	2.22E-34	1.032	7.93E-33	1.064	9.70E-37	1.005	1.73E-26	1.185	8.80E-27	0.814	3.79E-30
GOBP_INTRACELLULAR_TRANSPORT	GO:0046907	1.283	8.96E-17	1.345	1.28E-14	1.023	2.00E-33	1.182	8.95E-35	1.118	3.38E-45	0.870	4.23E-34	1.000	1.26E-52	1.059	2.85E-51	1.156	1.35E-52	1.066	9.19E-46	1.169	1.16E-39	1.028	5.37E-43	1.120	3.21E-35	0.979	1.01E-25
GOBP_CELLULAR_RESPONSE_TO_ENDOGENOUS_STIMULUS	GO:0071495	1.020	1.21E-16	1.028	8.28E-18	1.014	2.37E-40	0.996	3.42E-34	0.848	4.40E-33	1.008	5.69E-34	0.946	4.65E-43	0.886	7.24E-36	1.013	1.46E-48	0.967	2.54E-41	1.007	4.58E-53	0.911	1.95E-41	1.029	8.54E-32	0.994	1.48E-44
GOBP_POSITIVE_REGULATION_OF_MULTICELLULAR_ORGANISMAL_PROCESS	GO:0051240	1.092	1.31E-16	1.092	3.60E-13	1.046	4.85E-27	0.991	1.79E-38	1.004	7.21E-56	1.124	4.20E-49	1.120	1.19E-48	0.968	5.99E-63	1.041	1.64E-54	0.972	5.98E-43	1.029	3.49E-53	0.963	2.08E-45	0.957	1.91E-33	0.983	2.17E-59
GOBP_CELL_MORPHOGENESIS	GO:0000902	0.900	2.94E-16	0.919	1.74E-13	0.984	3.23E-31	0.795	3.05E-29	0.812	4.83E-41	0.803	6.28E-46	0.898	4.58E-36	0.784	1.32E-28	0.968	5.79E-49	0.824	1.04E-42	0.980	5.11E-55	0.836	9.72E-44	0.923	2.73E-46	0.958	8.81E-37
GOBP_CIRCULATORY_SYSTEM_DEVELOPMENT	GO:0072359	1.102	3.12E-16	1.119	1.27E-12	1.134	2.18E-30	1.037	1.68E-29	0.783	3.71E-34	0.968	3.91E-41	1.090	3.23E-50	0.934	5.70E-49	1.019	4.57E-43	0.919	2.96E-39	1.010	2.35E-63	1.011	2.06E-37	0.992	4.90E-33	0.998	6.39E-43
GOBP_CELL_PART_MORPHOGENESIS	GO:0032990	0.796	3.38E-16	0.791	6.76E-12	1.118	4.15E-21	0.768	4.47E-22	0.858	4.89E-31	0.829	1.89E-32	0.909	9.28E-26	0.841	3.35E-22	1.015	4.83E-33	0.889	2.82E-33	1.033	4.90E-34	0.853	1.17E-27	0.970	4.58E-35	0.969	1.37E-24
GOBP_POSITIVE_REGULATION_OF_TRANSCRIPTION_BY_RNA_POLYMERASE_II	GO:0045944	1.093	7.06E-16	1.080	1.29E-11	1.128	8.54E-33	0.899	2.02E-31	0.834	3.37E-30	1.028	6.87E-33	1.175	1.91E-47	0.972	6.08E-36	0.906	2.38E-47	0.961	5.23E-39	0.899	3.00E-43	0.972	1.61E-29	0.908	9.69E-41	0.950	5.01E-37
GOBP_POSITIVE_REGULATION_OF_DEVELOPMENTAL_PROCESS	GO:0051094	1.088	7.91E-16	1.089	2.97E-12	1.039	4.82E-33	1.013	1.09E-31	0.971	2.93E-35	1.078	2.37E-49	1.121	1.12E-34	1.002	6.97E-48	1.066	2.30E-45	1.013	1.42E-43	1.053	6.11E-54	0.998	3.78E-47	0.905	4.03E-38	0.997	7.66E-54
GOMF_ADENYL_NUCLEOTIDE_BINDING	GO:0030554	1.083	8.19E-16	1.139	5.30E-11	0.882	1.10E-09	1.012	2.10E-37	0.883	1.35E-43	1.055	1.39E-60	0.893	1.75E-38	0.963	1.18E-45	0.901	5.90E-40	0.922	1.12E-50	0.907	1.20E-45	0.940	1.76E-63	0.991	7.20E-44	0.931	8.50E-41
GOBP_DEFENSE_RESPONSE	GO:0006952	1.261	8.42E-16	1.336	6.51E-12	1.235	1.60E-20	1.295	8.75E-30	1.151	2.63E-26	1.158	3.02E-42	1.167	1.88E-36	1.174	3.90E-35	1.082	1.52E-47	1.071	1.33E-40	1.075	2.19E-63	1.196	6.65E-38	1.040	1.93E-36	1.132	9.84E-49
GOBP_REGULATION_OF_CELL_DIFFERENTIATION	GO:0045595	1.175	1.07E-15	1.153	3.80E-15	1.048	6.49E-51	0.912	7.34E-39	0.886	9.24E-42	1.067	1.15E-61	1.045	6.39E-39	0.920	1.03E-51	0.960	1.54E-43	0.910	8.46E-45	0.964	4.16E-75	0.920	7.88E-44	0.872	1.71E-52	0.990	2.30E-64
GOBP_CATION_TRANSPORT	GO:0006812	1.073	1.10E-15	1.069	1.31E-14	1.060	3.74E-35	1.060	3.31E-33	1.229	2.46E-43	1.130	4.45E-43	0.981	1.35E-28	1.003	1.60E-34	0.981	1.51E-41	1.008	1.85E-31	0.974	3.29E-39	0.829	1.09E-34	0.917	1.37E-34	0.847	1.07E-28
GOBP_CELLULAR_MACROMOLECULE_BIOSYNTHETIC_PROCESS	GO:0009059	1.396	1.14E-15	1.408	1.25E-14	0.963	7.50E-72	1.157	4.09E-35	1.073	4.06E-35	1.066	1.39E-44	1.113	8.26E-43	1.128	1.18E-42	1.074	5.63E-46	1.192	2.91E-50	1.102	4.94E-56	1.190	4.61E-39	1.115	1.20E-40	1.187	2.67E-34
GOBP_BIOLOGICAL_ADHESION	GO:0022409	0.962	1.74E-15	0.988	4.18E-18	1.018	9.01E-36	0.777	1.72E-49	0.800	1.97E-48	0.801	8.82E-48	0.899	1.31E-46	0.824	9.16E-57	0.900	3.83E-64	0.841	8.04E-70	0.909	1.44E-67	0.801	7.04E-48	0.815	2.02E-67	0.838	6.20E-68
GOBP_ION_TRANSMEMBRANE_TRANSPORT	GO:0034220	1.096	2.32E-15	1.088	1.54E-14	1.010	7.96E-33	1.023	7.82E-29	1.195	8.32E-40	1.170	1.30E-40	0.949	6.66E-32	1.051	1.71E-36	0.999	5.24E-46	1.030	2.60E-31	0.992	2.01E-37	0.863	1.41E-34	0.911	6.02E-31	0.860	3.45E-28
GOBP_ORGANELLE_ASSEMBLY	GO:0070925	1.314	2.55E-15	1.316	6.81E-12	0.878	3.65E-18	0.973	4.14E-32	1.033	2.31E-22	0.917	1.54E-20	1.036	4.62E-34	1.193	2.15E-21	1.156	3.68E-30	1.023	5.87E-26	1.154	7.83E-25	0.914	1.40E-22	0.925	1.60E-27	1.034	4.42E-19
GOCC_ORGANELLE_SUBCOMPARTMENT	GO:0031984	1.244	4.22E-15	1.261	2.33E-19	0.888	2.85E-38	1.079	6.24E-42	0.985	5.30E-27	1.079	5.88E-36	0.947	6.87E-38	1.069	3.01E-37	1.071	5.21E-37	0.971	9.37E-40	1.098	1.90E-49	0.951	1.33E-43	1.077	6.45E-40	0.908	3.29E-38
GOBP_REGULATION_OF_ORGANELLE_ORGANIZATION	GO:0033043	1.044	4.33E-15	1.090	3.30E-15	0.974	9.24E-22	1.069	4.12E-27	0.942	9.88E-28	1.004	2.13E-32	0.887	1.61E-52	0.991	1.39E-38	1.064	1.75E-37	0.902	4.67E-38	1.102	4.66E-39	1.000	1.19E-34	0.976	1.21E-36	0.996	5.67E-21
GOBP_CELLULAR_RESPONSE_TO_OXYGEN_CONTAINING_COMPOUND	GO:1901701	1.156	4.65E-15	1.195	4.43E-13	1.027	4.57E-25	1.100	1.92E-28	0.901	5.50E-34	1.083	5.45E-40	1.081	2.10E-35	0.937	5.06E-37	1.074	2.10E-40	1.026	1.51E-39	1.060	1.49E-35	0.932	8.86E-38	0.945	2.38E-30	1.009	2.75E-33
GOBP_ANATOMICAL_STRUCTURE_FORMATION_INVOLVED_IN_MORPHOGENESIS	GO:0048646	1.141	4.91E-15	1.155	1.39E-12	1.143	2.09E-27	1.080	1.18E-29	0.861	2.05E-36	0.996	1.85E-48	1.147	2.82E-43	1.022	6.38E-48	1.112	5.41E-55	0.940	1.13E-39	1.109	1.03E-65	1.044	1.84E-39	1.062	3.11E-38	0.991	7.99E-34
GOBP_REGULATION_OF_MULTICELLULAR_ORGANISMAL_DEVELOPMENT	GO:2000026	1.100	5.30E-15	1.137	1.05E-12	1.044	8.36E-38	0.969	9.38E-35	0.948	6.24E-40	1.029	6.15E-64	1.196	6.51E-42	0.993	3.75E-56	1.062	1.13E-44	0.925	4.03E-36	1.069	2.09E-70	0.970	2.93E-44	0.954	1.72E-38	1.049	3.39E-47
GOCC_PLASMA_MEMBRANE_REGION	GO:0098590	1.009	5.70E-15	1.047	2.15E-13	0.914	4.20E-42	0.933	9.27E-38	0.937	3.20E-38	0.961	4.00E-48	0.938	2.80E-44	0.976	1.73E-46	0.959	3.55E-48	1.005	4.96E-54	0.968	5.87E-47	0.841	3.12E-52	0.828	1.10E-44	0.885	1.47E-37
GOCC_CHROMOSOME	GO:0005694	1.263	6.27E-15	1.307	4.01E-19	1.059	9.26E-31	1.013	4.04E-40	0.871	6.84E-41	1.113	3.77E-44	1.001	1.22E-34	0.998	2.97E-47	0.942	1.42E-73	1.068	5.96E-46	0.964	1.06E-68	0.986	5.01E-49	0.961	4.31E-42	0.975	3.02E-60
GOCC_SUPRAMOLECULAR_POLYMER	GO:0099081	1.168	6.73E-15	1.152	1.66E-10	0.986	1.90E-23	0.948	1.02E-27	0.986	2.02E-23	0.876	7.24E-24	0.925	1.35E-35	1.012	3.87E-30	1.024	4.64E-32	1.000	1.96E-28	1.032	8.00E-30	0.950	1.53E-30	1.053	7.96E-29	0.900	1.33E-26
GOBP_VESICLE_MEDIATED_TRANSPORT	GO:0016192	1.067	7.67E-15	1.150	7.21E-14	0.958	1.69E-37	1.060	2.91E-35	0.983	4.06E-44	0.855	3.91E-49	0.866	9.64E-38	1.066	8.87E-53	1.090	1.80E-51	1.030	9.22E-64	1.090	2.03E-66	0.950	9.43E-53	1.035	1.44E-33	0.971	1.69E-34
GOMF_TRANSPORTER_ACTIVITY	GO:0005215	1.141	8.32E-15	1.144	3.18E-14	1.130	5.19E-33	1.102	5.30E-32	1.357	3.01E-38	1.330	1.45E-56	1.012	6.51E-35	1.042	1.15E-36	0.975	4.54E-48	1.026	3.31E-42	0.971	5.32E-40	0.939	1.50E-38	0.964	1.52E-29	0.971	6.12E-32
GOBP_POSITIVE_REGULATION_OF_GENE_EXPRESSION	GO:0010628	1.093	9.46E-15	1.092	7.24E-12	1.132	5.62E-22	1.067	9.79E-26	0.969	1.66E-29	1.144	6.91E-30	1.186	5.63E-39	0.958	4.31E-36	0.959	1.15E-37	0.977	1.87E-26	0.977	6.90E-47	1.034	1.40E-31	0.946	2.91E-25	1.010	1.15E-32
GOBP_REGULATION_OF_IMMUNE_SYSTEM_PROCESS	GO:0002682	1.122	9.76E-15	1.240	1.66E-13	0.948	1.32E-23	1.143	1.26E-28	1.094	1.77E-29	1.023	1.18E-33	1.049	1.16E-29	1.041	3.85E-49	1.029	4.04E-39	0.951	3.40E-41	1.018	8.09E-43	0.989	2.16E-38	0.906	1.59E-40	0.906	5.52E-40
GOBP_BIOLOGICAL_PROCESS_INVOLVED_IN_INTERSPECIES_INTERACTION_BETWEEN_ORGANISMS	GO:0044419	1.305	1.12E-14	1.375	1.81E-12	1.142	3.95E-23	1.257	2.54E-31	1.154	9.04E-36	1.096	2.22E-37	1.195	1.06E-39	1.188	3.86E-34	1.106	3.76E-46	0.980	1.30E-36	1.106	3.04E-48	1.054	2.18E-41	1.055	2.76E-41	0.972	1.49E-37
GOBP_NEGATIVE_REGULATION_OF_PROTEIN_METABOLIC_PROCESS	GO:0051248	1.088	1.18E-14	1.060	3.40E-12	0.851	4.38E-34	1.047	2.06E-20	0.909	4.05E-22	0.852	2.12E-22	1.143	5.08E-31	0.995	2.27E-26	1.074	1.10E-35	1.073	2.22E-29	1.089	9.77E-33	0.916	1.26E-22	0.974	7.77E-31	0.961	3.30E-29
GOBP_CELL_JUNCTION_ORGANIZATION	GO:0034330	0.810	1.45E-14	0.919	1.56E-08	0.774	2.09E-25	0.847	4.24E-24	0.836	9.88E-23	0.805	5.92E-31	0.866	9.05E-27	0.869	1.10E-23	0.923	1.75E-27	0.791	2.52E-24	0.937	3.73E-33	0.698	1.24E-25	0.832	4.03E-22	0.803	4.90E-26
GOBP_POSITIVE_REGULATION_OF_PROTEIN_MODIFICATION_PROCESS	GO:0031401	1.095	1.48E-14	1.171	2.47E-09	0.968	7.76E-18	0.903	1.98E-23	0.769	6.82E-27	0.889	5.45E-32	1.058	3.72E-29	0.916	1.54E-33	0.979	2.88E-33	0.997	9.44E-21	0.953	2.24E-43	0.883	1.67E-28	1.070	2.75E-30	0.999	1.07E-32
GOBP_IMMUNE_RESPONSE	GO:0002764	1.205	1.56E-14	1.279	5.26E-15	1.071	5.47E-20	1.071	1.01E-31	1.197	2.15E-34	0.986	1.05E-41	1.031	1.19E-34	1.134	5.20E-46	1.097	1.87E-45	1.016	1.73E-35	1.088	4.64E-50	1.032	3.06E-42	1.029	2.98E-43	1.013	7.98E-44
GOBP_PROGRAMMED_CELL_DEATH	GO:0012501	1.199	1.57E-14	1.253	4.85E-16	1.171	1.21E-35	1.008	1.49E-54	0.858	1.01E-43	1.071	1.61E-62	1.126	6.12E-55	0.900	1.26E-44	0.948	4.04E-57	0.923	1.44E-56	0.932	8.21E-74	0.969	6.39E-84	0.982	2.70E-52	1.033	2.48E-47
GOBP_CELLULAR_RESPONSE_TO_STRESS	GO:0033554	1.239	1.74E-14	1.310	5.20E-19	0.961	3.29E-39	1.030	5.45E-33	1.028	2.73E-40	1.035	2.28E-50	1.084	2.92E-48	1.068	3.19E-49	1.017	2.70E-48	0.998	4.84E-52	1.003	7.08E-42	1.119	6.31E-49	1.008	3.08E-41	1.106	5.10E-64
GOCC_CATALYTIC_COMPLEX	GO:1902494	1.391	1.79E-14	1.451	6.16E-19	0.941	2.17E-30	1.169	5.63E-28	0.970	6.20E-31	0.937	4.14E-32	1.117	3.51E-41	1.075	1.05E-39	1.039	1.44E-43	1.090	2.63E-28	1.044	7.45E-44	0.966	2.43E-31	1.151	1.38E-37	0.948	2.47E-32
GOCC_SECRETORY_VESICLE	GO:0099503	1.232	2.20E-14	1.302	2.14E-10	1.111	1.08E-25	1.192	3.80E-27	1.182	3.32E-30	1.024	3.63E-25	1.119	8.70E-40	1.110	4.72E-39	1.213	9.35E-38	1.012	1.01E-25	1.216	2.55E-34	1.118	1.13E-35	1.122	4.51E-32	1.019	2.16E-32
GOBP_REGULATION_OF_CELLULAR_COMPONENT_MOVEMENT	GO:0051272	1.020	2.57E-14	0.983	9.86E-15	0.889	1.63E-23	0.907	4.59E-30	0.804	1.63E-32	0.884	5.77E-35	0.992	1.14E-40	0.806	1.99E-35	0.955	9.93E-36	0.872	7.40E-42	0.958	1.82E-65	0.869	5.67E-39	0.971	1.09E-30	0.957	8.74E-51
GOBP_ORGANONITROGEN_COMPOUND_CATABOLIC_PROCESS	GO:1901565	1.321	2.74E-14	1.341	6.87E-16	0.831	2.74E-34	1.293	1.40E-30	0.976	8.53E-33	0.914	5.91E-33	1.092	4.93E-39	1.003	5.33E-35	1.035	3.46E-45	1.023	2.12E-36	1.039	1.12E-37	0.996	4.25E-39	1.059	1.02E-31	0.971	6.00E-28
GOMF_TRANSITION_METAL_ION_BINDING	GO:0046914	1.005	3.53E-14	1.003	1.68E-17	1.135	5.65E-33	0.986	1.62E-25	0.956	2.35E-29	0.964	2.11E-31	0.978	3.67E-32	0.892	2.70E-32	0.998	1.06E-32	0.849	4.11E-24	0.986	2.24E-46	1.074	1.25E-30	1.000	3.33E-32	1.065	1.05E-33
GOCC_MEMBRANE_PROTEIN_COMPLEX	GO:0098796	1.095	4.17E-14	1.149	3.54E-16	1.028	6.17E-30	0.954	2.65E-34	1.055	1.13E-26	0.953	1.32E-37	0.940	6.70E-45	0.996	3.36E-41	0.910	8.77E-42	1.012	9.51E-44	0.901	2.90E-34	0.884	6.31E-28	0.969	2.06E-28	0.916	9.72E-24
GOBP_DEFENSE_RESPONSE_TO_OTHER_ORGANISM	GO:0098542	1.348	4.31E-14	1.400	3.84E-09	1.253	4.40E-14	1.334	8.97E-22	1.294	6.55E-18	1.158	2.62E-22	1.222	2.67E-30	1.370	1.44E-28	1.201	1.70E-24	1.052	2.51E-26	1.209	2.31E-29	1.157	5.78E-27	1.042	7.11E-23	1.073	4.10E-24
GOBP_NERVOUS_SYSTEM_PROCESS	GO:0050877	1.132	4.43E-14	1.143	9.75E-12	0.952	6.46E-28	0.946	1.40E-32	1.031	9.11E-39	1.101	4.31E-41	1.079	6.09E-33	1.007	2.01E-35	1.033	6.31E-39	0.990	7.75E-32	1.047	4.63E-44	0.800	3.45E-40	0.879	1.61E-38	0.878	1.92E-30
GOBP_REGULATION_OF_PROTEIN_PHOSPHORYLATION	GO:0001932	1.117	4.48E-14	1.177	6.45E-11	0.955	6.59E-21	0.947	5.58E-26	0.843	1.26E-28	1.035	2.79E-32	1.060	1.07E-30	0.883	5.45E-35	1.000	3.75E-44	0.974	4.22E-37	0.966	2.76E-43	0.871	9.56E-32	1.095	1.76E-33	1.008	8.09E-32
GOMF_TRANSFERASE_ACTIVITY_TRANSFERRING_PHOSPHORUS_CONTAINING_GROUPS	GO:0016772	0.981	5.30E-14	1.048	1.29E-08	0.770	1.14E-15	1.061	1.98E-26	0.823	8.67E-25	0.912	6.49E-33	0.900	2.39E-30	0.966	2.72E-30	0.906	2.30E-32	0.841	1.15E-37	0.913	6.96E-34	0.791	2.35E-26	0.911	3.49E-27	0.869	1.29E-26
GOBP_EMBRYO_DEVELOPMENT	GO:0009790	1.122	5.42E-14	1.088	8.86E-14	1.005	8.68E-32	1.124	1.31E-38	0.808	1.25E-29	0.990	3.74E-34	1.117	9.62E-42	1.027	3.22E-48	1.154	8.77E-54	1.032	7.44E-40	1.146	2.62E-58	1.033	4.02E-46	1.007	1.87E-37	0.989	2.86E-37
GOBP_RESPONSE_TO_NITROGEN_COMPOUND	GO:1901698	1.087	5.71E-14	1.146	5.16E-12	0.998	6.66E-25	1.065	5.07E-30	0.977	6.04E-30	1.013	6.49E-32	0.971	2.78E-30	0.987	2.33E-29	1.054	6.00E-38	1.035	6.52E-33	1.053	3.19E-39	0.842	7.05E-34	0.964	5.09E-23	0.976	2.69E-35
GOCC_GOLGI_APPARATUS	GO:0005794	1.061	5.78E-14	1.019	1.80E-15	1.086	1.47E-37	0.931	5.02E-43	0.985	1.69E-44	0.930	2.16E-36	0.945	1.45E-50	1.025	6.30E-58	0.992	8.38E-45	0.957	1.63E-47	0.994	1.37E-49	0.913	7.11E-49	0.911	1.73E-41	0.939	2.88E-32
GOBP_POSITIVE_REGULATION_OF_PROTEIN_METABOLIC_PROCESS	GO:0051247	1.147	7.64E-14	1.229	9.87E-16	0.997	3.62E-29	0.974	2.27E-29	0.855	7.40E-36	0.916	1.03E-43	1.101	7.06E-46	0.952	8.99E-46	1.010	3.29E-50	1.016	6.58E-30	0.999	1.89E-42	0.882	3.58E-45	1.046	2.60E-43	0.930	1.13E-53
GOBP_EPITHELIUM_DEVELOPMENT	GO:0060429	1.040	9.10E-14	1.062	6.27E-17	1.038	1.12E-33	0.991	6.10E-25	0.843	1.08E-38	0.954	3.52E-40	1.123	3.53E-36	0.925	1.66E-43	1.100	4.34E-40	0.950	1.10E-40	1.093	4.28E-59	1.027	8.29E-42	1.030	2.21E-35	1.119	9.89E-40
GOBP_PROTEIN_MODIFICATION_BY_SMALL_PROTEIN_CONJUGATION_OR_REMOVAL	GO:0070646	1.292	9.52E-14	1.302	3.91E-11	0.920	1.20E-24	1.083	7.41E-28	0.970	7.29E-26	0.952	2.98E-24	1.154	7.43E-25	1.170	3.98E-28	1.082	1.95E-27	1.164	7.40E-29	1.102	1.28E-28	1.000	4.78E-20	1.176	4.84E-27	0.993	4.95E-21
GOBP_VASCULATURE_DEVELOPMENT	GO:0001944	1.117	1.03E-13	1.151	8.46E-10	1.108	4.59E-19	1.126	4.89E-22	0.904	4.12E-26	0.930	4.42E-27	1.131	3.32E-39	0.987	2.61E-35	1.064	2.80E-31	0.919	7.31E-28	1.055	4.82E-41	1.012	5.03E-34	1.092	2.18E-22	1.038	1.97E-32
GOMF_PROTEIN_CONTAINING_COMPLEX_BINDING	GO:0044877	1.115	1.11E-13	1.148	5.31E-13	0.906	1.49E-31	0.970	5.89E-28	0.895	7.57E-38	0.914	2.03E-32	0.990	5.88E-41	0.969	1.17E-33	0.932	7.08E-40	0.973	3.08E-35	0.933	4.58E-35	0.944	1.21E-33	0.983	6.47E-30	1.055	1.49E-36
GOBP_BLOOD_VESSEL_MORPHOGENESIS	GO:0048514	1.092	1.41E-13	1.159	5.99E-08	1.146	2.20E-15	1.194	7.91E-20	0.950	1.01E-22	0.890	4.34E-23	1.166	5.33E-34	1.071	2.05E-33	1.107	2.14E-28	0.967	3.02E-24	1.097	2.04E-43	1.032	9.51E-26	1.186	2.62E-21	1.080	1.79E-25
GOBP_MRNA_METABOLIC_PROCESS	GO:0016071	1.148	1.83E-13	1.292	7.70E-09	0.989	4.60E-14	1.006	1.50E-16	0.974	5.55E-17	0.901	1.66E-17	1.125	4.31E-18	0.943	4.22E-19	0.909	5.73E-25	1.138	1.29E-17	0.940	3.26E-22	1.034	4.38E-20	1.064	6.16E-17	1.218	1.80E-20
GOCC_MITOCHONDRION	GO:0005739	1.356	2.09E-13	1.365	1.14E-16	1.031	4.30E-31	1.190	1.98E-32	1.038	1.43E-38	1.138	2.35E-46	1.023	1.61E-34	1.074	4.45E-39	1.050	3.01E-43	1.065	5.44E-36	1.051	2.60E-48	0.989	1.04E-31	1.167	2.86E-32	0.927	1.83E-38
GOBP_GROWTH	GO:0040007	0.974	2.52E-13	0.929	2.67E-15	1.100	1.61E-20	0.908	2.26E-26	0.868	2.99E-34	0.978	1.06E-35	1.068	2.37E-23	0.870	1.50E-24	0.967	7.09E-33	1.026	2.28E-35	0.971	2.24E-33	0.947	5.98E-25	0.841	4.20E-27	1.073	3.11E-33
GOMF_IDENTICAL_PROTEIN_BINDING	GO:0042802	1.250	2.79E-13	1.255	6.80E-23	1.094	1.19E-37	1.079	9.53E-57	1.059	2.49E-56	0.971	4.20E-46	0.990	1.83E-61	0.917	5.51E-59	0.928	2.63E-57	0.939	1.18E-63	0.933	1.17E-61	0.989	3.41E-47	0.987	3.66E-47	0.988	1.22E-55
GOBP_REPRODUCTION	GO:0044702	1.320	2.89E-13	1.425	3.98E-14	1.106	6.80E-27	1.042	8.49E-40	0.940	6.89E-35	1.033	6.36E-44	1.217	1.19E-44	1.136	1.30E-34	1.283	1.30E-52	1.104	9.57E-45	1.309	1.09E-39	1.236	2.02E-37	1.060	1.25E-33	1.249	8.03E-49
GOMF_TRANSCRIPTION_REGULATOR_ACTIVITY	GO:0140110	1.082	3.08E-13	1.010	3.34E-16	1.150	8.80E-25	1.053	8.81E-43	0.956	2.36E-43	1.012	3.94E-43	1.103	3.67E-50	0.957	8.31E-53	0.912	8.47E-64	0.984	7.38E-53	0.918	6.75E-68	1.017	2.24E-46	0.898	5.80E-37	0.965	4.04E-62
GOBP_CELLULAR_AMIDE_METABOLIC_PROCESS	GO:0043603	1.430	3.65E-13	1.486	3.01E-11	0.926	6.61E-29	1.242	1.04E-29	1.146	2.76E-27	1.088	2.61E-23	1.123	7.97E-30	0.879	2.05E-23	0.869	5.13E-33	1.099	4.35E-32	0.886	1.34E-34	1.170	7.71E-26	1.020	3.35E-24	1.167	8.07E-23
GOBP_MACROMOLECULE_CATABOLIC_PROCESS	GO:0009057	1.289	3.84E-13	1.329	1.37E-14	0.818	1.24E-31	1.221	3.04E-28	1.005	4.07E-33	0.881	3.14E-28	1.118	3.16E-31	1.047	1.09E-30	1.006	3.82E-39	1.113	6.01E-38	1.032	2.20E-39	1.046	3.22E-37	1.084	1.36E-27	1.081	1.43E-36
GOBP_REGULATION_OF_TRANSFERASE_ACTIVITY	GO:0051338	1.129	4.80E-13	1.184	1.16E-10	0.930	6.91E-29	0.982	3.66E-24	0.839	2.79E-21	1.052	1.25E-25	1.119	1.72E-29	0.990	8.91E-29	1.073	1.96E-29	0.947	2.93E-35	1.041	8.72E-31	0.964	1.13E-30	1.122	4.26E-30	1.064	1.20E-30
GOBP_INTRACELLULAR_PROTEIN_TRANSPORT	GO:0006886	1.224	5.11E-13	1.262	3.45E-09	0.908	1.31E-23	1.131	2.12E-21	1.089	1.76E-23	0.929	1.22E-19	1.030	4.74E-27	0.898	3.06E-22	1.053	9.71E-29	0.940	1.44E-22	1.075	1.17E-25	1.041	2.16E-23	1.004	4.08E-21	0.894	1.49E-24
GOBP_NEGATIVE_REGULATION_OF_GENE_EXPRESSION	GO:0010629	1.164	5.51E-13	1.249	5.43E-09	1.091	7.91E-24	1.159	1.09E-21	1.039	3.40E-31	0.961	2.02E-30	1.243	7.04E-24	1.085	5.84E-26	1.106	6.44E-30	1.095	1.16E-23	1.090	2.88E-29	0.994	1.15E-28	1.032	4.01E-27	1.087	1.11E-32
GOBP_POSITIVE_REGULATION_OF_CELLULAR_COMPONENT_ORGANIZATION	GO:0051130	1.031	5.68E-13	1.025	1.16E-13	0.983	4.18E-22	0.955	5.22E-29	0.912	1.77E-36	0.973	8.68E-34	0.904	3.89E-33	0.838	1.09E-33	0.927	1.52E-30	0.849	2.47E-32	0.934	1.52E-42	0.860	6.67E-34	0.907	2.06E-32	0.875	2.28E-23
GOBP_CYTOSKELETON_ORGANIZATION	GO:0007010	1.120	6.02E-13	1.142	2.53E-13	0.783	2.66E-35	0.918	1.90E-37	0.921	1.10E-42	0.842	1.42E-37	0.831	8.16E-65	0.950	6.75E-42	0.995	1.43E-47	0.887	9.47E-57	1.006	3.18E-58	0.887	1.14E-54	0.969	1.94E-49	0.943	9.10E-52
GOCC_ANCHORING_JUNCTION	GO:0070161	0.962	6.52E-13	0.990	4.56E-11	0.967	1.74E-28	0.812	1.11E-23	0.781	3.67E-26	0.750	4.24E-32	0.821	2.14E-29	0.810	3.42E-43	0.879	2.78E-28	0.813	6.08E-38	0.905	5.69E-30	0.807	4.55E-30	0.812	1.28E-39	0.825	5.75E-32
GOBP_ANIMAL_ORGAN_MORPHOGENESIS	GO:0009887	1.121	8.28E-13	1.063	6.46E-14	1.142	8.34E-23	0.944	4.42E-28	0.754	1.77E-33	0.916	4.05E-40	1.062	5.97E-39	0.959	5.55E-47	0.996	8.41E-48	1.015	3.68E-39	1.000	2.39E-57	0.969	2.18E-45	0.969	1.92E-37	1.063	5.82E-38
GOCC_NUCLEAR_OUTER_MEMBRANE_ENDOPLASMIC_RETICULUM_MEMBRANE_NETWORK	GO:0042175	1.289	8.65E-13	1.332	1.70E-15	0.858	2.47E-26	1.102	1.02E-30	1.008	1.06E-22	1.069	2.21E-24	1.005	4.62E-33	1.050	5.01E-32	1.018	6.24E-29	0.954	1.75E-30	1.039	3.75E-36	0.961	7.60E-37	1.082	3.66E-36	0.915	1.39E-31
GOBP_RESPONSE_TO_ORGANIC_CYCLIC_COMPOUND	GO:0014070	1.079	8.96E-13	1.149	4.20E-08	1.108	8.25E-29	1.010	2.01E-24	0.950	2.31E-25	1.117	2.05E-29	1.007	7.84E-22	0.965	9.92E-23	1.005	3.58E-27	0.950	3.22E-35	1.020	5.90E-38	0.934	1.24E-27	1.055	6.53E-27	1.009	1.82E-27
GOBP_MICROTUBULE_BASED_PROCESS	GO:0007017	1.333	1.12E-12	1.451	9.32E-11	1.092	4.37E-14	1.057	5.12E-31	1.154	1.66E-27	0.973	3.71E-22	1.082	3.65E-35	1.231	2.98E-31	1.236	8.77E-27	1.162	5.02E-25	1.251	6.49E-32	1.026	3.29E-32	1.049	4.30E-22	0.967	9.77E-26
GOMF_PROTEIN_DOMAIN_SPECIFIC_BINDING	GO:0019904	1.041	1.13E-12	1.064	1.11E-09	0.855	3.93E-19	0.982	5.21E-23	0.844	9.55E-23	0.861	4.51E-25	0.861	1.60E-21	0.896	6.16E-23	1.044	1.95E-30	0.885	8.20E-21	1.049	2.37E-23	0.820	1.05E-24	0.765	5.21E-20	0.800	1.54E-25
GOBP_IMMUNE_SYSTEM_DEVELOPMENT	GO:0002520	1.220	1.21E-12	1.380	4.60E-12	1.008	5.03E-26	1.103	8.22E-32	1.025	4.20E-27	0.969	1.91E-26	1.027	4.75E-27	1.023	4.75E-39	0.994	2.78E-44	0.908	1.55E-29	0.969	4.59E-29	0.936	6.73E-33	0.962	1.24E-32	0.877	9.51E-25
GOBP_SMALL_GTPASE_MEDIATED_SIGNAL_TRANSDUCTION	GO:0051056	0.864	1.35E-12	0.919	6.37E-09	0.597	1.72E-13	0.927	2.34E-15	0.694	1.71E-17	0.875	9.66E-19	0.700	8.98E-17	0.880	7.39E-26	0.929	2.20E-22	0.840	1.46E-22	0.962	2.47E-27	0.783	5.63E-16	1.030	2.49E-18	0.747	1.01E-19
GOMF_SEQUENCE_SPECIFIC_DNA_BINDING	GO:0043565	1.170	1.39E-12	1.079	3.02E-13	1.131	2.18E-23	1.090	8.59E-42	0.980	9.38E-41	1.113	3.43E-34	1.126	1.08E-46	0.983	1.83E-43	0.928	1.81E-47	1.070	7.27E-52	0.943	1.08E-63	1.044	2.48E-44	0.933	4.69E-42	1.015	1.07E-40

GO Name	GO ID	Infection		Hospitalization		Severe Symptoms		IDP2834		IDP2831		IDP2195		IDP1985		IDP1274		IDP1160		IDP1115		IDP1069		IDP0957		IDP0910		IDP0766	
		enrich	p	enrich	p	enrich	p	enrich	p	enrich	p	enrich	p	enrich	p	enrich	p	enrich	p	enrich	p	enrich	p	enrich	p	enrich	P	enrich	p
GOBP_REGULATION_OF_CELLULAR_COMPONENT_BIOGENESIS	GO:0044089	1.022	1.44E-12	1.017	1.38E-12	0.762	7.82E-27	1.043	7.35E-33	0.908	1.62E-32	1.017	7.83E-30	0.861	2.88E-27	0.890	3.27E-27	0.875	7.56E-27	0.838	2.22E-29	0.880	5.84E-31	0.848	5.92E-31	0.753	2.69E-23	0.857	1.34E-21
GOBP_CELL_CELL_ADHESION	GO:0098609	0.921	1.46E-12	0.950	5.93E-12	1.036	1.85E-26	0.728	4.76E-33	0.765	4.79E-30	0.788	6.78E-32	0.965	9.83E-29	0.820	8.48E-34	0.862	1.59E-42	0.801	1.61E-31	0.877	3.10E-40	0.738	2.08E-28	0.750	1.45E-34	0.796	1.71E-42
GOBP_CELL_MORPHOGENESIS_INVOLVED_IN_DIFFERENTIATION	GO:0000904	0.893	1.53E-12	0.908	3.76E-10	0.932	9.74E-21	0.775	3.08E-19	0.839	2.84E-32	0.844	2.53E-36	0.905	1.46E-27	0.743	1.56E-22	0.916	1.02E-31	0.811	1.07E-31	0.934	8.09E-35	0.841	3.53E-32	0.926	5.57E-35	0.990	5.24E-25
GOBP_CELL_MORPHOGENESIS_INVOLVED_IN_NEURON_DIFFERENTIATION	GO:0048667	0.842	1.71E-12	0.854	6.55E-10	1.062	2.83E-17	0.757	2.36E-18	0.829	7.27E-27	0.877	1.10E-27	0.977	1.05E-23	0.759	4.77E-19	0.922	2.37E-26	0.807	1.70E-27	0.949	4.17E-27	0.832	4.34E-29	0.949	1.66E-28	1.002	9.11E-21
GOBP_NUCLEOBASE_CONTAINING_SMALL_MOLECULE_METABOLIC_PROCESS	GO:0055086	1.178	2.12E-12	1.142	2.32E-08	0.941	1.48E-13	1.229	4.10E-15	1.016	3.08E-14	1.015	3.14E-18	1.035	2.70E-19	1.037	4.03E-16	0.895	1.10E-20	0.933	2.75E-15	0.884	1.74E-15	1.156	3.56E-22	0.925	3.52E-15	1.089	4.29E-14
GOBP_INORGANIC_ION_TRANSMEMBRANE_TRANSPORT	GO:0098660	1.056	2.18E-12	1.081	1.62E-10	1.078	1.81E-26	1.077	1.33E-22	1.103	1.95E-28	1.131	1.65E-29	0.929	1.19E-24	1.090	3.44E-30	1.033	1.49E-32	1.084	2.44E-24	1.036	6.07E-31	0.877	5.81E-25	0.901	2.03E-24	0.857	5.14E-19
GOBP_CELL_ACTIVATION	GO:0001775	1.069	2.30E-12	1.253	2.23E-09	0.918	4.82E-26	1.060	1.05E-24	1.022	6.94E-29	1.107	2.19E-28	0.978	3.54E-25	1.002	1.13E-32	1.011	2.46E-36	0.928	5.68E-24	0.990	5.26E-30	0.866	4.36E-31	0.999	5.74E-29	0.847	1.17E-32
GOBP_CATION_TRANSMEMBRANE_TRANSPORT	GO:0098662	1.106	2.84E-12	1.122	2.09E-11	1.054	1.59E-27	0.996	1.06E-22	1.149	3.99E-31	1.172	9.88E-32	0.919	2.78E-27	1.052	1.52E-28	0.958	5.65E-36	0.996	6.91E-24	0.955	5.35E-30	0.802	5.56E-25	0.898	3.35E-27	0.806	8.23E-24
GOBP_DNA_METABOLIC_PROCESS	GO:0006259	1.350	3.67E-12	1.457	8.36E-12	0.858	3.87E-33	1.156	4.10E-19	1.050	2.37E-18	1.261	1.22E-20	1.159	1.50E-25	1.125	4.20E-20	1.025	7.77E-21	1.126	4.01E-21	1.064	4.41E-22	1.342	6.91E-35	1.107	7.53E-21	1.273	6.35E-24
GOBP_PROTEIN_LOCALIZATION_TO_ORGANELLE	GO:0033365	1.169	3.67E-12	1.189	2.57E-10	0.951	3.84E-18	1.026	7.66E-19	0.965	1.37E-22	1.076	4.55E-23	0.912	2.76E-23	1.005	4.22E-25	1.019	3.41E-23	0.919	1.80E-18	1.028	1.23E-25	0.929	1.01E-22	0.949	5.74E-21	0.909	5.23E-23
GOBP_TRANSMEMBRANE_RECEPTOR_PROTEIN_TYROSINE_KINASE_SIGNALING_PATHWAY	GO:0007169	1.044	3.85E-12	1.065	4.53E-10	0.881	1.32E-16	1.031	4.63E-22	0.802	2.55E-16	0.939	4.57E-31	1.045	1.41E-26	0.953	6.10E-21	1.016	1.83E-28	1.033	9.40E-34	0.984	6.84E-28	0.809	1.24E-30	1.099	6.15E-26	0.858	2.65E-22
GOBP_REGULATION_OF_CELL_DEATH	GO:0008219	1.252	3.95E-12	1.290	5.97E-13	1.143	6.69E-34	1.024	1.52E-42	0.929	7.20E-37	1.088	1.84E-48	1.137	1.93E-41	0.936	5.89E-42	0.985	3.57E-42	0.928	3.46E-44	0.969	3.52E-52	1.015	4.19E-55	0.967	3.37E-41	1.034	7.32E-50
GOBP_CHROMOSOME_ORGANIZATION	GO:0051276	1.221	3.99E-12	1.308	2.61E-12	0.943	8.20E-25	1.074	9.64E-22	0.963	9.09E-26	1.116	1.78E-32	0.945	1.14E-25	1.192	2.08E-26	1.028	7.11E-25	1.114	2.38E-19	1.040	2.74E-28	1.153	9.09E-34	1.121	1.84E-27	1.061	3.90E-31
GOBP_SYNAPTIC_SIGNALING	GO:0099536	0.917	4.08E-12	1.018	3.03E-07	0.955	9.21E-27	0.798	1.55E-30	0.951	8.90E-27	0.879	1.84E-37	0.960	3.75E-27	0.935	7.24E-27	0.959	1.72E-27	0.941	1.15E-26	0.771	5.64E-29	0.908	4.76E-25	0.905	1.33E-30		
GOBP_LIPID_METABOLIC_PROCESS	GO:0006629	1.172	4.23E-12	1.155	5.61E-15	0.950	2.54E-35	1.213	3.43E-37	1.047	5.55E-32	1.216	1.46E-33	1.187	7.31E-42	1.048	4.67E-36	1.019	5.02E-44	0.997	4.37E-53	1.008	4.21E-43	0.905	3.40E-32	1.081	3.34E-40	0.929	3.87E-39
GOBP_AXON_DEVELOPMENT	GO:0061564	0.790	6.00E-12	0.830	3.53E-08	1.195	1.45E-15	0.785	1.24E-16	0.886	1.63E-27	0.897	2.39E-25	1.065	3.15E-20	0.794	2.78E-18	1.017	2.79E-23	0.900	8.63E-24	1.047	1.37E-24	0.898	6.53E-22	0.959	4.56E-26	1.050	1.94E-16
GOBP_REGULATION_OF_CELLULAR_MACROMOLECULE_BIOSYNTHETIC_PROCESS	GO:2000112	1.397	6.17E-12	1.361	1.68E-07	1.021	6.01E-23	1.189	8.85E-18	1.065	1.21E-17	1.085	3.80E-15	1.245	1.33E-23	1.144	5.73E-17	1.061	6.50E-23	1.201	2.02E-19	1.091	1.77E-22	1.247	2.00E-20	1.039	8.54E-20	1.296	2.95E-23
GOBP_RESPONSE_TO_HORMONE	GO:0009725	1.100	6.28E-12	1.067	4.64E-11	1.024	1.01E-26	1.041	4.36E-33	0.873	4.25E-27	1.126	7.71E-29	0.961	2.02E-21	0.911	6.05E-25	1.005	2.78E-28	0.964	4.67E-32	1.004	8.30E-32	0.899	4.07E-26	0.928	2.48E-26	0.985	3.66E-34
GOBP_AMIDE_BIOSYNTHETIC_PROCESS	GO:0043604	1.424	6.46E-12	1.557	2.96E-09	0.945	7.30E-24	1.162	6.73E-21	1.080	2.62E-25	1.081	4.93E-17	1.091	1.45E-22	0.986	6.92E-19	0.959	2.44E-26	1.133	2.74E-23	0.981	9.29E-23	1.216	6.61E-21	0.998	5.99E-19	1.265	4.91E-16
GOCC_NUCLEAR_PROTEIN_CONTAINING_COMPLEX	GO:0140513	1.350	6.52E-12	1.460	1.01E-16	0.931	2.17E-33	1.020	1.45E-28	0.911	5.46E-34	1.010	2.14E-28	1.056	2.03E-37	1.058	2.51E-30	1.004	8.67E-40	1.077	1.03E-26	1.002	2.39E-31	1.040	2.39E-33	1.047	8.27E-28	0.997	4.32E-31
GOBP_REGULATION_OF_ION_TRANSPORT	GO:0043269	0.990	7.94E-12	1.010	1.73E-09	1.057	3.65E-19	0.882	2.22E-17	1.275	6.16E-34	1.006	5.81E-26	0.924	2.33E-17	0.935	1.24E-22	0.977	4.79E-27	1.001	3.00E-24	0.967	3.24E-24	0.786	1.91E-21	0.943	1.19E-21	0.885	5.28E-19
GOBP_TAXIS	GO:0042330	0.955	8.00E-12	0.971	1.43E-07	1.279	5.83E-14	0.999	2.06E-19	0.838	7.21E-26	0.928	7.54E-25	1.115	2.82E-23	0.831	7.89E-21	0.996	1.45E-30	0.956	8.80E-23	1.003	5.40E-34	0.981	1.19E-22	0.952	1.82E-26	1.126	6.72E-24
GOBP_CHEMICAL_HOMEOSTASIS	GO:0048878	1.178	9.08E-12	1.191	5.40E-12	1.102	5.83E-26	1.012	4.59E-30	1.010	3.18E-31	1.102	2.37E-35	0.945	1.35E-29	0.962	4.91E-30	1.038	5.91E-33	1.008	3.73E-34	1.052	7.52E-36	0.903	6.96E-35	0.965	4.03E-38	0.873	2.21E-28
GOBP_ORGANELLE_LOCALIZATION	GO:0051640	1.042	1.01E-11	1.150	7.56E-10	1.197	9.80E-10	0.974	2.04E-17	0.969	1.66E-17	0.882	4.26E-15	0.862	4.07E-19	1.016	4.41E-18	0.994	7.21E-20	1.059	4.47E-15	1.014	2.16E-14	1.135	1.10E-20	1.107	1.71E-17	1.113	5.46E-16
GOBP_POSITIVE_REGULATION_OF_PHOSPHORUS_METABOLIC_PROCESS	GO:0010562	1.085	1.06E-11	1.165	3.31E-09	0.982	6.26E-16	0.879	5.04E-24	0.756	8.13E-20	0.944	2.65E-35	1.165	6.10E-27	0.892	1.49E-38	1.052	1.54E-32	0.922	2.00E-30	1.023	1.59E-39	0.888	4.06E-30	1.101	1.54E-28	0.994	1.47E-30
GOBP_MRNA_PROCESSING	GO:0006397	1.171	1.38E-11	1.407	5.28E-07	1.089	1.66E-09	0.981	7.58E-10	1.084	5.92E-13	0.948	6.96E-13	1.314	5.72E-12	1.048	4.90E-12	1.023	9.01E-15	1.105	2.63E-11	1.051	7.26E-16	0.939	3.27E-11	1.134	2.50E-12	1.021	1.58E-12
GOBP_CENTRAL_NERVOUS_SYSTEM_DEVELOPMENT	GO:0007417	0.986	1.40E-11	1.043	2.27E-09	0.918	1.79E-47	0.923	5.26E-34	0.875	1.77E-38	0.949	2.88E-38	1.027	4.29E-28	0.914	3.36E-43	1.055	1.39E-39	0.915	1.54E-33	1.057	1.85E-67	0.993	1.68E-38	0.954	1.47E-58	1.064	2.20E-40
GOBP_POSITIVE_REGULATION_OF_IMMUNE_SYSTEM_PROCESS	GO:0002684	1.080	1.49E-11	1.176	1.08E-08	0.929	4.37E-13	1.196	9.50E-18	1.127	1.71E-20	1.072	2.56E-23	0.931	2.56E-15	1.026	6.80E-23	1.012	8.68E-27	1.072	1.50E-23	1.026	6.80E-23	1.052	1.56E-26	0.925	9.71E-25	0.964	2.06E-26
GOBP_CELLULAR_HOMEOSTASIS	GO:0019725	1.141	1.50E-11	1.200	5.42E-09	1.111	1.26E-18	0.968	1.46E-22	1.054	1.11E-28	1.137	2.81E-25	0.980	3.44E-29	0.909	5.02E-20	0.983	5.07E-35	0.993	2.07E-25	0.995	2.99E-27	0.738	2.56E-27	1.001	2.14E-29	0.799	1.65E-19
GOMF_ION_TRANSMEMBRANE_TRANSPORTER_ACTIVITY	GO:0015075	1.125	1.64E-11	1.146	4.42E-11	1.019	5.20E-28	1.058	7.54E-26	1.379	1.64E-30	1.332	4.36E-34	0.990	4.81E-26	1.093	2.57E-30	1.021	1.14E-34	1.003	1.60E-26	1.018	3.29E-29	0.930	9.29E-27	0.944	1.67E-21	0.953	3.82E-23
GOBP_POSITIVE_REGULATION_OF_TRANSPORT	GO:0051050	1.024	1.67E-11	1.081	1.37E-10	0.871	2.11E-23	1.075	2.06E-29	0.967	5.74E-30	0.949	4.64E-23	0.896	4.08E-23	0.996	3.01E-35	1.062	2.05E-26	1.109	1.38E-37	1.056	2.89E-33	1.026	4.67E-37	0.960	3.29E-24	1.011	2.41E-18
GOBP_EMBRYO_DEVELOPMENT_ENDING_IN_BIRTH_OR_EGG_HATCHING	GO:0009792	1.181	1.76E-11	1.169	1.11E-10	1.088	5.08E-23	1.071	4.58E-28	0.839	2.14E-18	0.959	4.62E-21	1.142	6.48E-22	1.124	1.34E-28	1.252	1.82E-26	1.128	3.90E-29	1.235	1.81E-38	1.158	7.83E-32	0.940	2.60E-22	1.086	2.67E-26
GOBP_PROTEIN_CONTAINING_COMPLEX_ORGANIZATION	GO:0043933	1.290	1.93E-11	1.307	1.12E-10	0.858	6.32E-28	0.974	3.89E-30	1.020	6.14E-38	1.107	4.35E-50	0.944	2.66E-47	1.063	6.56E-47	0.923	8.82E-43	0.940	1.82E-48	0.924	1.27E-38	0.909	4.07E-37	0.936	3.39E-44	0.907	7.76E-47
GOBP_REGULATION_OF_RESPONSE_TO_STRESS	GO:0080134	1.265	1.94E-11	1.322	5.08E-13	0.988	6.82E-22	1.130	4.68E-26	1.025	2.45E-31	1.101	7.04E-39	1.154	7.35E-37	1.070	6.00E-34	1.090	2.96E-41	1.005	2.24E-32	1.089	7.03E-37	1.108	1.16E-34	1.053	7.67E-37	1.056	1.52E-41
GOBP_REGULATION_OF_CELL_PROJECTION_ORGANIZATION	GO:0031344	0.957	2.03E-11	1.015	1.29E-09	0.991	2.31E-18	0.792	2.02E-21	0.972	3.99E-28	1.006	3.89E-25	0.892	2.78E-20	0.827	8.76E-19	0.873	3.30E-23	0.841	1.94E-23	0.880	3.40E-28	0.879	2.34E-25	0.858	1.01E-24	0.910	1.70E-19
GOBP_REGULATION_OF_GROWTH	GO:0040008	1.013	2.07E-11	1.003	7.51E-11	1.200	8.93E-16	0.871	3.96E-15	0.968	1.45E-23	1.016	1.46E-22	1.156	1.32E-17	0.896	9.46E-17	1.105	2.84E-23	1.089	1.56E-23	1.108	1.52E-27	1.077	3.97E-17	0.865	2.11E-19	1.160	3.33E-22
GOBP_PROTEIN_MODIFICATION_BY_SMALL_PROTEIN_CONJUGATION	GO:0032446	1.294	2.18E-11	1.293	1.58E-09	0.907	3.56E-22	0.986	2.14E-20	0.937	4.50E-22	0.940	9.91E-24	1.163	5.12E-24	1.185	9.55E-26	1.126	5.25E-25	1.143	2.10E-24	1.152	8.07E-24	1.019	5.45E-20	1.202	3.22E-24	0.989	5.08E-19
GOCC_NUCLEAR_BODY	GO:0016604	1.006	2.23E-11	1.155	1.35E-10	0.909	8.36E-21	0.931	5.67E-21	0.860	1.15E-19	0.902	3.77E-22	0.863	9.33E-22	0.887	1.78E-17	0.912	2.87E-25	0.950	5.52E-28	0.917	7.66E-25	0.810	1.20E-19	0.922	6.29E-19	0.954	4.88E-27
GOBP_POSITIVE_REGULATION_OF_CELL_DIFFERENTIATION	GO:0045597	1.151	2.50E-11	1.115	1.07E-08	1.024	4.48E-27	1.008	3.41E-28	0.904	9.18E-24	1.155	3.60E-29	1.020	3.80E-20	0.945	5.79E-35	1.034	1.38E-27	1.028	8.67E-27	1.014	1.80E-40	1.023	2.02E-35	0.831	9.72E-28	0.981	8.09E-35
GOBP_METAL_ION_TRANSPORT	GO:0030001	1.022	2.82E-11	1.010	9.75E-12	1.069	4.34E-26	1.089	1.96E-23	1.187	7.13E-33	1.095	4.51E-35	0.981	5.12E-21	1.018	9.65E-30	1.026	1.06E-33	1.066	7.92E-26	1.019	1.76E-31	0.810	2.34E-29	0.897	1.48E-25	0.808	2.77E-19
GOBP_NEGATIVE_REGULATION_OF_BIOSYNTHETIC_PROCESS	GO:0009890	1.161	3.16E-11	1.105	6.42E-13	1.163	8.25E-25	1.093	1.01E-41	1.023	5.14E-37	1.019	1.65E-37	1.132	1.86E-39	1.088	9.53E-65	0.944	1.15E-51	1.018	1.52E-46	0.946	2.08E-56	1.029	5.02E-39	0.952	4.22E-41	1.088	6.88E-49
GOBP_HEAD_DEVELOPMENT	GO:0060322	0.939	3.31E-11	0.967	1.18E-08	0.972	9.35E-34	0.978	2.41E-29	0.872	6.85E-35	0.922	1.32E-30	1.040	7.77E-24	0.893	1.11E-27	1.038	1.24E-35	0.903	5.74E-33	1.046	2.94E-45	0.935	1.83E-30	0.921	6.99E-37	1.032	6.44E-35
GOBP_MAPK_CASCADE	GO:0000165	1.074	3.68E-11	1.097	5.68E-09	1.159	2.68E-18	1.125	9.45E-20	0.846	3.60E-17	1.086	1.03E-28	1.039	2.25E-28	1.015	2.64E-24	1.135	4.38E-35	1.008	2.24E-28	1.096	1.08E-33	1.020	2.10E-24	1.139	5.22E-22	1.071	4.37E-30
GOBP_CELLULAR_PROTEIN_CATABOLIC_PROCESS	GO:0044257	1.330	4.42E-11	1.409	1.07E-09	0.862	1.53E-19	1.255	2.11E-19	0.990	2.23E-23	0.947	9.16E-18	1.036	1.36E-20	1.011	7.05E-17	1.022	3.15E-26	1.008	5.29E-25	1.032	4.92E-25	0.976	3.28E-23	1.136	1.73E-21	0.946	3.33E-18
GOBP_POSITIVE_REGULATION_OF_CELL_POPULATION_PROLIFERATION	GO:0008284	1.116	4.44E-11	1.162	7.52E-10	1.134	1.70E-22	1.160	1.05E-25	0.956	3.22E-26	1.120	1.76E-30	1.186	1.22E-24	1.047	4.32E-32	0.983	1.04E-27	1.091	1.38E-27	0.969	2.38E-31	1.000	5.67E-36	1.000	2.18E-25	1.068	2.64E-25
GOBP_CARBOHYDRATE_DERIVATIVE_METABOLIC_PROCESS	GO:1901135	1.192	4.72E-11	1.129	4.75E-13	0.874	7.53E-28	1.160	5.20E-28	1.027	8.64E-26	1.005	5.22E-46	0.991	7.95E-28	1.076	7.45E-32	1.029	1.89E-45	1.021	1.05E-34	1.035	1.35E-34	1.090	3.86E-32	1.010	1.04E-27	1.089	1.64E-21
GOMF_KINASE_ACTIVITY	GO:0016301	0.931	5.20E-11	0.983	8.89E-08	0.736	6.47E-14	0.962	2.43E-24	0.833	1.92E-21	0.924	2.84E-27	0.906	2.74E-28	0.943	4.85E-26	0.902	2.13E-28	0.807	1.26E-30	0.903	1.05E-28	0.767	1.29E-23	0.874	7.99E-23	0.852	2.04E-22
GOCC_VESICLE_MEMBRANE	GO:0012506	1.128	6.38E-11	1.214	3.11E-12	0.862	3.21E-38	1.064	9.21E-37	1.120	8.29E-35	0.946	3.01E-37	1.033	9.69E-43	1.112	5.42E-37	1.147	1.72E-36	1.022	4.24E-39	1.148	4.70E-40	1.071	1.19E-37	1.082	7.01E-33	1.012	5.41E-22
GOCC_SECRETORY_GRANULE	GO:0030141	1.300	6.55E-11	1.382	7.66E-09	1.197	1.05E-21	1.303	7.30E-24	1.142	1.46E-28	1.020	4.85E-19	1.117	1.51E-36	1.157	5.53E-35	1.181	1.04E-28	1.081	6.47E-22	1.172	1.41E-27	1.135	3.27E-31	1.093	9.19E-27	1.057	9.25E-27
GOBP_REGULATION_OF_CELLULAR_LOCALIZATION	GO:0060341	1.144	7.22E-11	1.242	4.31E-09	0.911	7.13E-30	1.034	3.04E-19	0.906	5.90E-21	0.986	6.69E-19	0.824	9.12E-26	0.854	5.09E-25	0.956	7.06E-28	0.927	1.50E-32	0.969	3.71E-23	0.799	2.72E-28	0.914	1.61E-24	0.798	4.77E-27
GOBP_DEVELOPMENTAL_GROWTH	GO:0048589	0.890	7.23E-11	0.835	2.07E-11	1.171	6.09E-17	0.822	1.56E-21	0.816	1.07E-22	0.934	6.75E-28	1.061	1.64E-18	0.822	3.63E-18	0.988	6.21E-23	1.047	4.01E-27	0.999	5.47E-27	0.891	4.39E-18	0.855	2.83E-21	1.062	5.12E-26
GOCC_CHROMATIN	GO:0000785	1.236	7.56E-11	1.208	4.94E-12	1.118	4.65E-20	0.984	2.00E-32	0.879	8.61E-29	1.026	2.80E-26	0.911	9.29E-29	0.877	8.75E-59	0.965	4.68E-42	0.905	9.16E-49	0.964	1.56E-40	0.858	5.85E-30	0.955	1.22E-46		
GOBP_INNATE_IMMUNE_RESPONSE	GO:0045087	1.367	7.60E-11	1.390	2.06E-08	1.253	6.04E-11	1.452	2.25E-17	1.325	1.78E-14	1.151	5.77E-19	1.221	1.18E-21	1.424	6.03E-21	1.274	4.20E-20	1.063	5.17E-26	1.280	2.80E-23	1.115	6.02E-18	1.000	8.72E-17	1.072	1.90E-21
GOMF_INORGANIC_MOLECULAR_ENTITY_TRANSMEMBRANE_TRANSPORTER_ACTIVITY	GO:0015318	1.077	7.66E-11	1.118	4.98E-09	1.093	1.38E-24	1.070	5.55E-22	1.332	3.70E-27	1.336	4.07E-32	0.955	2.59E-23	1.077	1.71E-23	1.018	1.07E-29	0.977	2.61E-22	1.020	6.70E-27	0.903	1.35E-19	0.925	4.75E-19	0.908	3.03E-16
GOBP_RNA_PROCESSING	GO:0006396	1.249	9.31E-11	1.354	4.49E-11	0.948	1.24E-17	1.059	1.38E-17	0.964	5.97E-28	1.068	1.16E-24	1.140	1.18E-19	1.096	2.78E-24	1.060	1.22E-23	1.102	1.46E-19	1.091	2.34E-24	1.038	4.00E-22	1.056	1.30E-20	1.046	4.65E-23
GOCC_VACUOLE	GO:0005773	1.292	9.40E-11	1.277	5.74E-10	1.128	2.77E-17	1.194	3.64E-22	1.208	3.47E-22	0.947	9.32E-22	1.225	6.17E-20	1.226	9.39E-30	1.070	6.27E-21	1.219	1.86E-29	1.000	2.13E-20	1.076	2.59E-21	0.997	2.96E-14		
GOCC_ACTIN_CYTOSKELETON	GO:0015629	0.929	1.13E-10	0.908	9.57E-09	0.727	4.75E-18	0.981	1.25E-16	0.854	1.30E-13	0.659	1.14E-13	0.714	8.89E-23	0.828	8.97E-24	0.809	3.67E-23	0.788	1.41E-18	0.793	3.51E-20	0.831	2.93E-18	0.837	1.83E-21	0.910	4.99E-20
GOMF_ZINC_ION_BINDING	GO:0008270	1.012	1.22E-10	1.011	2.88E-12	1.201	2.05E-24	0.976	1.44E-21	0.837	7.92E-23	0.977	2.71E-26	0.925	7.76E-24	0.864	6.69E-25	0.889	1.84E-22	0.801	2.88E-18	0.880	6.39E-31	1.102	1.18E-25	0.998	8.24E-25	1.049	1.80E-25
GOBP_REGULATION_OF_PROTEIN_KINASE_ACTIVITY	GO:0045860	1.084	1.22E-10	1.144	1.13E-07	0.960	6.58E-23	0.998	1.81E-16	0.884	1.55E-18	1.038	3.84E-20	1.141	2.23E-23	0.935	2.16E-24	0.967	6.04E-28	0.932	6.86E-27	0.927	2.28E-26	0.887	4.40E-22	1.109	1.45E-25	0.971	1.58E-21
GOBP_NEGATIVE_REGULATION_OF_CATALYTIC_ACTIVITY	GO:0043086	1.112	1.39E-10	1.197	8.91E-13	0.799	1.24E-15	1.059	1.32E-14	1.066	2.03E-19	0.995	2.83E-21	1.066	1.22E-20	1.014	1.09E-21	1.231	3.73E-24	1.039	1.60E-23	1.236	5.96E-20	0.963	1.39E-20	0.966	6.14E-20	0.829	1.20E-17
GOBP_REGULATION_OF_CATABOLIC_PROCESS	GO:0009894	1.115	1.40E-10	1.145	1.78E-13	1.014	1.99E-18	1.173	9.11E-18	0.928	1.35E-21	0.905	1.24E-31	1.043	3.20E-28	0.934	2.39E-26	0.923	3.15E-21	0.978	1.27E-22	0.945	3.27E-27	0.876	2.82E-29	0.980	1.03E-22	0.901	7.47E-23
GOMF_KINASE_BINDING	GO:0019900	1.203	1.45E-10	1.220	2.69E-10	0.935	3.00E-24	0.917	3.90E-20	0.928	1.23E-21	0.804	4.64E-23	0.943	4.55E-24	0.856	1.98E-24	0.841	2.62E-21	0.919	4.59E-26	0.860	5.85E-18	0.829	2.58E-24	1.007	1.26E-23	0.840	1.27E-21
GOCC_SOMATODENDRITIC_COMPARTMENT	GO:0036477	0.957	1.50E-10	0.982	2.44E-10	0.831	4.60E-27	0.994	3.41E-42	0.869	7.02E-33	1.030	4.59E-36	0.934	3.19E-34	0.940	6.83E-26	1.028	1.17E-32	1.034	9.45E-33	1.026	1.38E-25	0.785	9.78E-25	0.921	1.93E-27	0.922	3.45E-30
GOMF_MOLECULAR_TRANSDUCER_ACTIVITY	GO:0060089	1.108	1.63E-10	1.155	2.37E-10	1.384	2.08E-12	0.952	2.73E-30	0.963	9.30E-28	1.042	1.57E-55	0.992	1.11E-34	0.963	2.52E-31	0.962	6.23E-40	0.996	1.92E-31	0.956	1.49E-36	1.021	7.57E-38	1.071	5.96E-41	0.977	5.72E-27
GOBP_CELLULAR_RESPONSE_TO_NITROGEN_COMPOUND	GO:1901699	1.054	1.65E-10	1.068	1.48E-09	1.016	5.98E-15	1.098	9.47E-22	0.898	3.80E-20	0.978	2.67E-20	0.907	1.38E-24	0.825	2.65E-19	0.982	1.87E-29	1.000	6.96E-24	0.979	2.23E-24	0.825	1.62E-21	0.991	1.95E-18	0.937	2.10E-24
GOBP_PURINE_CONTAINING_COMPOUND_METABOLIC_PROCESS	GO:0072521	1.062	1.71E-10	1.008	5.05E-07	0.984	1.17E-12	1.201	1.21E-11	0.889	4.33E-11	0.951	3.66E-13	0.889	1.62E-13	0.997	1.02E-11	0.851	9.49E-15	0.806	8.86E-11	0.846	9.00E-13	0.960	4.60E-15	0.814	9.62E-14	0.996	7.93E-11
GOBP_REGULATION_OF_PROTEIN_LOCALIZATION	GO:1903829	1.139	1.93E-10	1.098	1.85E-11	0.924	4.38E-29	1.090	4.73E-19	0.780	8.82E-23	1.022	7.62E-19	0.824	1.72E-22	0.974	2.37E-19	1.016	4.47E-27	1.013	1.04E-28	1.013	3.77E-24	0.925	1.71E-28	0.941	4.85E-26	0.961	3.40E-23
GOCC_DENDRITIC_TREE	GO:0097447	0.898	1.95E-10	0.964	1.70E-08	0.858	8.79E-21	0.986	8.69E-25	0.834	1.80E-27	1.044	1.02E-26	0.871	6.54E-22	0.948	2.16E-19	1.101	4.20E-23	1.046	4.91E-23	1.103	3.41E-20	0.731	4.46E-16	0.898	9.92E-17	0.937	1.94E-20
GOCC_PRESYNAPSE	GO:0098793	0.900	2.13E-10	0.978	5.77E-07	0.982	3.60E-20	0.802	5.39E-18	1.084	7.69E-20	0.960	8.72E-24	0.843	6.57E-16	1.026	1.28E-16	0.994	1.13E-24	0.981	1.13E-26	0.990	4.36E-24	0.896	3.07E-25	0.956	9.65E-21	0.959	7.68E-25
GOBP_SYNAPSE_ORGANIZATION	GO:0050808	0.769	3.45E-10	0.841	2.72E-08	0.881	3.89E-18	0.735	2.63E-16	0.780	1.94E-16	0.960	2.37E-21	0.907	6.59E-20	0.872	2.75E-15	0.875	3.35E-16	0.784	8.56E-18	0.889	1.15E-19	0.598	2.70E-13	0.809	1.08E-15	0.772	1.50E-15
GOCC_MITOCHONDRIAL_ENVELOPE	GO:0005740	1.479	3.47E-10	1.571	4.07E-11	1.207	9.67E-14	1.281	6.30E-16	1.216	1.81E-16	1.226	7.89E-18	1.112	1.86E-17	1.265	5.51E-19	1.241	3.77E-26	1.066	8.47E-15	1.235	8.17E-27	1.185	6.18E-15	1.265	8.35E-16	0.964	3.65E-17
GOBP_PROCESS_UTILIZING_AUTOPHAGIC_MECHANISM	GO:0061919	1.130	3.54E-10	1.182	1.47E-08	1.174	6.64E-09	1.208	3.43E-14	0.940	1.31E-11	0.967	1.34E-13	0.871	3.00E-17	1.181	1.79E-15	1.085	9.01E-13	0.971	3.14E-15	1.071	2.56E-13	0.830	1.09E-11	1.026	2.21E-16	0.903	2.48E-08
GOBP_POSITIVE_REGULATION_OF_PROTEIN_PHOSPHORYLATION	GO:0001934	1.125	3.72E-10	1.226	2.44E-07	0.985	1.49E-12	0.909	4.82E-17	0.811	6.52E-18	1.014	4.56E-26	1.117	2.36E-22	0.943	9.40E-28	1.077	3.71E-29	1.022	7.02E-25	1.039	8.97E-34	0.904	8.58E-22	1.192	3.44E-24	1.052	5.61E-27
GOBP_CHROMATIN_ORGANIZATION	GO:0006325	1.050	4.01E-10	1.167	1.51E-07	0.979	5.57E-13	1.085	5.08E-14	0.954	1.82E-14	0.966	2.14E-18	0.982	3.60E-17	1.206	4.90E-17	0.998	1.35E-21	1.102	7.63E-10	0.989	7.00E-17	0.892	1.55E-20	1.029	9.77E-16	1.010	1.83E-15
GOBP_CELLULAR_RESPONSE_TO_HORMONE_STIMULUS	GO:0032870	1.035	4.56E-10	1.026	7.94E-11	1.018	7.61E-19	1.004	2.87E-23	0.728	7.77E-18	1.075	3.73E-27	0.837	8.28E-19	0.828	6.40E-17	0.917	2.12E-20	0.949	7.74E-25	0.922	4.56E-22	0.826	3.23E-23	0.906	1.18E-16	0.973	7.63E-22
GOCC_CELL_CELL_JUNCTION	GO:0005911	0.875	4.70E-10	0.859	1.64E-08	0.967	7.56E-15	0.818	5.65E-19	0.706	1.80E-16	0.765	1.33E-17	0.795	1.62E-14	0.739	2.62E-17	0.850	7.64E-19	0.899	9.49E-24	0.867	1.98E-20	0.755	1.13E-15	0.755	1.23E-18	0.801	1.77E-20
GOBP_NEGATIVE_REGULATION_OF_MULTICELLULAR_ORGANISMAL_PROCESS	GO:0051241	1.150	4.88E-10	1.275	8.11E-10	1.126	5.42E-21	1.077	9.44E-24	0.979	4.25E-30	1.018	1.37E-26	1.242	2.52E-24	0.959	7.99E-34	1.093	1.40E-34	0.887	7.09E-22	1.101	2.39E-42	1.044	3.02E-27	1.115	2.33E-34	1.183	7.42E-37
GOBP_ENDOMEMBRANE_SYSTEM_ORGANIZATION	GO:0010256	1.011	5.70E-10	1.038	7.18E-08	1.014	1.33E-15	1.042	8.26E-19	0.992	1.95E-13	0.889	2.09E-11	0.944	3.72E-17	1.106	7.34E-13	1.143	5.91E-14	1.044	5.20E-14	1.134	2.81E-16	1.028	1.06E-15	1.042	5.71E-16	0.956	2.51E-21
GOBP_POSITIVE_REGULATION_OF_INTRACELLULAR_SIGNAL_TRANSDUCTION	GO:1902533	1.088	6.77E-10	1.127	1.58E-11	1.123	8.37E-19	1.091	6.65E-22	0.988	7.34E-23	0.961	1.83E-26	1.058	3.04E-29	1.025	1.13E-29	1.126	6.03E-44	1.027	2.60E-45	1.087	9.54E-49	1.036	3.56E-32	1.205	3.04E-32	1.052	5.11E-41
GOBP_NEGATIVE_REGULATION_OF_DEVELOPMENTAL_PROCESS	GO:0051093	1.173	6.96E-10	1.191	1.06E-09	0.979	4.80E-23	0.941	8.97E-29	0.993	8.79E-29	1.014	1.35E-26	1.186	1.66E-29	0.923	6.88E-31	1.046	7.04E-29	0.884	2.04E-29	1.073	1.56E-38	0.989	9.40E-27	0.904	9.33E-30	1.104	5.11E-29
GOBP_G_PROTEIN_COUPLED_RECEPTOR_SIGNALING_PATHWAY	GO:0007186	0.999	7.24E-10	1.011	9.35E-13	1.261	2.22E-14	1.204	1.73E-23	1.185	4.86E-21	1.213	4.92E-36	1.070	5.97E-28	1.215	5.06E-22	1.263	3.66E-34	1.217	1.11E-25	1.126	1.09E-30	1.168	1.89E-33	1.241	5.68E-31	0.992	4.43E-32
GOCC_POLYMERIC_CYTOSKELETAL_FIBER	GO:0099513	1.223	7.42E-10	1.231	3.88E-08	0.907	1.51E-15	0.993	6.15E-20	1.143	2.42E-17	0.948	1.59E-17	0.981	2.35E-26	1.038	4.89E-20	1.083	3.79E-20	1.054	6.52E-18	1.097	3.88E-20	1.051	1.27E-21	1.159	2.18E-19	0.983	9.58E-17
GOMF_NUCLEOSIDE_TRIPHOSPHATASE_REGULATOR_ACTIVITY	GO:0060589	0.834	7.90E-10	0.825	6.30E-09	0.608	8.67E-11	0.896	1.00E-16	0.729	1.74E-17	0.863	3.73E-16	0.838	9.75E-20	0.889	5.77E-20	0.920	1.74E-25	0.817	2.27E-21	0.948	3.66E-28	0.798	8.67E-22	0.980	4.79E-18	0.829	2.71E-10
GOBP_REGULATION_OF_TRANSMEMBRANE_TRANSPORT	GO:0034762	0.988	7.92E-10	1.017	4.54E-08	1.013	1.26E-18	0.891	1.36E-17	1.187	6.30E-27	0.992	5.25E-22	0.934	1.74E-18	0.884	1.71E-19	0.934	5.91E-26	0.916	4.71E-21	0.924	1.50E-20	0.866	5.97E-19	0.886	1.09E-19	0.894	9.47E-21
GOBP_RESPONSE_TO_PEPTIDE	GO:1901652	1.040	8.72E-10	1.017	5.04E-10	1.024	5.71E-12	1.147	1.55E-16	0.986	1.63E-14	1.183	2.17E-19	1.051	8.88E-18	0.989	3.89E-18	1.162	1.64E-18	1.037	1.08E-12	1.143	8.75E-16	0.894	1.30E-15	0.883	1.36E-14	1.029	6.33E-15
GOBP_PEPTIDE_BIOSYNTHETIC_PROCESS	GO:0043043	1.610	9.39E-10	1.701	7.06E-08	0.972	3.68E-20	1.133	1.24E-16	1.099	7.51E-18	1.144	7.00E-15	1.163	1.32E-19	0.964	5.04E-15	0.996	5.62E-20	1.184	2.54E-19	1.012	1.98E-19	1.316	2.87E-18	1.086	9.81E-19	1.337	4.75E-15
GOCC_PERINUCLEAR_REGION_OF_CYTOPLASM	GO:0048471	1.111	1.06E-09	1.182	2.54E-09	0.848	4.35E-16	0.906	3.01E-19	0.875	8.56E-21	0.927	1.18E-21	0.920	9.64E-18	0.957	1.52E-24	1.019	1.38E-27	0.970	4.68E-20	1.009	1.26E-22	0.893	4.24E-23	0.919	1.23E-21	1.022	3.93E-22
GOMF_CIS_REGULATORY_REGION_SEQUENCE_SPECIFIC_DNA_BINDING	GO:0000987	1.246	1.18E-09	1.135	1.56E-10	1.085	3.66E-35	1.092	1.78E-25	1.016	1.37E-25	1.030	1.31E-27	1.061	1.83E-29	0.963	2.05E-33	0.893	1.53E-40	1.070	5.32E-41	0.911	4.01E-41	1.070	4.59E-37	0.911	6.26E-36	1.019	1.01E-35
GOBP_CELL_CYCLE	GO:0007049	1.394	1.24E-09	1.426	1.10E-11	0.978	2.92E-58	1.140	2.40E-35	0.944	1.99E-36	1.136	1.81E-34	1.033	7.40E-39	1.058	4.41E-34	1.061	5.63E-40	1.148	9.08E-51	1.094	1.31E-45	1.110	2.37E-53	1.042	7.04E-35	1.072	4.85E-50
GOBP_NEGATIVE_REGULATION_OF_CELL_DEATH	GO:0043069	1.283	1.24E-09	1.315	3.94E-09	1.255	5.35E-25	1.087	9.37E-24	0.982	1.69E-28	1.166	8.73E-31	1.181	9.36E-25	0.908	1.26E-25	1.013	1.86E-27	0.968	1.28E-29	0.996	3.63E-32	1.004	1.02E-27	1.028	5.50E-27	1.073	6.78E-36
GOBP_MICROTUBULE_CYTOSKELETON_ORGANIZATION	GO:0000226	1.337	1.37E-09	1.451	1.45E-08	0.851	2.29E-19	1.053	3.34E-25	1.081	1.47E-19	1.004	8.02E-16	1.018	4.52E-22	1.331	1.57E-23	1.260	5.74E-20	1.162	1.74E-20	1.281	4.09E-26	1.005	4.45E-22	1.024	3.35E-15	0.964	6.75E-18
GOBP_REGULATION_OF_IMMUNE_RESPONSE	GO:0050776	1.130	1.45E-09	1.294	7.13E-09	0.922	5.57E-11	0.999	2.62E-15	1.193	1.18E-17	1.002	1.97E-21	0.948	1.12E-16	1.080	1.08E-32	1.042	1.53E-24	0.997	9.11E-23	1.031	2.87E-24	0.965	8.36E-24	1.012	1.21E-23	0.931	2.96E-21
GOBP_PEPTIDE_METABOLIC_PROCESS	GO:0006518	1.505	1.59E-09	1.543	1.05E-08	0.940	5.06E-22	1.229	7.29E-22	1.073	1.66E-17	1.109	1.14E-18	1.053	1.25E-25	0.878	8.40E-19	0.914	2.85E-22	1.156	7.88E-24	0.932	2.33E-26	1.202	3.11E-22	1.082	1.44E-20	1.175	2.22E-18
GOBP_REGULATION_OF_ANATOMICAL_STRUCTURE_SIZE	GO:0090066	0.910	1.60E-09	0.988	2.35E-07	0.847	3.97E-16	0.838	1.04E-14	0.948	4.09E-18	0.933	6.56E-20	0.864	7.60E-18	0.884	3.64E-16	0.881	4.87E-17	0.871	5.27E-18	0.876	3.73E-15	0.805	3.11E-19	0.877	2.61E-18	0.915	1.29E-17
GOMF_DNA_BINDING_TRANSCRIPTION_FACTOR_ACTIVITY	GO:0003700	1.170	1.75E-09	1.059	1.70E-11	1.269	2.11E-19	1.116	5.39E-31	1.055	9.81E-34	1.116	3.93E-29	1.121	4.63E-37	1.005	8.41E-37	0.950	2.31E-45	1.090	1.97E-47	0.966	5.71E-46	1.059	3.09E-39	0.926	4.85E-27	1.028	9.16E-37
GOBP_REGULATION_OF_CELL_ADHESION	GO:0030155	0.993	1.76E-09	0.989	7.80E-10	0.869	2.91E-17	0.867	3.94E-28	0.828	6.37E-23	0.886	1.61E-22	0.878	1.58E-19	0.806	1.79E-30	0.872	4.42E-28	0.903	2.55E-33	0.865	5.50E-37	0.917	1.96E-28	0.723	1.18E-23	0.929	1.17E-38
GOBP_REGULATION_OF_RESPONSE_TO_EXTERNAL_STIMULUS	GO:0032101	1.126	1.84E-09	1.150	5.12E-10	0.903	2.98E-14	1.242	4.71E-27	1.011	8.14E-24	1.070	5.75E-33	1.173	4.20E-24	0.975	6.15E-22	1.046	4.91E-33	0.987	1.26E-28	1.047	9.99E-31	1.068	8.02E-25	0.926	8.98E-27	1.070	7.88E-36
GOMF_HYDROLASE_ACTIVITY_ACTING_ON_ACID_ANHYDRIDES	GO:0016817	1.467	1.85E-09	1.590	1.16E-11	0.863	6.71E-19	1.318	1.01E-14	0.991	2.18E-17	1.065	9.07E-18	1.065	1.52E-20	1.092	1.11E-18	1.126	5.85E-18	1.043	1.26E-17	1.139	2.50E-21	1.032	3.15E-24	1.343	1.62E-22	0.974	1.12E-17
GOBP_REGULATION_OF_CYTOSKELETON_ORGANIZATION	GO:0051493	0.886	1.99E-09	0.872	1.75E-08	0.802	3.08E-19	1.005	2.85E-13	0.930	5.82E-17	1.039	9.32E-19	0.951	6.05E-21	0.885	3.01E-13	0.939	4.87E-17	0.865	3.54E-20	1.054	2.47E-19	0.807	3.88E-22	0.935	7.64E-18	0.837	1.39E-16
GOBP_CELLULAR_LIPID_METABOLIC_PROCESS	GO:0044255	1.218	2.54E-09	1.219	3.51E-12	0.988	4.60E-24	1.346	1.63E-26	1.116	4.39E-27	1.198	4.35E-26	1.290	1.39E-37	1.073	1.91E-26	1.093	4.21E-32	1.012	1.69E-34	1.097	4.08E-35	0.977	7.56E-23	1.120	9.48E-31	1.029	3.30E-28
GOBP_CELL_PROJECTION_ASSEMBLY	GO:0030031	0.940	2.56E-09	1.004	8.02E-09	0.792	7.14E-15	0.849	1.24E-19	0.866	8.59E-17	0.898	8.27E-18	0.892	2.15E-20	1.111	1.87E-25	1.001	2.33E-25	1.042	4.45E-20	0.979	8.81E-27	0.877	5.54E-17	0.804	9.78E-20	1.077	9.39E-14
GOMF_LIPID_BINDING	GO:0008289	1.167	2.80E-09	1.156	4.07E-10	0.866	4.07E-20	0.911	1.19E-21	0.889	3.15E-25	0.926	1.16E-23	0.874	4.01E-21	1.059	1.19E-23	0.982	5.75E-28	1.165	2.97E-29	0.989	8.79E-21	0.907	1.18E-26	0.977	7.06E-26	0.904	9.64E-19
GOBP_MEMBRANE_ORGANIZATION	GO:0061024	1.096	2.83E-09	1.079	2.72E-09	0.983	2.79E-20	1.017	7.00E-22	0.994	5.84E-17	0.885	3.59E-19	0.728	2.44E-20	1.052	1.50E-24	1.070	1.61E-26	1.061	9.76E-26	1.062	1.11E-32	0.925	7.38E-31	0.982	5.20E-29	0.851	8.06E-25
GOBP_BEHAVIOR	GO:0007610	0.996	3.03E-09	1.002	4.62E-08	0.913	5.16E-16	0.842	6.19E-20	0.887	1.54E-23	0.965	5.55E-26	0.928	2.82E-19	0.965	1.01E-20	0.985	1.95E-20	0.968	7.97E-24	1.015	1.58E-27	0.783	4.60E-25	0.899	9.20E-21	0.847	3.06E-18
GOCC_ENDOSOME	GO:0005768	1.080	3.09E-09	1.114	1.12E-09	1.041	6.25E-23	1.103	1.53E-20	1.182	2.06E-28	0.895	4.53E-27	0.820	1.20E-32	1.001	1.01E-33	1.108	5.96E-31	0.962	6.42E-35	1.122	4.67E-35	0.936	4.40E-27	0.920	1.00E-26	0.946	6.29E-21
GOBP_NEGATIVE_REGULATION_OF_NUCLEOBASE_CONTAINING_COMPOUND_METABOLIC_PROCESS	GO:0045934	1.145	3.35E-09	1.095	1.67E-11	1.191	5.46E-21	1.093	2.99E-39	0.982	7.07E-34	0.978	9.13E-34	1.114	6.09E-36	1.069	1.22E-52	0.951	1.44E-49	1.001	3.20E-40	0.953	2.57E-50	1.015	4.61E-35	0.944	2.86E-39	1.040	2.09E-43
GOBP_CELL_CYCLE_PROCESS	GO:0022402	1.335	3.63E-09	1.394	2.04E-10	0.898	1.57E-38	1.107	1.35E-27	0.961	3.56E-26	1.106	1.13E-25	1.004	8.90E-28	1.070	4.81E-33	1.048	2.50E-31	1.126	3.99E-35	1.087	2.31E-36	1.136	6.46E-38	1.122	2.89E-27	1.049	2.41E-30
GOBP_MULTI_ORGANISM_REPRODUCTIVE_PROCESS	GO:0044703	1.330	3.71E-09	1.455	7.81E-10	1.143	8.67E-19	1.101	6.36E-27	0.977	3.42E-22	0.976	1.25E-24	1.160	4.08E-25	1.125	4.95E-23	1.016	1.26E-25	1.016	1.26E-25	1.355	1.18E-28	1.234	1.45E-28	1.160	1.37E-26	1.222	1.45E-28
GOBP_ESTABLISHMENT_OF_ORGANELLE_LOCALIZATION	GO:0051656	1.100	3.99E-09	1.172	6.30E-07	1.279	1.88E-07	0.972	7.74E-12	0.947	1.16E-12	0.886	5.16E-13	0.818	5.52E-14	0.953	6.12E-14	1.017	6.36E-14	0.999	6.26E-12	1.043	9.01E-11	1.166	4.76E-15	1.226	3.61E-14	1.121	4.21E-11
GOBP_DEVELOPMENTAL_PROCESS_INVOLVED_IN_REPRODUCTION	GO:0003006	1.289	4.22E-09	1.384	4.70E-09	1.129	4.87E-18	1.092	4.28E-33	0.891	9.51E-25	0.977	7.48E-26	1.250	1.26E-26	1.102	2.59E-23	1.159	3.72E-30	1.033	3.73E-24	1.172	1.69E-29	1.146	9.48E-30	0.951	1.83E-26	1.173	8.51E-28
GOBP_PROTEIN_CATABOLIC_PROCESS	GO:0030163	1.340	4.60E-09	1.401	3.20E-10	0.818	1.97E-24	1.256	3.53E-23	0.971	2.86E-26	0.892	2.03E-21	1.099	1.30E-27	1.031	2.79E-23	0.999	9.64E-28	1.021	5.12E-28	1.021	4.49E-30	0.970	1.98E-24	1.020	2.94E-23	0.947	2.39E-19
GOBP_MODIFICATION_DEPENDENT_MACROMOLECULE_CATABOLIC_PROCESS	GO:0043632	1.338	4.65E-09	1.351	3.06E-08	0.877	1.73E-18	1.308	4.07E-16	0.960	6.54E-18	0.893	3.04E-14	0.916	5.20E-15	1.013	1.23E-15	1.033	1.00E-21	0.955	5.00E-19	1.038	7.31E-22	1.028	2.52E-17	1.131	1.86E-17	0.930	1.08E-13
GOBP_SENSORY_PERCEPTION	GO:0007600	1.129	5.11E-09	1.092	1.40E-07	0.934	2.56E-14	1.080	8.11E-22	1.202	7.32E-23	1.287	2.28E-23	1.216	1.51E-20	1.126	2.81E-23	1.108	4.57E-22	1.132	2.98E-23	1.103	3.74E-25	1.020	2.83E-22	1.103	1.05E-21	0.997	2.50E-15
GOBP_MUSCLE_STRUCTURE_DEVELOPMENT	GO:0061061	1.046	5.47E-09	0.976	1.45E-10	0.916	4.67E-18	0.736	1.19E-15	0.680	8.00E-17	0.900	1.43E-22	0.913	6.16E-18	0.672	7.33E-21	0.804	8.68E-21	0.800	1.78E-19	0.799	1.06E-22	0.830	2.75E-23	0.844	1.89E-22	0.847	1.48E-28
GOMF_CYTOSKELETAL_PROTEIN_BINDING	GO:0008092	1.087	5.58E-09	1.096	4.26E-09	0.850	5.46E-29	0.894	1.26E-33	0.885	3.06E-30	0.812	9.50E-28	0.855	8.25E-47	0.897	6.85E-35	0.894	8.44E-29	0.883	1.09E-49	0.891	4.88E-33	0.905	3.57E-33	0.916	1.45E-32	0.883	6.56E-32
GOBP_CELL_GROWTH	GO:0016049	0.965	5.61E-09	0.919	1.51E-08	1.156	1.39E-11	0.941	1.45E-15	0.854	4.26E-18	0.894	7.39E-17	1.077	2.96E-15	0.818	1.63E-14	0.969	1.89E-17	0.937	1.12E-16	0.957	2.55E-17	0.922	2.92E-15	0.712	6.04E-15	0.895	1.28E-18
GOBP_MONOCARBOXYLIC_ACID_METABOLIC_PROCESS	GO:0032787	1.053	6.14E-09	1.054	3.94E-08	0.804	1.19E-16	1.286	8.53E-16	1.174	3.33E-17	1.225	3.07E-19	1.136	9.29E-19	1.015	9.53E-15	0.979	4.08E-21	1.047	1.38E-17	0.965	2.26E-16	1.034	7.97E-14	1.053	4.57E-17	1.086	4.05E-19
GOCC_MICROTUBULE_CYTOSKELETON	GO:0015630	1.268	6.31E-09	1.323	7.23E-10	0.912	7.35E-33	0.942	2.43E-30	0.970	3.02E-37	0.946	4.20E-30	0.948	3.28E-40	1.061	7.20E-35	1.073	1.95E-37	1.088	1.85E-35	1.077	1.66E-37	1.049	9.56E-40	1.037	5.31E-33	1.047	3.67E-47
GOMF_CELL_ADHESION_MOLECULE_BINDING	GO:0050839	0.882	6.35E-09	0.906	9.51E-08	0.943	3.44E-13	0.701	1.61E-16	0.775	1.62E-21	0.707	1.66E-17	0.737	2.38E-16	0.830	1.75E-23	0.828	3.99E-35	0.756	5.86E-27	0.843	3.87E-23	0.713	1.39E-26	0.734	2.19E-22	0.827	1.58E-27
GOBP_RESPONSE_TO_EXTRACELLULAR_STIMULUS	GO:0009991	1.104	7.06E-09	1.184	8.38E-09	0.909	1.86E-15	1.236	9.25E-18	0.917	1.54E-14	1.115	2.18E-18	1.096	3.21E-19	1.110	3.10E-16	1.245	1.00E-17	0.938	1.68E-14	1.197	8.40E-17	0.995	3.75E-16	1.069	9.93E-18	1.166	1.41E-12
GOBP_REGULATION_OF_ION_TRANSMEMBRANE_TRANSPORT	GO:0034765	1.021	7.15E-09	1.072	3.66E-07	1.108	4.49E-16	0.877	2.08E-14	1.187	1.46E-22	1.010	1.80E-19	0.914	1.48E-16	0.946	5.13E-18	0.933	8.20E-20	0.977	1.62E-17	0.929	1.26E-17	0.814	2.85E-16	0.906	2.48E-16	0.872	1.41E-14
GOBP_RESPONSE_TO_INORGANIC_SUBSTANCE	GO:0010035	1.137	8.33E-09	1.143	4.86E-09	0.957	2.12E-15	1.102	4.20E-15	0.867	3.84E-14	1.145	4.27E-16	1.025	2.53E-23	0.778	1.60E-11	0.943	2.98E-14	0.826	2.78E-14	0.934	8.78E-16	1.007	3.98E-14	0.990	2.79E-18	1.142	4.17E-15
GOCC_CENTROSOME	GO:0005813	1.115	8.69E-09	1.187	4.50E-07	0.895	9.96E-20	0.948	1.26E-19	0.941	4.19E-16	1.043	9.97E-15	1.008	1.84E-18	0.988	3.45E-17	1.008	1.44E-16	1.045	9.06E-15	1.020	2.88E-21	1.100	2.49E-21	0.976	2.26E-14	1.067	9.45E-25
GOMF_CATION_TRANSMEMBRANE_TRANSPORTER_ACTIVITY	GO:0022857	1.128	8.70E-09	1.164	4.24E-08	1.058	5.99E-24	1.045	6.97E-20	1.311	9.55E-24	1.369	9.80E-30	0.992	3.81E-21	1.082	2.15E-20	0.925	8.53E-27	1.008	1.82E-23	1.003	2.26E-21	0.901	4.14E-20	0.934	1.06E-17	0.922	3.37E-17
GOBP_REGULATION_OF_PROTEOLYSIS	GO:0030162	1.191	8.84E-09	1.267	1.45E-07	0.952	3.36E-17	1.150	3.33E-16	0.849	4.86E-17	0.915	3.06E-25	0.997	1.04E-21	1.104	5.82E-24	1.111	2.55E-20	1.024	8.98E-25	1.117	1.53E-24	0.839	7.19E-20	0.981	2.72E-23	0.777	2.49E-23
GOMF_HYDROLASE_ACTIVITY_ACTING_ON_ESTER_BONDS	GO:0016788	1.274	8.86E-09	1.314	2.63E-08	0.978	7.82E-15	1.059	4.07E-22	1.161	4.04E-20	1.003	7.36E-23	1.208	1.52E-27	1.136	1.01E-21	1.134	1.16E-24	0.873	7.90E-22	1.125	1.88E-28	1.038	8.66E-21	1.102	7.93E-32	1.164	2.39E-26
GOBP_REGULATION_OF_ACTIN_FILAMENT_BASED_PROCESS	GO:0032970	0.871	1.11E-08	0.864	3.26E-07	0.681	9.39E-16	0.990	7.01E-11	0.746	1.41E-15	0.831	4.29E-12	0.661	3.88E-11	0.856	1.10E-12	0.741	3.69E-12	0.788	3.69E-17	0.870	5.40E-17	0.710	2.61E-15	0.792	5.44E-12	0.808	4.71E-16
GOBP_MULTICELLULAR_ORGANISM_REPRODUCTION	GO:0022412	1.527	1.52E-08	1.486	2.87E-08	1.178	2.57E-16	1.132	1.85E-26	0.918	1.54E-21	0.940	1.51E-18	1.117	9.56E-25	1.086	2.59E-21	1.110	2.14E-23	0.941	6.26E-15	1.140	3.16E-23	1.202	1.27E-25	0.972	1.81E-23	1.208	4.12E-25
GOBP_MITOCHONDRION_ORGANIZATION	GO:0007005	1.494	1.74E-08	1.482	5.13E-08	1.253	2.70E-12	1.039	2.01E-11	1.141	8.93E-10	1.117	2.06E-12	1.061	1.49E-18	1.221	7.91E-13	1.194	3.74E-12	1.062	1.17E-13	1.176	1.69E-12	0.994	2.05E-11	1.286	3.74E-12	1.003	5.79E-11
GOBP_ENDOCYTOSIS	GO:0006897	0.999	1.80E-08	1.002	4.74E-10	0.972	4.14E-16	0.988	3.86E-19	0.946	4.85E-19	0.806	1.16E-19	0.843	2.10E-18	1.015	1.70E-20	1.019	6.15E-21	1.019	3.14E-25	1.016	9.38E-29	1.018	4.78E-19	1.016	1.39E-18	1.086	4.35E-14
GOCC_MICROTUBULE_ORGANIZING_CENTER	GO:0005815	1.224	1.89E-08	1.305	2.18E-09	0.826	1.03E-23	0.929	1.35E-22	0.937	8.86E-22	1.013	8.83E-18	0.966	1.72E-21	0.960	7.06E-25	1.080	2.82E-34	1.173	4.15E-24	1.074	3.34E-31	1.052	3.66E-23	1.021	2.55E-19	1.031	3.36E-27
GOCC_NUCLEOLUS	GO:0005730	1.383	2.05E-08	1.362	7.72E-12	0.960	3.65E-28	1.218	8.77E-26	0.755	1.84E-22	1.084	5.74E-23	1.135	2.44E-26	0.955	7.97E-28	0.954	7.26E-31	0.967	5.47E-32	0.966	1.75E-28	0.897	5.25E-25	1.116	2.54E-25	0.869	4.29E-26
GOCC_PLASMA_MEMBRANE_PROTEIN_COMPLEX	GO:0098797	1.070	2.31E-08	1.065	1.40E-09	1.024	2.60E-23	0.827	1.19E-18	1.065	4.61E-16	0.906	8.26E-25	0.902	2.72E-24	0.947	2.76E-22	0.858	2.56E-27	1.012	1.23E-26	0.844	1.26E-18	0.812	4.60E-18	0.857	1.89E-17	0.902	5.03E-23
GOBP_POSITIVE_REGULATION_OF_LOCOMOTION	GO:0040017	1.075	2.49E-08	0.987	2.10E-08	0.802	1.45E-14	1.082	5.48E-19	0.910	4.01E-20	0.990	2.55E-22	1.056	2.34E-17	0.895	5.54E-19	1.010	3.64E-22	0.959	2.91E-27	0.987	1.54E-24	0.951	3.74E-30	0.970	6.33E-20	1.031	5.58E-21
GOBP_RESPONSE_TO_CYTOKINE	GO:0034097	1.181	2.82E-08	1.195	7.63E-09	1.360	3.06E-18	1.229	1.50E-25	0.978	6.61E-21	0.982	2.55E-24	1.141	3.57E-24	1.179	8.86E-25	1.129	3.46E-25	1.034	3.33E-24	1.117	1.16E-26	1.109	3.04E-29	1.020	2.30E-20	1.093	5.70E-32

GO Name	GO ID	Infection		Hospitalization		Severe Symptoms		IDP2834		IDP2831		IDP2195		IDP1985		IDP1274		IDP1160		IDP1115		IDP1069		IDP0957		IDP0910		IDP0766	
		enrich	p	enrich	p	enrich	p	enrich	p	enrich	p	enrich	p	enrich	p	enrich	p	enrich	p	enrich	p	enrich	p	enrich	p	enrich	P	enrich	p
GOCC_CILIUM	GO:0005929	1.263	3.09E-08	1.307	3.40E-07	0.892	1.77E-11	0.999	8.43E-20	1.194	3.31E-24	1.036	4.24E-17	1.156	1.60E-22	1.309	2.69E-25	1.246	2.38E-30	1.174	2.86E-17	1.257	3.16E-24	1.073	6.25E-22	1.048	4.16E-19	1.032	1.96E-14
GOBP_ION_HOMEOSTASIS	GO:0006879	1.109	3.13E-08	1.145	1.05E-07	1.117	6.55E-17	1.051	1.64E-21	1.058	7.19E-24	1.086	3.94E-24	0.967	1.06E-20	0.944	1.16E-20	1.047	2.41E-26	1.003	1.38E-26	1.069	5.98E-27	0.810	5.26E-27	1.000	5.92E-28	0.793	3.01E-23
GOBP_RESPONSE_TO_RADIATION	GO:0009314	1.155	3.17E-08	1.248	1.48E-07	0.900	2.04E-09	0.886	2.68E-12	0.920	7.57E-13	1.032	6.58E-11	1.149	2.00E-13	1.089	1.25E-14	0.833	7.88E-10	0.941	7.36E-19	0.854	2.14E-13	0.923	1.80E-15	1.169	2.29E-13	1.078	2.86E-13
GOCC_INTRACELLULAR_PROTEIN_CONTAINING_COMPLEX	GO:0140535	1.432	3.32E-08	1.487	9.21E-10	0.810	5.09E-15	1.203	2.01E-20	0.943	1.97E-16	0.966	4.71E-17	1.029	1.58E-28	0.909	9.57E-16	1.033	1.12E-20	1.116	1.26E-13	1.030	1.62E-24	0.899	5.92E-20	1.046	6.16E-24	0.946	1.77E-22
GOBP_LOCALIZATION_WITHIN_MEMBRANE	GO:0051668	1.125	3.81E-08	1.104	5.62E-07	0.892	2.85E-20	0.863	5.17E-17	0.910	1.40E-20	0.825	3.32E-22	0.825	1.02E-20	0.960	2.63E-24	0.984	1.75E-27	0.941	5.14E-19	1.001	9.74E-23	0.833	4.93E-19	0.920	1.10E-21	0.990	1.40E-18
GOBP_CIRCULATORY_SYSTEM_PROCESS	GO:0003013	1.037	4.01E-08	1.138	1.79E-07	0.984	2.66E-23	1.100	1.64E-18	0.910	1.77E-23	0.974	4.23E-21	0.981	3.48E-17	0.891	1.22E-18	0.958	4.55E-21	0.973	1.61E-25	0.949	4.52E-26	1.009	8.70E-23	1.061	2.15E-26	0.796	1.17E-21
GOBP_REGULATION_OF_NEURON_PROJECTION_DEVELOPMENT	GO:0010975	0.923	4.77E-08	0.998	1.29E-07	1.103	2.56E-14	0.730	1.18E-12	0.879	3.14E-19	0.976	3.46E-20	0.930	4.22E-15	0.771	4.57E-14	0.878	5.12E-15	0.761	1.29E-19	0.895	5.85E-19	0.859	1.14E-18	0.890	3.08E-19	0.862	9.39E-24
GOBP_CELLULAR_RESPONSE_TO_ABIOTIC_STIMULUS	GO:0071214	1.172	5.28E-08	1.214	1.24E-07	0.795	2.25E-08	1.241	1.79E-09	1.110	3.26E-10	1.372	4.24E-10	1.119	1.41E-10	0.924	7.32E-11	0.978	8.09E-13	1.192	4.57E-13	0.997	2.53E-12	1.235	7.70E-11	1.068	2.62E-11	1.106	6.13E-10
GOBP_EPITHELIAL_CELL_DIFFERENTIATION	GO:0030855	1.117	6.96E-08	1.124	1.10E-08	0.942	4.44E-20	0.986	7.15E-16	0.934	4.61E-22	0.916	1.43E-19	1.242	8.35E-23	0.988	9.53E-24	1.104	2.15E-21	0.891	6.85E-20	1.080	8.45E-28	1.020	1.42E-17	0.971	1.18E-16	1.233	1.85E-23
GOCC_TRANSFERASE_COMPLEX	GO:1990234	1.458	7.41E-08	1.478	3.00E-11	0.882	1.74E-15	1.089	2.29E-16	0.910	2.48E-17	0.932	1.12E-15	1.068	5.25E-21	1.031	3.78E-17	1.009	5.47E-20	1.167	1.18E-12	1.017	2.07E-24	0.874	4.20E-19	1.131	3.09E-22	1.018	4.67E-20
GOCC_GOLGI_MEMBRANE	GO:0000139	1.172	7.96E-08	1.124	2.27E-07	0.847	2.55E-18	1.258	5.51E-21	0.949	1.91E-15	0.970	7.02E-22	0.945	8.49E-19	0.941	5.15E-24	0.973	5.76E-23	0.823	1.10E-15	0.989	4.04E-21	0.987	9.23E-20	1.058	7.54E-22	0.921	2.52E-16
GOBP_MULTICELLULAR_ORGANISMAL_HOMEOSTASIS	GO:0048871	1.128	9.43E-08	1.066	1.06E-08	1.042	1.45E-15	1.056	5.33E-15	1.099	2.51E-22	1.059	1.68E-15	1.162	7.25E-19	1.103	4.15E-27	0.894	3.25E-18	1.046	1.34E-19	0.888	7.13E-21	0.936	4.24E-16	0.896	1.10E-18	0.983	1.18E-18
GOBP_LIPID_BIOSYNTHETIC_PROCESS	GO:0008610	1.177	1.15E-07	1.196	5.80E-09	0.916	1.38E-18	1.279	1.48E-17	1.083	2.39E-18	1.161	4.78E-21	1.123	1.63E-19	1.109	1.42E-21	1.012	1.56E-22	1.082	1.22E-28	1.008	4.65E-26	0.888	2.93E-17	1.065	4.61E-20	0.921	2.66E-31
GOBP_LYMPHOCYTE_ACTIVATION	GO:0046649	1.058	1.15E-07	1.203	4.38E-07	0.910	4.84E-20	1.025	3.93E-18	1.012	1.03E-18	0.987	4.23E-19	0.987	2.64E-19	0.973	3.74E-28	0.995	1.93E-24	0.931	7.00E-18	0.976	6.32E-25	0.851	1.36E-23	0.976	8.22E-24	0.812	8.34E-24
GOBP_SMALL_MOLECULE_BIOSYNTHETIC_PROCESS	GO:0044283	1.177	1.18E-07	1.088	2.64E-07	0.862	6.74E-17	1.400	8.13E-16	1.105	8.69E-17	1.033	2.35E-15	1.125	1.59E-15	0.935	2.48E-15	0.833	7.69E-14	0.997	7.10E-14	0.824	3.12E-13	0.989	3.47E-12	1.160	1.52E-15	1.008	6.68E-16
GOMF_PEPTIDASE_ACTIVITY	GO:0008233	1.157	1.47E-07	1.238	6.78E-08	1.404	1.22E-10	1.407	6.10E-16	1.160	8.77E-16	1.077	6.39E-14	1.088	6.32E-15	0.961	4.98E-20	0.979	6.89E-17	1.193	1.63E-20	0.969	5.56E-18	1.243	1.57E-18	1.244	5.16E-16	1.202	9.13E-18
GOBP_RESPONSE_TO_XENOBIOTIC_STIMULUS	GO:0009410	1.083	1.59E-07	1.063	3.68E-07	1.129	1.26E-07	1.263	4.82E-14	1.204	2.65E-12	1.186	1.57E-12	1.073	3.16E-11	0.916	9.20E-14	1.060	3.39E-10	0.999	9.55E-10	1.048	1.00E-14	1.204	1.16E-16	0.950	7.81E-13	1.222	6.62E-11
GOBP_TISSUE_MORPHOGENESIS	GO:0048729	1.037	1.69E-07	1.021	2.01E-10	1.056	4.93E-28	1.027	3.18E-20	0.784	1.88E-20	1.027	3.01E-23	1.184	6.42E-20	0.935	5.61E-26	1.196	1.79E-34	0.983	3.25E-21	1.199	3.87E-38	0.971	4.31E-23	1.050	6.76E-25	1.024	2.73E-22
GOBP_GENERATION_OF_PRECURSOR_METABOLITES_AND_ENERGY	GO:0006091	1.493	1.76E-07	1.516	6.96E-08	1.315	2.23E-12	1.237	4.10E-12	1.199	6.50E-17	1.028	1.98E-13	1.240	2.39E-13	1.121	3.44E-13	1.059	2.20E-18	1.114	6.52E-13	1.046	3.88E-16	0.991	1.99E-12	1.138	6.82E-12	1.039	3.10E-12
GOBP_REGULATION_OF_CELLULAR_COMPONENT_SIZE	GO:0032535	0.895	1.79E-07	0.901	6.51E-08	0.783	2.19E-12	0.803	2.32E-11	0.897	7.53E-14	0.976	6.94E-14	0.856	1.70E-13	0.847	3.33E-15	0.911	1.35E-12	0.859	1.07E-12	0.927	1.14E-11	0.753	7.25E-14	0.770	9.77E-14	0.883	5.46E-13
GOBP_RESPONSE_TO_GROWTH_FACTOR	GO:0070848	1.096	1.84E-07	1.116	6.02E-07	0.979	6.15E-19	1.154	3.08E-19	0.904	1.96E-18	0.982	4.12E-25	1.055	1.14E-24	0.931	5.01E-26	1.075	7.02E-24	1.011	9.25E-33	1.052	8.00E-29	1.045	1.96E-26	1.066	1.22E-23	1.040	9.18E-29
GOBP_CARBOHYDRATE_METABOLIC_PROCESS	GO:0005975	1.224	1.94E-07	1.194	4.70E-10	0.849	8.27E-14	1.052	8.13E-15	1.072	3.12E-17	1.042	3.02E-18	1.072	4.23E-16	1.077	5.17E-15	1.068	2.80E-27	1.068	1.30E-18	1.084	1.75E-19	1.109	3.24E-18	1.047	1.73E-18	1.160	1.42E-18
GOMF_STRUCTURAL_MOLECULE_ACTIVITY	GO:0005198	1.409	2.00E-07	1.436	1.26E-10	1.046	1.00E-11	0.959	1.30E-20	0.996	7.94E-21	0.993	7.87E-21	1.126	9.60E-24	1.023	3.24E-24	0.960	1.71E-18	0.981	1.19E-19	0.972	1.99E-23	1.000	6.90E-20	1.055	3.47E-21	0.976	1.04E-19
GOBP_REGULATION_OF_CELL_CYCLE	GO:0051726	1.349	2.02E-07	1.404	4.75E-08	1.079	4.34E-35	1.054	1.97E-23	0.892	5.07E-22	1.114	8.69E-22	1.025	3.16E-31	1.138	1.64E-31	1.049	1.30E-30	1.138	3.36E-28	1.088	1.69E-35	1.055	1.26E-28	1.049	4.04E-24	1.021	7.47E-27
GOBP_RESPONSE_TO_PEPTIDE_HORMONE	GO:0043434	1.036	2.20E-07	1.001	3.04E-08	0.975	2.80E-10	1.283	1.32E-15	1.009	1.30E-13	1.125	2.97E-14	1.052	1.60E-12	1.004	4.11E-13	1.219	3.06E-14	1.111	5.66E-13	1.198	2.21E-13	0.862	8.89E-14	0.887	6.10E-12	1.019	6.86E-15
GOBP_CELLULAR_RESPONSE_TO_PEPTIDE	GO:1901653	1.045	2.27E-07	1.037	2.00E-08	0.955	7.95E-08	1.199	1.25E-13	0.869	1.33E-10	1.096	7.31E-16	0.934	1.13E-12	0.880	3.01E-12	1.028	4.66E-13	0.987	1.57E-09	1.001	1.11E-12	0.818	4.60E-13	0.887	4.67E-11	0.951	1.83E-18
GOCC_RIBONUCLEOPROTEIN_COMPLEX	GO:1990904	1.601	2.31E-07	1.706	5.72E-09	0.930	4.57E-08	1.272	2.27E-16	1.186	1.96E-14	1.254	1.58E-18	1.185	1.58E-15	1.091	1.27E-14	1.086	1.89E-17	1.229	4.33E-14	1.090	4.09E-22	1.199	7.59E-17	1.223	1.42E-19	1.091	1.35E-15
GOBP_ACTIN_FILAMENT_BASED_PROCESS	GO:0030029	1.002	2.73E-07	0.998	7.63E-08	0.672	6.68E-23	0.902	1.97E-22	0.843	5.94E-28	0.756	5.35E-26	0.774	3.47E-29	0.746	1.07E-28	0.868	1.59E-26	0.750	8.45E-37	0.875	1.14E-28	0.787	1.84E-33	0.904	1.03E-25	0.863	3.10E-38
GOMF_CALCIUM_ION_BINDING	GO:0005509	1.007	2.84E-07	1.024	3.51E-08	1.196	2.83E-12	0.792	6.26E-20	1.021	1.41E-30	0.961	1.46E-20	1.108	8.07E-35	0.953	9.89E-23	0.992	6.04E-30	1.036	8.85E-26	0.987	1.20E-27	0.873	1.80E-23	0.962	2.38E-22	0.864	1.07E-22
GOBP_MITOTIC_CELL_CYCLE	GO:0000278	1.401	2.89E-07	1.463	4.15E-09	0.816	2.76E-25	1.034	2.78E-19	0.939	2.55E-25	1.162	1.26E-22	1.043	2.77E-30	1.205	7.07E-25	1.152	2.09E-24	1.322	1.74E-29	1.199	7.46E-31	1.173	1.49E-25	1.062	1.69E-19	1.145	8.66E-21
GOMF_PHOSPHORIC_ESTER_HYDROLASE_ACTIVITY	GO:0042578	0.957	3.03E-07	1.013	3.09E-09	0.993	2.46E-08	0.900	5.52E-12	0.949	3.06E-09	0.874	3.47E-13	1.204	5.09E-19	1.058	1.19E-11	1.032	8.53E-15	0.689	7.71E-13	1.014	1.97E-15	0.879	2.42E-11	0.836	5.09E-16	1.025	5.76E-12
GOBP_RESPONSE_TO_WOUNDING	GO:0009611	1.008	3.22E-07	1.123	2.18E-07	0.917	7.64E-16	1.018	3.69E-16	0.812	8.39E-18	1.112	1.41E-18	1.098	2.25E-20	0.943	3.37E-19	1.005	1.02E-22	0.931	6.33E-19	0.992	1.78E-19	0.984	2.19E-26	1.130	3.91E-20	1.055	3.40E-19
GOBP_NEGATIVE_REGULATION_OF_CELL_DIFFERENTIATION	GO:0045596	1.272	3.43E-07	1.275	1.99E-07	1.125	9.15E-15	0.964	4.59E-18	0.876	3.41E-20	1.058	2.02E-19	1.255	7.04E-24	0.940	1.99E-21	1.008	2.36E-26	0.889	4.66E-22	1.020	9.25E-31	0.944	4.29E-19	0.902	6.75E-26	1.178	5.96E-22
GOBP_NEGATIVE_REGULATION_OF_CELLULAR_COMPONENT_ORGANIZATION	GO:0051129	1.113	3.45E-07	1.119	2.95E-08	0.964	4.82E-18	0.921	1.96E-16	1.066	3.37E-24	0.892	1.60E-19	0.989	3.10E-25	0.874	8.02E-19	0.958	1.13E-23	0.949	2.45E-17	0.961	1.23E-22	0.870	6.24E-22	0.915	2.69E-18	0.830	1.42E-27
GOBP_DEPHOSPHORYLATION	GO:0006470	1.001	3.50E-07	1.022	3.69E-07	0.861	1.12E-09	0.875	5.15E-14	0.758	1.41E-08	0.841	6.81E-13	1.151	1.52E-19	1.012	4.52E-14	1.023	3.92E-16	0.793	4.62E-15	1.008	1.04E-15	0.957	3.37E-17	0.733	4.98E-14	1.017	1.14E-12
GOCC_CELL_BODY	GO:0044297	0.952	3.91E-07	0.954	3.60E-07	0.893	2.96E-15	0.935	7.64E-22	1.000	1.26E-21	1.087	3.41E-23	0.932	1.13E-20	0.877	1.03E-16	0.957	4.03E-24	0.990	3.37E-20	0.939	2.25E-17	0.788	4.21E-16	0.963	6.42E-17	0.883	8.43E-18
GOMF_MOLECULAR_ADAPTOR_ACTIVITY	GO:0060090	1.017	4.14E-07	1.083	3.36E-07	0.923	7.22E-12	0.969	4.13E-11	0.874	2.07E-11	0.889	2.54E-11	0.905	1.62E-11	0.828	6.75E-18	0.945	1.09E-11	0.772	5.42E-17	0.944	3.43E-14	0.756	5.25E-12	0.921	1.36E-16	0.663	2.40E-10
GOBP_DEVELOPMENTAL_MATURATION	GO:0021700	1.015	4.81E-07	0.984	5.28E-07	1.309	1.04E-07	1.259	7.09E-13	0.968	6.59E-14	0.838	5.12E-10	1.084	1.77E-11	1.024	1.80E-10	0.959	4.10E-11	1.054	2.99E-10	0.928	4.14E-13	0.813	2.69E-09	0.968	1.38E-13	0.846	1.35E-08
GOBP_RESPONSE_TO_OXIDATIVE_STRESS	GO:0006979	1.320	5.99E-07	1.320	2.38E-08	0.922	6.55E-08	1.121	1.13E-10	0.958	3.91E-11	1.118	1.43E-15	1.219	1.81E-14	0.935	1.50E-09	0.982	1.90E-10	0.931	2.31E-14	0.960	1.27E-13	1.189	1.20E-11	1.023	4.08E-12	0.924	7.21E-12
GOBP_LEUKOCYTE_DIFFERENTIATION	GO:0002521	1.162	6.04E-07	1.349	2.98E-09	0.973	1.68E-16	1.063	5.18E-19	1.054	1.14E-15	0.877	2.27E-15	0.880	4.36E-16	0.974	2.66E-19	0.970	4.16E-22	0.944	3.98E-20	0.929	2.03E-18	0.920	3.44E-17	1.027	1.73E-20	0.845	1.25E-17

Supplemental Table 8: Non-redundant gene ontology (GO) enrichments and their classification into represantative domains. GO terms with nominally significant difference (difference-p<0.05) in enrichment statistics among COVID-19 outcomes (severe respiratory symptoms, 13,769 cases and 1,072,442 controls; hospitalization 32,519 cases and 2,062,805 controls; infection, 122,616 cases and 2,475,240 controls) are reported in red.

		Representative Domain	Infection			Hospitalization			Severe Symptoms			Infection vs Severe Symptoms		Hospitalization vs. Severe Symptoms		Infection vs Hospitalization	
GO Name	GO ID		Enrich	SE	p	Enrich	SE	p	Enrich	SE	p	z	p	z	p	z	p
GOBP_BEHAVIOR	GO:0007610	cell activation	0.996	0.168	3.03E-09	1.002	0.183	4.62E-08	0.919	0.113	5.16E-16	0.376	0.354	0.384	0.350	-0.027	0.978
GOBP_MICROTUBULE_CYTOSKELETON_ORGANIZATION	GO:0000226		1.337	0.221	1.37E-09	1.451	0.256	1.45E-08	0.851	0.095	2.29E-19	2.026	0.021	2.200	0.014	-0.338	0.736
GOBP_CHROMATIN_ORGANIZATION	GO:0006325		1.050	0.168	4.01E-10	1.167	0.222	1.51E-07	0.979	0.136	5.57E-13	0.327	0.372	0.719	0.236	-0.419	0.675
GOBP_MITOCHONDRION_ORGANIZATION	GO:0007005		1.494	0.265	1.74E-08	1.482	0.272	5.13E-08	1.253	0.179	2.70E-12	0.754	0.226	0.704	0.241	0.031	0.976
GOBP_CYTOSKELETON_ORGANIZATION	GO:0007010		1.120	0.156	6.02E-13	1.142	0.156	2.53E-13	0.783	0.063	2.66E-35	2.007	0.022	2.131	0.017	-0.098	0.922
GOBP_ENDOMEMBRANE_SYSTEM_ORGANIZATION	GO:0010256		1.011	0.163	5.70E-10	1.038	0.193	7.18E-08	1.014	0.127	1.33E-15	-0.016	0.494	0.100	0.460	-0.105	0.917
GOBP_CELL_PROJECTION_ASSEMBLY	GO:0030031		0.940	0.158	2.56E-09	1.004	0.174	8.02E-09	0.792	0.102	7.14E-15	0.792	0.214	1.054	0.146	-0.272	0.786
GOBP_CELLULAR_COMPONENT_MORPHOGENESIS	GO:0032989	cell projection organization	0.873	0.104	5.69E-17	0.854	0.135	2.49E-10	1.112	0.109	2.04E-24	-1.584	0.057	-1.484	0.069	0.109	0.913
GOBP_CELL_JUNCTION_ORGANIZATION	GO:0034330		0.810	0.105	1.45E-14	0.919	0.163	1.56E-08	0.774	0.074	2.09E-25	0.273	0.393	0.811	0.209	-0.567	0.570
GOBP_PROTEIN_CONTAINING_COMPLEX_ORGANIZATION	GO:0043933		1.290	0.192	1.93E-11	1.307	0.203	1.12E-10	0.858	0.078	6.32E-28	2.079	0.019	2.065	0.019	-0.061	0.951
GOBP_SYNAPSE_ORGANIZATION	GO:0050808		0.769	0.123	3.45E-10	0.841	0.151	2.72E-08	0.881	0.101	3.89E-18	-0.700	0.242	-0.218	0.414	-0.368	0.713
GOBP_CHROMOSOME_ORGANIZATION	GO:0051276		1.221	0.176	3.99E-12	1.308	0.187	2.61E-12	0.943	0.092	8.20E-25	1.404	0.080	1.755	0.040	-0.338	0.735
GOBP_MEMBRANE_ORGANIZATION	GO:0061024		1.096	0.184	2.83E-09	1.079	0.181	2.72E-09	0.983	0.106	2.79E-20	0.531	0.298	0.457	0.324	0.066	0.948
GOBP_ORGANELLE_ASSEMBLY	GO:0070925		1.314	0.166	2.55E-15	1.316	0.192	6.81E-12	0.878	0.101	3.65E-18	2.242	0.012	2.019	0.022	-0.006	0.995
GOBP_DEFENSE_RESPONSE	GO:0006952		1.261	0.157	8.42E-16	1.336	0.195	6.51E-12	1.235	0.133	1.60E-20	0.126	0.450	0.430	0.334	-0.302	0.762
GOBP_RESPONSE_TO_OXIDATIVE_STRESS	GO:0006979		1.320	0.264	5.99E-07	1.320	0.237	2.38E-08	0.922	0.171	6.55E-08	1.263	0.103	1.364	0.086	-0.002	0.999
GOBP_RESPONSE_TO_RADIATION	GO:0009314		1.155	0.209	3.17E-08	1.248	0.237	1.48E-07	0.900	0.150	2.04E-09	0.994	0.160	1.239	0.108	-0.292	0.770
GOBP_RESPONSE_TO_XENOBIOTIC_STIMULUS	GO:0009410		1.083	0.207	1.59E-07	1.063	0.209	3.68E-07	1.129	0.214	1.26E-07	-0.154	0.439	-0.221	0.413	0.069	0.945
GOBP_RESPONSE_TO_WOUNDING	GO:0009611		1.008	0.197	3.22E-07	1.123	0.217	2.18E-07	0.917	0.114	7.64E-16	0.402	0.344	0.842	0.200	-0.390	0.696
GOBP_RESPONSE_TO_ABIOTIC_STIMULUS	GO:0009628		1.098	0.127	4.94E-18	1.139	0.159	6.79E-13	0.902	0.109	1.53E-16	1.174	0.120	1.233	0.109	-0.201	0.840
GOBP_RESPONSE_TO_ENDOGENOUS_STIMULUS	GO:0009719		1.047	0.123	2.27E-17	1.048	0.116	2.15E-19	1.009	0.070	8.01E-47	0.265	0.396	0.286	0.388	-0.007	0.994
GOBP_RESPONSE_TO_ORGANIC_CYCLIC_COMPOUND	GO:0014070		1.079	0.151	8.96E-13	1.149	0.210	4.20E-08	1.108	0.099	8.25E-29	-0.161	0.436	0.179	0.429	-0.273	0.784
GOBP_RESPONSE_TO_CYTOKINE	GO:0034097	cellular response to stress	1.181	0.213	2.82E-08	1.195	0.207	7.63E-09	1.360	0.156	3.06E-18	-0.678	0.249	-0.634	0.263	-0.049	0.961
GOBP_TAXIS	GO:0042330		0.955	0.140	8.00E-12	0.971	0.185	1.43E-07	1.279	0.170	5.83E-14	-1.471	0.071	-1.226	0.110	-0.070	0.944
GOBP_CELLULAR_RESPONSE_TO_ENDOGENOUS_STIMULUS	GO:0071495		1.020	0.123	1.21E-16	1.028	0.120	8.28E-18	1.014	0.076	2.37E-40	0.041	0.484	0.099	0.461	-0.047	0.963
GOBP_RESPONSE_TO_PEPTIDE	GO:1901652		1.040	0.170	8.72E-10	1.017	0.164	5.04E-10	1.024	0.149	5.71E-12	0.072	0.471	-0.030	0.488	0.097	0.923
GOBP_CELLULAR_RESPONSE_TO_PEPTIDE	GO:1901653		1.045	0.202	2.27E-07	1.037	0.185	2.00E-08	0.955	0.178	7.95E-08	0.334	0.369	0.319	0.375	0.030	0.976
GOBP_RESPONSE_TO_NITROGEN_COMPOUND	GO:1901698		1.087	0.145	5.71E-14	1.146	0.166	5.16E-12	0.998	0.097	6.66E-25	0.513	0.304	0.772	0.220	-0.268	0.789
GOBP_CELLULAR_RESPONSE_TO_NITROGEN_COMPOUND	GO:1901699		1.054	0.165	1.65E-10	1.068	0.177	1.48E-09	1.016	0.130	5.98E-15	0.182	0.428	0.241	0.405	-0.061	0.952
GOBP_RESPONSE_TO_OXYGEN_CONTAINING_COMPOUND	GO:1901700		1.156	0.132	1.77E-18	1.195	0.142	3.90E-17	1.024	0.078	1.26E-39	0.862	0.194	1.057	0.145	-0.203	0.839
GOBP_CELLULAR_RESPONSE_TO_OXYGEN_CONTAINING_COMPOUND	GO:1901701		1.156	0.148	4.65E-15	1.195	0.165	4.43E-13	1.027	0.099	4.57E-25	0.724	0.234	0.871	0.192	-0.176	0.861
GOBP_CARBOHYDRATE_METABOLIC_PROCESS	GO:0005975		1.224	0.235	1.94E-07	1.194	0.192	4.70E-10	0.849	0.114	8.27E-14	1.435	0.076	1.546	0.061	0.100	0.920
GOBP_GENERATION_OF_PRECURSOR_METABOLITES_AND_ENERGY	GO:0006091		1.493	0.286	1.76E-07	1.516	0.281	6.96E-08	1.315	0.187	2.23E-12	0.521	0.301	0.595	0.276	-0.057	0.954
GOBP_DNA_METABOLIC_PROCESS	GO:0006259		1.350	0.194	3.67E-12	1.457	0.213	8.36E-12	0.858	0.072	3.87E-33	2.379	0.009	2.665	0.004	-0.371	0.711
GOBP_RNA_PROCESSING	GO:0006396		1.249	0.193	9.31E-11	1.354	0.206	4.49E-11	0.948	0.111	1.24E-17	1.357	0.087	1.740	0.041	-0.371	0.710
GOBP_MRNA_PROCESSING	GO:0006397		1.171	0.173	1.38E-11	1.407	0.281	5.28E-07	1.089	0.181	1.66E-09	0.327	0.372	0.954	0.170	-0.718	0.473
GOBP_DEPHOSPHORYLATION	GO:0016311		1.001	0.197	3.50E-07	1.022	0.201	3.69E-07	0.861	0.141	1.12E-09	0.578	0.282	0.654	0.256	-0.074	0.941
GOBP_PROTEOLYSIS	GO:0006508		1.213	0.144	3.18E-17	1.266	0.150	2.89E-17	1.029	0.083	1.28E-35	1.114	0.133	1.386	0.083	-0.252	0.801
GOBP_LIPID_METABOLIC_PROCESS	GO:0006629		1.172	0.169	4.23E-12	1.155	0.148	5.61E-15	0.950	0.077	2.54E-35	1.197	0.116	1.233	0.109	0.075	0.940
GOBP_MACROMOLECULE_CATABOLIC_PROCESS	GO:0009057		1.289	0.178	3.84E-13	1.329	0.173	1.37E-14	0.818	0.070	1.24E-31	2.471	0.007	2.746	0.003	-0.162	0.872
GOBP_CELLULAR_MACROMOLECULE_BIOSYNTHETIC_PROCESS	GO:0009059		1.396	0.174	1.14E-15	1.408	0.183	1.25E-14	0.963	0.054	7.50E-72	2.376	0.009	2.338	0.010	-0.046	0.963
GOBP_MRNA_METABOLIC_PROCESS	GO:0016071	peptidyl-amino acid modification	1.148	0.156	1.83E-13	1.292	0.224	7.70E-09	0.989	0.131	4.60E-14	0.779	0.218	1.169	0.121	-0.529	0.597
GOBP_PHOSPHORYLATION	GO:0016310		1.043	0.114	6.19E-20	1.093	0.129	2.89E-17	0.894	0.078	1.55E-30	1.081	0.140	1.321	0.093	-0.290	0.772
GOBP_ORGANOPHOSPHATE_METABOLIC_PROCESS	GO:0019637		1.184	0.137	5.12E-18	1.145	0.181	2.26E-10	0.951	0.099	5.52E-22	1.381	0.084	0.943	0.173	0.172	0.863
GOBP_PROTEIN_CATABOLIC_PROCESS	GO:0030163		1.340	0.229	4.60E-09	1.401	0.223	3.20E-10	0.818	0.080	1.97E-24	2.156	0.016	2.464	0.007	-0.191	0.848
GOBP_MONOCARBOXYLIC_ACID_METABOLIC_PROCESS	GO:0032787		1.053	0.181	6.14E-09	1.054	0.192	3.94E-08	0.804	0.097	1.19E-16	1.215	0.112	1.164	0.122	-0.002	0.998
GOBP_AMIDE_BIOSYNTHETIC_PROCESS	GO:0043604		1.424	0.207	6.46E-12	1.557	0.262	2.96E-09	0.945	0.094	7.30E-24	2.106	0.018	2.197	0.014	-0.399	0.690
GOBP_CELLULAR_LIPID_METABOLIC_PROCESS	GO:0044255		1.218	0.204	2.54E-09	1.219	0.175	3.51E-12	0.988	0.098	4.60E-24	1.015	0.155	1.150	0.125	-0.003	0.998
GOBP_SMALL_MOLECULE_BIOSYNTHETIC_PROCESS	GO:0044283		1.177	0.222	1.18E-07	1.088	0.211	2.64E-07	0.862	0.103	6.74E-17	1.285	0.099	0.962	0.168	0.288	0.773
GOBP_NUCLEOBASE_CONTAINING_SMALL_MOLECULE_METABOLIC_PROCESS	GO:0055086		1.178	0.168	2.12E-12	1.142	0.204	2.32E-08	0.941	0.127	1.48E-13	1.127	0.130	0.836	0.201	0.136	0.892
GOBP_PROTEIN_MODIFICATION_BY_SMALL_PROTEIN_CONJUGATION_OR_REMOVAL	GO:0070646		1.292	0.173	9.52E-14	1.302	0.197	3.91E-11	0.920	0.090	1.20E-24	1.904	0.028	1.765	0.039	-0.039	0.969
GOBP_MAPK_CASCADE	GO:0000165		1.074	0.162	3.68E-11	1.097	0.188	5.68E-09	1.159	0.133	2.68E-18	-0.406	0.342	-0.268	0.394	-0.094	0.925
GOBP_REGULATION_OF_PROTEIN_PHOSPHORYLATION	GO:0001932		1.117	0.148	4.48E-14	1.177	0.180	6.45E-11	0.955	0.102	6.59E-21	0.905	0.183	1.074	0.141	-0.256	0.798
GOBP_REGULATION_OF_IMMUNE_SYSTEM_PROCESS	GO:0002682		1.122	0.145	9.76E-15	1.240	0.168	1.66E-13	0.948	0.095	1.32E-23	1.004	0.158	1.514	0.065	-0.534	0.594
GOBP_IMMUNE_RESPONSE	GO:0002764		1.205	0.157	1.56E-14	1.279	0.164	5.26E-15	1.071	0.117	5.47E-20	0.684	0.247	1.035	0.150	-0.328	0.743
GOBP_ENZYME_LINKED_RECEPTOR_PROTEIN_SIGNALING_PATHWAY	GO:0007167		1.018	0.119	9.86E-18	1.029	0.142	5.00E-13	0.922	0.087	2.56E-26	0.651	0.257	0.638	0.262	-0.058	0.954
GOBP_G_PROTEIN_COUPLED_RECEPTOR_SIGNALING_PATHWAY	GO:0007186		0.999	0.162	7.24E-10	1.011	0.142	9.35E-13	1.261	0.165	2.22E-14	-1.134	0.128	-1.152	0.125	-0.055	0.956
GOBP_CELL_CELL_SIGNALING	GO:0007267		1.054	0.123	1.44E-17	1.113	0.155	7.62E-13	1.044	0.087	2.40E-33	0.066	0.474	0.388	0.349	-0.297	0.766
GOBP_POSITIVE_REGULATION_OF_CELL_POPULATION_PROLIFERATION	GO:0008284		1.116	0.169	4.44E-11	1.162	0.189	7.52E-10	1.134	0.116	1.70E-22	-0.088	0.465	0.125	0.450	-0.180	0.857
GOBP_NEGATIVE_REGULATION_OF_BIOSYNTHETIC_PROCESS	GO:0009890		1.161	0.175	3.16E-11	1.105	0.154	6.42E-13	1.163	0.113	8.25E-25	-0.012	0.495	-0.307	0.380	0.240	0.810
GOBP_REGULATION_OF_CATABOLIC_PROCESS	GO:0009894		1.115	0.174	1.40E-10	1.145	0.156	1.78E-13	1.014	0.116	1.99E-18	0.483	0.315	0.676	0.249	-0.130	0.896
GOBP_CELL_CYCLE_PROCESS	GO:0010564		1.335	0.226	3.63E-09	1.394	0.219	2.04E-10	0.898	0.069	1.57E-38	1.849	0.032	2.159	0.015	-0.187	0.852
GOBP_BIOLOGICAL_ADHESION	GO:0022409		0.962	0.121	1.74E-15	0.988	0.114	4.18E-18	1.018	0.081	9.01E-36	-0.377	0.353	-0.208	0.418	-0.156	0.876
GOBP_REGULATION_OF_CELL_ADHESION	GO:0030155		0.993	0.165	1.76E-09	0.989	0.161	7.80E-10	0.869	0.103	2.91E-17	0.639	0.262	0.630	0.264	0.017	0.986
GOBP_REGULATION_OF_PROTEOLYSIS	GO:0030162		1.191	0.207	8.84E-09	1.267	0.241	1.45E-07	0.952	0.113	3.36E-17	1.016	0.155	1.183	0.118	-0.236	0.813
GOBP_REGULATION_OF_PROTEIN_MODIFICATION_PROCESS	GO:0031399	regulation of intracellular signal transduction	1.121	0.120	1.29E-20	1.160	0.150	1.17E-14	0.952	0.080	1.69E-32	1.169	0.121	1.221	0.111	-0.202	0.840
GOBP_REGULATION_OF_CELLULAR_COMPONENT_SIZE	GO:0032535		0.895	0.171	1.79E-07	0.901	0.167	6.51E-08	0.783	0.112	2.19E-12	0.546	0.293	0.586	0.279	-0.024	0.981
GOBP_REGULATION_OF_ACTIN_FILAMENT_BASED_PROCESS	GO:0032970		0.871	0.153	1.11E-08	0.864	0.169	3.26E-07	0.681	0.085	9.39E-16	1.092	0.137	0.967	0.167	0.034	0.973
GOBP_REGULATION_OF_ORGANELLE_ORGANIZATION	GO:0033043		1.044	0.133	4.33E-15	1.090	0.138	3.30E-15	0.974	0.102	9.24E-22	0.418	0.338	0.673	0.251	-0.237	0.813
GOBP_REGULATION_OF_GROWTH	GO:0040008		1.013	0.151	2.07E-11	1.003	0.154	7.51E-11	1.200	0.149	8.93E-16	-0.877	0.190	-0.918	0.179	0.049	0.961
GOBP_POSITIVE_REGULATION_OF_LOCOMOTION	GO:0040017		1.075	0.193	2.49E-08	0.987	0.176	2.10E-08	0.802	0.104	1.45E-14	1.244	0.107	0.905	0.183	0.335	0.738
GOBP_REGULATION_OF_CELL_POPULATION_PROLIFERATION	GO:0042127		1.197	0.142	3.63E-17	1.229	0.157	4.55E-15	1.054	0.081	4.30E-39	0.876	0.190	0.991	0.161	-0.149	0.882
GOBP_NEGATIVE_REGULATION_OF_CELL_DEATH	GO:0043069		1.283	0.211	1.24E-09	1.315	0.223	3.94E-09	1.255	0.122	5.35E-25	0.113	0.455	0.235	0.407	-0.104	0.917
GOBP_POSITIVE_REGULATION_OF_MOLECULAR_FUNCTION	GO:0044093		0.971	0.091	2.14E-26	1.015	0.128	2.03E-15	0.970	0.079	9.10E-35	0.006	0.498	0.296	0.383	-0.279	0.781
GOBP_REGULATION_OF_CELL_DIFFERENTIATION	GO:0045595		1.175	0.146	1.07E-15	1.153	0.147	3.80E-15	1.048	0.070	6.49E-51	0.781	0.217	0.649	0.258	0.103	0.918
GOBP_REGULATION_OF_TRANSPORT	GO:0051049		1.052	0.113	1.63E-20	1.103	0.133	9.87E-17	0.933	0.064	8.31E-48	0.916	0.180	1.155	0.124	-0.292	0.770
GOBP_REGULATION_OF_PHOSPHORUS_METABOLIC_PROCESS	GO:0051174		1.086	0.123	8.87E-19	1.151	0.153	6.30E-14	0.918	0.087	6.13E-26	1.114	0.133	1.319	0.094	-0.332	0.740
GOBP_POSITIVE_REGULATION_OF_RNA_METABOLIC_PROCESS	GO:0051254		1.109	0.122	8.50E-20	1.093	0.137	1.67E-15	1.028	0.074	6.54E-44	0.570	0.284	0.414	0.339	0.091	0.928
GOBP_REGULATION_OF_CELL_CYCLE	GO:0051726		1.349	0.260	2.02E-07	1.404	0.257	4.75E-08	1.079	0.087	4.34E-35	0.988	0.162	1.197	0.116	-0.148	0.882
GOBP_REGULATION_OF_ANATOMICAL_STRUCTURE_SIZE	GO:0090066		0.910	0.151	1.60E-09	0.988	0.191	2.35E-07	0.847	0.104	3.97E-16	0.342	0.366	0.647	0.259	-0.321	0.748
GOBP_SYNAPTIC_SIGNALING	GO:0099536		0.917	0.132	4.08E-12	1.018	0.199	3.03E-07	0.955	0.089	9.21E-27	-0.239	0.405	0.289	0.386	-0.424	0.672
GOBP_VASCULATURE_DEVELOPMENT	GO:0001944		1.117	0.150	1.03E-13	1.151	0.188	8.46E-10	1.108	0.124	4.59E-19	0.045	0.482	0.189	0.425	-0.140	0.889
GOBP_IMMUNE_SYSTEM_DEVELOPMENT	GO:0002520		1.220	0.172	1.21E-12	1.380	0.199	4.60E-12	1.008	0.096	5.03E-26	1.081	0.140	1.683	0.046	-0.607	0.544
GOBP_LEUKOCYTE_DIFFERENTIATION	GO:0002521		1.162	0.233	6.04E-07	1.349	0.227	2.98E-09	0.973	0.118	1.68E-16	0.725	0.234	1.468	0.071	-0.574	0.566
GOBP_DEVELOPMENTAL_PROCESS_INVOLVED_IN_REPRODUCTION	GO:0003006		1.289	0.219	4.22E-09	1.384	0.236	4.70E-09	1.129	0.130	4.87E-18	0.624	0.266	0.944	0.173	-0.296	0.767
GOBP_EMBRYO_DEVELOPMENT	GO:0009790		1.122	0.149	5.42E-14	1.088	0.146	8.86E-14	1.005	0.086	8.68E-32	0.680	0.248	0.493	0.311	0.161	0.872
GOBP_EMBRYO_DEVELOPMENT_ENDING_IN_BIRTH_OR_EGG_HATCHING	GO:0009792		1.181	0.176	1.76E-11	1.169	0.181	1.11E-10	1.088	0.110	5.08E-23	0.448	0.327	0.383	0.351	0.047	0.963
GOBP_DEVELOPMENTAL_MATURATION	GO:0021700		1.015	0.202	4.81E-07	0.984	0.196	5.28E-07	1.309	0.246	1.04E-07	-0.925	0.178	-1.035	0.150	0.112	0.911
GOBP_NEUROGENESIS	GO:0022008		0.972	0.102	2.17E-21	0.971	0.120	4.69E-16	1.023	0.070	4.34E-48	-0.412	0.340	-0.370	0.356	0.002	0.999
GOBP_TUBE_MORPHOGENESIS	GO:0035239	tissue development	1.027	0.119	5.27E-18	1.075	0.169	1.84E-10	1.143	0.113	6.60E-24	-0.702	0.241	-0.332	0.370	-0.231	0.817
GOBP_TUBE_DEVELOPMENT	GO:0035295		1.046	0.115	1.20E-19	1.080	0.153	1.64E-12	1.222	0.116	5.05E-26	-1.073	0.142	-0.737	0.231	-0.177	0.859
GOBP_MULTI_ORGANISM_REPRODUCTIVE_PROCESS	GO:0044703		1.330	0.226	3.71E-09	1.455	0.237	7.81E-10	1.143	0.129	8.67E-19	0.721	0.236	1.158	0.123	-0.382	0.703
GOBP_LYMPHOCYTE_ACTIVATION	GO:0046649		1.058	0.200	1.15E-07	1.203	0.238	4.38E-07	0.910	0.099	4.84E-20	0.666	0.253	1.135	0.128	-0.465	0.642
GOBP_DEVELOPMENTAL_GROWTH	GO:0048589		0.890	0.137	7.23E-11	0.835	0.125	2.07E-11	1.171	0.140	6.09E-17	-1.434	0.076	-1.791	0.037	0.298	0.766
GOBP_ANATOMICAL_STRUCTURE_FORMATION_INVOLVED_IN_MORPHOGENESIS	GO:0048646		1.141	0.146	4.91E-15	1.155	0.163	1.39E-12	1.143	0.105	2.09E-27	-0.014	0.495	0.062	0.475	-0.067	0.947
GOBP_NERVOUS_SYSTEM_PROCESS	GO:0050877		1.132	0.150	4.43E-14	1.143	0.168	9.75E-12	0.952	0.087	6.46E-28	1.041	0.149	1.012	0.156	-0.048	0.962
GOBP_HEAD_DEVELOPMENT	GO:0060322		0.939	0.142	3.31E-11	0.967	0.170	1.18E-08	0.972	0.080	9.35E-34	-0.201	0.420	-0.025	0.490	-0.127	0.899
GOBP_EPITHELIUM_DEVELOPMENT	GO:0060429		1.040	0.140	9.10E-14	1.062	0.127	6.27E-17	1.038	0.086	1.12E-33	0.014	0.494	0.162	0.436	-0.119	0.905
GOBP_MUSCLE_STRUCTURE_DEVELOPMENT	GO:0061061		1.046	0.179	5.47E-09	0.976	0.152	1.45E-10	0.916	0.106	4.67E-18	0.622	0.267	0.320	0.374	0.298	0.766
GOBP_ION_TRANSPORT	GO:0006811		1.056	0.117	1.27E-19	1.052	0.118	4.78E-19	1.026	0.075	4.35E-43	0.222	0.412	0.190	0.425	0.026	0.980
GOBP_ENDOCYTOSIS	GO:0006897		0.999	0.177	1.80E-08	1.002	0.161	4.74E-10	0.972	0.119	4.14E-16	0.128	0.449	0.150	0.440	-0.012	0.991
GOBP_ION_TRANSMEMBRANE_TRANSPORT	GO:0034220		1.096	0.138	2.32E-15	1.088	0.142	1.54E-14	1.010	0.085	7.96E-33	0.526	0.299	0.471	0.319	0.038	0.969
GOBP_INTRACELLULAR_TRANSPORT	GO:0046907		1.283	0.154	8.96E-17	1.345	0.174	1.28E-14	1.023	0.085	2.00E-33	1.480	0.069	1.660	0.048	-0.265	0.791
GOBP_ORGANELLE_LOCALIZATION	GO:0051640		1.042	0.153	1.01E-11	1.150	0.187	7.56E-10	1.197	0.196	9.80E-10	-0.625	0.266	-0.171	0.432	-0.451	0.652
GOBP_ESTABLISHMENT_OF_ORGANELLE_LOCALIZATION	GO:0051656	vesicle-mediated transport	1.100	0.187	3.99E-09	1.172	0.235	6.30E-07	1.279	0.246	1.88E-07	-0.580	0.281	-0.317	0.376	-0.237	0.812
GOBP_LOCALIZATION_WITHIN_MEMBRANE	GO:0051668		1.125	0.205	3.81E-08	1.104	0.221	5.62E-07	0.892	0.097	2.85E-20	1.028	0.152	0.877	0.190	0.072	0.943
GOBP_TRANSMEMBRANE_TRANSPORT	GO:0055085		1.100	0.123	4.13E-19	1.106	0.133	1.04E-16	1.005	0.077	6.21E-39	0.657	0.256	0.658	0.255	-0.033	0.974
GOBP_NITROGEN_COMPOUND_TRANSPORT	GO:0071705		1.132	0.112	3.24E-24	1.165	0.136	8.59E-18	0.963	0.067	8.80E-47	1.301	0.097	1.336	0.091	-0.187	0.852

GO Name	GO ID	Representative Domain	Infection			Hospitalization			Severe Symptoms			Infection vs Severe Symptoms		Hospitalization vs. Severe Symptoms		Infection vs Hospitalization	
			Enrich	SE	p	Enrich	SE	p	Enrich	SE	p	z	p	z	p	z	p
GOBP_INORGANIC_ION_TRANSMEMBRANE_TRANSPORT	GO:0098660		1.056	0.150	2.18E-12	1.081	0.169	1.62E-10	1.078	0.101	1.81E-26	-0.120	0.452	0.016	0.494	-0.110	0.912
GOBP_CATION_TRANSMEMBRANE_TRANSPORT	GO:0098662		1.106	0.158	2.84E-12	1.122	0.167	2.09E-11	1.054	0.097	1.59E-27	0.284	0.388	0.351	0.363	-0.066	0.947
GOBP_MITOTIC_CELL_CYCLE	GO:0000278		1.401	0.273	2.89E-07	1.463	0.249	4.15E-09	0.816	0.079	2.76E-25	2.057	0.020	2.478	0.007	-0.168	0.866
GOBP_CELL_ACTIVATION	GO:0001775		1.069	0.152	2.30E-12	1.253	0.210	2.23E-09	0.918	0.087	4.82E-26	0.860	0.195	1.476	0.070	-0.709	0.478
GOBP_PEPTIDE_METABOLIC_PROCESS	GO:0006518		1.505	0.249	1.59E-09	1.543	0.270	1.05E-08	0.940	0.097	5.06E-22	2.108	0.018	2.100	0.018	-0.103	0.918
GOBP_MICROTUBULE_BASED_PROCESS	GO:0007017		1.333	0.187	1.12E-12	1.451	0.224	9.32E-11	1.092	0.145	4.37E-14	1.016	0.155	1.345	0.089	-0.404	0.686
GOBP_CELL_CYCLE	GO:0007049		1.394	0.229	1.24E-09	1.426	0.210	1.10E-11	0.979	0.061	2.92E-58	1.749	0.040	2.046	0.020	-0.103	0.918
GOBP_REGULATION_OF_CELL_DEATH	GO:0008219		1.252	0.180	3.95E-12	1.290	0.179	5.97E-13	1.143	0.094	6.69E-34	0.539	0.295	0.727	0.234	-0.146	0.884
GOBP_TISSUE_DEVELOPMENT	GO:0009888		1.108	0.117	3.10E-21	1.093	0.109	1.25E-23	1.035	0.074	3.59E-44	0.527	0.299	0.443	0.329	0.091	0.928
GOBP_PROGRAMMED_CELL_DEATH	GO:0012501		1.199	0.156	1.57E-14	1.253	0.154	4.85E-16	1.171	0.094	1.21E-35	0.156	0.438	0.455	0.325	-0.245	0.806
GOBP_VESICLE_MEDIATED_TRANSPORT	GO:0016192		1.067	0.137	7.67E-15	1.150	0.154	7.21E-14	0.958	0.075	1.69E-37	0.695	0.244	1.124	0.131	-0.405	0.685
GOBP_CELL_MIGRATION	GO:0016477		0.954	0.107	6.72E-19	0.916	0.105	2.28E-18	0.977	0.081	7.39E-34	-0.166	0.434	-0.463	0.322	0.259	0.796
GOBP_PEPTIDYL_AMINO_ACID_MODIFICATION	GO:0018193		1.117	0.122	5.40E-20	1.238	0.173	7.70E-13	0.991	0.096	6.59E-25	0.809	0.209	1.248	0.106	-0.573	0.567
GOBP_ACTIN_FILAMENT_BASED_PROCESS	GO:0030029		1.002	0.195	2.73E-07	0.998	0.186	7.63E-08	0.672	0.068	6.68E-23	1.596	0.055	1.645	0.050	0.016	0.987
GOBP_CELL_PROJECTION_ORGANIZATION	GO:0030030		0.910	0.088	5.52E-25	0.927	0.109	1.91E-17	1.025	0.071	1.37E-47	-1.024	0.153	-0.756	0.225	-0.124	0.901
GOBP_CELLULAR_RESPONSE_TO_STRESS	GO:0033554	null	1.239	0.162	1.74E-14	1.310	0.147	5.20E-19	0.961	0.073	3.29E-39	1.570	0.058	2.125	0.017	-0.323	0.747
GOBP_GROWTH	GO:0040007		0.974	0.133	2.52E-13	0.929	0.118	2.67E-15	1.100	0.119	1.61E-20	-0.707	0.240	-1.027	0.152	0.256	0.798
GOBP_LOCOMOTION	GO:0040011		0.977	0.101	3.66E-22	0.973	0.103	3.24E-21	1.024	0.072	1.74E-46	-0.378	0.353	-0.407	0.342	0.030	0.976
GOBP_HOMEOSTATIC_PROCESS	GO:0042592		1.160	0.137	2.96E-17	1.178	0.131	2.25E-19	0.997	0.077	1.89E-38	1.035	0.150	1.195	0.116	-0.098	0.922
GOBP_PEPTIDE_BIOSYNTHETIC_PROCESS	GO:0043043		1.610	0.263	9.39E-10	1.701	0.316	7.06E-08	0.972	0.106	3.68E-20	2.251	0.012	2.191	0.014	-0.222	0.825
GOBP_CELLULAR_AMIDE_METABOLIC_PROCESS	GO:0043603		1.430	0.197	3.65E-13	1.486	0.224	3.01E-11	0.926	0.083	6.61E-29	2.360	0.009	2.347	0.009	-0.187	0.852
GOBP_SMALL_MOLECULE_METABOLIC_PROCESS	GO:0044281		1.194	0.118	3.09E-24	1.155	0.131	1.37E-18	0.999	0.146	7.08E-12	1.042	0.149	0.795	0.213	0.222	0.824
GOBP_BIOLOGICAL_PROCESS_INVOLVED_IN_INTERSPECIES_INTERACTION_BETWEEN_ORGANISMS	GO:0044419		1.305	0.169	1.12E-14	1.375	0.195	1.81E-12	1.142	0.115	3.95E-23	0.799	0.212	1.031	0.151	-0.273	0.785
GOBP_CHEMICAL_HOMEOSTASIS	GO:0048878		1.178	0.173	9.08E-12	1.191	0.173	5.40E-12	1.102	0.105	5.83E-26	0.378	0.353	0.443	0.329	-0.054	0.957
GOBP_PROCESS_UTILIZING_AUTOPHAGIC_MECHANISM	GO:0061919		1.130	0.180	3.54E-10	1.182	0.209	1.47E-08	1.174	0.202	6.64E-09	-0.163	0.435	0.027	0.489	-0.188	0.851
GOBP_PURINE_CONTAINING_COMPOUND_METABOLIC_PROCESS	GO:0072521		1.062	0.166	1.71E-10	1.008	0.201	5.05E-07	0.984	0.138	1.17E-12	0.361	0.359	0.097	0.461	0.209	0.834
GOBP_CELL_CELL_ADHESION	GO:0098609		0.921	0.130	1.46E-12	0.950	0.138	5.93E-12	1.036	0.097	1.85E-26	-0.705	0.240	-0.508	0.306	-0.151	0.880
GOBP_CARBOHYDRATE_DERIVATIVE_METABOLIC_PROCESS	GO:1901135		1.192	0.181	4.72E-11	1.129	0.156	4.75E-13	0.874	0.080	7.53E-28	1.605	0.054	1.451	0.073	0.265	0.791
GOBP_REGULATION_OF_INTRACELLULAR_SIGNAL_TRANSDUCTION	GO:1902531		1.012	0.106	1.14E-21	1.031	0.116	8.53E-19	1.039	0.085	2.07E-34	-0.198	0.422	-0.057	0.477	-0.118	0.906

Supplemental Table 9: Results of the drug-repurposing analysis based on gene ontologies shared between COVID-19 outcomes (severe respiratory symptoms, 13,769 cases and 1,072,442 controls; hospitalization 32,519 cases and 2,062,805 controls; infection, 122,616 cases and 2,475,240 controls) and brain imaging-derived phenotypes (IDP, assessed in 33,224 participants).. Findings surviving false discovery rate multiple testing correction (FDR q<0.05) are reported. Statistical significance was defined using the Kolmogorov-Smirnov method. ES: Enrichment Score.

Drug	Description	ES	p	FDR q	Top GO Term
ribavirin	antiviral medication	-0.519	4.15E-04	0.038	kinase activity
canadine	alkaloid used for the treatment or prevention of cardiac arrhythmias	-0.504	6.68E-04	0.038	phosphoric ester hydrolase activity
oligomycin	antibiotic that inhibits mitochondrial ATP synthase	-0.504	6.69E-04	0.038	adenyl nucleotide binding
decitabine	medication for the treatment of myelodysplastic syndromes	-0.500	7.67E-04	0.040	ion transmembrane transporter activity
indapamide	thiazide-like diuretic	-0.497	8.48E-04	0.043	phosphoric ester hydrolase activity
epivincamine	alkaloid used as as a vasodilator and antihypertensive agent	-0.468	4.25E-04	0.038	nucleolus
nafcillin	second-generation beta-lactam antibiotic of the penicillin class	-0.457	6.34E-04	0.038	chromatin
metolazone	thiazide diuretic	-0.440	1.16E-03	0.046	chromatin
esculetin	natural coumarin derivative with antioxidant, anti-inflammatory, and anticancer properties	-0.436	1.32E-03	0.047	Golgi membrane
PNU-0251126	perturbagen signature curated by the drug signature database	-0.432	1.51E-03	0.048	cell body
ceforanide	second-generation cephalosporin antibiotic	-0.207	2.40E-04	0.038	immune response
genistein	phytoestrogenic isoflavone with antioxidant properties	-0.198	5.17E-04	0.038	intracellular protein transport
amikacin	antibiotic medication used for bacterial infections	-0.197	5.49E-04	0.038	mitotic cell cycle
levocabastine	second-generation antihistamine medication	-0.195	6.47E-04	0.038	mRNA metabolic process
sulfanilamide	short-acting sulfonamide antibiotic	0.185	1.47E-03	0.048	neuron development
bucladesine	cyclic nucleotide derivative that can mimic endogenous cAMP and inhibit phosphodiesterase enzyme	0.185	1.42E-03	0.048	central nervous system development
W-13	calmodulin antagonist	0.186	1.37E-03	0.047	regulation of cell differentiation
fosfosal	non-acetylated salicylic acid derivative used as an analgesic and anti-inflammatory agent	0.186	1.37E-03	0.047	mRNA metabolic process
chlorprothixene	antipsychotic drug of the thioxanthine class	0.187	1.28E-03	0.047	mRNA metabolic process
gefitinib	tyrosine kinase inhibitor used as first-line therapy to treat certain forms of carcinoma	0.188	1.21E-03	0.047	ion transport
Prestwick-685	compound with anti-infective properties	0.189	1.05E-03	0.044	response to organic cyclic compound
proscillaridin	cardiac glycoside used in the treatment of congestive heart failure and cardiac arrhythmia	0.190	9.92E-04	0.043	immune response
SC-560	potent and selective inhibitor of Cyclooxygenase-1 enzyme	0.191	9.42E-04	0.043	mitotic cell cycle
ouabain	cardiac glycoside used to treat treat hypotension and arrhythmias	0.191	9.26E-04	0.043	immune response
ranitidine	histamine-2 blocker used to decrease the amount of acid created by the stomach	0.192	8.77E-04	0.043	mRNA metabolic process
MG-132	proteasome inhibitor used to study protein degradation and apoptosis	0.193	7.65E-04	0.040	mRNA metabolic process
lanatoside_C	a cardiac glycoside used in the treatment of congestive heart failure and cardiac arrhythmia	0.195	6.68E-04	0.038	immune response
nortriptyline	antidepressant medication also used to treat chronic pain	0.196	6.09E-04	0.038	intracellular protein transport
helveticoside	steroid glycoside used as antineoplastic and cardioprotective agent	0.202	3.84E-04	0.038	immune response
hydroquinine	organic compound used as antimalarial agent and antibacterial among other applications	0.202	3.81E-04	0.038	innate immune response
niclosamide	anthelmintic medication used to treat tapeworm infestations	0.202	3.69E-04	0.038	immune response
DL-thiorphan	specific and potent inhibitor of enkephalinase	0.205	2.74E-04	0.038	mRNA metabolic process
2,6-dimethylpiperidine	chemical compound used in the synthesis of anti-oxidant compounds	0.206	2.65E-04	0.038	ion transport
clozapine	antipsychotic medication	0.209	2.02E-04	0.038	embryo development ending in birth or egg hatching
minoxidil	medication used for the treatment of high blood pressure and pattern hair loss	0.213	1.39E-04	0.038	response to radiation
digitoxigenin	cardiotonic used to increase cardiac contractility in the treatment of heart failure	0.215	1.21E-04	0.038	immune response
tetryzoline	alpha-adrenergic agonist used to relieve eye redness and nasal congestion	0.223	5.49E-05	0.036	innate immune response
methylbenzethonium_chloride	quaternary ammonium compound used as a disinfectant and preservative in various products	0.228	3.40E-05	0.036	intracellular protein transport
palmatine	alkaloid with anti-cancer, anti-inflammatory, and neuroprotective properties	0.458	6.18E-04	0.038	synapse
methazolamide	carbonic anhydrase inhibitor used to treat certain forms of glaucoma	0.489	1.08E-03	0.044	cell adhesion molecule binding
urapidil	sympatholytic antihypertensive drug	0.504	6.76E-04	0.038	enzyme regulator activity
